# Supplementary material for: Perceived access to PrEP as a critical step in engagement: A qualitative analysis and discrete choice experiment among young men who have sex with men
Source: PLoS One. 2022 Jan 26;17(1):e0258530. doi: 10.1371/journal.pone.0258530 (PMC8791519; doi:10.1371/journal.pone.0258530)
Supplement: S3 File — (DOCX) [file pone.0258530.s003.docx]

**Appendix 1.**

**Information shown to participants during screening, screening flow, and closed-ended questionnaire for participants after completion of the discrete choice experiments.**

1. **Screening**

**Welcome to the DiCE for PrEP Study**

You have entered this site because you are interested in participating in a research study on PrEP. To see whether you might be eligible to participate in this study, we would like to ask you some questions about yourself. Some of these may be sensitive. Please try to answer them as honestly as you can.

OK, here we go:

1. So we know you’re not a robot:

What is 2 + 3? ____

1. Check the box for the age group you fall into:

- Less than 18 years old
- 18-30 years old
- 31-40 years old
- 41-50 years old
- Older than age 50

***[Did not answer 2+3 correctly=ineligible]***

***[incorrect age/range = ineligible]***

1. Where do you live? (another option for this question is to actually have a drop-down list for countries)
   - United States or U.S. territories
     1. If yes, drop down to state
   - Europe
   - Asia, including the Indian subcontinent
   - Africa, below the Sahara
   - North Africa or the Middle East
   - Pacific Islands
   - Australia
   - Other
2. Which of the following best describes you?
   - Male
   - Female
   - Trans man
   - Trans woman
   - Other

***[Displayed if respondent is not male, age 18-30, does not live in the U.S. or U.S. territories: “Thanks for your interest, but based on your responses, you’re not eligible for this study at this time.”]***

1. Are you currently taking or have you ever taken PrEP? This includes the pill Truvada, or any PrEP medication you may be taking as part of a study.
   - Yes
   - No
2. To your knowledge, are you living with HIV (HIV-positive)?
   - Yes
   - No

***[Displayed if respondent is currently taking or has taken PrEP or is HIV-positive: “Thanks for your interest, but based on your responses, you’re not eligible for this study at this time.”]***

Because PrEP is a medication to prevent HIV, we need to know something about your risk for HIV. These next questions ask about your HIV risk.

1. Have you had sex in the last 6 months (oral sex, anal sex, or vaginal sex)?

- Yes
- No

***[If yes, then ask next question]***

***[If no then go to message below related to ineligibility]***

1. Have you had anal sex (either as a top or bottom) **with another man** in the last 6 months?

- Yes, as a top (my penis in my partner’s anus or butt)
- Yes, as a bottom (my partner’s penis in my anus or butt)
- Yes, as both a top and a bottom
- No anal sex with another man in the last 6 months

***[Any yes response is potentially eligible, pending remaining questions. No is ineligible.]***

1. With how many **male** partners did you have **anal** sex in the last 6 months?
   - 0
   - 1
   - More than 1
2. Did you use a condom each time you had anal sex in the last 6 months?
   - Yes, I used a condom each time I had anal sex
   - No, I did not use a condom each time I had anal sex
   - I did not have anal sex in the last 6 months

***[Answer must be “No, I did not use a condom each time I had anal sex” in order to be eligible; other answers are ineligible]***

***[Must have had more than 1 partner to be eligible UNLESS answers to questions 11 are “Yes” or “Unsure” OR answer to 12 is “Yes”]***

1. Over the last 6 months, did any of your partner(s) have other partners besides you?
   - Yes
   - No
   - Not sure
2. In the last 6 months, were any of your partners HIV-positive?
   - Yes
   - No
   - Not sure

***[Displayed if respondent has 1) not had condomless anal sex with at least 1 male partner in the last 6 months and 2) the respondent or a partner has had at least 1 other partner or is not known to be mutually monogamous (“Yes” or “not sure” to question 11) UNLESS the partner is known to be HIV-positive: “Thanks for your interest, but based on your responses, you’re not eligible for this study at this time.”]***

***[Displayed if respondent meets all eligibility criteria: Thanks for your responses. Based on what you’ve told us, you are eligible to participate in this study. Please let us know if you’re interested in learning more about participating in this study. If you are, you will be asked to review a consent form first. The consent form will give you information about the study and what it would mean for you to participate.]***

- *Yes, I would like to review the consent form.*
- *No, thank you, I’m not interested in participating at this time.*

1. **Informed Consent (see separate informed consent form)**
2. **The Discrete Choice Experiment**

*Note to IRB: Discrete Choice Experiments evaluated preferences for specific attributes (features of a good or service) and levels (different amounts or types of each attribute). Subjects will be shown a series of 10 choice tasks in Section 1 and 12 choice tasks in Section II. Each participant may get a slightly different set of choice tasks, but they will all see some combination of the same attributes. The below are possible combinations that a subject might see. For a full list of attributes and levels, see the end of this document.*

**Before you start, here is some information to get you oriented.**

From now on, we will be using the word **PrEP** to describe any medication that can be taken to prevent HIV. As it stands now in the real world, the only available form of PrEP is a pill that should be taken every day. But, other forms of PrEP are being studied and may become available in the future and so part of our survey will be dedicated to asking about these possible future options.

***[Prior to Step 1: PrEP Access questions]***

**Discrete Choice Experiments for PrEP Step 1: PrEP Access**

- Some people may find it easy to get access to PrEP. They may already have medical insurance covering PrEP and they have a clinic close by that offers PrEP. Others might have more difficulties accessing PrEP.
- In this next section, we’d like you to think about aspects of PrEP access such as insurance coverage options, how long it would take you to get on PrEP, and where you would get your care. We’ll be showing you pairs of options for accessing PrEP. Choose which set of options you like best. If you don’t like either, select the “No PrEP” option.

***[Example Step 1 DCE]***

| **PrEP Option A** | **PrEP Option B** | **No PrEP** |
| --- | --- | --- |
| **You need to get a new insurance plan that covers PrEP** | **PrEP is covered by your current insurance** |  |
| **Start PrEP at first appointment** | **Start PrEP after 4 weeks lab and paperwork processing time** |  |
| **Costs you $50 per month** | **No cost to you** |  |
| **Your insurance information is completely private** | **Parents, spouse or employer may know you are on PrEP through insurance claims** |  |
| **See provider in the office** | **Communicate with provider online (requires visits to lab only)** |  |

***[Prior to Step 2: PrEP mode, frequency, and delivery questions]***

**Discrete Choice Experiments for PrEP Step 2: Other PrEP Options**

- The next set of questions is about what types of medicine you might prefer (a pill vs. an injection, for example), how often you might prefer to take PrEP (every day vs. once a week, for example), and other features of PrEP.
- For the next set of questions, ***please imagine that you do have at least some insurance coverage and a place to access PrEP***.
- Your costs, in this section, refer to your TOTAL overall costs per month for PrEP, including copays for provider visits, laboratory tests, and the medication itself.
- The efficacy in preventing HIV refers to the efficacy when taken as prescribed. For example, if we say a daily pill is 90% effective, we mean that it is 90% effective only when taken every day. Typically, taking PrEP less often than prescribed makes it less effective.
- As before, choose the set of options you like the best, or select the “No PrEP” option.

| **PrEP Option A** | **PrEP Option B** | **No PrEP** |
| --- | --- | --- |
| **injection** | **pill** |  |
| **used weekly** | **used daily** |  |
| **costs $50 per month** | **costs $20 per month** |  |
| **90% effective** | **85% effective** |  |
| **Brief headaches and nausea for 1 in 100 people** | **No side effects** |  |

**Self-Administered Questionnaire**

These questions will help us understand your potential risk for HIV.

Some of the questions ask about sensitive topics. In order to understand our results, it is important that you answer all the questions as honestly as you can.

1. What race(s) do you consider your race to be (check all that apply):
   - Asian/ South Asian/ East Asian
   - Black/ African/ African American/ Black Caribbean
   - Native American/ American Indian/ Native Alaskan
   - Native Hawaiian/ Pacific Islander
   - White/ Caucasian
   - Other, please describe:
   - (free text for other)
   - Prefer not to answer
2. Do you consider yourself Hispanic or Latino/a/x?
   - Yes
   - No
   - Prefer not to answer
3. Please enter your year of birth ______

- Prefer not to answer

1. How would you describe the area you live in (where you spend MOST of your time)
   - A large city (about 500,000 population or more)
   - A medium sized city (about 150-500,000 population)
   - A small city (about 30-150,000 population)
   - A rural area
   - Prefer not to answer
2. Are you currently a student?
   - Yes, full-time student
   - Yes, part-time student
   - No, not a student
   - Prefer not to answer
3. Are you currently employed?
   - Yes, employed full time
   - Yes, employed part-time
   - No, not employed
   - Prefer not to answer
4. What is the highest level of education you have completed?
   - Some high school
   - High school diploma or GED
   - High school diploma plus some college/university
   - College/university degree
   - Graduate studies
   - Prefer not to answer
5. Please describe your health insurance coverage
   - I have no health insurance
   - I am covered through my parents’ health insurance
   - I have Medicaid, Child Health Plus, or another government subsidized insurance
   - I am covered through my employer’s health insurance
   - I am covered through an insurance that I purchased on the exchange
   - Other, please describe:
   - (free text for other)
   - Prefer not to answer
6. How worried are you that you might acquire HIV during your lifetime?
   - Not worried at all
   - A little worried
   - Moderately worried
   - Very worried
   - Prefer not to answer
7. What do you think is your current risk for HIV infection?
   - Extremely low risk
   - Low risk
   - Moderate risk
   - High risk
   - Extremely high risk
   - Don’t know/ not sure
   - Prefer not to answer
8. Have you considered, or have you made changes in your life recently in order to reduce your risk of HIV infection?
   - No, and I do not intend to in the next 6 months
   - No, but I intend to in the next 6 months
   - No, but I intend to in the next 30 days
   - Yes, I’ve been doing so, for less than 6 months
   - Yes, I’ve been doing so, for more than 6 months
   - Prefer not to answer
9. Throughout your **lifetime**, have your sexual partner(s) been
   - Men
   - Women
   - Both
   - Prefer not to answer
10. In your **lifetime**, how many **male partners** have you had **anal sex** with as either the top or the bottom (your partner’s penis in your anus or butt or your penis in your partner’s anus or butt?)

- 1
- 2
- 3-5
- 6-10
- more than 10
- Prefer not to answer

1. **In the last 6 months**, how many male partners have you had anal sex with, as either the top or the bottom?

- 1
- 2
- 3-5
- 6-10
- more than 10
- Prefer not to answer

1. **In the last 6 months**, how often have you used a condom when you have had anal sex with another man?
   - Never
   - Less than half the time
   - About half the time
   - More than half the time
   - Always
   - Prefer not to answer
2. **In the last 6 months**, how many **female partners** have you had **vaginal or anal sex** with?

Number of **female** partners in the last 6 months

- 1
- 2
- 3-5
- 6-10
- more than 10
- Prefer not to answer

1. Have you had group or party sex (sex with more than 1 other person at the same time) **in the last 6 months**?
   - Yes
   - No
   - Prefer not to answer
2. Have you used online/phone apps or dating sites to meet any of your partners **in the last 6 months**?
   - Yes
   - No
   - Prefer not to answer
3. Please check any drugs you have used **in the last 6 months**
   - Marijuana
   - Heroin
   - Meth
   - Cocaine or Crack Cocaine
   - Ecstasy
   - Spike/Spice
   - K
   - Molly
   - None
   - Prefer not to answer
4. How often do you drink more than 6 alcoholic drinks in one setting?
   - Daily or almost daily
   - Weekly or once every few weeks
   - Monthly
   - Less than once a month
   - Never
   - Prefer not to answer
5. **In the last 6 months**, did anything happen that you wish hadn’t happened due to your drinking or drug use?
   - Yes
   - No
   - Prefer not to answer
6. **In your lifetime,** how many times have you used PEP (***post*-exposure prophylaxis**) to prevent HIV?
   - Once
   - Twice
   - More than 2 times
   - I don’t know what PEP is
   - Prefer not to answer
7. **In your lifetime**, have you ever been treated for any of the following sexually transmitted infections? Please check all that apply.
   - Chlamydia
   - Genital or anal herpes
   - Gonorrhea
   - HPV or genital warts
   - Syphilis
   - NGU (non-gonococcal urethritis)
   - I have never been treated for a sexually transmitted infection
   - Prefer not to answer
8. When was the last time you were treated for a sexually transmitted infection?
   - In the last month
   - Between 1-6 months ago
   - Between 6 months to a year ago
   - More than a year ago
   - I have never been treated for a sexually transmitted infection
   - Prefer not to answer
9. What would you consider to be your knowledge level about PrEP prior to participating in this study?
   - Little knowledge (I had heard of it but didn’t know much about it)
   - Some knowledge (I knew something about what it is and how to use it)
   - Good knowledge (I knew a substantial amount about what it is and how to use it)
   - Prefer not to answer
10. How likely do you feel it is that you would be able to access PrEP if you wanted to?
    - Highly likely
    - Moderately likely
    - Unlikely
    - Impossible
    - Don’t know
    - Prefer not to answer

*[Participants should be shown and asked to answer any questions they didn’t complete]*

*[Thank you! You have reached the end of the research. If you would like to receive compensation for your time, please enter an email address below to which you have access and can collect your gift card. Gift cards will be sent within 4 weeks of survey completion. This is because surveys must be checked for validity.]*

- *Click here if you would like to have your data entered without an email address. If you choose this option you will not receive any reimbursement.*

*Please enter your email address: __________________*

*Please re-enter your email address:________________*

*(only works if they are matched)*

Appendix 2:

Further information on the DCE methods

Specification details. The chosen specification affords analysts additional advantages in terms of control over the type of distribution of marginal willingness to pay (mWTP) for each choice attribute.^1,2^ We chose this specification in order to obtain estimated coefficients that can be readily interpreted as individual mWTP for changes in one unit of the attributes, or $w_{kn}$. From the random utility model represented in specification (1), mWTP values are typically computed post-estimation from the ratio:

$w_{kn}=-\frac{\beta_{kn}}{\beta_{\$n}}$ , (A1)

where $\beta_{\$n}$denotes the individual cost coefficient. So, we denote the cost attribute of each alternative as $c_{itn}$ and adopt a version of this utility rescaled by $\lambda_{n}=\frac{\beta_{\$n}}{\kappa_{n}}>0$, where $\kappa_{n}$is the error scale factor set to 1, to express marginal effects of utility in the “marginal willingness to pay (mWTP) space”:

$U_{itn}={\lambda_{n}(w}_{n}^{'}x_{itn}-c_{itn})+\varepsilon_{itn}=V_{itn}+\varepsilon_{itn}$ (A2)

The econometric issue we are addressing is that, in the specification of equation (2), there are estimated parameters with associated distributions in the numerator and denominator of this predictor of mWTP. The interaction of these distributions has been found to lead to a high probability of improbable values at some distance from the mean (26). In the approach taken in this analysis, we address this issue by estimating in mWTP space rather than preference space.

Note that heterogeneity in preferences across respondents implies that the *k* individual utility coefficients in the vector ${[w}_{n}, \lambda_{n}]$ vary according to some random rule, which we assume ${[w}_{n},\ln{(\lambda}_{n})]$ is distributed in the population according to a multivariate normal distribution, with a *k*-sized vector of means $\mu$ and an unrestricted *k*$\times$ *k* variance-covariance matrix $\Omega$. The estimation task is therefore that of using the observed *i* $\times$ *t*$\times$ *n* indicators of stated choices $y_{itn}$ along with the *k* descriptors (attributes) of alternatives $x_{itn}$with identical dimension to estimate $\mu$ and $\Omega$.

Assuming an independent and identical Gumbel distribution for $\epsilon_{itn}$facilitates the task as it delivers a logit closed form of the choice probabilities, when conditional on $\beta_{n}$:

${Pr}_{itn}=\frac{\exp(V_{itn})}{\sum_{j=1}^{J} \exp(V_{jtn})}$ (A3)

Naturally though, each sequence of *T* choices by the same respondent *n* will be executed with a random draw of $\beta_{n}$so that the unconditional probability needs to be computed by integrating out such variability, which in our case is assumed to be generated by multivariate normal $\varphi(\mu,\Omega)$:

${Pr}_{n,t\to T}=\int_{\beta_{n}} \prod_{t=1}^{T} {Pr}_{itn}\varphi(\mu,\Omega)d\beta_{n}$ (A4)

In estimation, this is achieved by using a Bayesian estimator as described in Train and Weeks^3^ and implemented by a purpose-written code in R and sampling with Stan^4^. In this application, one crucial advantage of Bayesian estimation versus the more commonly employed estimation based on simulated maximum likelihood is that it delivers the unrestricted variance-covariance matrix $\Omega$ at no additional computational cost (in terms of time). Convergence was evaluated using the Shiny-Stan package, and robustness of key results was also evaluated with results from Stata with a diagonal $\Omega$ matrix (e.g. zero covariances). A second advantage is that it delivers the posterior distributions for each respondent and each random parameter, thereby enabling researchers to evaluate all features of these distributions, including quantiles, and not only the central moments. In our analysis, we contrast mWTP for one baseline characteristic of that attribute in contrast to an alternative characteristic. For example, the attribute of PrEP timeliness (i.e., how long after an initial evaluation one would have to wait before starting PrEP) has as a baseline option of immediate and alternative options of one week or four weeks. In this case, our prior expectation is that there will be a negative impact on mWTP as timeliness decreases. Table 2 lists all baseline attributes and alternative options.

Critically, note that out-of-pocket costs from $0-$200 USD per month were always included as part of the packages that participants could select to allow us to estimate in mWTP space. Selecting a package with a particular dollar amount attached indicated a quantifiable willingness to pay for the remaining attributes of the selected package. A particularly attractive attribute of access to PrEP, therefore, would be expected to induce selection of a package even with a higher overall package cost.

1. Thiene M, Scarpa R. Deriving and Testing Efficient Estimates of WTP Distributions in Destination Choice Models. *Environ Resour Econ*. 2009;44(379). doi:https://doi.org/10.1007/s10640-009-9291-7

2. Scarpa R, Thiene M, Train K. Utility in Willingness to Pay Space: A Tool to Address Confounding Random Scale Effects in Destination Choice to the Alps. *Am J Agric Econ*. 2008;90(4):994-1010. doi:https://doi.org/10.1111/j.1467-8276.2008.01155.x

3. Train K, Weeks M. Discrete Choice Models in Preference Space and Willingness-to-Pay Space. In: Scarpa R, Alberini A, eds. *Applications of Simulation Methods in Environmental and Resource Economics. The Economics of Non-Market Goods and Resources.* Springer, Dordrecht; 2005. doi:https://doi.org/10.1007/1-4020-3684-1_1

4. Carpenter B, Gelman A, Hoffman M, et al. Stan: A Probabilistic Programming Language. *J Stat Softw*. 2017;76(1).

Appendix 3.

| **Table 1. Characteristics of 18 in-depth interview participants in Upstate New York, aged 18-30** | | | | | |
| --- | --- | --- | --- | --- | --- |
|  | median | IQR | n | % |  |
| **Age** | 20 | 19-22 | **Insurance type** |  |  |
| **Male sex partners in the last 6 months** | 4 | 2-6 | None | 1 | 6% |
|  | n | % | Parental | 10 | 56% |
| **Race (check all that apply)** |  |  | Medicaid | 3 | 17% |
| Asian | 1 | 6% | Employer | 1 | 6% |
| Black | 7 | 39% | Exchange | 0 | 0% |
| Native American | 1 | 6% | Student health plan | 3 | 17% |
| White | 10 | 56% | **Employed** |  |  |
| Other | 3 | 17% | full-time | 1 | 6% |
| **Hispanic ethnicity** |  |  | part-time | 7 | 39% |
| Yes | 5 | 28% | not employed | 10 | 56% |
| No | 13 | 72% |  |  |  |
| **Current student** |  |  |  |  |  |
| full-time | 15 | 83% |  |  |  |
| part-time | 2 | 11% |  |  |  |
| no | 1 | 6% |  |  |  |
