# Supplementary material for: Perceived access to PrEP as a critical step in engagement: A qualitative analysis and discrete choice experiment among young men who have sex with men
Source: PLoS One. 2022 Jan 26;17(1):e0258530. doi: 10.1371/journal.pone.0258530 (PMC8791519; doi:10.1371/journal.pone.0258530)
Supplement: S4 File — (DOCX) [file pone.0258530.s004.docx]

Interview IDA-201

**So can you tell me some of those things that sort of you've learned about prevention...?**

So first of all... safe sex, using a condom. You, I think that's generally the biggest thing I've heard and I think... you know, like, if somebody is has I think open needles or is... you know, if there's any way I can have, so you know if somebody has a big cut on their body, I generally try my best to not have direct contact with that. Also I think just knowing if someone, you know, what they think. So for example, when you're talking to someone on Grindr and they tell you about meeting up and stuff, if they are even concerned of, you know, if they ask you your HIV status, if they, you know, want to ask you, so should I bring condoms or lube or not, if that conversation ever comes up, I think those are metrics in which I understand that this person cares about their health and is being safe. Also you know, reusing condoms, all these things. PrEP is something that I've heard very recently. I'm not on PrEP. I think I told you that on the phone. So that's another thing I've heard that a lot of people use as another sort of additional layer of protection. Am I missing anything? I hope not. These are the things I know to protect yourself from AIDS.

**That's great, that's great. And you said you sort of learned most of this it sounds like from some TV shows you mentioned watching and...**

So a couple of things is that, I don't know if I told you my major, but.

**You said...**

Bio-medical engineer.

**Engineer.**

Right, right. So, so I had to learn about a lot of the ways in which bio attacks happen on the body. So a lot of this was also covered in coursework, so, yeah. So I guess the combination of academia, pop culture, friends, videos, things like that.

**And so I just, I know we've sort talked a little bit about condom use, but I just want to ask some specific questions about that. So... how often... well how often do you use condoms now? I know that you're in a relationship.**

So here's the thing. Now I don't use anymore. Even when I was dating my current boyfriend, I used to for I think 8 months. But now I don't use condoms anymore. If you're talking about my previous sexual experiences, I have almost always used condoms. Like literally always, except two instances when I didn't. And those were people who I knew and was like, so they were not people in the community but people who went here. And you know, I was so scared after those experiences. I like almost in a week got tested and then made sure I wasn't, I made sure of my status. Except those two experiences, I've never had unsafe sex. And now, I do have unsafe sex. But, my boyfriend and I are also monogamous. So I guess, yeah.

**Do you mind telling me sort of about those two times? How that came to be?**

Yeah, I mean, I think what ended up happening is we were both extremely drunk and... we were all at another friend of mine's place and then the two of us ended up going back to his place. And we were both extremely drunk and neither of us had condoms on us. He did, though, so when I say extremely drunk, that's not necessarily I guess fair. I think it's more like tipsy, but not like, when I say extremely drunk, people might think that I was sort of incapacitated. I wasn't. I just had a lot to drink. But, so, when I say drunk to the point of not being able to go get condoms from the store. But still, you know, asking and having valid consent. So I consented to having sex. But I knew he would not be able to get condoms and I still consented. So, so that was one. The other one, I was actually at a debate competition. And this one was very risky. I had sex with someone who was on a different debate team of a different university but I knew him a lot. But yeah, just had it there. Yeah, I regret both of those times. I like really strongly regret it because I, you know, no matter what, I don't know where someone has been and what their status is. Even without knowing that, I had unsafe sex, so I was not happy about it. But you know...

**You said you were really worried after both of those times. And so you went and got tested**

I got tested... I mean, the first thing I did was I texted them to ask them, hey I know we had unsafe protect- unsafe sex last night, can you please tell me your HIV status or can you share that with me? Both of those people immediately said I am negative. But I, you know, it's just their word versus actually knowing. So that was relieving, you know, that they told me that. But I also verified clinically.

**And so I just wanted to double check also. You said that usually it was for oral sex you wouldn't use condoms.**

Never. I've never used condoms for oral sex. Does anyone really? I will bet 8 out of 10 don't use. I don't know. There's no way to know.

**Okay. And so I wanted to ask some questions also sort of about sort of the worry you were mentioning. You were saying you were pretty worried about being infected. Have you ever worried about any other sort of sexually transmitted infections or...?**

Yeah, I did. One person that I had, that I was with for a good time, had contracted chlamydia. And they let me know that. They con- they were positive for chlamydia. I never was. But, I was really sort of, we didn't have sex in those times. But, but yeah that was the only other time I was concerned for another sexually transmitted disease. But generally when I get tested, and I do that every year, I get tested for a reason, just to be sure.

**Maybe this is different because you're now in a relationship. I mean, are you still sort of worried about contracting HIV at any point in the future? Is that still a big worry of yours?**

No. The reason no, because I really trust this person. And you know, the only, the only way I would have any STD is if he does, I'm assuming. And so... I guess no. But you know, you trust people. People break your trust, right. They do. And then you here these stories and stuff, but like, people do. So, no, not in the foreseeable future. I have, yeah.

**So what.. so on a scale of 0 to 100, where 0 is the lowest possible chance and 100 is the highest possible chance, what would you say sort of is... what would you say is sort of your risk of getting HIV?**

Right now?

**At some point, at any point.**

Ever?

**Yeah.**

*Laughs nervously*. I don't know. What factors would you base this on? I guess factors you would base this on would be like you know, how many sexual partners do you have, how often do you have unprotected sex. I mean, how else could you get an STD. I don't know, if you do a blood transfusions or if you do... acid or something. I don't know. I don't know. Some people inject drugs. I don't do any of those. If you could help me, if there's any other factor that I'm missing.

**Well whatever factors you think are relevant, you know.**

*Laughs* Can I google this?

**Actually there might be some sort of website that'll give you a number of 0 to 100. What.. do you just have a rough guess?**

I don't wanna say zero because like I don't know. My boyfriend could cheat, could have HIV, and could infect me. I guess. I don't know. But I do think that's slim to know, so I will say like 5.

**So it's small.**

Yes. But I think. *Laughs*

**Okay, so the next set of questions I have are just about PrEP. I know you mentioned PrEP already, so.. so you can sort of tell me just what have you heard about PrEP?**

I've met a lot of people who are on PrEP. So, the way I understand it, it is... something you have to take, like you have you need to have recurring appointments. So you know, you meet the doctor or something. Like people tell me I have a PrEP appointment on this day and stuff. And as far as I understand you can’t contract or it's *snaps fingers* drastically minimizes your chances of contracting HIV, even if you are exposed to it. So I, the way I've heard of it, people look at it as an additional layer of protection against HIV. So that's what I know. I also have heard that it's expensive. And I.. am I missing anything else? That's all. It's like a pill.

**Yeah. And so, to where have you sort of heard some of that information?**

So, Grindr now has a section in which it says HIV status and you can say negative, on PrEP. So you can see that. So first of all, that's there. And secondly, I think there's just a, I think a lot of the LGBT pro like LGBT health advocacy, rights advocacy groups in [Upstate NY City 2], so there's a couple of organizations, [Health Center Upstate City 2] Health, I don't know if you've heard of them.

**I have, yeah.**

So there's always, and I follow them on Facebook, so you can see a lot of advertisement, advocacy for PrEP. So I think those would be my sources of information, hearsay among people and those groups that I guess have educated me about, and then after knowing, oh there's something called PrEP. And I like to know what's it, what is this going on, so I like googled and I found out and so it tells me. And google easily tells you what it is and a couple of stuff related to it.

**So you've done a little bit of digging on your own?**

Personal research, yeah.

**Is it anything you've, I mean aside from the social media you were just describing, [Health Center Upstate City 2] health on Facebook, is it anything you've sort seen represented or discussed in media other than that?**

This is media, like social media.

**Right, I mean other than that, any thing else?**

For PrEP, no. I've not heard it anywhere.

**And so you said you have, you do know some people who are-**

On PrEP. I've met with people. I've also hooked up people who are on PrEP. Yeah.

**Do you have conversations with them about PrEP at all or...?**

You know, the people who have who are all on PrEP have always come across to me as like very on top of their health and stuff. I didn't ask too much. I guess I... I didn't want to necessarily inquire. And if you remember, I was always, whenever I hooked up with people, I was always trying to not build a relationship with them, except this person, right? So, that's why I never bothered. I was like, I'll just do this digging by myself. Yeah.

**So have you ever, have you ever thought about taking PrEP?**

I have, but I decided against it.

**Can you tell me why?**

The reason I decided against it is because so first of all, I am not sure if I can pay for it because I, you know, at the completion of my university status, I don't have university health insurance anymore. Regardless of whether or not the university would cover it, I don't have it anymore, so I can't. So I'm having to wait for my employment and then after that get employment health insurance and then let's see from there. So that was a big factor. Second, I think, is that I... I don't know if... I necessarily will need it since I am pretty sure of being in a monogamous relationship. So I guess, you know, if I was still hooking up, I think would have, not that I would definitely have it, but I think the consideration would be at least tilted in the favor. But, but because I don't see an imminent need and I'm not sure how to pay for it, it seems like... not a reason for me to take it.

**Okay, okay. So I just wanna go through, and some of these you've already brought up, but I just wanna go through some of the sort of factors that others have said are sort of important to them when they're sort of making decisions about whether to take PrEP or not. You'd said that you'd heard that it's expensive. Can you sort of tell me about that? Where you heard that? And sort of what, if you got a sense of maybe how much it would cost?**

I don't remember, but I remember when I googled it it was like a decent amount. And I don't remember it. But I did... see that, you know, I don't have health insurance, so why bother. Yeah.

**Have you ever heard about anyone sort of struggling to pay for PrEP?**

No.

**Okay. Do you know... ... okay, so you'd mentioned sort of insurance. So you know that it sort, insurance would cover it, correct? And that was...**

Right, and so I think what I'm saying is that I guess I didn't care because I don't have it, so why bother knowing. But yes, if I, once I have an employment based health insurance, I would like to look if they covered PrEP. But because I didn't have it, I just didn’t bother even looking at it.

**Do you, I mean, do you know of any other sort of ways to pay for it? Programs that might assist, anything like that? Aside from insurance?**

No.

**And how about access to PrEP? Would you know where to go to get PrEP if you wanted to begin taking it?**

I am so sure [Health Center Upstate City 2] will give me access because they advertise it all the time. So yes, that's where I think I could get PrEP from. I assume similar organizations wherever in the world they exist would be able to give me PrEP?

**And going there, you think, do you think it would be sort of easy to get?**

Uhm... yeah? So many people have it, so.

**And so what about taking PrEP? Sort of what it's like to take PrEP, to be on PrEP? Have you heard sort of much about those experiences?**

It seems like it's annoying, right? 'Cause you have to take a pill. Is it everyday? I think it's everyday, if I'm not wrong. So I think you have to take it pretty recurringly and also you have to meet with the doctor. So these are... not convenient for people. So yeah. That's all I know. I don't know if there are additional inconveniences. But these two seem to be fairly inconvenient.

**Have you heard anything sort, did you mention it's a pill?**

Yeah, that's what I was told. Oh my god, if I'm wrong!

**No, no, no! I'm sort just trying to get a sense of what you-**

Okay, I was told, I really, really, really remember somebody telling me that it's a pill. Please tell me it's a pill.

**I'm not trying to like grill you. I'm just trying to get a sense of what you've heard and what sort of knowledge base you feel like you have.**

Right, yeah. I did hear it is a pill. You have to take it very frequently, not sure exactly what's the frequency, and that whoever I've met and has been on PrEP had like a doctor's appointment sometime in the future. So.

**So I mean, with those sort of inconveniences, do you feel like if at some point you take PrEP that's the sort of, would those inconveniences be manageable for you?**

No. It's annoying, right? It has to be worth it. Why would I knowingly inconvenience myself? Not that I'm saying had I not been with a monogamous relationship that I wouldn't have done it, but I'm saying these are factors. All of this matters. I'm also not too... happy with the culture that is going on with people on PrEP and telling others. But that's a different story than my own personal experience.

**Can you tell me about that though?**

Okay, so here's the problem. So, the way I understand it, you have do a lot of these things, right. You have to take it frequently. You have to go to these appointments. I think my concern in the LGBT community is that a lot of people could say that, and they do, right, it says on your profile that you're on PrEP, and I think that gives a sense of confidence, sense of assurance to a prospective person who's trying to sleep with them, that even if they have HIV, I won't contract it. It will, it does protect both ways, right? Like if somebody has PrEP, are they, so for example, if you have PrEP and you're sleeping with somebody, that person would still have an additional layer of protection, assurance that they may not get it from you.

**You mean, does it help prevent like you-**

Delivering to somebody?

**I-**

You don't know. Okay, so anyway, I think the culture that it does perpetuate is that this, if somebody has PrEP, they are, they are not that high risk because they, you know, they're so on top of their shit. They have an additional layer of clinical protection to be able stop giving HIV. But the problem is, I don't know if this person is actually taking these pills everyday or every now and then, actually going to these appointments. And I think it creates a false perception to the person who they will be prospectively sleeping with. I have a problem with that because otherwise, I think, if that person would have necessitated safe sex, they may be like, you know what, he's on PrEP, let's just now do it without a condom. And in my opinion, that's a problem. I also don't know if some people should be trusted if just they say that they are on PrEP. I don't know if that word has any meaning, right? Like I could put it on my profile, could I not? Now you could say that about, oh well you don't know if anybody has it, if anybody says they're HIV negative, you don't know they're negative. They could be lying. But, if I always necessitate using condoms, then I'm using the most, you know, it's 99 percent of a 100 percent assurance, but I'm still using it. But I think a lot of people may be dissuaded from using condoms because they assume that just because the person they're sleeping with is on PrEP that they are literally the epitome of being on top of their health. That's my concern. Because... I can see plenty of people who are very new to the gay community and who like, let's say come to college and they're 18 and they have a lot of sex, that they may have, they, you know, they maybe like, they have another reason to doubt the using of condoms and that's my concern. So that's what I have. But that isn't a concern on myself, right? That is a concern I have just with the culture of PrEP. Like this one like, this one word you use to create a perception to somebody else that they can never get HIV from you. And if you're deceived, then you're really, really screwed. So, yeah.

**Have you sort of heard anything about people or do you know of someone who sort of, like you were saying, you could just add that line to your-**

No, I don't. I have, you know, you could always believe people are in good faith. But it's not just adding that line. Somebody could, they might be telling the truth they're on PrEP, but you don't know if they're taking it everyday. You don't know if they're going to these appointments, right? So they may, it may not be an outright lie, but there may be inconsistencies in them having to follow up with all of these things. And I don't know, correct me if I'm wrong, but you have to do that all the time, right?

**That you have to go to the like three months, I think it's every three months.**

Those appointments. And take it everyday? Right? So, how do I know? I'm sorry. Can I... I find it's suspicious sometimes that someone might not have kept it. I mean, I don't know if not keeping it once takes it away from you. I don't know what the protocol is. But I think that is a concern. I just think it requires a level of diligence and you know consistency that, sure, people in good faith will do but I think, you know... why do we even ask people to use condoms, right? People could just ask, hey are you HIV negative? If they say negative, they just don't have to use condoms, right? But we still say people should use it, just to be safe. It's all about health and safety. If it is about health and safety, now you're giving another reason for people who, you know because for a lot of people, and I'm sure you can corroborate with any other subjects you have or other people you've con- talked with, condom use isn't convenient. You have to put it on. And for a lot of people it hurts. Or it's not as amazing, right? A lot of people say that. And now you're giving people an additional reason to doubt it. I don't think that's safe for college freshman or people who are vulnerable or just entering the community and are not necessarily equipped with a lot of this information. It's not necessarily a concern about the PrEP or the discovery of the pill, but a culture that we are perpetuating about this noble user of PrEP who is just angel in disguise and is just solving all your problems is my concern.

**Okay, I see. Do you think that, do you think that if there were sort of fewer inconveniences in taking PrEP, that if you didn't have to take it daily, you didn't have to go to doctor every three months-**

Right.

**Do you feel like you might be a little more trusting?**

Then I would feel a lot more confident. If it is that you take it once and then in three months, it's good, then I, okay fine. People are like to take it three in three months. But... like, for all your college classes and your PhD classes, did you always turn in every assignment on time and get a hundred? No, you didn't, right? Like people screw up. You know? I worry. Because in all my years, when I was sleeping all, who, everybody, I always tried to necessitate safe sex whenever, whenever the option came. I hope that culture doesn't go away. Yeah.

**I thought it was really interesting to that you sort of mentioned your image of like the PrEP taker or the PrEP user as sort of the noble angel?**

Yes, yes, and this know-it-all, be-all, which is like look, I, you know, if someone is taking PrEP and is doing all of these things and being on top of their health and being very safe, that is, honestly it is noble. I don't know if everybody deserves that noble label just because they have that option on their thing. I don't think people are knowingly trying to mislead people or lie to them. But I'm saying that the culture of growing use of PrEP and what it means as an image for someone or as a perception to someone who hears of someone is using PrEP expands the room of doubt for guaranteed measures of safe sex, which is using a condom. I do see a lot of occurrences, , not like I see it like I've seen it, but I feel like it is fair for me to predict a lot of the occurrences in which people are already doubting or dissuading getting, dissuading from condoms to do it more. Or I also see people having what I think is perverse interpersonal dynamics, to tell you that, like come on, I'm using PrEP., I'm on PrEP, do we really have to do this? Do we really have to? I feel a lot of people getting not bullied but sort of pressured into not using condoms because they'll tell you I'm using PrEP, why? Why are we using condoms? It's so much inconvenient. Or it's we gotta go to the store. It's just that they'll ultimately be like okay, yeah, yeah, fine. But you don't know all of these things. You don't know if they're doing all these things and being precautious. So, that's my concern.

**So do you think... I'm wondering sort of where that image of the noble PrEP user sort of comes from. Is that sort of something, just an impression that you've developed on your own or is that something you've seen somewhere?**

So I think... it's sort of comes from, you know, the, I guess, specific language and... how do I say it? The language and the vibes that things carry it in wherever I've seen the portrayal of PrEP, right? So for example, if you google PrEP, you know, like, if you look at most of the advertisements for PrEP, you know, you should take PrEP, you should protect yourself. You should, you know, all the, it's a good thing to do, right? Like, so, anybody who hears of somebody using PrEP would associate that positivity of that image to that person because they are taking care of their health. They are taking protection. So I think it comes from any part- like why would anybody to take PrEP if it wasn't something good? But my problem isn't using PrEP. My problem is getting away with this one word that has a lot of responsibility attached to it. I guess I don't know if... if that's a fair way of looking at it. So it's not that I think that every somebody who takes PrEP is always extremely noble. And you know, that was more like me being provocative. But I think that, you know, it is advertised in a way that it is something good to do. It's something healthy. And it is a... it is a thoughtful precaution that one can undertake to protect themselves against HIV. Yeah.

**So... So I know we said it's sort of a pill. Do you think that any of the other sort of modes would be more sort of appealing or attractive to you?**

As opposed to like an ointment?

**Like an injection?**

*Gasps*

**Or like an implant?**

*Gasps* No, no, no.

**Pill is definitely better.**

Among the three, is definitely better. Injection, are you crazy? No. *laughs* I don't want more needles in my body. And also every day? What are you saying? Would you be okay with taking an injection every day?

**I don't think I would.**

Right! How would you do it? Like by yourself? Are you even capable of that?

**You could do that, I guess.**

Oh god.

**You know, people who are, say, diabetic have to.**

Right, like okay so, I mean, obviously, if you have to, then you have to. But to me, somebody who is diabetic has to take precautions because they have no way out of it. As opposed to you're choosing to do this voluntarily on yourself? Why would you? So, no. I do think pill among the three is least inconvenient and therefor the most preferable.

**Okay, just checking, okay. And how about effectiveness? I mean, have you, what have you heard about-**

I've heard good things about, I think, and also from reputable websites like the CDC, maybe? Yeah. CDC and other websites corroborating the peer reviewed academic research that has been done to verify this. So I don't doubt the effectiveness of PrEP IF it is used with the diligence and consistency that requires it. And so, and you know I defer to experts, but you have these kind of things. I'm not the kind of person who thinks global warming is a hoax, so. Hopefully you're in the same basket. *Laughs*

**No, I am. So, so how about side effects? What, if anything, have you heard about the side effects?**

I don't know.

**No? You haven't heard anything?**

I assume that, you know, most doctors should be to, you should be able to talk with them. So, you know, obviously, if I have any known allergies, sorry, if whatever I'm allergic to, if you know, a doctor should be able to give me that sort of consultance and like tell me, nope it's fine. But, I, you know, anything in the sp- any form of medical procedure or medication has side effects. There's nothing that doesn't have any side effect. But, you know, I'm assuming that since it's in the market place and the CDC recommends it, that those side effects are minimal and they're so rare or not that grave. But you know, I don't know what they are, if that's what you're asking.

**So I just sort of wanted to ask a little bit about, and we sort of talk around this some, the possibility of there being a sort of a stigma attached to PrEP.**

I think the opposite.

**Yeah, it sounds like maybe you see the opposite.**

I really don't think stigma, no.

**Do you think that, you know, if you've had conversations with friends about PrEP, did they seem to share your view too, that it's...**

I haven't talked to too many people about PrEP. I know one person. See, here's the thing. I think when I hear somebody has PrEP, is on PrEP, it, for me, is reassuring. It, you now, tells me that this is somebody who is on top of their shit. Stigma, I guess there could be one way people associate stigma with it because if you are on PrEP, means that you have additional protection, means that you probably have a lot of sex, and they're okay with and comfortable having a lot of sex. So I think that could be a stigma. But I am somebody who doesn't associate being, you know, sexually promiscuous as something bad, so I don't care. But I think the people who associate it with stigma would be the same kind of people who associate sexually promiscuous as being worthy of stigma. So I... words like slut and promiscuous doesn't carry any baggage with me. You know, someone who's on Grindr will probably be meeting other people, so I don't care if you're having a lot of sex with everybody. I do care about your health and your ability, and my health that comes associated with it. So, yeah, you know, I guess I think that most people I know think of it as either positive or neutral. I don't think anybody has necessarily negative stigma associated with it. And the people who do, as I said because they just don't like people who are slutty, then those are the people who I'm not too fond of. And I think they, you know, I think then the stigma isn't really coming from PrEP. It's coming from being sexually promiscuous, which is different than taking a medication. So, and I don't think anybody can solve that. Like how are you going to stop being people judged of being slutty, from being that. So I think that's, I guess, the more deeper reason as to why there's stigma associated with it, if there is. But I don't think there is.

**So if you decided to being using PrEP at some point, would you, would you feel comfortable telling people, your sexual partners, that you're using PrEP? Would you be reluctant to do that?**

So, correct me if I'm wrong, but the whole point of it is to, you know, know that you can't pass or HIV or your ability to pass HIV is significantly reduced, even if you had it or it's contracting it from somebody. Why would I not want someone to know that? It.. I don't find a reason, why would I not tell anybody? In fact, I will probably tell them because they will probably be like more likely to be assured that they can't have, that they can't get HIV from me. I don't know. I have no reason not to tell them. Also if I'm doing so much work and being so diligent, yeah I'm gonna tell them. But I don't know.

**This might be obvious, given some of what you've said already. How would you sort of react if someone that you were with had tells you that they were using PrEP?**

[01:37:29.05] R: Yeah, so, I did hookup with people who did. It was always reassuring. I still used condoms regardless. Yeah.

**It was reassuring.**

It was reassuring to me because, you know, I guess I'm the more... naive gullible optimistic people you will see. So you know if someone tells me, I take them at their word. So it means that, you know, they're taking more precautions, which is great for me. It means I'm less likely to get any diseases they may, HIV, that they may have. But I would use a condom anyway. Yeah.

**Okay, so, okay so, we're getting towards the end here. I wanted to give you this sort of fact sheet about PrEP.**

Uhoh. This is gonna disprove, like here's why you're wrong. It is an injection that you actually take. You know nothing about PrEP.

**Just take a look over this and then we'll just talk about it kind of quickly.**

***Reading over fact sheet***

See, that's my concern. Condoms are still a highly effective way to prevent HIV. I hope everybody knows that still. I really do.

***Reading over fact sheet***

Yeah, good luck to all these different options, like injections or infusions. Scared the hell out of me. What do you mean an implant under your skin? Like a?

**[01:40:34.14] I: Like the some of the birth control implants that people can take that you'll put under like in your arm and it lasts for-**

That's like slightly more desirable. Only slightly.

***Reading over fact sheet***

Cool. Wonderful.

**So... I don't know that I'll necessarily be able to answer them, but so do you have any sort of new questions or comments that come to mind, having just read this?**

So I told you my biggest concern about the culture of PrEP. You're somebody doing a study on this. You have presumably some knowledge and research and have like exposed yourself to this. Respond please. *Laughs* Like what do you, how can I... I am, you know, somebody who cares about this community and I am concerned. Does my argument seem totally like nonsense to you after, and you can be honest with me.

**Your argument about?**

Regarding the fact that this culture of PrEP and individuals who may already be dissuaded from condoms, but now will have to because they know that that's the only way of assuring. But as opposed to they're now with a partner who has PrEP and they'd be like, okay, this person has PrEP, let's just not use the condom. Or being pressured not to use a condom by that person who's using PrEP.

Is it, is it okay if we, if we, we're getting near the end. Is it okay if we sort of finish up my questions for you and then we can?

Oh, sure. I thought you asked me to ask you questions.

**Yeah. If you just have them. Like, if they're, not like about PrEP. Like about.**

Oh, okay, I'm so sorry, okay. It's fine.

**Okay. You're okay. Does any of this sort of change the way you think about PrEP at all?**

Does it does it does it? Some of the wording of the side effects are a little alarmist and inflammatory, didn't need to be that. But, but it's fine.

**What specifically felt alarmist and inflammatory to you?**

See. About 1 in 3000 people could have a kidney problem. 1 in 10 people, 1 in 10 people is fine. But I think 1 in 3000 people could have a kidney problem, I think if sometimes if you are able to say that point oh oh oh three percent, people realize that that's the fraction you're talking about people. And I think for a lot of people, numbers or like quantifiable things stay in their head in the back of their mind. So just that. It just seems like one person could get it, as opposed to really rarely. And I also think that... I also think that, for example, when you're saying... so far, so at the end of the first paragraph, so far, with many thousands of people taking PrEP worldwide, there has been only one person known to have gotten HIV while taking PrEP everyday. That person was infected with a very rare strain of drug resistant. I know what you're trying to convey at. But I think, you know, at the end of the day, you know, with many thousands of people taking it, if you can be a little more specific and tell me that, so if it is what, five thousand people and one person got it, it's literally zero point, that many zeros percent of people. One person got it. And that's enough. I think it seems to be unnecessarily defensive. It's fine if that person's a rare whatever, like, the simple fact that it's such a low number is a good thing. And, you know, I wish in terms, specially for people who are, I guess today's millennials or whatever, I guess it's we like to see things that are as least verbose and more like, here is, here is your risk. It's zero point zero zero zero four percent from anybody who's taken it to have HIV. This percentage of people have a kidney problem and that's it. As opposed to, you know, this is just a rare, a resistant form of HIV, I think could help. I think this is great. But I think that could become even better. I guess it's less of questions and more of suggestions to make it even more conducive to people.

**Thank you. Do you, do you feel like if, do you feel like more.. How many. Do you feel like this information is sort of widely understood in the LGBT community? Or?**

So, so, to sort of double check with me, I think I knew most of it. Like, you know, I.. so I think one piece of information that I did get that I didn't have is this part, for people who don't have insurance, there is a program. That's really cool. But I didn't know that. But other than that, it seems like I have followed this. I will say that, you know, just, did you ask for suggestions? I'm sorry. What?

**Those are great. Anything's great. Just anything in response.**

So I think maybe just restructuring this, in terms of overview and then side effects, miscellan- you know ho generally things are? I think people like compartmentalize things. It's just easier. I think that could be better, but yeah, I think this is fine. Yeah.

**So do you feel like this is informa- it seems like you knew most of this. Do you think most people?**

I do think this is informative. Do most people know this? I'm sure my boyfriend has no clue about this. So sure. Well they know what PrEP is, but not all of this. Maybe most people don't. Am I more informed that general people? I don't know. I don't know. As I said, the reason I dug all of those is because I met people who are on PrEP and I saw in social media, so I wanted to know more because you know, it's in the LGBT community. So what's going on? I like to know. So, I don't know. I think it's fifty fifty. I think every one in two people may not know of this to every person who does know of this. So I think this is great. The information is very succinct. I think if I were just revising this, I would just restructure it in like sub groups because it's a lot of information. So for me, this is fine, as a research subject. But if you were looking to have it as a promotional material, that might be more suited to. But yeah, those are very minor suggestions.

**And do you think that this the information here would.. Do you think it would change people's sort of likelihood to take PrEP or not? Or affect their decisions?**

It really depends upon what why they were taking it to begin with. You know? For someone who, so for example, this part really like still highly. I'm sorry I took this. This daily medication with three months. Do you really? I just have. Maybe this is me just not being optimistic for once. But that requires a lot of diligence. I don't know if I could trust the average person with this much diligence. But, I don't think this has any more information than for dissuading someone. I think, I think, you know, there are some amazing benefits. There are a lot of people in the LGBT community who are, you know, who have sex a lot. And this could be an additional layer of protection for them. This is true. For people with, who are HIV positive, they can take it and not be worried about passing it to other people. That is also true. These are concrete benefits that PrEP has. You know, I don't think anything that's starkingly reason why people won't take them. Yeah.

**Okay, well, okay so. Thanks again.**

Yeah. No worries.

**For everything. For sharing so much. I really appreciate it. There's just two last little exercises I want to do that are really brief and then there's a quick self administered questionnaire I'll give to you that takes I think five minutes at most. Okay, so the first thing that I want to do involves these index cards that have some of the factors that we've talked about around taking PrEP, its cost, how easy it is access, its effectiveness, its side effects, stigma, how you can take it, the mode. We talked about the pills, injections, and how often you have to take it. First of all, do you think there are any other factors that are really sort of important in shaping your decision to.. to take PrEP or not?**

To take PrEP? Whether or not you're in a monogamous relationship. I think that is one.

**Okay, okay. If you want to write those down. So just-**

So in a? Are you in a? Is that for?

**Sure, sure.**

***Writing on index cards***

**Okay. So is there anything else?**

I guess, are you sexually promiscuous because somebody who was sexually promiscuous would always want to make sure that they are being safe, so they are more like to take PrEP. That's what I think.

**And any others? No? No. Okay. So I want to give these to you, so you can look through them. And I'm gonna ask you to rank them for yourself, in terms of what is absolutely most important and what's least important.**

I have a question. So for example, yes, I know that cost is important. But I also know this, so I want to rank it low. Is that fine? Or is this not knowing anything, what would you?

**This is for sort of you, right now, your circumstances, and so, just yeah. Just put the most important factors at the top and the least important at the bottom.**

I mean, obviously, if something's not effective, why am I gonna take it? Like, if it doesn't do anything?

**So this is most important for you?**

I think. I may like shuffle things around.

**: Sure, absolutely.**

I think for someone, co- like this is hard. 'Cause cost, like how much? Like if it was like let's say 20 dollars, even if it was cost, I like a lot of people would take it then, I feel like I would take it. So I don't know what the. But with this, so when you say covered, it's zero, fully covered?

**What was the?**

Is covered by almost almost all insurances policies.

**Yeah. Right.**

So it's zero. So it's free.

**There might be copays. I'm not sure for like doctor's visits or yeah. It probably depends on your insurance plan, that sort of thing. But for you, you know what I mean, where you are.**

So at right now, I don't have an insurance plan. As I told you, I graduated. And it says there is a special program. I haven't taken it, so I'm just going to assume that there's probably some copay and they're probably not like deadly. Yeah. Ease of access, I don't know. [Health Center Upstate City 2] and everywhere, it's there. Side effects. I trust medical organizations that are reputable and trusted. I don't think there's too much stigma. How I can take it. I know it's a pill so it's fine. Wait. Rank in order of how important it is?

**Right, how important each of these factors are to you.**

So because it's a pill, I don't care. It's gonna be hard to rank these bottom ones. How often you have to take. Are you in a monogamous relationship. Are you sexually promiscuous. Like honestly, I, if I don't, if it's not effective and if I'm not sexually promiscuous and in a monogamous relationship, then I won't take it at all. I think like these are like prerequisites. It has to be okay to these, then I'll even look at these. So.

**Oh, I see. I see. So these are like almost separate. Like if you're. Is that right?**

Right.

**I see. Yeah.**

These are like, if this thing that I'm gonna take, go bother to take daily and go to doctors isn't even effective, I'm not saying it isn't effective. It seems that only person has proven it's to be uneffective, so it is very effective. So that's why it matters to me so much. So it's gotta be effective. I have to be sexually promiscuous. Otherwise, if I am only sleeping with two people every year and they're definitely. I think the number of people definitely increases the likelihood that you know I may contract HIV so I should be more precautious. So I think it matters. And this is basically like the flip side of this. Maybe. So, these are equal. So this is number two, this is one. Okay so, so after all of these are okayed, then I'm gonna look at this. After all of this. Because I'm thinking. How often might not be that bad... So I think there are almost no side effects that it make it distinguishly bad. So I'm gonna put this at the bottom. I think ease of access if also, you know, fine because I know so many places where I could get it. I, how I can take it, because it's a pill, it's not that terrible. I don't think that there's that much stigma actually. I think there's probably less stigma here, here, here. And reasonably assuming, I can get this covered, but there's probably gonna be copays. It's probably not gonna be that terrible. Copays are what? Depending on insurance plan. I'll put it second and then I'll put this first. So this is my sort of, this being top, this being sh- on tie of second place. Three, four, five, six, seven, eight.

**Okay, okay. So at the top is effectiveness. That's the most important thing for you. It has to be effective.**

Yes.

**Okay. And then there's sort of these questions about-**

Sexual history.

**Right. Okay. Are you in a monogamous relationship? Are you sexually promiscuous? Then how often you have to take it. Then cost. Then how you can take it. Then ease of access. Then stigma. Then side effects.**

Yes.

**Is that right?**

My, my basic sort of justification for this is that if isn't effective and I don't, if my sexual history doesn't warrant it, then I'm not even gonna bother to look at this.

**Right. So if it doesn't pass these tops, what you're-**

Yeah. Then it's a no. Right. But once those are granted, I think I care least about the side effects because they're so low. I don't care too much on the stigma because it seems to me that it is a good thing to do for myself and I also think it is a positive way of protecting yourself. So I don't think it's something bad or negative or whatever. I also think that ease of access comes third last for that reason because it's very readily available, at least here in [Upstate NY City 2], since you told me to look at myself and my experience. It's a pill, so that's fine. It could be costly, but because it's covered, it's probably free and there may be some copayments, which I'm willing to bite, since it's effective, as I need it. But I think the number of times is just too much. It's just annoying. Yeah. But that's how I.

**Great. Okay. Thank you. So I'm gonna collect these. And then the next exercise that I want to do, take a look at this. So imagine, imagine that I'm gonna flip a coin, right. And so, if heads comes up, you obviously get what's in the heads row or column. If tails comes up, you get what's in the tails column. So if you could only pick one of these, which one would you pick?**

What do you mean? What are these? I don't-

**So, so these are dollar amounts that you would get. Imagine I'm gonna flip a coin and if comes up heads and you choose row 1, you get 50 dollars. If it comes up tails, you get 50 dollars. But the other end, if you choose row 4 and it comes up heads, you get 500 dollars. But if it comes up tails, you get 0. So of these four. Does that make sense?**

Yeah.

**Okay. So of these four rows, if you were, if we were gonna do that. We're not.**

Okay. *Laughs* I think you're gonna give me 500 dollars. Which one would I do?

**Yeah. Which one would you pick?**

I'm willing to take the risk of getting 30 dollars if it includes the chance of hundred.

**Okay, so you'd take row 2. Heads a hundred dollars, tails 30 dollars.**

Yeah.

**Okay. Great. Okay. So, that's the last, that's the last exercise. I really, again, I know I said it before, but I really appreciate you answering these questions.**

Yeah, I really, I hope I didn't bore you at several times, like why is he speaking for so long?

**No this was absolutely fascinating and very helpful. The last thing that I have for you is just this self administered questionnaire. It usually takes 5 minutes. Take as long as you need obviously, but it shouldn't take terribly long.**

***Filling out the questionnaire***

*Laughs slightly* Okay, you should really have South Asian as different.

**Sure. You can and you can, yeah.**

Yeah.

***Filling out the questionnaire***

Oh, I made a mistake. So this is definitely crossed out.

**Okay. Sure.**

***Filling out the questionnaire***

What do I do here if I've not given any of them?

**Oh, then that's, you don't have to check any.**

I'll just write none.

**Yup. That's great.**

***Filling out the questionnaire***

So here's the thing. I, I've never had chlamydia, but do you remember the time when I told you that my partner at the time contracted chlamydia from someone else? So I took the medication. So I have been treated for it, but I never had it. So is that okay if I?

**Yeah, yup.**

Okay. Well, vaccination doesn't mean treatment, right? Yeah.

**Right.**

I mean, I have HPV vaccine. But that doesn't count for treatment. It only asks for treatment.

**Right. Yeah. You're right. Right.**

**Okay, that's it. Okay, so thank you very much. So the consent form is yours to keep. And just as a reminder, there's the numbers on the back with the that you can contact if you have any questions and you want to talk to someone who's sort of outside the study. The PrEP fact sheet is yours.**

Which numbers on the back?

**Right here. Under the subject advocates. Okay so then we have to make sure you get compensated. So 40 dollars for your time. And then how about travel?**

I told you 8 dollars.

**So it was 8? Okay. Great. So let's see. So there's 5, 6, 7, and 8.**

Thank you.

**Yeah, of course. Thank you. So, if I could just have you then fill this out. So there you go. 48 dollars. Just the top line.**

So I just write my name and si-

**Yeah. Yeah, yeah.**

So I, how many subjects do you need for this study?

**We, I think we're gonna try to do at least 30 interviews. But it's actually part of a larger project. The first part's qualitative interviews and then there's a, we're gonna use some of the data from that to develop, they're discrete choice experiments, to sort of try to get a sense, you know, the things that are important to people when they're making decisions about whether to take PrEP or not and under what circumstances they'll take PrEP. That's why. Okay, so it's okay if I turn this off?**

Yeah.

**Okay.**

Interview IDA-202

**And how about prevention?**

Uhm you know. Condoms are very very effective. But you know, there is the one in a hundred chance that it'll break, which is I guess where PrEP comes in. And yeah.

**And I know you were saying a moment ago that you know, you feel like it's not a death sentence anymore. Can you talk a little bit about what treatment options are available?**

I know that there is medication that can essentially make it undetectable. You know. I know that you still, of course, you don't want it because, you know, you will be taking medication the rest of your life. And you know, you still, you're not gonna be a super healthy person. It's certainly a lot better than people used to be. Yeah but no it's amazing there's a lot of treatment out there available.

**And so you'd said that you'd mostly learned about HIV and AIDS from your research sort of on your own?**

Yeah. Yeah.

**Okay. Are there any other sort of sources of information? I know you said you'd talked to some people.**

Yeah. I mean I have at time had specific questions that I would ask Planned Parenthood. But yeah.

**So, so you mentioned a moment ago that you always use condoms?**

Yes.

**Okay. Okay. So there aren't any circumstances in which you wouldn't?**

Well, well I always use them for intercourse. But not necessarily oral sex.

**And that doesn't depend on your relationship with the partner?**

No. Yeah, no, it doesn't matter how well I know them. I always use condoms.

**Okay, okay. So has there ever been a time when you thought you might be infected with HIV AIDS?**

One time I had sex with someone and the condom did break and that was pretty scary. I didn't really think, you know, it was likely. But I was still concerned.

**So, yeah, I mean can you tell me about that? I mean that probably is a scary thing.**

Yeah, no. It was just, it was this person that I didn't really know that well. I knew he was very sexually active. So I was like, well it's not impossible. And yeah I got the post exposure prophylaxis thing. And then yeah, it was, it was really just. I think it was a little bit just me being paranoid and me not being very experienced. This was the first person I'd ever had sex with. So, yeah. I think if that happened now, it would probably be with someone that I'd had talked to more first and talked to more, you know, if they get tested, et cetera. So I don't think I would be in the same situation nowadays.

**And you said that was the first person you'd had sex with?**

Yeah. Also the brand of condom we used, they're prone to breaking. I mean, now I only use trojans for that.

**What brand was that?**

It was the like lifestyles ones. The ones they give out for free. I've heard they break all the time. So, that's not, yeah.

**Okay. Have you, have you ever had any other, have you ever had a sexually transmitted infection?**

The herpes virus, yes.

**Can you tell me about that?**

Basically just got it through oral sex. And yeah. I don't know. I mean it was definitely unpleasant. But I don't know. The more research I did, the more I found out you know there are many people who get it because just yeah. It's so common and it's not like you can prevent it with protection. The only way you can prevent it is not having sex. And you know, that's not gonna happen. So, yeah, I don't know. I definitely came to terms with it.

**So, so was that pretty early in your sexual experiences or more recent?**

No, that was, I'd already been having sex for like over a year.

**And has that changed the way you think about or practice sex or?**

I think it just made me a tad more careful and a little more understanding. You know. If someone were to tell me oh I have herpes, I wouldn't automatically be like well, you know, you're dirty or anything like that. I'd just be like well you know, it happens.

**Was that sort of your, sorry, sorry, was that sort of your impression before hand?**

Not necessarily that they were like, you know, that they slept around too much or anything. But I remember thinking if anyone has anything, I'm not gonna go anywhere near them. And now I've kind of been like well, you know, herpes is very very different from HIV. It's not like you can group all STDS into one thing.

**So I guess I'm wondering if that was, you know, if you kinda felt that way, how did, how did you feel when you were diagnosed?**

It was definitely concerning. I remember at first thinking that you know that no one would want to be intimate with me again. And yeah I don't know. Just through talking with people cause I had a friend that actually got it not too long later and felt comfortable enough telling me and yeah, I don't know. It was definitely a journey.

**So is this something you tell sexual partners about or have told anyone about?**

I've told one partner just cause he told me he had it too. But basically, you know, I was told, you know, it's not that easily spread unless you're in the middle of an outbreak and you know, it's something that so many people have anyway. So no, I can't say I tell my partners usually.

**Okay. So thinking about HIV again, are you at all worried that you that you might contract HIV at some point in the future? Is that something you worry about?**

I can't say I do because I'm a pretty safe person. I do use condoms. And I don't. I generally don't sleep with people I don't know that well. Not that someone I know couldn't have HIV, but I feel like it's less likely than a complete stranger. And in general, I've just never been a very sexually active person. You know, I haven't slept with that many people. So I can't say I'm worried about that.

**So, there's a question we ask everyone. On a scale of 0 to 100, where 100 is like the highest chance, where would you sort of put your chance of being infected at some point in the future?**

I'd say like 58 or something?

**58?**

Yeah. You know. You know, I am homosexual, but I don't know. I am safe. You know. And I do generally not sleep with a ton of strangers. So yeah, no, I'd say I'm pretty confident in my ability to protect myself.

**Okay, okay. Okay. I'm wondering also about how you sort of think about sort of yourself in comparison to other gay people. Is it.. do you feel like you have fewer sexual partners than most?**

I don't know about. Yeah I think I'd say fewer than most. Though not to the extent at which I originally thought. You know, originally I thought everyone was always having sex. And then yeah, the more I got to know people, the more I found out it was more not really, but I'd still say I have less than most, I think. A lot of, a lot of homosexuals are very promiscuous. That's just the truth. I think that's just how men are, really. And, when you take women out of the equation, it's just a lot more direct. So, yeah.

**So how do you, so how do you feel about that? About others being so promiscuous?**

I I don't know. I think you know, really whatever works for someone. But I think if someone is gonna be promiscuous, they should definitely get tested regularly. And they should definitely use condoms. Which a lot of people don't, you know. But I think there's a safe way to be promiscuous. Though I think there is an extent to which where, you know, even if you're safe, if you're sleeping with that many people, you're still putting yourself at risk. But that'd have to be quite a bit, I'd say.

**So is that, is that part of the reason why you have, you feel like you've had relatively few sex partners? Do you sort of worry about others being promiscuous?**

Yeah, I can't say I trust a total stranger. Just cause so many people just don't use condoms for some reason. And yeah, no I'd say that's the main reason I'm not very sexually active is to just yeah to protect myself from STDS.

**So that is that main reason?**

Yeah, I'd say that. Also you know, I do like sex better when I know someone. But I'd say that's the main reason.

**So why do you think so few people use condoms?**

I don't know. I think there's this idea that it's, that it interrupts, you know, the process. And I think a lot of people think it feels better without a condom. Though I don't think that's true. I've heard from numerous people that it's really not that different. Just, there's this idea that it's better. Yeah. I think. Yeah.

**Have you ever had sex without a condom?**

Nope. Not once.

**Okay. You'd just heard that it's-**

Yeah, yeah. No, I have friends who have. And they say, really, physically, it's not that different. It's probably more like seems closer and more sexual.

**Can I ask also, I know we've talked a little bit about some of, you know, friends that you talk to and learn through them. So are your friends, do they and the people you talk to, do they tend to use condoms also?**

Yeah. Yeah. No. My friends generally are pretty safe. I do know some that if they're in a relationship with someone, you know, that's not necessary. But I, I don't know. I don't really feel that way.

**Okay. Okay. So do you feel like maybe using or not using condoms is stigmatized? Would you say? One way or the other?**

Mmmm no. I think our generation whether you do or don't use them, people don't really judge you. I think if you tell someone that never uses condoms, well I do, I don't think they'd be like well you know, I don't agree with that. I think they'd be like well it's your choice. Same the other way around. I think if someone was like I never use a condom, maybe like some people would be like that's not very safe. But most people would be like well I'm not gonna sleep with you but you do you. So yeah, no, I wouldn't say it's stigmatized.

**And how about for you personally. If someone tells you they don't use condoms at all, how do you think about that?**

I defintely kind of rule them out as a sexual partner. I do have a partner that hasn't used in the past. And I basically told him, well if you get tested then you know, I will sleep with you. But we are using a condom, which was a level of risk fine with me. You know. I think. I mean, it's pretty safe. But in general, like if I meet someone and I don't know them yet and they tell me I don't use a condom, like well, you know, sorry.

**Do you, do you sort of look negatively on people who don't use condoms?**

A little. It does seem irresponsible to me. But I think if they're honest with me about, I do respect that. You know. I think the thing I respect the least is if someone didn't tell me. Or lied about it.

**Okay, okay. So the next set of questions are about PrEP. And you'd already referenced PrEP, so I take it you're at least a little bit familiar with PrEP?**

Yeah.

**Okay, so, so, so for starters, can you maybe tell me what you've heard about PrEP?**

Basically it is a medication that's been used to treat HIV for a long time and it's being used a preventative. It's extremely effective. It's like 97 percent or something if you take it regularly. Yeah. I don't know.

**Okay. Do you.. so how have you learned about PrEP?**

I don't remember where I first heard about it. But I remember it wasn't that long ago and I was kind of amazed. But, yeah, no, I've definitely gotten information from Planned Parenthood and stuff. And also from just research online.

**Okay. Are there other organizations beside Planned Parenthood?**

The county has an office, an STD clinic, and they have information about it.

**Okay. And what are some of the, are there sort of trusted sources online for you? Go to websites?**

I think anything from the CDC is pretty, you know, trustworthy. Yeah I don't know. You just have to play it by ear.

**So, it's kind of interesting. You'd said like when you first heard about it, it was kind of amazing. So what was amazing about it?**

It's essentially a vaccine for HIV. I know it's not perfect. But it's pretty close to perfect and I just think it's amazing we have, you know, all these ways to prevent it. And I think they should really be implented, you know. I know the governor said, you know, we could eradicate AIDS by 2020 and I totally agree with that. And I think further down the line, we could you know essentialy, you know, mostly eradicate HIV. We could make it a very rare disease. And you know, that's really, you know, just if we implement these types of things.

**Okay. Do you personally know anyone who's taking PrEP?**

Yes. I know one person who just got on it.

**Okay. Can yiou tell me about your relationship with them and any conversations you've had about it?**

Basically I know he doesn't always use a condom and truthfully I think he's just taking it so he never has to use one. And yeah I don't know. I know he just started it. He found out that his insurance covers it. You know. And yeah.

**Okay. Does he seem to be having a positive experience thus far?**

Yeah, I know he's gotten headaches and stuff. But he says it's not that bad.

**Okay. Okay. So have you considered taking PrEP yourself?**

Yeah. No, I looked into with the county. The only thing is it would be under my parents insurance and even with HIPAA and all that, I don't really want to run the risk of, you know, an awkward conversation. But yeah, no, someday when I'm on my own insurance, whenever that'll be, I would like to.

**Okay. So, so is it just an awkward conversation you're worried about or?**

Yeah. I don't wanna essentially say, you know, I'm being promiscuous so I'd like to take this thousand dollar medication. It's just an awkward conversation.

**But your parents, you'd said you'd come out to your parents?**

Sort of. You know, I mean they know. It's just not something we talk about. But yeah.

**Okay. Can you, can you maybe, if we just go back to that for a minute, can you just tell me about when you came out to them? Was it like a just a conversation?**

No, well I guess so, yeah, with each individually. But it wasn't something that I like brought up. It just kinda came up. And yeah. No. It's not something we really talk about.

**How did it just come up?**

I don't know. It just sort of did.

**Okay. And so.. so you just don't want to, is that right? It sounds like maybe you just don't want to have like another conversation like that?**

Yeah. It's just, it's a very direct conversation. It's saying, you know, I am having sex with men. I don't want to get HIV. I would like to get on this medication. It's just, it's yeah, I don't know. It's too much too soon, you know.

**What do you mean by that? It's too much too soon?**

It's just, it's a very intimate conversation and I don't think I have that level of communication with my parents really. So it's just a lot. Yeah no I'm sure they would say like yeah go for it. It'd just be very awkward.

**Okay. But you do think they would be supportive?**

Yeah. Yeah, no. My parents are inherently logical people, you know. I mean that's why they've never, you know, like really criticized me for being gay. You know, they're, they know, they're like, well you know, it's not like he chose to be and it's not that big of a deal, you know. They're just, they're older. They're from a different generation. But, yeah, no and I'm sure with this too, they would be like well, logically, you know. You know.

**So you don't worry they would like judge you or think badly about you?**

No. I don't think that's it. It's more just a very uncomfortable thing. My insurance is also kinda finicky with very expensive medications, so there's that.

**Okay. Okay, so the, this last set of questions really are just about some of the factors that other people who've considered taking PrEP have said are kind of important to them when they're making that decision. So I just want to run through these and see how you think about them and what you've heard about them.**

Mhm.

**So how about the cost of PrEP?**

Like what do I think is the cost?

**Yeah. Have you heard much about it or what's your sense?**

I think it's like a like a little over a grand. Something like that. But there's a lot of assistance programs out there, at least in New York. Yeah.

**And I know you said your insurance is a little finicky. Can you tell me about that?**

They would cover it, I'm sure. But like, like I went to the emergency room and they sent a notice saying you know, you can't use your insurance this much or we're gonna cancel your policy. And I had allergy shots and it was the same thing. And it's just not like they'll deny a claim necessarily, though they have. It's more just, I don't know. It seems very complicated and I don't want to run the risk of losing my insurance for my whole family. So better not.

**So that's part of it too for you is that you feel like there's a -**

Yeah I don't know to what extent they would cover it and all that. It's just not a great time.

**Have you heard about anyone sort of struggling to pay for PrEP?**

No.

**No. Okay. Okay. Can you tell me about some of the programs you'd said you'd heard about?**

Yeah. No. They did look into them for me to see if I was eligible, which I already knew I wasn't. Well I have insurance. But I know, Giliad, the manufacturer of the drug is willing to pay for it for people that are uninsured and even if you do have insurance, they're willing to pay the copay. And it's all part of the governor's plan to eradicate AIDS. So just right now is a really good time to get on PrEP, you know. Maybe not for me, but for a lot of people.

**Okay. How do you feel about that? The fact that it seems like such a good time for everyone else but not you?**

Disappointed, but it's not really the end of the world. You know. Condoms are still very effective. Knowing your partner is still effective. I don't feel like I need PrEP to have sex. It just it would be definitely, it would make me feel a lot more secure.

**So you would like to be on it, it's just not a good time.**

Yes.

**Okay. And how about access? It sounds like maybe if, if you wanted to, and you do, you would know where to go?**

Yeah, I would go through the county. I know they set you up with someone to check your liver function, all that. And yeah then get you started.

**So is your sense that that's kind of an easy process? Or is that a little complicated?**

I think it's pretty easy. The hard part is figuring out how to pay for it exactly. And even that, it's really not that hard. I mean I'm sure my insurance would cover it. There's a good chance my parents would never even find out. It's just I don't wanna run the risk. I would, I could probably be on PrEP if I really, really wanted to. It just, it doesn't seem worth the risk. You know, I'm not, yeah.

**Because of that possibility for that awkward conversation.**

Yeah, that possibility and the possibility of my insurance getting cancelled. All of that. It's unlikely. But, you know, a risk is still a risk. And I don't absolutely need PrEP. So, you know.

**And what about taking PrEP? What have you heard about that? About how you take it?**

You just wanna take it around the same time every day. And it's a pill.

**Have you heard anything about doctors visits? Or?**

I believe. I know that when you start it, they check your liver function and all that. But after that, I'd imagine you'd go back in once in a while. But I really don't know.

**And how about some of your own preferences. Does taking it everyday, would that be a problem for you, do you think?**

No. I mean I take allergy meds everyday. It's not that different.

**And how about it being a pill. Would you sort of prefer it be an injection, an implant?**

I'd much rather prefer a pill. But I'm not super picky. That's the easiest.

**So even if you have to take it every day? Taking a pill every day is better than?**

Yeah, I'd say so.

**Okay. And I know we talked about this already, but I think you'd mentioned at a time [loud bang makes the next word unable to be heard]. What have you heard about the effectiveness of PrEP?**

Very effective, like 97 percent or something. Yeah. And really, I have, not through a doctor, through the grape vine, I've heard of people like getting screwed over or like people that stop taking it, suddenly they're even more susceptible to HIV. And I've heard of people that think that a new stronger form of HIV's gonna form. But I think all of these are just rumors. I have brought up concerns to doctors and they all say well we've never heard that. I think. I mean, it's too early to tell of course, it's still a new medication. But I don't think there's any downsides to Truvada biologically. I think the only downside is a lot of people take it and then stop using condoms, so other STDS are spreading like wildfire, which isn't as bad as an HIV epidemic but things like HepC, you still don't want them.

**So can you tell me a little bit more about some of, you'd said that you'd heard about people getting screwed over. Can you tell me a little more about that?**

Yeah I've just heard of people that were on it and still got HIV. I think, I think a lot of that was probably people messed up taking cause I know if, I know it's a pretty forgiving medicine. If you miss a day, you're probably still fine, though you should probably start a month over. But, you know, I think, I think those cases were probably someone messed up their medication or something like that. So I don't think that's much of a concern. Also if you use PrEP and a condom, I think your odds of getting it are pretty much zero, you know. Like I, just yeah, it just doesn't seem like it would happen.

**So those stories you've heard, you don't, it sounds like maybe your skeptical of them.**

Yeah I think people are always very suspicious of new medications, not that Truvada's new, but it's new as a preventative. But yeah, I think people are just kind of ridiculous.

**So it hasn't, hearing those things hasn't like affected the way you think about PrEP or your own decision making?**

I mean, I'd be lying if it didn't make me a little more cautious. But I think, it's still, I'd brought up these concerns to my doctor and he was like well I don't, I've never heard of that. I think PrEP does seem a little too good to be true. But only time will tell. But for now, I think, I think as long as you're not solely depending on PrEP to not get HIV, you know, as long you pair it with other safe sex methods, I think it's a great kind of back up.

**And what do you mean by too good to be true?**

Like a medication that you just take everyday and you are immune to HIV and no side effects sounds a little too good to be true.

**Okay. Yeah, so can you tell me a little bit about side effects? Have you heard anything at all? I know you mentioned someone said he had headaches.**

Yeah. I know some people get headaches when they first start it. Some people get nauseous when they first start it. Other than that, I'm sure it does take a toll of your liver. They wouldn't test your liver first for nothing. Though that might take years and years. Yeah I don't know. I don't know of any other side effects. But I feel like a medication that strong, there'd be something. Maybe not something terribly serious, but I doubt you just take it for years and you're fine, you know. I'm sure it has something.

**So what for you personally, what are some of the side effects that would be most concerning, that you just wouldn't?**

Liver damage would definitely be a concern. You know, if that is a thing. But other than that, if it was something like mild fatigue or something, I'd be like, well not ideal but worth it, I'd say. But yeah.

**So headaches, or that sort of thing.**

Yeah. Like the headaches to begin with, just suck it up. They'll go away.

**I'm kind of interested also in stigma. Some people have mentioned stigma as a sort of factor that affects their decision making. Do you think people look favorably or badly on people who take PrEP?**

I think a lot of people when they see someone takes PrEP, they assume they don't use a condom. But like I said earlier, no one really seems to care about that, so, no I wouldn't say there's a stigma.

**So you don't look positively or negatively yourself?**

I'd say I look positively because I mean it means they are taking some sort of measure to be safer. I know I would be a lot more willing to have sex with someone if I knew they were on PrEP.

**If you were on PrEP, would you be reluctant at all to tell a sexual partner?**

No. No I wouldn't.

**Okay. So, so we're getting near the end of the interview. One of the things I want to do is give you this fact sheet, which you can keep, if you're interested. If it's okay, would you mind just looking over that and then we'll talk about it a little bit.**

***Reading over fact sheet***

Oh wow. Well that explains something. That, yeah, I don't have any questions.

**Okay. So what did you learn?**

Only one person. There are strains of HIV that are drug resistant, so that is scary. But like you said, very rare. And yeah, the new forms of PrEP that they're working on. I didn't know any of that. Sounds promising.

**So it sounds like you kind of had a mixed reaction. Some scary things, some promising things.**

Just I, I thought there weren't different strains of HIV. That drug resistant one sounds terrifying. But, you know, there's all kinds of scary diseases out there. You just have to be safe and live you life.

**So do you think that your information will change the way you think about PrEP going forward?**

Mmm no, not really. I think, you know, it was overall just very good news. One person out of thousands and thousands. And yeah I didn't know they were working on different kinds of forms to take it, you know. I hope someday I'm able to take PrEP and who knows what form it'll be, so.

**And you said it's the sort of the daily that's probably sort of your preferred form?**

I'd say so. The implant under the skin sounds a little more convenient. But I'm pretty good at taking medications. Especially cause I feel like they would just make sure you don't run out. So, yeah, no, I really, I would rather not inject myself everyday. But that's the only one I'd say I wouldn't be a fan of.

**Okay. I'm also wondering. Now that you've read that, to what extent do you think other people in the gay community are sort of aware of that information?**

I think people know somewhat about PrEP. I think people know it doesn't protect against other STDS, it does protect against HIV. It's a pill you take everyday. Et cetera. You get headaches when it starts. Stuff like that. I think that's about the extent of people to which people know. And like I said, there are rumors floating around. But in general, I don't think most people believe them, you know. I think that's just I don't know. People love to find fault with everything. But yeah.

**Do you know anyone who sort of believes those rumors?**

Yeah. No, I mean I've heard them through friends, so of course they believe them.

**Okay. What's your reaction to that?**

I just think if you didn't hear it from reputable source, don't repeat it. Cause I mean rumors can be awful, especially regarding sexual health. I can't think of an example right now. But there are rumors that have made people do things that aren't safe, so I think, it's very irresponsible to repeat anything unless you heard it from a reputable source.

**Okay. So do you think if, so do you think if this information was sort of more widely known, that that would affect people's decisions about PrEP?**

Maybe. I think telling people that someday they're gonna have it in forms other than a pill, that would appeal to some people. But it's not like they would start taking it right now because of that. I'm sure some people are like oh I don't want to take a pill everyday, I'd rather just get HIV. Cause you know, there's people that think that way. But yeah, you know.

**Okay. So those are all of the my questions for the that I have for the for the the that first part of the interview that interview. So if you remember, I'd said that there are just a few sort of quick exercises that we'll do here at the end. The first one is a sort of a ranking exercise. So I have here some index cards that have on it, again, some of the factors that people have said are pretty important to them. So how often you have to take it, cost, how you take it, pill injection implant et cetera, effectiveness, ease of access, stigma, and side effects. So just looking at this list, is there anything that you think is missing? Any other factors that would be really important for you?**

No I think you got them all, really.

**Okay, so I'd like to ask you to rank them in terms of what is most important to you to what is least important.**

Okay.

***Moving index cards around***

**Okay. So tell me about this ranking. Why this ranking for you?**

Well the cost for starters, if you can't afford it, you can't afford it. That's not really even a choice really if it's that expensive. Effectiveness, you know, if it's not reasonably effective, it's like what's the point. And you know side effects, yeah. You wanna watch out what you're putting in your body. Easy of access. Et cetera. Stigma, there isn't a stigma. And even if there was, who cares. It's your body. It's not like you have to tell people. How often you have to take it, I mean, if it was like seven pills a day maybe then, but it's not, so. And how you can take it is not super important.

**Okay. So, so for you, the most important is cost. Then effectiveness. Then side effects. Then ease of access. Then how you can take it. Then how often you take it. Then stigma.**

Yup.

**Right. Okay. Great. Thank you. So the next thing that I want to do is go over this. So this is pretty unlike everything we've done so far. So, so just assume hypothetically I'm gonna flip a coin and you can pick one of these rows. And whichever row you pick, if it comes up heads, you'll get what's in the heads row, same with tails. Now we're not actually gonna do it. But if that were the case, which row do you think you would pick? You can just mark it.**

**Okay. So you picked row 2. Why would you pick row 2?**

I'm not a huge risk taker, but a little risk I think is worth it.

**Okay. Great. Okay. And then the very last thing that we'll do, if it's okay, I'm gonna have you fill out this questionnaire. Obviously your name doesn't go on it. And it usually only takes about 5 minutes. But then once you're done with that, you're all set.**

Oh cool.

***Filling out the questionnaire***

**All done? Okay. Thank you very much. This will go right in here. And so we just have to make sure you get paid too. So did you have any travel costs?**

No, I walked.

**You walked. Okay. So just the 40 dollars.**

Mhm.

**Okay. I just have to make sure you sign this.**

***Signing logbook***

**Okay, you signed that. I will get you your cash.**

***Getting the money***

Thank you.

**Can you just make sure to print your name? Sorry. Sorry.**

Oh, yeah.

**Okay, so we're all set. Thank you so much for your time and for talking with me. I know it's not easy always to share that kind of stuff so I really appreciate it.**

Yeah. Thank you.

**Yeah, sure.**

Have a good one.

**Thanks, you too. Take care. Oh, did you want to take any of these?**

Oh, yeah.

**Okay. There's also, I included there at the back, I forgot to mention at the beginning, it's just a list of some resources. You seem pretty knowledgeable about them anyway, but just in case.**

Cool. Thank you.

**Yup, take care.**

Interview IDA 203

S1 00:58:34.912 Okay. And how about prevention?

S2 00:58:40.271 Well, there's always condoms, which are not foolproof. There's PrEP and kind of a reaction to it. There's, I think it's PEP, where you could take, I think it's within 72 hours of the possible exposure.

S1 00:59:03.475 Okay. Okay. And so do you have a sense of what treatment options are like for people who are HIV positive?

S2 00:59:15.859 Expensive. Yeah, really expensive. They're not the way they used to be. You used to be basically taking meals full of pills, whereas now, I think that I read that it's four pills a day. Three in the morning, one at night, because you're taking a repeat. So you're not taking that many. I already take four pills a day. Two for thyroid and two for depression, so.

S1 00:59:45.314 Okay. So maybe it's not as burdensome as it used to be, yeah. Okay.

S2 00:59:52.125 I think you definitely have to stay on it. And these medications have also been able to prove to create undetectable levels of HIV and AIDS which basically, is, from what my doctor explained to me, means that there's almost a zero chance of you transmitting it to someone. It's as if you don't have it. You can live with it, but you still need to stay regimented.

S1 01:00:19.715 Okay. So you said a moment ago that you've sort of done some research. Can you tell me how you found out about HIV and AIDS? How you've learned about it?

S2 01:00:29.599 Health class, and in recent-- it's been my worry going through CDC statistics, and going through health plans and things like that. Talking to my doctor.

S1 01:00:47.663 Okay. Is it something you feel like you've learned about through media at all?

S2 01:00:57.542 I don't think the media does a really good job of explaining it. I think they're trying to scare people.

S1 01:01:02.391 Yeah. Okay. Can you talk a little bit about that? Where you've seen something that you think is just trying to scare people?

S2 01:01:11.995 Well, I mean, I'm very dated, but when Ryan White had his issue, they were portraying him as a monster. They were allowing him to be seen as someone who was not okay or normal, and yet it allowed parents who were in Indiana [inaudible] to really act like they could judge him. That's why I don't take the media's word on a lot of stuff like that.

S1 01:01:45.205 Okay. Is it something you see represented in the media much?

S2 01:01:47.581 No.

S1 01:01:48.139 Yeah, okay. Okay. I know we've mentioned condom use a little bit. Can you tell me how regularly you use condoms, if at all?

S2 01:02:02.654 The first six times I didn't. That's why I was so scared. But since my testing, it is now a requirement.

S1 01:02:13.451 Okay. Yeah. Okay. And so does it matter for you, your relationship to the person, or--?

S2 01:02:24.086 For me, at this point, to not use one, I would need-- we would need to have both gotten tested together. I would need to see the documentation from the health department or wherever that says, "You don't have an STD."

S1 01:02:39.710 Yeah. You wouldn't feel comfortable just accepting someone's word. You would want to see--

S2 01:02:43.716 I'd want to see-- I have documentation. When I got tested, I printed off the results. I have them in my room. It probably wouldn't do much for the mood, but if someone ever asked me, all I'd have to do is reach over in my desk, and I have my July 2017 STD records.

S1 01:03:03.302 Yeah. You ever had to do that?

S2 01:03:06.810 No.

S1 01:03:07.510 No? Okay. Okay. Are there particular kinds of sex that you would want to use condoms with but not others, or across the board?

S2 01:03:17.782 All across the board.

S1 01:03:19.048 Okay. Okay. So you said you do experience some anxiety about [inaudible]--

S2 01:03:28.829 Yeah.

S1 01:03:29.240 Yeah. Okay. How about any other kinds of sexually transmitted infections? Do you worry about those, too, or--?

S2 01:03:36.593 Mainly it's HIV, but that's because HIV is the one that really just can't go away. The other ones are treatable. I mean, gonorrhea. I know that there's an antibiotic-resistant gonorrhea going around, but it's nowhere near the United States. It's in Spain, Japan, and somewhere else in Europe, I think. But gonorrhea is a shot. You get a shot. I was talking to my doctor about that. For most of it, it's just antibiotic use. They don't have to be deadly if you get checked. So it's mainly just HIV.

S1 01:04:24.616 Yeah. So you just don't worry much about the others for those reasons?

S2 01:04:27.435 Because they're not long-lasting.

S1 01:04:29.828 Okay. And I know you said that most recent was negative. Have you ever had a sexually transmitted infection?

S2 01:04:37.147 No.

S1 01:04:37.742 No. Okay. Okay. So sort of on this question about your anxiety or worry, say on a scale of 0 to 100, okay? Where 0 is the lowest chance and 100 is the highest chance, what would you say is your risk of getting HIV in the future?

S2 01:05:04.120 What am I taking into account?

S1 01:05:07.080 Well, anything and everything really. Just--

S2 01:05:12.958 I would say below 50.

S1 01:05:14.502 Okay. Okay. Do you have a specific number?

S2 01:05:24.886 No.

S1 01:05:25.915 Just somewhere below 50?

S2 01:05:27.386 Yeah. Well, being gay and white puts me on a one in six chance. It puts me at roughly a 17% chance right there off the bat. So using those statistics that's about-- I would just say below 50.

S1 01:05:49.299 Okay. Okay. Okay. So the next set of questions are about PrEP and it sounds like you've heard about PrEP and you're interested in taking PrEP. So just for starters, can you tell me what you've heard about it?

S2 01:06:06.821 It's a preventative drug that is used to prevent HIV and AIDS. It's not fool-proof. It just lowers the risk of you being able to contract it from someone who does have it. It takes two weeks. Then they recommend that you wait two weeks for it to take full effect. I think it's Truvada. Is that the drug? I think so.

S1 01:06:36.865 Okay. So where have you heard about it? Where have you learned about it?

S2 01:06:43.041 In all honesty?

S1 01:06:44.082 Mm-hmm.

S2 01:06:45.367 How to Get Away with Murder.

S1 01:06:46.718 Okay. I haven't heard that. Yeah.

S2 01:06:50.345 Because there was a plot where the gay couple, one of them ends up with HIV and they want to stay together. They still want to maintain their sexual relationship so the one who does have it takes PrEP. No, not PrEP. The one who doesn't have it takes PrEP. The one who doesn't have it takes his medication until he gets down to-- until he's able to get it to undetectable so that they can have safer sex. And obviously, they still used a condom.

S1 01:07:26.212 Okay. So that was the first time you'd heard about PrEP?

S2 01:07:30.063 Yeah. That was the first time.

S1 01:07:30.465 Yeah? So what was your reaction to seeing that?

S2 01:07:33.085 I thought it was fake. I thought it was [inaudible] for TV but after doing research and when I went to the health clinic in Charlotte, I was told that I could get that. That I could get PrEP.

S1 01:07:49.331 Okay. So when you did the research after that, what are some places you got that information?

S2 01:07:56.298 Mecklenburg County Health Clinic.

S1 01:07:57.794 Oh, okay. Okay. And were they helpful?

S2 01:08:03.428 Yeah. The guy was helpful. He handed me a few brochures. He answered all my questions.

S1 01:08:08.339 Okay. Okay. Do you feel comfortable going to a health clinic?

S2 01:08:14.806 Mm-hmm.

S1 01:08:15.782 Yeah?

S2 01:08:15.745 They deal with people who've gone with a lot worse than me.

S1 01:08:21.508 Yeah. Okay. Do you personally know anyone who's taking PrEP?

S2 01:08:27.339 No.

S1 01:08:27.651 No? Okay. Okay. So I know you said you were considering it, can you tell me what some of the factors are in your thinking?

S2 01:08:43.802 Just if I could get it.

S1 01:08:45.288 That's [crosstalk]--

S2 01:08:45.689 Yeah. I want it. It's something I think is necessary.

S1 01:08:51.432 Okay. Okay. Okay. So now I just want to ask you about some of the factors that other folks have said are important and I just want to gauge, for you, sort of how important they are, what you know about some of these factors. So what, if anything, do you know about the cost of PrEP?

S2 01:09:14.872 In terms of its cost, I was told that with certain health care plans it's $25 a month for a co-pay.

S1 01:09:22.719 Okay. Okay. And is that the health care coverage that you have?

S2 01:09:28.084 Blue Cross Blue Shield, North Carolina.

S1 01:09:29.698 Okay. Okay. Does that seem feasible to you?

S2 01:09:36.992 Yeah. 25 bucks a month would seem feasible.

S1 01:09:40.502 Okay. And would you pay that? Would your family pay that?

S2 01:09:47.801 My mom has said that she's thought that she would pay that.

S1 01:09:50.374 Okay. Okay. So you wouldn't feel uncomfortable with your family knowing that you're taking PrEP?

S2 01:09:58.269 My mom and I have discussed this and while she was uncomfortable with the idea of me in that situation at all, she came to an understanding that it was better to be protected and safe rather than just not be, so.

S1 01:10:16.378 Can you tell me about that conversation you had with her? How did that go?

S2 01:10:19.035 It was uncomfortable. I told her that I wanted to make sure that I was protected. She said, "Well, then just don't have sex with people you don't know." And I said, "Well, that's really easy for someone who's engaged and not going to. But when you're in college, that does not always pan out, and I would rather just be protected."

S1 01:10:38.782 Excellent.

S2 01:10:38.895 That was my response, and we agreed that it would be good. But then my physician went-- she no longer my physician anymore. [inaudible] before I left. So it's not like I can just go in, rush in, and get a new one. So I didn't get it when I was in Charlotte. Plus, they wanted me to run tests, I think, on my liver. They said that that's what they wanted, that they would run tests on my liver before they prescribed me Truvada.

S1 01:11:10.138 Okay. And you didn't want to do that?

S2 01:11:12.548 No. It was a matter of just that would have taken more time.

S1 01:11:14.752 Oh, I see.

S2 01:11:15.051 We just didn't have time to go in, do tests, and then get prescription.

S1 01:11:19.989 Yeah. Okay. So you said your mom was initially resistant, but eventually came around?

S2 01:11:25.253 Not resistant. Just very like, "Why would you need this?" type thing, questioning it.

S1 01:11:32.331 Yeah. Yeah. Okay. Have you heard about-- I know you mentioned insurance. Have you heard about anyone sort of struggling to use insurance and getting insurance to cover it?

S2 01:11:45.846 No.

S1 01:11:46.520 No. Okay. Okay. Okay. So I know you said access was really the big thing for you. So what, if anything, have you heard about where you could go to get PrEP here?

S2 01:12:11.537 I haven't. I haven't heard yet. I plan on dropping by the health clinic maybe within the next week or two, because I'm not quite sure. Plus, I need to go in and get the rest of my HPV vaccines completed.

S1 01:12:23.946 Okay. So what have you heard about taking PrEP and what you have to do to take it and what it's like?

S2 01:12:35.161 You have to take it regularly. You can't just take it on and off. It's not like you pop it before sex. You need to be on it regularly, on a daily basis.

S1 01:12:45.597 So it's daily?

S2 01:12:46.523 Yeah.

S1 01:12:47.118 Okay. Do you know sort of what form it comes in? Liquid? Pill?

S2 01:12:51.491 Pill.

S1 01:12:52.219 Pill? Okay. Do you know anything about doctor's visits for folks who are on PrEP?

S2 01:12:58.356 No.

S1 01:12:58.987 No? Okay. So since you're planning to go in the next-- you said week or so?

S2 01:13:07.237 Yeah.

S1 01:13:07.752 I mean, do you think it'll be easy to get.

S2 01:13:11.668 I'm not quite sure. Just depends on the process of whether or not I can get it here at the clinic or if I have to go to a physician here in [Upstate NY City 1], but I have no clue about the process.

S1 01:13:24.692 Okay. And once you get it, do you feel like you'll be able to do the once-daily pills?

S2 01:13:31.294 Oh, yeah. Yeah. For something that important I would.

S1 01:13:34.382 Yeah. Okay. So that doesn't feel like too big of an inconvenience for you?

S2 01:13:39.053 No.

S1 01:13:39.637 No? Okay. I'm wondering about some of your preferences, too, in terms of how to take it. Would you prefer if it were say an injection over a daily pill?

S2 01:13:52.898 Depends on how often the injection was.

S1 01:13:54.726 Okay. I see. How do you feel about say an implant, something that would go under the skin and last for a longer period? Would you be interested in that?

S2 01:14:05.165 I would be interested in that.

S1 01:14:06.474 Okay. More so than a pill you think?

S2 01:14:07.959 Yeah. Because then it becomes easier to manage. You just go in and you get it done. I think that would be preferable to a pill. Something you don't have to worry about each day. You don't have to worry about when you go on vacation, did you pack the pills.

S1 01:14:24.204 Yeah. Okay. And what about the effectiveness of PrEP? Is that something you've heard anything about?

S2 01:14:31.491 I've heard that it was extremely successful. That it was able to prevent it in almost every case.

S1 01:14:38.480 Yeah. Okay. And I'm wondering how important that is for you to-- how effective would it need to be for you to be interested in taking it?

S2 01:14:52.684 Over 90%.

S1 01:14:54.203 So that's your threshold, is 90%?

S2 01:14:57.149 It would need to be so-- it would need to be rather minuscule number of unsuccessful cases. It couldn't be like a quarter of all cases. It would need to be less than 10%.

S1 01:15:09.141 Okay. Have you heard anything about side effects?

S2 01:15:16.614 No.

S1 01:15:17.535 No? Okay. What do you think, for you, are some side effects that you just couldn't live with?

S2 01:15:30.549 Not quite certain. I mean, this is my health. This is my DNA. I'm not sure that there's much that I wouldn't-- unless it's death and stroke. I'm not certain that there's much that I wouldn't live with.

S1 01:15:47.089 Okay. So you're willing to put up with quite a few side effects it seems.

S2 01:15:51.420 Mm-hmm.

S1 01:15:52.705 Yeah. Okay. I'm wondering if you think that there's a stigma around PrEP? Do you have a sense--?

S2 01:16:01.190 Oh, I don't. For the most part, from what I've seen is just people trying to be safe. I mean, there's as much a stigma around PrEP as there are people who use condoms. Just trying to stay safe.

S1 01:16:15.912 Yeah. Do you think other people think either positively or negatively of people who use PrEP?

S2 01:16:22.173 I haven't had that discussion with most people. Usually, it's just between me and my doctor.

S1 01:16:29.339 Okay, okay. So you don't really have a sense of how other people perceive it? So if you were taking PrEP, would you feel comfortable telling your sexual partners?

S2 01:16:46.394 Oh, yeah. I think I would feel obligated to. I feel obligated to let them know that I'm protecting myself.

S1 01:16:57.787 And if your partner was using PrEP, would you expect them to tell you too-- it seems.

S2 01:17:02.655 Yeah. I would be happy. I would be a lot happier if they were just making [me?] safer.

S1 01:17:08.086 Okay, okay. And you said you're 18, right?

S2 01:17:18.303 Yup.

S1 01:17:18.876 Okay, okay. Was this something-- PrEP that you'd started thinking about before your 18th birthday?

S2 01:17:27.947 Mm-hmm.

S1 01:17:28.543 Okay. So, at that point, where you sort of thinking at all about how your age might factor into getting it?

S2 01:17:34.081 Yeah. I was quite certain that I would not be-- that there would be people who would look at me differently because I was a minor on a sexual prevention. It'd be like any girl who was on birth control before she turns 18. Like, "Why do you need it? What makes you think you're going to need this before you turn 18?" And I just want to get prevention.

S1 01:18:01.813 Yeah. So before you were 18 did you think it would be hard to get access to PrEP?

S2 01:18:10.136 Mm-hmm.

S1 01:18:11.432 Yeah. Okay. Did you know anything at that point about whether you would need, say, permission from your parent or guardian?

S2 01:18:19.757 I knew that I would.

S1 01:18:20.423 You would? Okay, okay. And so when was it that you had that conversation with your mom?

S2 01:18:30.153 I didn't have the conversation until around June.

S1 01:18:33.356 Okay. So you were 18 at that point?

S2 01:18:35.942 No, I had a conversation.

S1 01:18:37.609 Yeah. Okay. Before that, did you worry about sort of whether your mom would be supportive? Like she would take you to doctor's appointment and that kind of thing?

S2 01:18:50.023 Not really. I think it would take her some time to get around to it but-- I mean, just to come around on the idea but I think she would still do it because she understands it's a health concern.

S1 01:18:59.239 Yeah. Okay, okay. So I want to do something a little different now. We're definitely nearing the end of the interview but this is a different part of the interview. And so what I want to do now is show you - this one here - this. So this is a fact sheet about PrEP, and this has some facts that might be helpful or interesting for you. You might know a lot of this. But why don't you just take a second and look over it, and then we'll sort of get your initial response to it. Okay?

S2 01:19:42.668 Okay.

[silence]

S2 01:21:48.306 I just want to [inaudible] my doctor. She didn't say anything about having to visit her, every three months.

S1 01:21:53.563 So that was new information for you?

S2 01:21:56.233 Yeah. Is it a requirement?

S1 01:21:59.193 Yes, I believe so. I believe so. So are there other sort of new questions or thoughts that come to mind, having now read this?

S2 01:22:13.584 No, I was pleased to hear that only one person has been infected with HIV to do this, to go through this. But what scares me is always the fact that there's a drug-resistant strain. That's why it happened because that means that there are some drug-resistant strains that could still kill people who are taking preventative and reactionary medication.

S1 01:22:35.261 Yeah. So that fact that it's drug-resistant--?

S2 01:22:38.348 Just the simple the fact of medicine that scares me.

S1 01:22:41.460 Yeah, okay. Any other questions, comments, responses? No? Okay. I wanted to ask also, to what extent do you think others sort of in the gay, bisexual, trans communities-- to what extent do you think they're aware of this information?

S2 01:23:01.107 I wouldn't to say they're that well aware.

S1 01:23:03.311 Yeah. What makes you say that?

S2 01:23:05.691 Simply because most of them who have-- the ones who are aware don't really need to be aware because they're not as sexually active. And the ones who need to be aware aren't aware because they're not worried about that. They didn't live through the HIV scare of the 1980s. So they don't understand to the extent that it could be.

S1 01:23:27.718 Yeah, okay. And do you think if there was an increased awareness of these facts that it would change the way people think about or choose to take PrEP?

S2 01:23:38.510 I think you'd definitely see a larger increase in usage.

S1 01:23:41.641 Okay, okay. Okay. Thank you so much for talking with me. That's the last part of the open-ended interview. I just two really quick little exercises that we'll do. It will only take a few minutes and then there's just a really quick closed-ended survey I'm going to ask you to take. And then we'll be all done.

S2 01:24:02.409 Okay. I'm just wondering. I read on there that there's payment for this. How do I get paid?

S1 01:24:08.818 I will give you cash once we're done. So you'll walk out with it today.

S2 01:24:13.346 Awesome, sounds good to me.

S1 01:24:14.796 Okay. So the first exercise is an exercise in which I'd like to ask you to rank some of the factors that we were just discussing that people tend to consider when they're deciding whether to take PrEP. Here are the ones that I have listed. Side effects, effectiveness, ease of access, cost, stigma, how you can take it, the form, how often you have to take it, so just the frequency. And okay, and that's it. But I have these others-- these are the blank cards that we can use, do you think there are other factors that are important to you?

S2 01:25:00.596 No. Those are [the only?] ones.

S1 01:25:02.415 Okay, so--

S2 01:25:04.268 [Swap?] them?

S1 01:25:04.925 Yeah. So what I'm going to ask you to do is rank them where at the top are the factors that are most important to you, and then at the bottom are least important to you.

S2 01:25:13.575 Okay.

[silence]

S2 01:25:29.560 Okay.

[silence]

S2 01:25:41.199 And then stigma.

S1 01:25:42.163 Okay. So most important, like you said during the interview, is ease of access.

S2 01:25:46.366 Yeah.

S1 01:25:47.248 Okay. Then effectiveness, then cost, then side effects, then how you can take it, then how often you can take it, and finally, stigma.

S2 01:25:58.155 And to me, it's easy because if you can't get it, then its effectiveness doesn't matter. And if it's not effective you're not going to pay. And if you can't pay then the side effects really don't matter. And if the side effects don't matter, pill or injection doesn't really matter to me. And then how often you have to take is going to affect really whether or not there is a stigma, because stigma to me is whether or not you-- it also depends on how people see you as a person who takes it. Like for instance, people knew if you had HIV in the past because there were visible problems with you. But, if how you take it and how often you take aren't visible, then it doesn't matter.

S1 01:26:45.440 Okay, okay. Okay, great. Thank you. Okay, so the next thing we'll do-- so the second of these really quick exercises. So we'll imagine a hypothetical scenario. Imagine I'm going to flip a coin and if it comes up heads, you'll get what's in the heads column, if it comes up tails, you'll get what's in the tails column, right?

S2 01:27:12.471 Hmm-mm.

S1 01:27:13.067 And it's just hypothetical. So based on that knowledge which one of these rows would you pick? Row one, two, three, or four? Does that make sense?

S2 01:27:30.215 In terms of what your odds are? In terms of how much money you would get?

S1 01:27:34.798 Right. So if it's the first row you get $50 either way. The fourth row you get $500 if it's heads, 0 if it's tails. So if you're in that situation, which one would you pick? Which one would you feel most comfortable?

S2 01:27:49.214 Can I write on this?

S1 01:27:50.521 Sure.

S2 01:27:54.133 Because the only thing that I've ever been good at in math is statistics [laughter].

[silence]

S2 01:28:17.011 I would choose row two.

S1 01:28:18.755 Okay. Why is that?

S2 01:28:21.634 Because in row one it's not really worth betting on. You're just going to walk out with $50 in row two. And in row four, I'm not taking 50% chance on $0. Row three. Well, yeah. I could walk out with 200, but there's the equal possibility I could walk about with 10. And in row two, you could walk out with $30, which is still sizable, or you could walk out with $100, even more sizable. Basically, it's if they both have preferable outcomes with the possibility of making more money than in the others.

S1 01:29:00.343 Okay. So you said row two is what you would pick?

S2 01:29:02.026 Mm-hmm.

S1 01:29:02.828 Okay. Great. Okay. Thank you very much. Okay.

S2 01:29:07.928 I'm just wondering how that works out for that [inaudible].

S1 01:29:12.077 Yeah. So really what we're trying to get at with this is your sense of risk and risk-taking. Right? As in row four, you want to talk a lot of risk, or one there's no risk. Yeah. Okay. So thanks so much for talking with me. Do you have any other questions? Or do you think there's anything you want to talk about that we haven't talked about that you think is important?

S2 01:29:43.041 No.

S1 01:29:43.606 No? All set?

S2 01:29:44.345 Mm-hmm.

S1 01:29:45.057 Okay. So then the very last thing I'll do, I'll give you this questionnaire. Just to ask you to fill that out, and then when you're done, go ahead and slide it into this envelope.

S2 01:29:59.271 Okay. Am I currently employed? I'm going to put, yes, part-time. Because I am.

S1 01:30:10.525 Yeah. Okay.

[silence]

S1 01:32:33.543 All done? Okay. [Tight in there, isn't it?]? Okay. So here's the $40.

S2 01:32:51.373 Thank you.

S1 01:32:52.102 Thank you so much, again. We really, really appreciate it. The only thing I have to ask you to do is just print, sign, and date this just indicating that you did receive your payment.

S2 01:33:03.759 That's fine. Right there? And today's the 7th?

S1 01:33:16.004 Yep.

S2 01:33:19.259 All right.

S1 01:33:18.773 And you just print your name also in the first part of the [inaudible]. Okay. Thank you so much.

S2 01:33:32.941 Thank you very much.

S1 01:33:33.805 I really, really appreciate your time and your willingness to talk with me. It was very helpful.

S2 01:33:39.518 Yeah. Of course.

Interview IDA 206

S1 00:46:14.253 Okay. So how about either through your research or through the class, what are some of the ways that you've learned that you can prevent the transmission of HIV?

S2 00:46:27.067 Through the class and through my own research I've learned condoms or abstinence, and that's it. And then through [Health, Outreach, Community Service Center Upstate City 1], that's when I first found out about PrEP.

S1 00:46:40.247 So how did they talk about PrEP when they brought it up?

S2 00:46:44.956 They said that in my scenario it would probably be the best option for me.

S1 00:46:51.919 So why would it be the best option?

S2 00:46:54.487 Because during that time I wasn't using condoms and I was having sex with gay men or men in general.

S1 00:47:04.407 Okay. So I do want to come back to that a bit later. So about HIV and AIDS-- so do you know anything about the treatment options that are available for if you did contract HIV?

S2 00:47:27.702 I do know if it's under a 72-hour window period, you can go to the hospital and you can get it out of your system somehow. I'm not sure how that works, but I didn't figure [inaudible]. I found that out on my own. And I think that's it.

S1 00:47:50.184 So say you didn't know, and it's been after those 72 hours, and you've been diagnosed with HIV, do you know of any treatment options that might be available at that point to help kind of manage the disease itself?

S2 00:48:05.649 I know you can take medication, and it makes you undetectable.

S1 00:48:13.506 So can you explain for me what undetectable means?

S2 00:48:16.878 It means that the HIV virus is still there, but you can't pass it on to somebody.

S1 00:48:21.917 Okay. So have you ever seen anything about HIV or AIDS in media? So like in any of the books that you've read, different movies, TV shows, magazines, things like that?

S2 00:48:36.574 Mm-hmm.

S1 00:48:37.437 So can you give me some examples of places you've seen it?

S2 00:48:40.192 Rent.

S1 00:48:41.112 What was that?

S2 00:48:41.024 Rent.

S1 00:48:42.274 Rent. [crosstalk]. So how does HIV or AIDS get talked about in Rent?

S2 00:48:51.076 Because one of the two main characters, they were [inaudible] needles and that was another way that they were transmitting the disease was the passing of and sharing needles.

S1 00:49:02.955 So I've seen the movie, but just so we have it for the interview. So what happens once the characters are diagnosed as HIV positive?

S2 00:49:17.353 They couldn't afford the treatment for it, so they were just suffering with it. But--

S1 00:49:24.988 Go ahead. Sorry.

S2 00:49:26.127 Oh. But I don't know much else.

S1 00:49:28.913 Sure. Are there any other things in the media that you've seen or talked about HIV or AIDS?

S2 00:49:36.098 I definitely see the HIV AIDS walk-- like a walk. Those signs.

S1 00:49:41.559 Is that something you've ever done or--?

S2 00:49:43.844 Mm-mmm.

S1 00:49:45.289 Is there a reason for that or--?

S2 00:49:49.190 I have no idea, honestly.

S1 00:49:50.942 That's fine.

S2 00:49:55.616 Probably just laziness.

S1 00:49:57.033 Sure. So other than Rent, have you seen in any movies, or books, or anything? No? Okay.

S2 00:50:07.092 Not that I can think of.

S1 00:50:08.513 So I know you mentioned a little bit ago that you were told that you're kind of at one of the most at-risk populations for contracting HIV, particularly because you weren't using condoms. So can you tell me a bit about your decision-making when it comes to using condoms? Like why would you use one? Why wouldn't you use one?

S2 00:50:33.178 Are you talking about personally?

S1 00:50:35.128 Yes.

S2 00:50:37.493 Man, I just didn't want to use them, I guess. I just didn't really care.

S1 00:50:46.019 Did it matter if you were topping or bottoming?

S2 00:50:50.282 What was that?

S1 00:50:51.174 So if you were--

S2 00:50:51.950 Oh, okay.

S1 00:50:51.863 --topping, would you be more likely to use a condom versus bottoming or--?

S2 00:50:57.516 It didn't matter.

S1 00:50:59.351 Were there times that you did use condoms?

S2 00:51:02.050 No [laughter].

S1 00:51:04.362 That's fine. Have your partners asked or suggested using condoms, or--?

S2 00:51:12.244 No.

S1 00:51:13.359 Okay. So even with the girl that you'd been with a few months ago, did you use a condom with her?

S2 00:51:19.931 Mm-mm.

S1 00:51:21.380 Okay. So, any of your sexual partners kind of talk to you about STD transmission or being worried about anything like that?

S2 00:51:35.503 No.

S1 00:51:36.422 No, okay. So other than just kind of not wanting to use condoms, are there any other reasons for not using them, or--?

S2 00:51:47.050 Probably also the embarrassment of going to the cash register, or the actual cost of condoms, even though I do know that you can get them for free at clinics.

S1 00:52:01.759 Okay. So I know you mentioned a bit earlier that you thought you might have contracted chlamydia or gonorrhea, which turned out to be a bladder infection. But have you ever been worried that you might have contracted HIV?

S2 00:52:19.406 Yeah, right before I got the kit.

S1 00:52:26.530 So, what happened that made you worried?

S2 00:52:29.718 It's when I found out that you could pass on the virus through fluids. And then I realized that that was a big problem for me. And then that's when I got just very-- I'm sorry, [right on board?].

S1 00:52:48.882 So after the home kit that you did, have you been tested again or since?

S2 00:52:55.914 Mm-mm.

S1 00:52:57.031 Okay. Have you ever been worried about other sexually transmitted infections or diseases? There was the one incident where it turned out to be a bladder infection, but other than that, have you been worried?

S2 00:53:10.603 Mm-mm.

S1 00:53:12.988 So, thinking forward in time, how worried are you about contracting HIV?

S2 00:53:21.942 If I kept on with the same habits that I did, I'd be very, very worried.

S1 00:53:29.850 Okay, so then are you trying to change your habits now, or--? So how are you trying to change them?

S2 00:53:39.178 Honestly, just staying in school [laughter]. Or staying home and just ignoring my phone.

S1 00:53:51.331 Another option, have you-- or, go ahead.

S2 00:53:56.342 I'm sorry. More trying to stay abstinent than anything.

S1 00:53:58.999 Sure. But not necessarily by using condoms, or--?

S2 00:54:06.070 What was that? So sorry.

S1 00:54:08.083 So we're talking about ways that you've kind of been trying to change your behavior.

S2 00:54:11.857 Yeah. Yeah, not like that. Yeah. I get what you're saying now.

S1 00:54:14.878 More by just refraining from having sex at all?

S2 00:54:18.067 Yeah.

S1 00:54:18.871 Okay. So if you had to put this kind of like on a numerical scale, your being worried about contracting HIV, where 0 is like absolutely never going to happen, impossible, and 100 over here is absolute certainty, like 100% guaranteed going to happen, how would you rate your risk for contracting HIV?

S2 00:54:47.064 Probably 34.

S1 00:54:48.573 34%?

S2 00:54:49.709 Mm-hmm.

S1 00:54:50.736 So is that based on old habits or what you're trying now?

S2 00:54:55.554 Old habits. But if it was old habits, then 100. It should have happened.

S1 00:55:01.742 Right. So then the 34 is kind of incorporating some of what you've been trying to do lately?

S2 00:55:07.134 Mm-hmm.

S1 00:55:08.040 Okay. All right. Great. So is there anything else about HIV or AIDS that's important to you or stands out before you go on to the next part?

S2 00:55:19.589 No.

S1 00:55:20.152 Okay. Sorry, just to revisit this, you said you haven't talked with your parents about HIV at all?

S2 00:55:29.422 Mm-mm.

S1 00:55:30.237 No. Okay. So when you were worried about potentially having HIV, before you got the kit, was that something you talked to anyone about, or--?

S2 00:55:41.062 Mm-mm.

S1 00:55:41.666 No? Okay. So then the next set of questions is much more about-- it's referred to as HIV pre-exposure prophylaxis, or PrEP for short. So in the questions that kind of follow this, it's basically just kind of saying what do you know about the drug itself, the things surrounding it, how have you learned it? Those kind of things. So like I'd said before, I want to come back to this. You said in your ACR class they talked about PrEP, right?

S2 00:56:17.787 Mm-hmm.

S1 00:56:18.270 So can you tell me a bit more about that? Like how did they talk about it, what kind of things did they say?

S2 00:56:24.085 Well, it wasn't really a class. It was when I was visiting. Went to get checked out, and they were describing that option for me. And they described it as more of like a second barrier against HIV, and not like, "Oh, don't use condoms anymore. All you need is this." And what they said was basically it just prevents you from contracting the disease.

S1 00:56:55.364 So is PrEP something that you've considered taking before?

S2 00:56:57.164 Mm-hmm.

S1 00:56:59.416 But you are not taking it now?

S2 00:57:02.091 Right.

S1 00:57:03.148 So can you explain a little bit about what's kept you from taking it, or--?

S2 00:57:06.457 Honestly, I just don't understand how you go about getting it or if there's a cost behind it. I doubt, but there might be. Like if my insurance would cover it. You know, just stuff like that.

S1 00:57:26.140 Of course. So other than the [Health, Outreach, Community Service Center Upstate City 1], have you ever found out about PrEP anywhere else?

S2 00:57:33.989 Mm-mm.

S1 00:57:34.702 Okay. So is it something that you've looked into online at all, or--?

S2 00:57:39.082 Mm-mm.

S1 00:57:39.837 Have you seen anything about PrEP on Grindr?

S2 00:57:45.923 Mm-hmm.

S1 00:57:47.131 So what kind of things show up on there?

S2 00:57:50.198 Definitely the study.

S1 00:57:52.541 The study came up on Grindr?

S2 00:57:54.081 Yeah.

S1 00:57:54.884 Really?

S2 00:57:55.790 Somebody messaged me on Grindr about the study, and that's how I got to, yeah.

S1 00:58:02.807 Do you know if they were affiliated with the project, or just someone who had kind of gone through it already and--?

S2 00:58:08.924 Oh. It was probably somebody who had probably gone through it already because there's these guys who are [inaudible], yeah.

S1 00:58:16.138 Hmm. That's interesting.

S2 00:58:17.485 Good way to get the word out.

S1 00:58:18.123 Yeah, definitely. We were going back and forth whether we should actually make a profile and try to recruit that way. I think ultimately we decided not to. But it's really interesting to hear that it's still circulating [out there?]. But so have you-- so this person that you talked to that told you about the study, did they actually tell you about PrEP at all, or was it just, "Hey, these people are doing a study. Go talk to them"?

S2 00:58:50.878 Yeah. I mean, he said they're doing a study on PrEP, and they gave me the requirements to be in the study and the email. And I went from there.

S1 00:59:01.054 Sure. Great. Well, I mean, I'm glad you're participating.

S2 00:59:04.755 Thank you.

S1 00:59:06.464 So I guess I'm trying to also ask too, on Grindr I know occasionally there's different pop-ups?

S2 00:59:11.143 Yeah.

S1 00:59:13.849 I haven't used it in a long time, but I've heard that some of them do talk about PrEP. So do you know what I'm talking about at all?

S2 00:59:22.210 Honestly, I've never seen a pop-up with that. Usually, when there's an advertisement on Grindr it's [one word?].

S1 00:59:31.511 Okay. I know some people have mentioned that they've seen a little bit about PrEP on there. Especially if you look at someone's profile, it will tell you if they're on PrEP or not now?

S2 00:59:42.548 Oh, yeah. Definitely.

S1 00:59:44.589 Oh. When you've been talking to any guys on Grindr, has that come up in conversation, the fact that they're on PrEP or not?

S2 00:59:53.796 Mm-mm.

S1 00:59:53.983 Okay. So other than [Health, Outreach, Community Service Center Upstate City 1], have you found out about PrEP any other way?

S2 01:00:01.038 No.

S1 01:00:01.823 Okay. So, your parents haven't talked to you about it?

S2 01:00:06.068 My Mom doesn't even know about it [laughter]. She was confused at what I was doing today.

S1 01:00:10.796 Did you tell her?

S2 01:00:11.762 Oh yeah.

S1 01:00:12.384 Okay. Well, hopefully by the end you'll have more information, if you want to share it with her, you can [laughter]. Have any teachers talked to you about PrEP at all?

S2 01:00:22.636 No.

S1 01:00:23.813 In your health class or anything?

S2 01:00:24.738 No.

S1 01:00:25.643 So then just [Health, Outreach, Community Service Center Upstate City 1]?

S2 01:00:27.111 Mm-hmm.

S1 01:00:30.360 Okay, are there any-- similar to how I asked about HIV, but have you seen anything about PrEP in media? Movies, books, TV shows?

S2 01:00:40.008 Mm-mm.

S1 01:00:40.944 Okay. Do you know anyone who's taking PrEP right now?

S2 01:00:46.215 I do.

S1 01:00:47.362 You do?

S2 01:00:47.874 Mm-hmm.

S1 01:00:48.991 Okay. So is it just one person, or--?

S2 01:00:51.847 It's just one person.

S1 01:00:53.858 And so how do you know this guy?

S2 01:00:56.171 He lives around the corner from me, and he's a guy that I'm talking to on Grindr at the moment.

S1 01:01:02.993 So, he's talked to you about PrEP, then? Or do you just know from his profile?

S2 01:01:07.310 I know from his profile [laughter].

S1 01:01:09.140 Okay. Have you tried to talk about it at all, or--?

S2 01:01:13.313 [inaudible].

S1 01:01:14.430 Okay. Now, is he someone that you've hooked up with before, or just talking to?

S2 01:01:19.550 Just talking to.

S1 01:01:20.607 Okay. Right, so we covered that. So I want to ask you about some things that other people have told us are important for thinking about deciding to use PrEP. So if you had mentioned how you would get it, the cost, the insurance, like those things you would think matter to you. So there's other things that people have brought up. So I'm going to kind of go through those to see what you know about them. So there's no right or wrong answer, it's mostly just going to be, "What do you know about this?" And it's perfectly fine to say, "Nothing," or, "I don't know."

S2 01:01:58.835 Okay.

S1 01:01:59.548 Just answer as freely as you can for each one.

S2 01:02:02.054 Okay.

S1 01:02:03.944 So I think you mentioned this a little bit. So, what if anything, do you know about the cost of PrEP?

S2 01:02:11.068 Nothing.

S1 01:02:12.034 Nothing, okay. Have you heard about anyone who's had difficulty paying for it?

S2 01:02:21.320 No.

S1 01:02:23.482 Do you know if there are insurance plans that cover PrEP?

S2 01:02:27.587 Mm-mm.

S1 01:02:31.168 If your insurance did cover PrEP, would you feel comfortable using it?

S2 01:02:36.963 Yeah, totally.

S1 01:02:38.141 Okay. So, are you on your own insurance, or a parent's, school?

S2 01:02:42.687 I'm on my Mom's insurance.

S1 01:02:43.612 So, even though being on your Mom's insurance, you don't think she would react negatively to you being on PrEP?

S2 01:02:52.517 I mean, she can be awkward about it because she's awkward about everything but [laughter] other than that, I don't see a problem.

S1 01:03:02.497 So what, if anything, have you heard about where you can go to get PrEP, if you wanted to start taking it.

S2 01:03:12.658 [Health, Outreach, Community Service Center Upstate City 1] talked to me about a class that they did have about PrEP and that I should go to it and I never did. And they were probably going to [describe data?] like where to go and stuff.

S1 01:03:26.369 So was there a reason why you didn't go to that class then, or--?

S2 01:03:30.011 I was probably just busy that day, or something, or transportation.

S1 01:03:34.799 So what, if anything, have you heard about what you have to do to take PrEP? So how often do you have to take it? What form is it in? How often do you have to go to the doctor's? What have you heard about that?

S2 01:03:53.050 I'm probably imagining this but I feel like it's kind of like a birth control pill, where you take it like every set hour. It probably isn't [laughter], but.

S1 01:04:07.196 So I'm not here to-- right now, I'm not here to tell you if you're right or wrong. This is just really, what do you know? Well, so then, how about what form is it?

S2 01:04:18.172 I don't know, honestly, but I [would say?], pill?

S1 01:04:21.703 Okay. Have you heard anything about having to go to the doctor's office while you're on PrEP?

S2 01:04:29.219 Mm-mm.

S1 01:04:30.155 Okay. So PrEP is a pill. So would you feel comfortable taking a pill every day like that?

S2 01:04:43.003 Yeah.

S1 01:04:44.712 I know some people have this kind of aversion to swallowing pills. How would you feel if PrEP was an injection that you got once every few months?

S2 01:04:57.601 I guess that would be fine since it's just every few months. I'm okay with injections, honestly.

S1 01:05:05.631 But if they were more frequent than that would that potentially be a problem?

S2 01:05:11.004 If they were more frequent than I would probably just take the pill.

S1 01:05:14.102 And how would you feel if PrEP was an implant that you could have under your skin, like in your arm or something?

S2 01:05:22.644 I don't really know much at all about implants. I don't really see a negative or-- pretty neutral, so.

S1 01:05:35.492 Okay. So what have you heard about the effectiveness of PrEP?

S2 01:05:44.023 Well, the lady at ACR described it as a second barrier so I'm going to assume it's effective. But you shouldn't entirely count on it. I don't want to say that though. But I'm not sure.

S1 01:05:57.305 So how effective would it have to be for you to consider taking it?

S2 01:06:03.373 Like 97% of a chance.

S1 01:06:08.595 So reducing your chance by 97%?

S2 01:06:11.885 Yeah.

S1 01:06:12.175 Okay. So say, if PrEP only cut your risk of contracting HIV in half that wouldn't be enough for you to take it?

S2 01:06:23.586 It would also have to depend on-- I would just have to see the value, I guess, in it. It would have to also depend on the cost if there was a cost.

S1 01:06:34.664 Okay. So even it was only say, 50% effective if it's-- the cost would be another big factor for it?

S2 01:06:40.841 Yeah.

S1 01:06:41.867 Okay. So have you heard anything about the side effects from taking PrEP?

S2 01:06:48.376 Mm-mm.

S1 01:06:49.402 No. Okay. Do you think friends or potential partners look favorably or badly on people who take PrEP?

S2 01:06:58.809 They probably look favorably on people who take PrEP.

S1 01:07:01.586 So why do you think that?

S2 01:07:03.869 Just because it's that second barrier. You really care about yourself. You're being really safe.

S1 01:07:11.083 So if you were taking PrEP and say, you're going to hook up with someone or you were in a relationship, would you tell your partners that you were taking PrEP?

S2 01:07:21.546 Mm-hmm.

S1 01:07:22.361 So why?

S2 01:07:23.074 Just so they knew that there wasn't any risk-- well, that there was less of a risk.

S1 01:07:29.957 So would you expect a sexual partner to tell you if they were taking PrEP?

S2 01:07:36.809 That's a good question. Yeah. I would like them to. Yeah.

S1 01:07:42.846 Okay. And why?

S2 01:07:45.220 Because if they weren't taking PrEP then I probably would want to do a-- I would probably wear a condom. So that's the only reason why I would want to know.

S1 01:08:00.331 Okay. And how would you feel if someone told you that they were taking PrEP, would that change the way you think about them, or--?

S2 01:08:11.802 Mm-mm.

S1 01:08:12.617 No? Would it make a difference if this was someone you've been talking to for a while and then out of nowhere they say, "Oh, by the way, I'm taking PrEP now."

S2 01:08:23.231 Cool.

S1 01:08:24.850 Okay. Sure. Okay. So great. So those are some of the other things that people have said are important. So things like cost, ease of access, where do you get it, how do you take it, those kind of things. So I actually have a short sheet, just one-sided. It just has a bunch of different facts about PrEP so it's a lot of information about the drug itself, so I'd like you to just take a minute or two to read through this. And you're welcome to take this with you when your interview is over.

S2 01:08:58.963 Okay.

S1 01:09:00.582 If you have questions about what something means, let me know. I'll do my best to answer that [laughter] but I'm not--

S2 01:09:06.831 Okay.

S1 01:09:08.087 --not exactly a medical expert or anything [laughter].

[silence]

S2 01:11:08.343 Okay.

S1 01:11:09.551 So are you finished going through it?

S2 01:11:09.902 Mm-hmm.

S1 01:11:10.928 Great. So, based on what's there, is there anything that was kind of surprising to you, or that you just didn't know before?

S2 01:11:19.338 Oh yeah. The part where it said that with how many people were taking PrEP only one person has gotten it, and it was a rare strain of HIV. I thought that was really interesting. I like those [inaudible] [laughter].

S1 01:11:35.480 Sure. It shows that it is effective, right? So what about some of the things like-- I think this sheet talks about insurance a little bit, or different plans that help. So based on this, how has your decision towards taking PrEP changed at all?

S2 01:11:53.851 Honestly, I might have to look into PrEP because, I mean, if my insurance will cover it. Well, almost all insurance plans-- but then again it also says that there are several PrEP providers.

S1 01:12:08.608 Yeah. So how about the side effects for PrEP. You'd mentioned before that you didn't know anything about them. So is there anything that kind of stood out to you that might be something you're worried about, or--?

S2 01:12:24.112 Well, the only thing I would be worried about, personally, is the headaches and nausea for the first one to two weeks. Just because I have that now [laughter], right?

S1 01:12:37.012 So to what extent do you think others in the LGBT community know about some of this information that's here?

S2 01:12:48.079 I would have to guess, not much. They might know what PrEP is but this is definitely an eye-opener.

S1 01:13:00.374 Sure. Do you think if more information like this was publicly available or if people knew these kind of facts, do you think it would affect how-- or people's choosing to use PrEP?

S2 01:13:15.218 Honestly, yeah. I could just see a poster at the clinic or something that describes it's effectiveness.

S1 01:13:23.640 And you think that would make people take it or be more likely to take it?

S2 01:13:27.654 Yeah, or at least look into it.

S1 01:13:29.907 All right. So thank you for sharing all that, for running through that with me. So there's just a couple other small things that I'd like to do. So the first thing is-- so I have these index cards and there should be seven of them. No, there might be eight. Yeah, I think there's eight. So these are some of those factors that I talked to you about before that people said, "These are important when I'm thinking about taking PrEP." So I want you to look through these cards. So it's things like cost, ease of access, how do you take it, how often. So I want you to look through those and see if there might be something that you would add to it. Like for you, personally, is important but it's not in this pile. Okay, there are seven. I just miscounted.

S2 01:14:37.905 Probably just the transportation to get the actual medication. So ease of access. [inaudible].

S1 01:14:44.398 So you want to include that in ease of access?

S2 01:14:45.081 Yeah.

S1 01:14:46.107 Because I do have a ton of note cards if you want to write on any of them. So it's up to you.

S2 01:14:51.741 No. I think ease of access describes it perfectly.

S1 01:14:54.065 Okay.

S2 01:14:54.657 Clear.

S1 01:14:56.227 Sure. All right, so--

S2 01:14:57.242 [Ease of access?] [laughter].

S1 01:14:58.906 So, taking these cards, I'd like you to put them, either across the table or up and down, in order of what's most important to you, to what's least important.

S2 01:15:11.633 Okay.

[silence]

S2 01:15:41.731 It's kind of like this.

S1 01:15:43.180 Okay. So, at the top, so the most important, you have cost. Then ease of access, then effectiveness, then side effects, then how often you have to take it, then how you can take it, and then last is stigma.

S2 01:15:59.186 Yes.

S1 01:15:59.779 So can you explain to me a little bit about why you put them in this order?

S2 01:16:04.337 Well, cost is definitely number one right now, just because we already have a ton of medical bills, with the two grandkids that my grandmother left behind. Ease of access, I take a bus and I walk a lot, so I would have to walk or take a bus there. Effectiveness, I would want it to be effective enough for me to want to take it. Side effects, this doesn't really matter to me as much. But if there was some crazy outlandish side effect, then I would definitely want to know about it. And then how often you have to take it, I forget to take pills all the time. So honestly, yeah, let's do that.

S1 01:16:55.184 So, you moved the frequency just above side effects?

S2 01:16:58.988 Yeah.

S1 01:16:58.840 Okay.

S2 01:17:00.942 And how you can take it, I take pills and I've had injections before, so that's kind of like-- it doesn't really matter.

S1 01:17:12.955 Sure.

S2 01:17:14.211 And the stigma, I don't care what people think [laughter]. I couldn't see why there would be a bad stigma around it.

S1 01:17:22.603 Okay, well thank you for explaining the thought process behind it. That's really helpful. So if you want, can you kind of put them back together. And then there's one or two other little things, if I could just find the sheet. Okay. So, this little exercise is on this sheet of paper. So I want you to take a look at this. Not much there. So what I want you to do is imagine that I am going to flip a coin and you're going to pick one row. And so based on the row you've picked, you would get the money for the coin landing on heads or landing on tails. Unfortunately, we don't actually have the funds to do this for real but so hypothetically, you're going to pick one row. And so when you've picked one, let me know.

S2 01:18:24.231 Could you explain again picking a row for what?

S1 01:18:26.498 Sure. So you're going to pick one of these four rows. And so, say you pick row one, if the coin lands on heads you get 50--

S2 01:18:35.754 Oh, okay.

S1 01:18:36.509 --if it lands on tails--

S2 01:18:36.980 Okay. I get it.

S1 01:18:37.905 Whereas with row four, if it lands on heads you get 500 or zero.

S2 01:18:44.534 Row one.

S1 01:18:45.349 Row one. So you're going with the 50/50 outcome?

S2 01:18:48.447 Yeah. I could kind of see like where it's going with that but-- maybe. I don't know.

S1 01:18:53.046 So where do you think I was going with that?

S2 01:18:58.136 The effectiveness of PrEP. Is that where it's heading towards?

S1 01:19:03.740 Kind of. Can you tell me a little bit more of like what--?

S2 01:19:06.912 I don't know. I guess I took it as it's better to have that 50% chance than getting hit with that 500--

S1 01:19:21.130 Okay.

S2 01:19:22.670 If that makes sense.

S1 01:19:23.654 No, it does. I'm just curious what you think-- if you could explain maybe a little bit why you picked row one? That's what--

S2 01:19:29.420 Oh, okay. Row one because either way, you're going to get $50 but with the rows below it, of course, your tails value significantly decreases. So if you got tails you'd be very upset if you had row four.

S1 01:19:47.418 Okay. Good. So yes, it's a way to kind of think about it or look at risk assessment. So you were pretty close when you were saying like, "Oh, it's about PrEP's effectiveness," and how much you're willing to give or take, right. Okay. So thank you, [Tom?]. And then the last thing that I have and I mentioned at the beginning-- it's this really short questionnaire. So none of your-- actually, I have a pen. There we go. So none of your information is actually going to be attached to this. This is more just so that we can look at some of the demographic kind of data for the study. So as you go through it if there's questions that you don't want to answer or don't feel comfortable, just skip them. Some of them will start at the bottom of one page but then the responses are at the top of the next. So if you see a question with no boxes to check, it's probably on the next page.

[silence]

S1 01:24:55.415 Are you all set?

S2 01:24:56.260 Yes.

S1 01:24:56.430 Okay. Great. Thank you for doing that.

S2 01:24:58.724 No problem.

S1 01:25:00.222 So just kind of to wrap this up a little bit, I have another sheet here for you. So at the top here are a list of places in [Upstate NY City 1] that have PrEP available, and so there's the Onondaga County Sexually Transmitted Disease Clinic or Upstate Immune Health Services and there's phone numbers there and when they're open. On the bottom half are different counseling services around [Upstate NY City 1]. One of the risks of participating in these kind of in-depth interview studies is that you can touch on experiences or memories that might be traumatic or difficult to deal with. So kind of to help alleviate them a little bit, we do have this resource guide that we've put together. So if you want to make an appointment with one of these places and you want our help to do so, please reach out to us and we'll do everything to help you with that. On the back, there's substance abuse resources. And then at the bottom, there's an LGBT center, I believe.

S2 01:26:05.891 Yeah. I've been there before.

S1 01:26:06.857 Oh. I haven't had a chance to go yet, so I [crosstalk] [laughter].

S2 01:26:14.455 I don't know. The people there, I guess they're just-- they're very colorful.

S1 01:26:23.771 What do you [crosstalk]?

S2 01:26:24.921 Not kind of my crowd people. A lot of people that are at the Cuse center right now are pretty goth. Pretty dark. Yeah.

S1 01:26:33.037 It seems like maybe the opposite of colorful.

S2 01:26:35.290 Yeah. Kind of ironic.

S1 01:26:39.233 Sure [laughter]. Okay. But so, again, if you want help, either scheduling an appointment to get PrEP or with any of the counseling services, I believe you have Aaron's email, phone number, his [crosstalk].

S2 01:26:55.514 Yes.

S1 01:26:55.714 Okay. You have my email. You have--

S2 01:26:57.786 Yes.

S1 01:26:58.740 You have my phone number as well. So if you want help with any of this, please reach out to us. As a reminder, at the back of the consent form, you do have the contact information for participant advocates. So if you want to talk to someone who's not necessarily even part of the project [inaudible] that isn't working with us, those are who you would reach out to for that. So I just want to remind you that you do have a variety of ways to reach out if you need something. So then the last thing is to just get you paid for the interview. So did you need travel compensation for--?

S2 01:27:42.470 $2 for the bus.

S1 01:27:43.829 Okay. So I'm going to have to stop into the office and grab $2. I don't have any singles on me [laughter]. Okay. So I'll do that. So if you just want-- this is for budgetary purposes really so I can kind of track where the survey money is going. So if you want to just put the date here, and then print, and [crosstalk]--

S2 01:28:08.581 [crosstalk] today is the 5th, right?

S1 01:28:09.667 The 5th.

S2 01:28:10.474 [inaudible].

[silence]

S1 01:28:29.088 Great. Okay. So here is the 40 for participating in the interview.

S2 01:28:39.955 Thank you.

S1 01:28:41.483 And then I'll get you two singles in just a second.

S2 01:28:43.475 Okay.

S1 01:28:45.003 So before I step out to do that, though, were there any other kind of questions, or concerns, or thoughts that you might have?

S2 01:28:53.757 Do you actually know where the Onondaga County-- where this is actually located?

S1 01:29:00.640 The STD clinic?

S2 01:29:02.481 Yeah. The first one.

S1 01:29:04.522 I'm not sure. I can look that up while I step out.

S2 01:29:07.729 I could look it up. I was just wondering.

S1 01:29:10.464 I know that there is a clinic downtown that does the kind of on-demand STD testing and I think it might be the same one, but when I [inaudible]--

S2 01:29:20.048 Oh. At the Civic Center?

S1 01:29:22.843 I think so.

S2 01:29:23.749 Okay. Yeah. That makes sense. [inaudible].

S1 01:29:26.968 Because I know that one and so I'm pretty sure they're the same one. But were there any other questions or concerns? Anything that you thought of earlier that like, "Oh. This is important," but didn't have a chance to bring it up?

S2 01:29:39.506 No.

S1 01:29:41.305 Okay. Great. So I'll turn this off. So thank you again.

Interview IDM 104

[01:43:57.12] I: So then how about ways to prevent the transmission of it?

[01:44:01.08] R: Definitely condoms. There are dental dams. I don't know how much dental dams do. But whatever, they exist. But also, with drugs like PrEP and there was something else. Truvia? Or is that the same thing?

[01:44:22.22] I: Truvada?

[01:44:25.04] R: Truvada.

[01:44:26.09] I: I think it's the company that makes PrEP. I'm not totally sure about the specifics.

[01:44:31.01] R: But I know that there was PrEP and then another pill. But PrEP is the more popular one that I know of. I've never, I've never taken the pill. But yeah. I've never taken PrEP.

[01:44:48.02] I: How about treatment options for HIV AIDS? Do you know anything about those?

[01:44:51.21] R: I don't know. I mean, I've seen my friend's medicine. And I feel like it would depend on the actual person because there are levels of..

[01:45:07.14] I: Like you said your friend is undetectable?

[01:45:08.27] R: Right. So he had told me that he when was diagnosed, he had hives all over him. He was really, really sick all the time. Couldn't get out of bed. And some sort of like a number for something. Like he basically had AIDS. But when he was taking his medicine, he became undetectable. But yeah, so I would imagine that the amount of medicines that you take would depend on how severe it is to you. Or how severe your condition is.

[01:45:53.14] I: Have you ever you thought you might be infected with HIV? Are you worried about that?

[01:45:57.25] R: I have like when I had gonorrhea, I thought it could be it. I was freaking out. But I don't think there. I've always questioned whether or not like the one time or whatever time I've been with a guy, could it have, cause I don't always ask. But I do always have condoms. They just are always used. Or they haven't always been used.

[01:46:35.19] I: Are there circumstances for why they haven't been used?

[01:46:38.26] R: Not any that are like strict. It's just not happened. Or haven't just grabbed the condom. But yeah. Last summer, I was out studying and I met up with a guy who was who wasn't from, he was visiting [Upstate NY City 1]. And he so we met up, hooked up. And then maybe a week later, he had a car accident. And basically I thought that he or no, was it, it was either right after we hooked up or a couple days after, he had a car accident. Cause I wanted to see him again. And I felt that he didn't want to see me and I was like why doesn't he want to see me. So I was like freaking out, thinking that he might have been positive and that he like just you know, but also I know that it's illegal to not tell someone your status. So I kinda like calmed myself down. I mean I wasn't super super freaking out and that was after I had gotten gonorrhea. I had been treated for gonorrhea. But I've definitely like been skeptical at times when I meet guys and they don't want to talk to me anymore, basically, if I wanted to talk to them or meet up again.

[01:48:28.23] I: So if you had to say in the future, how worried are you about contracting HIV?

[01:48:33.23] R: I am, I would say I'm like 60 40 percent. 60 percent not worried, 40 percent not worried. Because I know like being in situations where I'm about to have sex and knowing there's a condom there and the guy didn't grab the condom and yes I could say. And I have. Many occasions said I have condoms and you know. And I don't know. I don't think it's because I feel like the guy wouldn't want to have sex without a condom but a lot of guys like some guys have short dicks and they can't wear, well they can wear condoms definitely but they don't want to. But that's only an excuse not to wear a condom. So there are those situations and I'm just like I'm not continuing basically or I'd rather not continue if you don't wear a condom. Yeah, so that's pretty much.

[01:49:39.10] I: So like 60 40?

[01:49:40.05] R: Yeah. I mean and I do know like I, I wouldn't say the type of guy I meet, but a lot of guys that I have met aren't, you know, don't just, aren't as social or as in the know as I am. So a lot of guys in, I wouldn't say, so basically guys who I met, some may be local, some in impoverished areas in [Upstate NY City 1], like they might not have the best resources to know about safe sex basically. They know about AIDS and HIV, but not, you know, how to not and they probably even if they did, it's probably even hard cause getting the medication or whatever, you know, something may be preventing them from doing that, you know. It's different for everyone. I mean the medicine's available for everyone, but, or maybe not, I don't know. It might not be available to everyone. Is PrEP available to everyone? Or it depends on like insurance?

[01:50:59.13] I: It depends on, yup, like accessibility, like if you have access to doctors, if you the right insurance plans. But actually that's kind of a good segue to move into a discussion about PrEP. So that's the last section of the interview. So it's just kind of seeing what you know about HIV pre-exposure prophylaxis, or PrEP. A daily pill that you take to prevent HIV. So for starters can you tell me what you've heard about PrEP in general?

[01:51:29.20] R: Well I've heard that it was a pill you take daily that prevents HIV or helps prevent HIV. I've seen, I think I've seen what the tablets looks like. But it doesn't prevent or it doesn't cure HIV AIDS and I read an article, I don't know how true it was, but it was posted by Logo or Out about a guy who had been taking PrEP and he wasn't HIV positive, but he ended contracting HIV, even though he was on PrEP. But I haven't heard a lot of stories like that.

[01:52:22.18] I: I believe he's the only case and it was a rare drug resistant form. How have you found out some of this information? You said that you read an article aboout it?

[01:52:32.28] R: I've read articles, mostly through the internet or even like ads on dating apps, stuff like that. But mostly through like social media.

[01:52:48.02] I: Is it something you've talked with-

[01:52:49.28] R: Well also like when, well I don't know, when I was treated for gonorrhea, it wasn't, like they didn't tell me about PrEP or anything like that but I saw the pamphlet like somewhere, on the table. But yeah so I've seen posters and whatnot, so it's definitely been on our radar.

[01:53:15.02] I: Have you friends, have you talked about it with friends or other guys you've been seeing?

[01:53:17.14] R: No. I haven't. And I don't think my friend is taking PrEP. I think he's taking something else. I don't think it's PrEP. I'm not sure. I haven't asked. But I haven't talked about PrEP with anyone.

[01:53:40.08] I: So then do you know anyone who's currently on PrEP at all?

[01:53:42.16] R: Yes. I mean not like, I've met people who are on PrEP. But I have never talked to them about PrEP.

[01:53:56.12] I: Have you ever considered taking it, like from some of the brochures or ads that you've seen?

[01:53:59.28] R: I have. But like I said, I've never taken a pill.

[01:54:08.03] I: Is that just personal choice or?

[01:54:12.16] R: Wellllll it's not *laughs* I mean, I don't know, like, it's so it's so dumb like if I can suck a dick, I can take a pill, basically. But it's like I tried taking a pill when I was younger and I threw up, so I've only taken liquid medicines. But like the idea of swallowing a pill, I think about it, there was one time where I choked on, well I choked on steak many times, like three times, and it was kind of similar to when I tried to take a pill and I threw, I couldn't. So but it was all a mental thing like me thinking about putting this thing in my mouth and swallowing it whole. Not chewing it. But yeah so like I definitely, if I got PrEP of course I would take it, but I would have to, you know. I've never asked my or seen about or taken the measures to get PrEP through my health insurance or anything like that. But I do have, I think I have that option.

[01:55:18.02] I: So is it really the just the pill form kind of what's keeping you from or?

[01:55:24.19] R: It, I could, I feel like I could swallow smaller pills. But the biggers ones, I think they just scare me. Maybe.

[01:55:35.12] I: Sure. That's understandable.

[01:55:37.29] R: But people do it. So, you know.

[01:55:42.11] I: So next I wanna ask you about some things that other people have told us are important when they think about PrEP, some of the factors that have affected their decision making. So there's really no right or wrong answers. I'm gonna say some characteristics about PrEP and just tell me what you think about them. So what, if anything, do you know about the cost of PrEP?

[01:56:06.03] R: I don't. I don't know. I'm not sure. So I know Martin Screlli whatever his last name is was like or I think he hyped up part of the prices that was used in drugs like PrEP. I think drugs like PrEP were part of that. Or drugs that prevented HIV basically or drugs that helped prevent HIV. But I didn't check up on like current prices or you know cause I never thought they were the same price for everyone. But I also don't know.

[01:56:49.15] I: But so you said you think your insurance does potentially cover it?

[01:56:53.15] R: Well I have the school's health insurance. I've just never looked into it.

[01:57:01.24] I: So what, if anything, have you heard about where you can go to get PrEP if you wanted it?

[01:57:07.08] R: Health centers. The one on campus. And then there are local centers.

[01:57:13.16] I: Would you say it's easy, challenging to get it?

[01:57:17.20] R: I would say it's easily accessible. I mean, for those who can.

[01:57:28.10] I: Right. So what, if anything, have you heard about taking PrEP? How often do you take it? I mean, we talked about this a little bit before.

[01:57:45.00] R: I know it's a daily pill, but I don't know if that's like first, I think it's like every single day. I don't know if it's a certain amount of months or in increments. I feel like you have to consistently take it. If you're having unprotected sex basically or even if you're having protected sex bascially. It's just better to take it. Some people just do that.

[01:58:11.04] I: Do you know or have you heard, do you have to visit a doctor frequently?

[01:58:15.02] R: I don't know, but I would imagine. Well I know in the case of the guy who did contract HIV when he was taking it, I think he was part of a study. Not sure. I feel like. He may not have been a part of the study. But he was like reporting to the doctor.

[01:58:37.18] I: Do you think a daily pill would be too much for you to take then?

[01:58:42.10] R: I don't think so. Like people take daily pills. Like I don't know the effects of the drug. Or I mean, I do, but I don't know side effects, if any. But people take daily pills all the time, sometimes more in a day than just one.

[01:59:01.17] I: But for you personally? I know you're somewhat averse to-

[01:59:03.01] R: No, like I wouldn't, pill taking hasn't been part of my daily life ever, but I mean, it's not, I don't think it would be hard for me to take a daily pill.

[01:59:13.15] I: So have you heard anything about the effectiveness of PrEP? You did mention the one article you had read. But have you heard anything else about how effective it is at preventing HIV transmission?

[01:59:25.10] R: I feel like I've seen that it was like 99 point 9 percent effective. For most people. But I feel like that also varies. So I would say it's pretty effective or it has been.

[01:59:43.09] I: So for you personally, how effective would something have to be for you to have to take it? So say if the pill-

[01:59:49.24] R: I don't know even know if it's that. There are a lot of very very effective things that like I am just not part of, like my life, my daily life. But I feel like, like I feel good about knowing that's an option. Not necessarily like. I don't want to put my position, put myself in a position where it's like too late for me to take it. But yeah, I don't know. I do tend to do things that are last minute a lot. But yeah, I know that it's highly effective.

[02:00:27.21] I: So say if it were only 50 percent effective, and it decreased your chance of contracting by 50 percent, is that something you would still take?

[02:00:35.25] R: Hmmmm I feel like.

[02:00:38.00] I: Or would you want it to be more?

[02:00:39.17] R: I feel like it's better than nothing but also taking in like a lot of people have I don't know. It's there are many an- I feel like there are many answers because a lot of people would have other things to consider as far as you know if the drug is only 50 percent effective and they are you know this sick or whatever they might not consider taking it. Or I feel like I would still take it even though I don't but I feel like I would still take it if it was 50 percent. Maybe if it was 45 percent, I probably wouldn't. But like 50 50 because I know everything can't be the same for everyone all the time. But I'd say 50 percent's better than nothing.

[02:01:38.20] I: So then you said you haven't reallly heard anything about side effects, right?

[02:01:41.13] R: No.

[02:01:42.26] I: Okay. Do you think your friends, potential partners, or you look favorably or badly on people who take PrEP?

[02:01:50.26] R: I do not think so at all. I don't think so. I mean, I feel like it's definitely a benefit to taking PrEP and there should be. I wouldn't see why there's a stigma in taking PrEP?

[02:02:07.11] I: Have you heard anything about a kind of stigma around PrEP or about people who use it?

[02:02:12.19] R: I've seen hints. I've seen hints. Definitely on social media as far as well Grindr. Cause people would say they're on PrEP, negative but on PrEP. But then some people think that's like a way for people to just be, like I'm a sex positive person, I don't slut shame and I don't. Some people do slut shame people who take PrEP because they see them as being more free to have sex because they are taking PrEP basically. Or like free to have more sex because they're taking PrEP. So I've definitely seen that. But that's pretty much about the only thing I've seen, negative wise.

[02:03:03.19] I: Have you heard anything from friends or anyone?

[02:03:08.18] R: No.. I mean, I've only met people who've taken it. I've never known them long enough to.

[02:03:18.07] I: To get into that kind of conversation.

[02:03:19.07] R: Yeah.

[02:03:19.24] I: Okay. Thank you for answering all of those. So here I have a fact sheet about PrEP. So just a bunch of different information about the drug itself. You're free to take that with you at the end of interview if you'd like. So I just want you to take a minute or two to read over it. If you have questions about something or want clarification, please ask. I'll do my best to answer. I'm not an expert on it, so.

***Reading over fact sheet***

[02:05:44.21] R: Wow. I didn't know that. That they're trying ways to get PrEP as injections or infusions or implants.

[02:05:53.23] I: So it would be something similar to the birth control that you have in your arm. So does any of the information in there change the way you think about PrEP?

[02:06:07.03] R: No.

[02:06:07.04] I: Was there anything there that, so you mentioned you didn't know that maybe they were developing new ways to use it. Was there anything else that you were surprised to see?

[02:06:15.25] R: Well I didn't know how often you saw a healthcare provider, getting tested regularly, that's like a given. I didn't know like how many, I mean I knew a lot of people were using PrEP, but the facts about it. What the side effects, how long they lasted. Didn't know it could cause kidney problems in 1 out of every 3000 people. That's about it, yeah. But also, I didn't know, I mean I had heard about PEP but I didn't know that you could take that like in cases, in those cases.

[02:07:25.23] I: So in your opinion, to what extent do you think others in the LGBT community know about PrEP?

[02:07:38.12] R: I..

[02:07:41.13] I: Or know about these kind of facts about PrEP?

[02:07:43.12] R: I don't think many people know all the facts unless they're taking it. I didn't even know all the facts and I'm not taking it. But I knew I was aware of it. But I just feel like a lot of people in certain communities, especially the African American community don't have the resources, unless they're like of a certain class probably. I don't know. I just feel like all the resources aren't made available to everyone basically.

[02:08:29.07] I: Thank you for sharing that, reading through that for me. There's just two little things that I'd like to do. Shouldn't take too much time. And then I have a self administered questionnaire that you'll fill out on your own that shouldn't take more than five minutes. Okay so first up, I've written on these index cards some of the factors or characteristics that I had mentioned before. So what I'd like you to do is rank these in order of importance to you. So I believe there's seven cards here. So it's things like cost, ease of access, how often you have to take it, how you take it. And then I have some blank cards so if you think there's something else that's really important that's not included there, we can add that. So if you can't read the cursive, just let me know. I have a hard time writing in print.

[02:09:33.28] R: It's way better than my handwriting.

***Reading through the cards***

[02:09:58.19] R: I feel like the effectiveness is definitely important. A lot of people don't want to take things that aren't effective. Stigma. How often you have to take it is not that important. Well if I had to take a pill three times a day, that would. That is important. How I can take it is definitely important. Stigma, I really like, and this is specifically with the pill itself.

[02:10:39.28] I: With PrEP in general.

[02:10:42.16] R: Not important, or least important. I would want to be able to access it. And pay for it. Be able to pay for it and then access it. I mean the side effects are like a given...... Or do I care about the side effects? I mean, I've seen the side effects. But the side effects could lead to other things. I think this is what I would say.

[02:11:49.12] I: Do you think there's anything missing? Or anything that you would add that would be important for thinking about whether or not you would take PrEP?

[02:11:58.17] R: Hmm.. I can't think of anything.

[02:12:12.10] I: That's fine. Okay. So at most important, you have effectiveness. Then how you can take it. Followed by cost. Then ease of access. Then side effects. How often you have to take it. And then finally stigma at the bottom.

[02:12:31.16] R: Right cause I mean I feel like, well, actually. I mean like I know how often people take PrEP, but if it was like three times a day or like two, you know, I would want it to be really effective, you know. But then like it's kind of a 50 50 because I know it's a one a day pill, so it doesn't matter how often.

[02:13:08.19] I: So also if you think, they are trying to develop other injections or implants. So if it was something you maybe had to do once every three months, would that be more important to you now?

[02:13:20.23] R: It would. It would. I mean cause if it was an injection, I'd be shooting up pretty much or like if it was an implant that just takes care of dealing with a pill. But yeah. So I would say how often you have to take it actually.

[02:13:51.27] I: So you've added how often you have to taken in between effectiveness and how you can take it. Okay great. That's perfect then. Okay. So then the second little exercise that I have here. So I have this sheet of people. Imagine I'm going to flip a coin and depending on what you picked, either heads or tails, you'll get what's in that. So say you only can pick row one, row two, row three, row four. You can't pick two, you can only pick one. So depending on what's in those columns there, which would you pick if say you got. I don't know if I'm phrasing this or if that makes sense.

[02:14:39.05] R: So if I chose row 2 and you said or you flip the coin and it landed on heads or tails, that's what-

[02:14:50.02] I: Yeah. So say you picked row 2 and it lands on heads. You'd get 100 dollars.

[02:14:54.16] R: I don't know! *Laughs* I mean, I would probably do row 1 because I get 50 dollars regardless. But like, I like taking risks too. But I'd rather be safe than sorry. I would say row 1.

[02:15:35.06] I: Row 1?

[02:15:36.00] R: Yeah.

[02:15:37.15] I: Okay. Great. Alright so again I just want to say thank you for taking the time to do this. I really appreciate it. Before we kind of get to the self administered questionnaire and wrap up, do you have any questions? Okay great. So this is for you to fill out. If there's anything you don't want to answer, feel free not to. Your name isn't attached to this. There's a couple questions that kind of go onto the next page. So like number 7, some of the boxes start here, continue here. Some of them, I think, so like 23, the question is on the bottom of this page but then the answers aren't actually up until here. So there's that. Take all the time you need to do that. And then we'll get everything wrapped up.

***Going through the questionnaire***

[02:18:34.26] R: Does, number 9, is the, if I have the school's healthcare plan is that like commercial insurance, or would that be under other?

[02:18:49.09] I: I would say other and then just write university insurance.

***Questionnaire***

[02:26:45.14] I: Alright. Great. Thank you. Okay. So all that's left is to get you paid.

[02:26:53.17] R: That'll happen today?

[02:26:53.26] I: Yeah. I've been given cash, just so you can be done with it right now.

[02:26:59.08] R: Okay *laughs*

[02:26:59.21] I: But just as a reminder on the back of the consent form, there's the numbers for the subject advocates if at any point in the future you feel like you want all of your information taken out of this, you don't want to participate, you can reach out to us, to them. There's number for people if you want to talk to. I have a resource sheet if you're interested in learning more about PrEP or different PrEP providers. I think I was given the wrong one cause I think this is for [Upstate NY City 2]. If you'd like, I can email you one about [Upstate NY City 1].

[02:27:38.14] R: Do you, is my email address in there?

[02:27:41.19] I: I don't think so. Tim would have it, right?

[02:27:45.02] R: Yes. He has it for sure.

[02:27:46.23] I: I can ask him. And I'll send you the [Upstate NY City 1] one and it'll have different health providers in [Upstate NY City 1] that offer it or counselors if you want to talk to if you want more information about it. Alright so, can I see that pen, I should have brought more. Alright did you need any reimbursement for travel or parking?

[02:28:13.29] R: No.

[02:28:14.16] I: Okay. Great. Alright, any final comments or questions?

[02:28:24.05] R: It's been very insightful.

[02:28:25.23] I: Thank you for being so open.

[02:28:27.12] R: *Laughs* I find it's better for me to be.

[02:28:35.01] I: Do you mind if I just turn this off then?

Interview IDM 105

S1 00:56:01.938 Are there any kind of campaigns or public health advertisements in Pakistan about HIV/AIDS, or about prevention, anything?

S2 00:56:12.814 About prevention, yes. But it's again, abstinence. Abstinence, like the Mean Girls go, "Don't have sex or you'll get chlamydia and you'll die."

S1 00:56:21.703 "You'll get pregnant."

S2 00:56:21.715 "You'll get pregnant and die [laughter]." And it's crazy. It's just like that. Just abstinence only, abstinence only. And I feel like that is not how it should happen. But I think if I did get it in Pakistan, I would go talk to a doctor in the most secret of ways. But to my knowledge, there are no public health advertisements or-- public health advertisements or people that really, really say that, "Yeah. We're going to help if you have been diagnosed with HIV." Maybe I've not looked into it, but so far that I've lived in Pakistan, I've never seen that. We're working on tuberculosis, we're working on polio, we're working on yellow fever, we're working on all this, but HIV is the gray area. You're like, "It's sexually transmitted disease. Maybe you shouldn't have had sex." That's what they'd say.

S1 00:57:15.958 So some of the blame would almost be shifted onto [crosstalk]--?

S2 00:57:17.337 Of course. Of course. It's like being the rape victim. In Pakistan, also, we always say, "She shouldn't be wearing that." Usually we're very horrible people.

S1 00:57:28.664 So can you tell me a little bit about what you know of HIV transmission? So how is it spread? Or how does it move from person to person? You've mentioned a little bit, but can you just tell me a little bit more?

S2 00:57:42.325 My confusion lies in oral sex. So I don't know for sure if oral sex is how you also get HIV of it's transmitted from person to person. But I think what mainstream I've heard is, not using a condom, or if the condom breaks if you're using one and the semen or any of the bodily fluids are inside the bodily cavity. And the same happens in anal sex or vaginal sex-- what was the other one? Oh, yeah. If you use the blood of-- if the blood has not been screened properly and if you're transfused with the blood of a person who has already been infected, and lastly, sharing needles if you're using them for drugs or for injections.

S1 00:58:38.159 Right. Okay. So what can you tell me about HIV prevention? How can you go about or keep yourself from acquiring this virus?

S2 00:58:47.767 I think getting tested regularly. That's what I would think. Not using syringes that I've not seen being opened in front of me, even at the tattoo place or even at the place where they-- even at the hospital, or when you're donating blood or anything, that. Using a condom. That's what I've learned [laughter]. Using a condom at all times, making sure that both of the parties is safe and one of the parties are safe, and that's about it. That's the most I think I know about prevention.

S1 00:59:24.987 So say if you were diagnosed with HIV, what would you know about treatment options?

S2 00:59:33.787 I think the only reason I know about any treatment options is because Grinder recently introduced like, "When were you last tested, PrEP, [op?]." And then I got really interested. I was like, "What does this mean?" So I looked up and I didn't really read into it. So it was like PrEP is there and then there's PVP, [PEVP?]?

S1 00:59:52.395 There's PrEP and PEP.

S2 00:59:54.765 PEP. Oh yeah, PEP, PEP. There's PrEP and there's PEP. I don't really know the major differences between the two. I know that PrEP is when you feel that you are at a high risk because you have a partner that's been diagnosed as HIV positive. And PEP is taking an emergency situation when you feel that the condom broke. That's all I know. And I don't know if they're taken in form of pills, or if they're injected, or if they're orally, or-- yeah. I don't know how the mode of administrations for those medicine is.

S1 01:00:33.796 So do you know more about treating HIV as opposed to-- so other than PrEP or PEP, do you know anything about the medicines that people take to help prevent the spread of it or to prevent it from becoming AIDS?

S2 01:00:49.717 No. Not at all.

S1 01:00:50.944 Okay. So then, how have you found out about HIV and AIDS? I know you mentioned your classroom settings, right?

S2 01:00:57.822 Mm-hmm.

S1 01:00:58.623 You've talked about it with friends. Are there any other sources that you used to learn about it?

S2 01:01:02.908 I think the Internet--

S1 01:01:03.796 The Internet.

S2 01:01:03.937 --to a certain extent. I think menshealth.com and magazines have really, really put a very important role into it. TV ads, especially in the United States. We get Netflix in Pakistan as well. It's the US Netflix. So whenever there are ads, or anything, or TV shows, you do hear about HIV, or AIDS, or STDs. So I think that's about it.

S1 01:01:32.762 Okay. Do you talk on Grinder frequently?

S2 01:01:39.581 Mm-hmm.

S1 01:01:40.289 Have you talked about HIV or AIDS with anyone on there?

S2 01:01:43.161 No, no.

S1 01:01:44.806 Other than your boyfriend in high school, did the conversation about AIDS or HIV come up at all with--

S2 01:01:53.382 Sexual partners?

S1 01:01:54.109 Yeah.

S2 01:01:54.678 Not at all.

S1 01:01:55.498 No? Okay.

S2 01:01:56.288 Never.

S1 01:01:57.998 Okay. So let me just see what else is here. All right. So you've said that you want to start regularly using condoms, right?

S2 01:02:11.656 Yes.

S1 01:02:13.318 Are there any instances or circumstances in which you think maybe you wouldn't necessarily need to use a condom?

S2 01:02:19.546 Yeah, yeah. If there is a guy that I've been really into, and he's like-- he says like, "The condom is not-- I don't like using condom," I may think about it. I feel like my principles are not strong enough [laughter]. But then I would be like, no, no. I feel like, in that moment, I would give this a thought, but I would almost certainly come to the conclusion of leaving the person instead of not using a condom. But I don't know for sure, because I've never been tested like that. So that's there. But I think being always with a condom, or with just one single partner after having gotten tested, is the best way to go.

S1 01:03:00.758 Okay. Do you think it would be important to use condoms for oral sex as well, or would you just use it for anal sex?

S2 01:03:10.440 Only for anal sex.

S1 01:03:11.233 Only for anal? Okay. Have you ever thought that you might be infected with HIV? Has that thought kind of ever crossed your mind?

S2 01:03:19.510 Yeah. Yeah, yeah, yeah.

S1 01:03:20.052 All right. Can you tell me a little bit more about that? So when did that happen?

S2 01:03:24.174 I think it happened after coming back from the US and getting it on regularly with the neighbor. I think that's when it really started. I think it started when I read more about it in the biology textbooks and-- but I never did anything about it. I was like, "Maybe I do have STD." But I never showed any symptoms, so I'm like, "It's fine. I'm not sick. I'm never been feverish for a long time." Because the symptoms, they also-- the textbooks also describe the symptoms, you know?

S1 01:03:53.716 Sure.

S2 01:03:53.817 That you're going to feel dizzy or your white blood cells count will decline. They said that you will get infected with other infections. And time went on and I never felt like that. I never ever really felt like that. But I think that was the only time that I had a scare, but there was nothing I could do about it, so I was like, why take-- why be tense about something that I cannot fix? So that's about it. And then what me and my boyfriend did was only because we wanted to get it done and be in the clear at all times. At all times.

S1 01:04:31.265 Have you ever been diagnosed with any kind of sexually transmitted infection or disease?

S2 01:04:35.633 No.

S1 01:04:36.335 No. Have you ever worried about having one? I know you mentioned a little bit with the neighbor in Pakistan. Was that worry just about HIV exclusively, or was it about STDs in general?

S2 01:04:46.937 Just STDs. Yeah. STDs in general. So I feel like there's chlamydia, but like-- AIDS is the only one that I really know about, so I feel like it could have only been AIDS and nothing else.

S1 01:04:58.434 Sure. So in the future, moving forward, having sex with other people, how worried are you about getting HIV?

S2 01:05:07.251 I feel like that I am worried now. I am worried, having been through the orientation in this US college in [Upstate NY City 2], and just with how the LGBTQ community has made it as one of their very top priorities, about sexually transmitted diseases. I think not very, no. I think that I would never really take the chance, even if I'm very, very excited to get it on with a guy [laughter], I would never really take the chance at all. And I feel like it's been mostly because of knowing that you have to take these pills all the time, and it just becomes-- your life becomes really hard for just 15 minutes of fun.

S1 01:05:54.260 Right, sure. So then, if we had to put this on a scale. So, say we have 0 to 100. 0 is the lowest chance, never going to happen ever, and 100 is like absolute certainty, will happen, what would you say is your risk of getting HIV in the future?

S2 01:06:15.124 I'd say 40.

S1 01:06:16.958 40.

S2 01:06:17.525 I'd say around 40, 45. Because I feel like I do engage in reckless behavior, but-- I will say it's higher. I would say it's 60. Now that I think about it, I would say it's 60 because I do engage in this reckless sexual behavior often, without thinking about the consequences, just for those 15 minutes of fun. But I feel like that number will decline in the future, once I-- because I feel like now I'm confident enough to ask that person for a condom, instead of just going along with it. So I feel like I may be at risk, but I'll try my best to minimize it.

S1 01:06:56.358 All right, great. Thank you. All right, so moving into the next set of questions, they're going to be about PrEP, one of the things this study is about. So we're going to be trying to determine what you know about HIV, pre-exposure prophylaxis or PrEP. So it's a daily pill that's taken to prevent HIV, right? So in these questions, I'm going to be using the term PrEP. We've kind of used that before but it's just a lot easier to say that than pre-exposure prophylaxis.

S2 01:07:30.325 Definitely.

S1 01:07:31.180 So, for starters, can you tell me what you know about PrEP, or what you've heard about it in general? We talked a little bit about this already. You mentioned that you've heard about PrEP and PEP, but about PrEP specifically, what have you heard or what do you know?

S2 01:07:48.323 Most of it just came from seeing people being negative-- on Grindr, "What's your HIV status?" "Negative, but on PrEP." So I feel like that always just reached to a whole new audience about what PrEP was. And then you could click on the button right next to it, "What is this?" And then it gives you whole what is PrEP. What I remember is that it's taken to prevent getting-- prevent the risk of getting infected with HIV. And I've heard that you take a pill twice daily or once for 28 days. If you feel like that you're going to continue with the person who has a high risk of HIV, or you're not using a condom with a person who you don't know, that's when PrEP is taken.

S1 01:08:41.872 So who would you say then PrEP is for? Who would be the ideal user of PrEP?

S2 01:08:49.937 I would say anyone is uncertain of their partner's or partners' HIV status. That's what I would say. And unwilling to talk to them about it, or just feeling that they've been lied to.

S1 01:09:08.418 Sure. Do you think there's any kind of gendered component to that?

S2 01:09:12.181 No.

S1 01:09:12.766 No. So then PrEP would be kind of universal, right, for--?

S2 01:09:16.276 I would certainly believe that if someone from the female gender or non-binary felt in a way that they have been infected-- they might be infected with HIV, or at risk with it, they would take PrEP as well.

S1 01:09:30.968 Okay, great.

S2 01:09:32.345 They can take PrEP as well.

S1 01:09:34.323 So you said you found out about PrEP mostly through Grindr?

S2 01:09:41.288 Mm-hmm.

S1 01:09:42.097 Right. So when you click on that button that says, "What is this?" what actually pops up? I haven't been on Grindr in a while.

S2 01:09:49.866 It just pops up. It's a window, which I feel is preloaded because it never really takes time to load, it's always there. And it's more of an information of what is PrEP, who is it for, and what does this mean, the negative but on PrEP, positive but on PrEP. And then I think there's PEP as well, but I'm not sure.

S1 01:10:10.477 Does the popup window, does that include any links to learn more, or about how to get PrEP?

S2 01:10:16.788 Yeah. I don't think there's one to get PrEP. I think there's learn more. But I never really clicked on it.

S1 01:10:22.472 Is there a reason why you never went through that, or--?

S2 01:10:26.564 I think it's mostly because I just scan through it and I was like, "I understand what this is about. I feel smart already [laughter]." But then I didn't really go into the details because I felt like it didn't really concern me. And now I would because now that I know that it's a big deal, so.

S1 01:10:46.176 Sure. Have you ever talked about PrEP with friends, or with a teacher, or anyone?

S2 01:10:51.547 No.

S1 01:10:52.131 No. Have you ever talked about it with any of the guys that you've been with?

S2 01:10:56.891 No.

S1 01:10:57.561 Do you know anyone who is taking PrEP?

S2 01:11:02.600 No.

S1 01:11:03.564 All right. Any of the guys on Grindr that you've talked to, have they shown in their profile that they're taking PrEP?

S2 01:11:07.910 Yeah. Oh definitely, definitely.

S1 01:11:09.466 Has that come up in conversation?

S2 01:11:10.978 No.

S1 01:11:12.805 No. Is that something because you just aren't interested in talking about it, or it's just hard to get to that conversation, or--?

S2 01:11:20.348 I think people do talk about it. It's just that I was never-- okay, so there is this Facebook page as well it's called Grindr Aesthetics and it's crazy. It's like people post screenshots of any Grindr conversation they think is funny.

S1 01:11:35.397 And this is called Grindr Aesthetics?

S2 01:11:36.884 Grindr Aesthetics.

S1 01:11:38.025 I've never heard of this before.

S2 01:11:39.044 It's hilarious.

S1 01:11:40.788 I need to look [inaudible].

S2 01:11:41.572 It's crazy. It's a group, actually. It's a group, so Grindr Aesthetics. And people share any-- people on Grindr who have very eccentric profiles. They blur out all the faces and everything, and it's a user-submitted thingy. So they share things like if someone in their description is like, "White people only," or like, "Donald Trump," or being very wrong in some instances, or it's just a conversation where it's really funny, or wholesome, or something. So it's really funny. And then I've seen that on countless screenshots people have been talking about, "So why are you taking PrEP?" So it's normally in the instance that the person who posted the screenshot is the one whose profile says they are on PrEP and then the other guy is like, "Oh. You're taking PrEP. I would never fuck you," or something like that because I feel like that's immediate prejudice because they think that they've already been infected with HIV, although it says negative and on PrEP. So I've read those conversation many times, and it's really interesting. It's really, really interesting.

S1 01:12:56.233 So then there seems to be kind of this understanding that PrEP is associated with already being HIV positive.

S2 01:13:02.464 Yeah. Plus the LGBTQ community has a lot of biases built inside it already, and this is just another one.

S1 01:13:10.919 Do you have any feelings about people who take PrEP or any kind of ideas like that?

S2 01:13:17.479 I did not have any preconceived notions, but before I learned about it in details, before clicking on that button, I was always like, "Oh. Maybe it does mean that those people are infected with HIV." But then when I started seeing this, "Negative but on PrEP," "Negative but on PEP," then I was like, "Oh. Okay. This makes sense. This makes sense. Then these people [aren't] necessarily infected with the virus. It's just that they're being extra careful," I would say.

S1 01:13:47.600 Sure. So would your decision to sleep with someone potentially be affected by them being on PrEP?

S2 01:13:56.309 No.

S1 01:13:56.985 No. Okay. Is that something that you would want to talk to a partner about before having sex, or if you just saw on their profile, would that kind of be enough?

S2 01:14:06.783 I think that would kind of be enough, but it could be a conversation, for sure, just to get things going and everything. And I would feel like it would be-- I would never really bring it up myself because I feel like they've given enough information already--

S1 01:14:23.805 It's shared publicly.

S2 01:14:24.722 Yeah. And they're being brave about it already, so why just fidget with it even more?

S1 01:14:31.230 Sure. So if you were with someone who was taking PrEP, would you still use condoms?

S2 01:14:38.409 Yeah.

S1 01:14:41.307 Okay. Great. Have you ever been concerned that, maybe on someone's profile, they may not be honest about actually taking PrEP?

S2 01:14:50.113 No. Not at all.

S1 01:14:53.127 Do you know, is there any kind of-- on Grindr, if you were to put that setting onto your profile, is there any verification for that? Or is just anyone can put that there?

S2 01:15:04.308 I think anyone can put that there. Because when you make a new profile, it just-- it asks you, and I feel like I never had to click on it. I always said HIV status negative. And then it asks you last tested, when were you last tested? So I think that's about it. But the option always is not to put anything there. Just leave it blank and it never shows up on your profile. So I feel like a lot of people could be hiding by not putting anything over there, so I feel like it's good to have a conversation about it beforehand personally with them.

S1 01:15:36.738 Sure. Have you ever considered taking PrEP before?

S2 01:15:41.299 No.

S1 01:15:41.683 No. Is there a reason why?

S2 01:15:46.036 Because I feel like if I get a blood test done and it comes back negative, there is no reason for me to get PrEP. I feel like my insurance provider has not given me enough information about having-- how to get PrEP. And the school, especially University of [Upstate NY City 2], as transparent as they are with all of these things, they never talk to us about PrEP or PEP.

S1 01:16:09.419 That's somewhat surprising, actually. Have you seen anything around campus that mentions PrEP or PEP?

S2 01:16:16.414 Mm-mm. Even though we had our first LGBTQ meeting, the Pride meeting, and the room just-- and I feel there were around 150 people there--

S1 01:16:26.198 150? Wow.

S2 01:16:27.204 150, and most of them are freshmen. And the room was just like--could not hold it, because the fire marshal [laughter]-- so it was really interesting when nobody talked about it there, also. And there were faculty members, and they all introduced themselves, and they never-- they were always like, "Yes. You can talk to us."  But it never was on the angle-- from an angle of coming to talk to us about HIV or if you're diagnosed positive.

S1 01:16:52.916 So was that meeting held in a LGBT space as well, or--?

S2 01:16:57.463 No. So in our orientation brochure, it was like LGBTQ meet and greet, and it was [date?] and time. And we went there, where there was like around-- well, as I said, 150 to 200 people over there, and it was open to all. So I think there were some straight people there too, but most of them were freshmen students, and students of the school, and a lot of faculty members. Yeah. And even our dean of students was there. So it was just this fantastic meeting, but nobody talked about STDs, HIV.

S1 01:17:30.830 That's somewhat surprising. Do you know if there's an LGBT resource center on campus? Have you been there yet?

S2 01:17:38.105 No.

S1 01:17:38.731 No. Is that something you're planning to check out?

S2 01:17:40.818 Yeah.

S1 01:17:41.475 Okay. I'm just curious if maybe on the walls in there, they might have a flyer about--

S2 01:17:47.334 Oh. Most probably they would. Most probably they would. But I have not been there, so I don't know yet.

S1 01:17:53.113 Sure. Okay. So I'd like to ask you about some of the things that other people have told us are important when it comes to thinking about taking PrEP. So there's no right or wrong answers to any of these. And it's really fine if you don't have a response, or maybe just a few words. Totally fine. Just answer each question to the best of your ability, then. So what do you know about the cost of PrEP? You mentioned a little bit that your insurance hasn't given you a lot of information. But other than that, do you know anything about how much PrEP might cost or how you would pay for it?

S2 01:18:33.698 No. All I know is that I feel like Medicaid covers most of it. Medicaid covers it. Because of, I think, one of YouTube ads that came up, and it was like-- it talked about PrEP, but when-- before I skipped it, it was like-- it did say that Medicaid in your state might cover it to the full.

S1 01:18:55.443 If say you didn't have anything covered. Do you know how much it might cost?

S2 01:19:00.145 No. Not at all. Not at all.

S1 01:19:02.575 Have you heard about anyone that's had trouble paying for PrEP, or has had trouble getting access to it, financially?

S2 01:19:09.078 No. No, no, no.

S1 01:19:11.524 So yeah, a lot of these, you might just be saying no to, especially if you haven't talked to other people about PrEP. Would you be worried about using your health insurance to pay for PrEP?

S2 01:19:23.537 No. Yeah. Definitely not, because I feel like--

S1 01:19:27.531 Because you're the one paying for [your?]--

S2 01:19:28.545 Yeah. I feel like I'm very confident enough to know that whatever I do would be in my best interests in my health, so [that's why?].

S1 01:19:35.522 And so the insurance that you have is through the university.

S2 01:19:38.420 Yes, sir.

S1 01:19:39.446 Okay. So then, if you were to, say, go back to Pakistan in a few years, or when you're done with school, would you go back onto your parents' insurance, or would you have to find your own?

S2 01:19:49.072 I feel like I would find my own insurance at that point. Yeah.

S1 01:19:52.140 Do you think it would be possible to use that insurance to get PrEP there?

S2 01:19:56.298 That's where I really, really do research into it, and I feel like I need to find the correct one for me. And if that doesn't work out, and I do need to use PrEP, then I think I would make a quick trip back to the US.

S1 01:20:09.006 Sure. That's understandable. Okay. So what have you heard about where to go to get PrEP if you wanted to start taking it?

S1 01:20:21.057 I feel like I've heard that going to the UHS, University Health Center, would be the first step. Or this is what I feel like if I go to any of the safe spaces on campus, to the LGBTQ plus organizations, the Pride Network, I feel like I would get information from them very, very easily.

S1 01:20:39.808 Do you think it would be easy to actually start getting PrEP and taking it?

S2 01:20:44.575 No. I don't think so.

S1 01:20:45.610 Why wouldn't that be easy?

S2 01:20:47.176 Because I feel like there would be a lot of blood tests involved to get me to-- it wouldn't be as easy getting an aspirin. It's not over-the-counter medication, I'm presuming. So I feel like that I would have to have a lot of bloodwork done, talk to a lot of people, and then all would be very helpful, but I feel like it would take a long time before I get PrEP.

S1 01:21:14.602 Okay. So say you actually did get PrEP, what have you heard about taking PrEP? So like the mechanism of taking it, the frequency, what do you know about that?

S2 01:21:27.982 I have no knowledge about that. I don't know how to take it, if it's oral, or if it's rectal, or even if it causes side effects or not.

S1 01:21:38.590 Do you know if you would have to go to the doctors frequently too while you're taking PrEP? Have you heard about that, or--?

S2 01:21:45.839 No.

S1 01:21:47.051 Okay. So if it were, say, a daily pill, do you feel you would be able to take that if you wanted?

S2 01:21:55.735 Yes.

S1 01:21:56.402 Yeah. All right. I know some people just have really big resistances to swallowing pills, so. If PrEP were an injection, so say like every so often you just have to put it into your arm or something, would that be more preferable than a daily pill?

S2 01:22:13.465 I think daily pill would be more preferable than an injection because it's just like you just swallow it, right, instead of making a big deal about getting an injection, doing it, and I feel like using a pill is much more better than using a needle because it's just less scary.

S1 01:22:28.490 Sure. But if it were, say, an implant that you have it in your arm, and maybe every few months you have to get it changed out. You know how they put birth control things like you need to [crosstalk]. Would that be preferable over the pill or injection?

S2 01:22:42.854 I'd still take the pill.

S1 01:22:44.457 You'd still take the pills. Okay. Why would that be?

S2 01:22:47.707 Because I feel like the implant could potentially be a problem for me if I'm traveling back and forth in Pakistan, and then going through airport security or something like that. And then a pill is just discreet, just so discreet enough.

S1 01:23:04.159 Absolutely, yeah. So what do you know about the effectiveness of PrEP? How effective do you think it is at actually preventing the transmission of HIV?

S2 01:23:16.616 I have no idea. All I know is that it just makes sure your immune system is strong enough to work against other diseases. But I feel like that could be wrong, and that's-- so I basically know nothing.

S1 01:23:31.736 Okay. So how effective would it have to be for you to actually consider taking it? So say it only cut your risk of getting HIV by 50%, is that something you would still take?

S2 01:23:43.299 Yeah, definitely.

S1 01:23:45.540 If it only did say by 30%?

S2 01:23:48.419 I would still take it, but I would also start being really, really careful with who I'm sleeping with, what I'm doing.

S1 01:23:54.781 Sure. Is there a threshold that would be, "If it's only this effective then I'm not going to take it?"

S2 01:23:59.076 I don't think so. I think even if it's just 1% effective, it is effective. And if I'm getting it easily enough, I feel like I'll continue taking it.

S1 01:24:08.365 Okay. And do you know anything about the side effects of PrEP?

S2 01:24:12.818 No.

S1 01:24:13.405 Okay. All right, we talked a little bit about this, but do you think you being on PrEP would cause a friend or a potential partner to change their opinion of you, or feel differently about you?

S2 01:24:28.442 I think if they have had similar experience, or if they are at least knowledgeable enough on knowing what PrEP does and what it's doing, they would understand. The friends and the partner.

S1 01:24:42.690 All right. And would you feel comfortable sharing that you're on PrEP with friends?

S2 01:24:46.723 I would feel more comfortable sharing it with a partner or close friends, but just not new friends. Or even if I've had them for a long time but I feel like they might have some prejudice, I might not just share with them.

S1 01:24:58.431 Is it something that you would put onto your Grindr profile if you were--?

S2 01:25:00.912 Yeah, definitely. Definitely.

S1 01:25:04.918 So then, you said you would tell sexual partners. Or is that just something, if it came up you would talk about it?

S2 01:25:10.297 I feel like it's important to be honest, immediately. Even if it cuts your chance to have sex, but it's still being honest enough to tell them, "I am on PrEP. It doesn't mean that I have HIV. I may still be negative but I am on PrEP. I am being careful."

S1 01:25:26.206 Okay, great. Do you want to do that part? Okay. So to kind of wrap this up, there's a few exercises. But before we do that, do you have any other questions or comments about PrEP?

S2 01:25:43.794 It would be really nice to have information about PrEP as we end this. Because now there are so many questions I have [laughter], I'm like, "Oh."

S1 01:25:54.599 Okay. So this might actually be helpful. So I'm going to give you a fact sheet about PrEP. So it's just a page of information about PrEP. And you are absolutely free to take that when the interview is done. So what I'd like you to do is just take a couple minutes. Read through this. If you have questions about what something means or you're just kind of confused about something, let me know. I'll do my best to answer, but I'm not a doctor. I'm not an expert, so I'll do my best. But yeah, go ahead and read through that, and then we can talk about it.

S2 01:26:29.750 All right. Thank you.

S1 01:26:34.260 It was just great timing. You said you wanted more information and there you go.

S2 01:26:37.499 [inaudible].

[silence]

S2 01:27:44.535 This is fantastic.

S1 01:27:45.561 Really [laughter]?

S2 01:27:46.291 90% and you're like, "You started with 50 only [inaudible] [laughter]." 90%. Insurance companies, especially in New York covers most of them. It's a pill, which is fantastic. And you don't have to have had HIV to take it. It's so great [laughter].

S1 01:28:09.888 I'm really happy that you're so excited about this [laughter].

S2 01:28:12.692 It really makes me happy [inaudible]. So especially like when here where it says thousands of people have been-- tens of thousands of people have been taking PrEP worldwide, and there's only been one person to have-- that's just amazing odds. Wow. I like it [laughter]. Plus no side effects really. Headaches and nausea, but wow [inaudible]. I like it.

S1 01:28:43.132 So having kind of read through that, do you have any new questions about PrEP or does this answer just a good amount of them?

S2 01:28:49.436 I think it does answer a good amount of them. It's fantastic. I mean, it also mentions condoms and chlamydia, syphilis-- it doesn't mention what-- this question had come up. Women taking PrEP? What's up with that? Can women take PrEP as well?

S1 01:29:08.108 I believe they can. That's not something I actually know off the top of my head. But since it's designed for the prevention of HIV transmission, I don't see why it would be limited to just men.

S2 01:29:22.626 All right. And what about being when they're pregnant?

S1 01:29:25.882 That is a really good question. I don't have an answer to that right now.

S2 01:29:29.091 Not right now. That's not a problem. Don't worry about it.

S1 01:29:32.756 So then I'm not sure if on there it mentions PEP at all.

S2 01:29:36.308 It does mention PEP. It does mention PEP.

S1 01:29:38.158 Okay. So PEP is the one you take in that kind of emergency situation, right? Like you mentioned.

S2 01:29:43.083 Yeah.

S1 01:29:44.182 So having gone through this sheet, too, do you think any of this information has changed your mind about taking PrEP?

S2 01:29:53.116 I think if I do start having regular sex over in [Upstate NY City 2] and PrEP is easily available, I would start taking it. I actually would start taking it. I had no idea it was-- I don't know why they don't advertise about it more. I don't know. But I feel like it has really just changed my whole thinking about PrEP.

S1 01:30:18.953 That's great. Do you think other people in the LGBT community know about PrEP or know this information?

S2 01:30:25.800 I don't think so.

S1 01:30:27.971 Do you think if this kind of information was more publicized or shared, say these LGBT meetings, do you think that would affect people's choice?

S2 01:30:38.654 Oh, yeah. Definitely. Like it just affected my choice, I feel like my friends and the LGBTQ community, even if there was just one session of just this information, I feel like it would really, really change their mind. It would really let them know that something like this exists. And we all know there's something like PrEP, but PrEP, that's all. There's PrEP. Even I was under the guise that you have to have had HIV or maybe if you're very high risk for HIV then you take PrEP. But PrEP is just-- it's preventing HIV [inaudible]. So it's like a condom but in your body. That makes a really bad analogy, but an analogy nonetheless.

S1 01:31:21.497 Does make sense. No, it's great. Thank you for sharing your thoughts about PrEP, about everything. So we're just about at the end of the interview. Couple more exercises to do. So first, what I would like you to do-- so on these really large index cards, I have some of the stuff that people have told us are important about taking PrEP, what matters for their decision. So I want you to take a look at these. Let me know if you think something is missing because I have blank cards that we can fill up. So if there's something that, for you, is really important when it comes to taking PrEP or thinking about PrEP, we can put that there.

S2 01:32:06.395 All right.

S1 01:32:08.103 So then once you've kind of read through the cards, I think there's seven, put them in order. You can go - probably across the table might be the easiest - of what is the most important to you and what's the least important.

S2 01:32:19.911 Gotcha. Gotcha.

S1 01:32:31.629 If you can't read my writing, please [crosstalk].

S2 01:32:33.033 It's fine. It's not a problem.

[silence]

S2 01:33:27.299 I feel like ease of access and cost are-- so this is from the most important to the least important, and effectiveness, I feel like, is the most important. But even if it was just 5% effective, I would still use it, but I can certainly see when it's 90% effective I would just completely change my mind about the usage. Ease of access and cost, I feel like they're both on the same level. Then how often you have to take it, how you can take it, side effects, and stigma is on the very end, and even side effects are on the very end.

S1 01:34:06.273 Sure. Do you feel like anything is missing or there might be some other factor that would change why you would take PrEP?

S2 01:34:13.940 I was thinking about that and I feel like this covers most of it to the most of what I would think about buying a new-- about a new pill. Just [effective?]. I think it covers most of it, I would say. I think it covers most of it. Yes.

S1 01:34:35.439 Okay. Can you tell me a little bit why you put stigma at the bottom?

S2 01:34:41.190 Because stigma does not really mean that much to me [really?]. What's at stake is my health or my partner's health. Also, in PrEP at least, I feel like the drug can be taken incredibly discreetly if a person wants to be discreet. And the laws and insurance all have ensured that everything is kept quiet and everything. But even if the stigma-- if I was told to my sexual partner that I'm taking PrEP, and they leave me for it. I feel like I am a better person. Or they are a very bad person, and I'm just a better person for letting them know instead-- I could have hid it from them. And I've just gotten rid of something bad in my life.

S1 01:35:29.901 Okay. So then it's definitely fair to put it down there?

S2 01:35:32.069 Yep.

S1 01:35:32.781 Okay. Great. Okay. So the next exercise-- we can slide these away. There's so much paperwork. Everything just takes up so much space.

S2 01:35:45.606 That's fine.

S1 01:35:48.025 So on this sheet here-- let me find the right wording for this one. It's awkward to talk through. So the second exercise would go something like this. Imagine that I have a coin, and I'm going to flip it. And you're going to pick one of these rows. So depending on which row you pick, if the coin comes up a head, you would get the 50 if you picked row one. You would get 100 if you picked row two. But if it comes up tails for that row, you would only get 50, 30, 10. Unfortunately, we're not actually going to give you this money. It's just more of a hypothetical.

S2 01:36:27.970 All right. I get it.

S1 01:36:29.131 If you had to pick one of those rows, which would you pick? Does that make sense?

S2 01:36:36.061 It makes complete sense. It's a conundrum.

S1 01:36:40.750 All right. So if you take row one, you get 50 no matter what happens. But as you go up in the rows, the amount you might receive is higher.

S2 01:36:51.339 But then there's also the fact that I would not receive anything.

S1 01:36:55.051 Right, like if you went with row four.

S2 01:36:56.859 Yeah. Oh, that's so weird [laughter]. Who comes up with these?

S1 01:37:01.612 Apparently, it's a really common kind of question to ask in these studies.

S2 01:37:10.798 There's no wrong or right answer? Okay. That's--

S1 01:37:13.034 This is just personal preference. What would you pick?

S2 01:37:15.314 I think I would go with row one.

S1 01:37:16.840 Row one?

S2 01:37:17.553 I feel like the odds [laughter]-- yeah, I will go with row one--

S1 01:37:23.400 Row one.

S2 01:37:24.488 --as the statistical possibility.

S1 01:37:27.831 So can you, in a couple sentences, explain why row one?

S2 01:37:30.810 Yeah. I think row one because even if I get heads or tails, I just always have the comfort of knowing that I am getting $50. Instead of like in row four, when the coin is up in the air and I'm just dying inside. But even though I-- with 50% I win 10 times if I get heads in row four as well. But I feel like row one would be the-- row one would be the best option.

S1 01:37:58.529 Okay. Great. All right. So that's easy. So the last thing that I have for you to do is I have this short questionnaire. So it's really just some kind demographic questions. Just some stuff about you. If you don't feel like answering, you don't want to, you can just skip over it. Your name isn't going on this. We just need some kind of data to look at later. So there's that. Here's a pen. This survey should take maybe about five minutes or so.

S2 01:38:33.776 Don't worry about-- I'll try this out?

S1 01:38:35.684 Yeah. Go ahead. If you have any questions or want clarification, let me know. Again, skip over whatever you want.

S2 01:38:42.704 Thank you.

[silence]

S1 01:42:10.438 All set?

S2 01:42:11.013 Yes.

S1 01:42:11.119 Okay, great. So just a couple of formalities. Just a reminder, in your consent form you do have phone numbers if you want to reach out to anyone, talking about people in the study, or people who are involved with the study, or you have the subject advocates. If you just don't feel comfortable for whatever reason, you also can contact the IRB, the human rights review board. You have my email. So if you want to reach out to me, if you have more questions, if you want copies of the fact sheets so you can share with people, that's totally fine. You can reach out to me at any time. I'm usually pretty good at responding to emails [laughter]. Sometimes it might take me a day, but--

S2 01:42:52.027 That's fine, no problem. No problem at all.

S1 01:42:54.287 So you have your fact sheet. You have your consent form. I also want to give you this. So it's a list of providers in the [Upstate NY City 2] area. So if you are interested in getting PrEP, these are some of the places you can go other than UHS. Also, I know sometimes talking about these things can be difficult or upsetting, so if you do need counseling help, that's down here as well. There's no obligation for you to talk to any of these people at all. But it's just a reference for you if you feel that you want it or need it.

S2 01:43:27.028 Makes sense, okay.

S1 01:43:28.851 Okay.

S2 01:43:29.048 There are a lot of PrEP providers here.

S1 01:43:30.664 Right? Yeah, especially in the [Upstate NY City 2] area, there's a good amount.

S2 01:43:33.704 Wow, there's six.

S1 01:43:36.818 I've heard good things about [Health Center Upstate City 2]. So, if you wanted to, they might be one of the ones I would go to first.

S2 01:43:42.810 Which ones? This one?

S1 01:43:44.683 [Health Center Upstate City 2], the top one.

S2 01:43:45.697 Oh, [Health Center Upstate City 2]. Oh, okay.

S1 01:43:47.525 But again, you do have the University Health Services here.

S2 01:43:50.446 Definitely, definitely.

S1 01:43:51.498 That could be a great resource for you.

S2 01:43:53.766 Okay.

S1 01:43:54.818 So then, all that's left is to get you paid. So did you need any reimbursement for parking or for travel?

S2 01:44:07.546 I did travel. It was $7, I think.

S1 01:44:10.435 Okay.

S2 01:44:11.290 Do you need to see the receipt?

S1 01:44:13.093 Yeah, would you mind?

S2 01:44:14.119 Yeah.

S1 01:44:14.933 Sorry. Just [a formality?].

S2 01:44:16.364 No, don't worry about it.

S1 01:44:18.009 Okay. I'm just going to steal the pen back.

S2 01:44:32.284 Just trying to get Internet here.

S1 01:44:37.649 If you can't pull it up it's not the end of the world.

S2 01:44:39.489 Yeah, it's no problem.

S1 01:44:41.524 Okay. So if you want-- I'll fill out the [paper?]. If you just put your first and then last name. Okay. So then this is just saying that you've done the written consent, that you've been offered the copy of it, the amount of reimbursement for your time, and then the amount for your travel.

S2 01:45:10.100 Perfect.

S1 01:45:10.909 Okay. And then, so I know you have received this amount of money. All right. So again, I just need you to print your name. You're going to sign and then put the date. And that's just for budgetary things, saying that you've actually received this money.

S2 01:45:29.829 So here?

S1 01:45:30.371 Yep.

S2 01:45:30.499 Printed?

S1 01:45:38.337 You said you took an Uber here, right?

S2 01:45:40.039 Yeah. And today is--

S1 01:45:42.253 The 2nd.

S2 01:45:42.549 --the 2nd of September.

S1 01:45:44.091 Yeah, it's been a little while since my last interview.

S2 01:45:50.039 Here you go. Past [inaudible] trips. Past [inaudible]. It was from my place to-- it has dropped me off at [Corner?] House but then he came back and he dropped me over here.

S1 01:46:08.175 All right, perfect. That's fine. All right. So there's [inaudible] for participating in the study. And then that's the travel reimbursement. So, okay. Do you have any other questions, any comments? If you think maybe in half an hour you're going to get home and just kick yourself thinking, "Damn, I wish I'd brought this up."

S2 01:46:28.325 Yeah. I don't think so. I really don't think so. If I do, then I certainly have your phone number-- your email address. And as usual, have the internet, and then all these numbers on the fact sheets, that's all good.

S1 01:46:40.055 If you want my phone number, you can have that as well.

S2 01:46:43.708 I think I just email you if there's any problems.

S1 01:46:45.886 Sure.

S2 01:46:46.534 Because I don't think there will be any problem.

S1 01:46:48.119 Okay.

S2 01:46:48.937 That would be good.

S1 01:46:49.356 All right, so I'm going to go ahead and turn this off.

IDM 106

kay. Um, how about some of the ways to prevent of HIV?

Participant: Um, I only know safe sex, and then, um, PrEP now. But that's it. I don't know of any other.

Interviewer : Do you know about the effectiveness of either of those?

Participant: Um, condoms are 99% effective, of 98 I think it was. PrEP, no.

Interviewer : Okay. So say if you were to acquire HIV. Do you know anything about treatment options that are available?

Participant: No. I would have to go definitely talk to the doctor for that one.

Interviewer : Um, so basically then the way you found out about HIV and AIDS is through classes or ... like, have your parents told you new information, or more just "be careful" kind of stuff?

Participant: They, yeah. It's more just be careful kind of stuff. It's kind of like the stuff you hear, the typical "HIV's bad. Gays get AIDS." That's about it. Not anything else.

Interviewer : But so for the stuff you have learned about it, that came from-

Participant: Yeah, that came mostly from my classes and um ...

Interviewer : Have you ever seen anything in the forms of media about HIV or AIDS? Like TV shows, movies, whatever.

Participant: Uh, I've seen movies, but just because we were assigned to them in class, in my Queer Looks class. But aside from that, not that I can think of.

Interviewer : Do you remember what some of those movies were off the top of your head?

Participant: I don't remember the name, um, I do know the movie was definitely about two ... I can't remember if one or both of the guys had HIV. I think it was only one. Uh, but it kind of just like shows like him at the beginning just like living his life, how he has AIDS, and HIV, this was before, this was like way before trea-, before there was actually treatment to prevent it from turning into AIDS ... and then after that he just kind of like got AIDS, and you could completely see his whole body sort of changing. Um, and then at the end he had like spots everywhere. He was just like in pain. And then it was just like a completely different person. It looked like a skeleton, but yet he was like still alive.

Um, and then he ended up dying in the documentary you can, like you actually see his completely dead corpse. Like you see the police come in, and like, not the police, the paramedics like wrap him up and things like that. So that's about as real, as much as I've seen in that sense. But that movie I feel, as far as I know, captured like as realistic as you can get to how it can get.

Interviewer : And that was a documentary?

Participant: It was, yeah, it was a documentary that we got, um ... not that, no, it wasn't a documentary. It was just like ... I don't know what you would call it. It was, it wasn't a documentary of like they were interviewing them, um ... um, it was like a movie of them like speaking about it.

Interviewer : Sure. Okay.

Participant: So I don't know what you would call that.

Interviewer : Um, I feel like a documentary might still like be a good term for it, but ... um, but so that's like the extent that you've seen of like-

Participant: Mm-hmm (affirmative).

Interviewer : Okay. Um, so kind of going back a little bit to sexual experience, um, can you tell me a bit about condom usage? So like how regularly do you use them? If you use them for some types of sex and not others? Um, or if there's cases where you don't use condoms?

Participant: So, I don't use condoms for oral, but I have, have used condoms every single time I've had sex, whether I'm topping or bottoming.

Interviewer : And with a man or woman?

Participant: Yes.

Interviewer : Um, is there a reason why you don't use them for oral?

Participant: No, not really (laughs). I know it's not a good thing, but like, it just doesn't happen.

Interviewer : I'm not trying to judge you-

Participant: No, I get it.

Interviewer : -I'm just trying to see if there might be like a reason.

Participant: No, there's no specific reason. Just, I don't know, just a flavored piece of plastic ... yeah.

Interviewer : Um, do you think there, there might be a time when you wouldn't use a condom with someone?

Participant: I thought about it actually. I thought about it with my ex. Um, when him and I first started, um, I actually, we both went to get tested first thing, because I wanted, if I was going to be in a relationship I wanted to be clean with them.

Interviewer : Sure.

Participant: Um, towards the end of our relationship I was kind of curious, just because I was like, I wonder like, like with him, I trusted him, so I was just like, even if this is like the only time I've ever tried it before, like I got married, or like in a very, very serious relationship, um, I would've been willing. But besides that I think in order for me to have sex with someone without a condom it would have to be, like, I, it would have to be something extremely serious. Like I would have to know, like, that they really care about me a lot to the point where I can trust them, that I know they're not going to be with somebody else. But then again you still never know.

Interviewer : But that's what trust is all about, right?

Participant: Yeah (sighs).

Interviewer : Um, have any of your partners ever brought up the idea of not using a condom?

Participant: No.

Interviewer : No? Okay. All right. Um, so have you ever thought that you might be affected with HIV or you might have acquired it somehow?

Participant: Oh, no.

Interviewer : Okay. How about any other kind of like STDs or STIs? Have you ever just been worried that like maybe you have one?

Participant: Not ... from seeing my own things, no. Um, maybe just like, after, after the club on Saturday, because I got strep throat like the next day, that kind of worried me but then like I've gotten strep throat in the past, so like my immediate reaction was just like, "Oh my god, what if I've got something." But then after that I was like, "I've gotten this in the past. It's not something new." So I already recognize the symptoms, and the symptoms kind of played out. So that's about really the only time, um, yeah.

Interviewer : Sure. Um, so you mention you had gotten tested with the boyfriend that you were with. Um, how frequently do you get tested for STDs or HIV?

Participant: So I ... so I'm not, I wasn't sex-, sexually active before this year.

Interviewer : Mm-hmm (affirmative).

Participant: Um, so last time I got tested before him I think was last, it was the last time I went to the doctor's. So it was like end of December, beginning of January. And then I got tested with him again in, um, in like March, April. And then before that it was like not this summer, but the previous summer. So about every six months.

Interviewer : Okay. Um, and have you ever been, have you ever tested positive for any STDs?

Participant: No.

Interviewer : No. So in the future, how worried are you about actually getting HIV?

Participant: Um, I feel like I'm always going to be worried. It's, I don't feel as worried, because most, because mostly the people that I've talked to isn't some just like person that I meet, or that I've seen one time and then just never see again. It, it usually, besides like the guy that I fooled around with like the first day of school, after that anybody I've ever usually fooled around like hung out with. I have people that I either know or still talk to, or things like ... or knew before, and then hooked up. So it's not something that really concerns me as much. Because I don't usually go hooking up with random people like that.

Interviewer : But so you are still-

Participant: But yeah, I will always be worried (laughs).

Interviewer : So can you explain a little bit about why that is?

Participant: I feel like just because nowadays it's all so kind of ... you also don't know how many people are really telling you the truth, even if they say that they are. So yeah, that's why I am.

Interviewer : Sure.

Participant: And also a lot of people may say that they don't, but they may not know it, so.

Interviewer : Sure. Especially if they're not getting tested regularly.

Participant: Exactly.

Interviewer : Um, so, so taking that kind of response, so on a scale of like zero to 100, where zero is lowest chance, never going to happen, 100 is the highest chance, absolute certainty, what would you say your risk for getting HIV is?

Participant: Um, I would put it between like a 10 and a 20. Just because ...

Interviewer : Mm-hmm (affirmative). So it's still there, but very low.

Participant: Yeah, it's, yeah. It will always be there. It will be very low, um, but, I don't think that ... I don't think I go out enough in that sense to have it be like very high.

Interviewer : Sure. Um, have you had conversations about HIV or other, um, sexually transmitted diseases or infections?

Participant: So I've never had, I would never ask for like specific things, um, but usually I do end up hooking up with someone, before it even happens I do make sure to ask like, like have you gotten tested, like, if you have when was the last time, like are you clean? And things like that.

Interviewer : Sure.

Participant: So I do ask them about it. And if they end up lying ...

Interviewer : Right. Have you ever been worried that they might have lied, or?

Participant: Um, I'm never, I'm not too worried, um, that they might've lied, but I feel like there will always be like a small doubt, just that you never know. I feel like it was ... recently, because all the people I have been with I still talk to them afterwards and things like that, the, that wariness really isn't there. But if I were to be with someone and I after I just completely never see them again I will probably have that doubt.

Interviewer : Sure. Um, and that's totally understandable too, right? If they just disappear after.

Participant: Yeah, and then I'm like ...

Interviewer : Um, so if, um, if someone were to tell you, like a person like you might be planning to hook up with, um, if they were to mention that they were HIV positive do you think that would change-

Participant: Yes.

Interviewer : Really? Why is that? Just because worried about contracting it, or?

Participant: Worried about contracting it, just because I don't really want to run just the possibility of having it at that point.

Interviewer : Mm-hmm (affirmative). Sure. So I know on Grindr now you can actually put on your profile whether or not you're HIV-

Participant: Yeah.

Interviewer : -negative, positive, when you've been tested. So if you were to see that someone says like "positive but undetectable," would that still kind of affect talking to them?

Participant: Yeah.

Interviewer : Okay. No it's, again, I'm not trying to judge you.

Participant: No, I completely get it. No, for me in that sense it's something I'd rather not risk.

Interviewer : So even, so say someone was like undetectable and you have condoms available. Is that still-

Participant: For me, yes.

Interviewer : Okay. Well that's, it's understandable and fine. Um, I'm just trying to get like, a little bit about how you think of HIV, with other people, with yourself.

Participant: Yeah, no, the way I see it is I've personally thought, even though I say no, I still find it kind of messed up, just because like ... maybe, I don't know how they got it and things like that. But it, it's not something I would want to run a risk for.

Interviewer : Sure.

Participant: So.

Interviewer : No, I understand. All right. So the next kind of set of questions that we're going to move into, um, are about, uh, HIV pre-exposure prophylaxis, or PrEP for short. Um, so in these, in the questions that are coming I'm going to be using PrEP to refer to that, because it's a lot easier to say that the pre-exposure prophylaxis. Um, so to begin with can you just tell me, if anything, what you've heard about PrEP?

Participant: All I know about PrEP is that helps in the prevention of getting HIV. That's it.

Interviewer : Do you know who PrEP is for? Like who can take it?

Participant: Um, I was actually was talking to my friend about this yesterday.

Interviewer : Really?

Participant: Um, because he's the same one that ... basically I talk about like HIV and AIDS and all that. Um, he was actually telling me about PrEP is actually, um, that anybody can take it. It's more advertised in the gay community, because of guys, but he says that really anybody can take it. Um, what's it called, it's even helpful in terms of like if you have a partner that's like HIV but it's undetectable, it's easier if the other person ... like if they use precautions, but they also do PrEP so that way there's really a very, very minimal risk of them getting it and all that.

Interviewer : Mm-hmm (affirmative).

Participant: So in terms of that, that's about as much as like ...

Interviewer : Sure. So how have you found out about PrEP? I know you just mentioned that your friend, yesterday you were talking about this. Um, I think you said in your Queer Looks class?

Participant: Yeah, my professor mentioned it and I didn't even know it was a thing.

Interviewer : So had, so you hadn't heard of it before that class?

Participant: No.

Interviewer : Okay. Is that something that's ever come up with your parents in conversation, or?

Participant: No, I don't even know if they know.

Interviewer : Um, and when getting tested or going to a doctor have they ever asked about it or told you about it.

Participant: Mm-hmm (affirmative).

Interviewer : Okay. Have you talked about it with other friends than this one person, or?

Participant: Um, no. Surprisingly no. Is PrEP a universal thing. Like can you get it in any place? Whether it's here in [Upstate NY City 2], or over in LA, or like-

Interviewer : Yep.

Participant: Wow. That's amazing. I've barely heard, I've only heard about it hear.

Interviewer : So have you seen anything about PrEP like in media, like movies, TV shows, um, games, anything?

Participant: No.

Interviewer : No, okay. So some of these answers might just be no then. But do you know anyone who's taking PrEP right now?

Participant: My friend.

Interviewer : Oh, he is.

Participant: Yeah, he's on PrEP. I, now, I have ... I know two for sure. I can't think of the third for sure is taking it. But I know two of them that are for sure taking it.

Interviewer : And, but you've only talked about friend with the one.

Participant: Only with one, the other one like briefly, like not really go into detail. He was just like, "Yeah," because I asked him like, "Oh, you're on PrEP?" And he was like, "Yeah." That's about it. But like didn't really go into detail about it.

Interviewer : Um, have, have either of them told you about experiences with PrEP or what it's like to be on it, or anything about it?

Participant: Yesterday I was talking about it. Because I was actually curious about that. Because my family doesn't really take medicine.

Interviewer : Sure.

Participant: So I'm the one that takes the most out of the family, and I take, and I rarely ever take medicine. So I actually asked him, because I was like, "What really do you get? Like what are the negatives, aside from positives obviously?" And he told me, "It's just a tablet you take a day. Um, it's a tablet you take every day. You don't have to, but obviously it's more effective if you take, take it every single day."

Interviewer : Mm-hmm (affirmative).

Participant: Um, and that the, one of the negatives is that as time progresses, um, you lose muscle or something. I forget what, I forgot what it was. But you lose something with your muscles. Muscle, muscle, muscle deficiency, that's what it was. But then that's as far as I know. As far as I know he doesn't have any issues or anything.

Interviewer : Mm-hmm (affirmative). Um, have you ever thought about taking PrEP?

Participant: I've thought about it. Um, I've thought about it, but as of right now, because I'm not sexually active, um, I don't think it's something that I want to put my body through unless I'm actually like ... if it's actually worth going through it.

Interviewer : So are there other factors, um, affecting that kind of decision process other than being sexually active?

Participant: Um, for me to actually take it?

Interviewer : Yeah.

Participant: Um, just on a, just the idea ... again, since I don't really take medicine, I guess if I don't really take medicine I don't see the point of me taking, taking PrEP right now if I'm also not really using it for technically what it's, what most people take it for.

Interviewer : Sure.

Participant: Because the people I do know that are taking it are 10 times for sex-, sexually active than I am. So for me that makes sense. Um, for me it wouldn't make sense as to really take it right now, with I don't hook up with random people like they do.

Interviewer : Right. Okay. Um, that, that makes sense, too. Because it's kind of been advertised as like, if you're sexually active this is something that's good.

Participant: Yeah.

Interviewer : Um, so uh, I want to ask about some of the things that other people have told us are important when it comes to taking PrEP, or why they've considered using it or not. So there's no right or wrong answer to any of these, um, and it's fine if you don't have anything to say or don't know anything about it. Just try to answer, you know, to the best of your ability. So what do you know about the cost of PrEP?

Participant: Cost, um, is, uh, my friend, I asked him once also ... it wasn't recently. Um, I think what he told me was that after insurance I think for him it was like 25. I could be wrong. Um, that's as far as I know. What we told me, it wasn't really an expensive thing to take. I don't know how much he got, like how much time span, basically, or for how many days. Um, but as far as I know it wasn't really that expensive.

Interviewer : Um, have you heard about, other than your friend, like people have issues affording PrEP?

Participant: No.

Interviewer : Okay. Um, do you know if your insurance covers the cost of PrEP?

Participant: Um, no. I don't know that.

Interviewer : Um, if it did, um, would you be worried about using your health insurance to pay for it?

Participant: Oh, absolutely not. (laughs)

Interviewer : So do you have ... I guess what I'm trying to ask is do you have your own health insurance like through the university, or are you on your parents'?

Participant: I'm on my parents', but if I were to tell my parents like, "I want to take it," whatever, I know it'd be an issue, not because of like the sex thing, but like because it's uh, it's a pill. And they're very off pills and things like that. But it'd be something that I would just have to talk to them about. But it wouldn't be something that I would be scared to talk to them about.

Interviewer : Sure. Um, so then for you, if it came out to that cost, let's say like $25 for a month's prescription. Would that be affordable for you, or is that too expensive?

Participant: No, I think that's perfectly ... that's more than a right amount.

Interviewer : Um, if you wanted to get PrEP, um, do you know where you would go, or have you heard about places you can go to, to get it?

Participant: I think, um, can't you get it at the [[Health Center Upstate City 2] 01:30:43] health center here? I think that's where I ... that's where I got tested. I think that's one of the places where I think I saw it get, um, advertised.

Interviewer : Mm-hmm (affirmative).

Participant: But yeah.

Interviewer : So then do you think it's necessar-, um, easy to get on PrEP?

Participant: I think it's easy. I think you just have to seek it out.

Interviewer : Sure. Um, okay, so what have you heard about taking PrEP? I know you mentioned your friend talked to you a little about this. So, um, just to revisit it, how often do you have to take it, what form is it in, that kind of thing.

Participant: Um, I think it's a blue pill. And as far as I know it's just, you take it like a regular pill once a day.

Interviewer : And do you know how often you have to go to the doctor's office when you're on PrEP?

Participant: Um, he told me this also. Um, I think it was ... is it once every two or three months?

Interviewer : I believe so.

Participant: Okay. Wow, I can't believe I got that right. I can't even remember all that.

Interviewer : Um, all right. I have some information about PrEP though, I'll give to you at the end, too. Even if I can't answer some of your questions, it's on the sheet. Um, so I, you mentioned that your family doesn't really take medicine. Is that specific to all types of medicine, or just pills?

Participant: Everything.

Interviewer : Everything.

Participant: Um, they're more of go natural type thing.

Interviewer : Sure. Do you, if, um, if you were on PrEP do you think you would be able to take a pill everyday? Or would that be a problem?

Participant: Oh no, that'd be easy.

Interviewer : I know some people have just this complete aversion to swallowing pills.

Participant: I used to. Not anymore. Now I'm just like, time to start my day. (laughing)

Interviewer : If PrEP were an injection would that be preferable to a daily pill?

Participant: Absolutely not.

Interviewer : No?

Participant: No. (laughing)

Interviewer : So I'm guessing a needle issue there?

Participant: No, uh-uh (negative). Especially having me to do it, absolutely not.

Interviewer : How about if it were like an implant that you had like under your arm here, and it got, you know, redone every few months?

Participant: No, that, ugh, oh god. No, because I know there's a birth control like that and even that makes me uncomfortable.

Interviewer : So then for you the kind of the best option would be the pill.

Participant: The pill.

Interviewer : Okay. I'd asked this a little bit earlier in the interview, but um, what have you heard about the effectiveness of PrEP?

Participant: Nothing. In terms of that sense I don't know the probability, or how much it prevents it. I know nothing. All I know is that it prevents it.

Interviewer : So, um, do you think it could be effective, or how effective do you think it would be?

Participant: Um, for the amount of people that I've seen on Grindr that say, like, "Negative but on PrEP" and things like that I would say that it would tend, it tends to be, like, between moderate to highly effective.

Interviewer : Sure. Um, so how effective would PrEP have to be for you in order to consider taking it? So say, you know, the medicine issue wasn't there, you didn't have to worry about whether or not you're sexually active, but if you're on it how effective would it need to be?

Participant: At least like ... I want to say like 85% at least.

Interviewer : Okay. So it would have to reduce your chance of getting it by 85% to consider taking it.

Participant: Between like 80, yeah, starting at like 80.

Interviewer : Okay. Um, so you mentioned a little bit about the, um, the side effects that your friend had mentioned to you.

Participant: Mm-hmm (affirmative).

Interviewer : Um, were there any that were particularly concerning to you when hearing about that, or?

Participant: Since he just mentioned, um, bone deficiency, that one a little bit, just because again, I have a very small figure all ready. So I don't know if that would be like the best thing for me.

Interviewer : Sure.

Participant: So yeah.

Interviewer : Um, have you heard about any other side effects associated with taking the drug, or?

Participant: No.

Interviewer : Okay. Um, and then do you think friends or potential partners would look favorably on taking PrEP?

Participant: Um, some friend-, hm, I, no, I don't know. See, I don't know, because a lot of them ... I know that the ones that are tend to be ... no, actually no. I wouldn't say that most of my friends would be willing to, just because they'd be like too lazy to go out and get it, or like having to go to the doctor every three months and all that.

Interviewer : Sure. Um, but so then, if you were on PrEP how would your friends kind of react to that if you told them about it?

Participant: I think they would just be ... well, my friends who are on it would be like, "Yes!" Like, "Get on the train!"

Interviewer : Right. (laughs)

Participant: But the other ones I think would just be like, "Oh, cool. Good for you." Or like, "Oh, I didn't know that PrEP was even a thing." Things like that.

Interviewer : Um, do you think your parents would have an opinion on it, or?

Participant: I think they would have an opinion on it just because it's a medication I'd be taking, kind of like birth control ... I see it as like the equivalence of like a birth control, but like in terms of HIV that anybody can take. Um, so because they're not all fans of like medicine.

Interviewer : Um, but so not necessarily because it's advertised for men who have sex with men, or that kind of thing?

Participant: I think if I, if people did know that I was on PrEP I think the very first thing that would go to their brains, if they didn't know what it was already, would be that I'm extremely sexually active and things like that, so.

Interviewer : Would that bother you?

Participant: No, because my sex life isn't like other people's, like ... if it bothers them, it bothers them. I'm not going to stop living my life whether it's good or bad, like for other people.

Interviewer : Sure. Um, so if you were on PrEP would you tell sexual partners then, or?

Participant: Yes, I would.

Interviewer : Um, and would you expect them to tell you if they were using it?

Participant: No.

Interviewer : Okay.

Participant: I wouldn't expect for ... oh, if it ... partners as in someone, you know, just anybody that I'm hooking up with?

Interviewer : Mm-hmm (affirmative).

Participant: Um, I wouldn't expect them to tell me, just because it's not something that I would expect a lot of people to kind of just like tell people, even though they're hooking up. For me it's something that I'd kind of just like would like to just get out, like to say, be like, because for me if I hear that somebody's like, "Oh, I'm on PrEP," then chances are that that person, I see that person as more like, they do tend to focus a lot on that they're clean and things like that.

Interviewer : Mm-hmm (affirmative). So kind of branching off of that, how would you feel if, say you were going to go hook up with someone and they mention that they were taking PrEP?

Participant: I would think, "Oh, this person probably hooks up with multiple people, but also focuses, but also like focuses on staying clean." Because chances are I'd talk to them about being clean, and if they told me that they were clean or that they already got tested and things like that, and then I found out that they were on PrEP then it would kind of just be like, um, an enforcer at that point, uh, like, yeah they do take care of themselves.

Interviewer : Do you think it would make a difference, say, say you were dating someone, um, and like two months in they tell you that they want to go on PrEP. Do you think that would make you feel differently than just like some casual partner saying, "Oh, by the way I'm on PrEP?"

Participant: Yes, in a way, yes, because I'd be like, "Why are you trying to go on PrEP when we're only seeing each other and you already know I'm clean?"

Interviewer : Sure.

Participant: So that would be my concern.

Interviewer : Um, have you talked to people on Grindr that have on their profile say that they are using PrEP? If you've paid attention.

Participant: Um, I've paid attention but I don't think it's something that I've like, memorized, like if I did end up messaging them or not. I'm pretty sure though that I ... not, I haven't, there's only one person on Grindr, no, two people I've hooked up with from Grindr. One of them I think he was on PrEP. But I didn't, we didn't have sex.

Interviewer : Has PrEP ever come up in conversation on Grindr at all, or?

Participant: No.

Interviewer : Okay. So um, all right. So I have here, I mentioned a few minutes ago, um, this sheet about PrEP. So it's just some facts that have been put together about the drug itself. Um, and you are more than welcome to take it with you, um, when the interview is done. So I'd like you to just take a couple minutes to read through it. It's just the one side. If you have questions about what something means let me know and I'll do my best to, uh, to explain it.

Participant: Mm-hmm (affirmative). All right. That was all very interesting.

Interviewer : Great. Um, so having read through that, um, do you have any sort of new questions or thoughts about PrEP?

Participant: Uh.

Interviewer : Or was there anything that was kind of surprising to you, or that you didn't know?

Participant: Um, the part that I was surprised at was just that only one person has been to known it, and it happened to be a very rare strain of HIV. So but besides that, um, what kind of like kidney problems ... because that does seem to be like very low in terms of like, one in 3,000 people have kidney problems.

Interviewer : Mm-hmm (affirmative).

Participant: So what exactly problems do they end up having from that?

Interviewer : That's not something I know. I could ask some of the people in charge of like the project, and I can forward you some more information if you're interested.

Participant: I'd be interested in that.

Interviewer : Sure.

Participant: But uh, yeah, then aside from that, um, the headaches, nausea, like taking it, I feel like that's very common when you start taking a brand new medication. You're body's just adapting to it. Um, but besides that ... it definitely seems like it's a good idea. (laughs)

Interviewer : So, um, so I think we're at that. Do you feel a little bit differently about PrEP now? Or has your changing about it changed? Or, have your thoughts about the drug changed?

Participant: I mean, I feel like it, I do see it as something I could possibly take in the future, but it would definitely have to be something that I would like sit down and really consider before actually deciding to sign up for it.

Interviewer : If you were, say a long term relationship with a woman, do you think that this would be something that you'd be interested in taking?

Participant: Um, if she doesn't HIV as far as I know then there's no point for me.

Interviewer : Okay. And so to what extent do you think others in the LGBT community or people in general know, um, some of this information about PrEP?

Participant: I feel like the people that know about PrEP are really like ... like you may see the ads, but I feel like a lot of people just kind of look at them, and just kind of go like, "Oh, it's another HIV STD prevention thing." That's it. But I feel like most of the people that are fully active, um, sexually active in the community are the ones that are actually on this, and like fully know about it and things like that.

Interviewer : Sure.

Participant: Like I said, most of my friends that aren't fully sexually active don't actually know much about this, but the ones that are, um, very sexually active, they're the ones that are on this, and know most of the information on it.

Interviewer : Um, do you think if, um, people knew more of this sort of information or some of these facts about PrEP, do you think it would change, um, decisions about taking it?

Participant: I think more people would take it if they knew more of these facts. I think this is actually something that should be taught, like, even in the high school. Like they shouldn't, I, like this is more focused on the LGBT community, but I feel like it should be just like a general thing.

Interviewer : Right, especially since it's something that almost anyone could take.

Participant: Yeah.

Interviewer : Or get use from, so. I agree. Um, so thank you for going through that, for sharing a bunch of stuff with me. Um, we, I promise we're almost done. (laughs)

Participant: It's fine.

Interviewer : Um, there's just a couple little exercises that I want to have you do. Um, so it's a little different than just the talking that we've been doing. Um, so the first thing is on these oversized index cards I've written out some of those factors that, um, I ask questions about, that people have said "These are important to us," when we're thinking about taking PrEP. Um, so I want you to take a look through there, um, I think there's seven. Um, and so maybe stuff that I asked you like about the cost, about the effectiveness, about how you take it. Um, and so I want you to tell me if there's something missing from this pile. It's not a trick question.

Participant: Yeah.

Interviewer : It's more if you feel like something else should be there. Um, I have other cards that we can actually right on as well, so. If you can't read your handwriting, please just-

Participant: No, your handwriting actually reminds me a lot of mine.

Interviewer : (laughs).

Participant: Um. I'm thinking of a word. It's very related to ease of access. Instead of ease of access, well besides ease of access I'd say something like, um, just like ... actually putting it out there. Like literally having people talk about it.

Interviewer : So kind of like, would awareness be it?

Participant: Awareness. That's the word I was looking for.

Interviewer : Okay. Do you think there's anything else that could be added to the pile.

Participant: No, one of the things, one of the word things was like [inaudible 01:46:35], so.

Interviewer : Okay. So if you were to take these cards, including the one that we've just introduced, um, can you put them in order like across the table or this where, where the most important is on one side going down to the least important. So effectively ranking them for you.

Participant: So for me ... um. Personally, I don't care what people really have to say. (laughing). Uh. I'll say there. Oh, this is hard.

Interviewer : (laughs). Um, you can potentially put them in the same place if you think they're of equal importance.

Participant: Um, I don't think you have to take it ... I don't, I don't think that one's as important.

Interviewer : Sure.

Participant: Awareness I think would match up more with those. Ease of access goes over here. This one I'll say is like in the middle.

Interviewer : Okay.

Participant: Unless, well, I mean ... if we're not considering injection, if we're saying it's just the pill as it is right now then I don't really care.

Interviewer : Okay.

Participant: So I'm going to say that, because I don't care. I'm just going with what it is already.

Interviewer : Sure.

Participant: I think cost would be like right there. Yeah, that's about a tie.

Interviewer : Okay.

Participant: Um, I'd say it would be like, [inaudible 01:48:27]

Interviewer : Okay, so going from least effective to most effective.

Participant: Yeah.

Interviewer : Okay, so least at the bottom we have how you can take it.

Participant: Yeah.

Interviewer : Then stigma, then how often, then awareness of it, and then ease of access is equal to cost.

Participant: Yeah, that's what I would say.

Interviewer : Okay. And then the most important is both effectiveness and side effects.

Participant: Yeah.

Interviewer : Okay. Great. So, all right. Because when we're transcribing the interview later we won't have the cards like in front of us, so it's important to, to verbalize it. Um, so is there a reason why you put, uh, the effectiveness and the side effects as equal to each other?

Participant: Because to me, um, if I'm going, if I could possibly be messing up my body, my body's in some way, but protect, I have to know that how well it's going to be effective.

Interviewer : Sure. Okay. Um, and how about for ease of access and cost? Is there a reason why they're equal to each other?

Participant: Because I feel like in terms of that, one's effectiveness and side effects at that point, clearly I'm already interested in the product, and I want to get on it at that point. So is it really worth, is it near me? Can I get it? And then with that comes can I afford it if it's near me?

Interviewer : Sure, okay.

Participant: Because if it's, if there's not that much ease of access chances are it's going to be a lot more expensive.

Interviewer : Right. Great. Okay. So we can put these away then. Um, another small exercise. Um, so I have here this sheet, um. So this is kind of like a hypothetical thought kind of experiment. So imagine that I'm going to flip a coin. And you're going to pick one of these four rows. So depending on how the coin lands you would get $200 if you picked row three and it lands on heads. But if it lands on tails you'll only get $10.

Participant: Yeah.

Interviewer : Unfortunately we're not actually going to do this for real, so you don't get that money, but if this were actually going to happen, is there a row that you would pick?

Participant: Um.

Interviewer : If that, does that make sense?

Participant: Yeah, I totally get what you're talking about. Um. What I would say is ... oh, that's hard. I'd probably go with row two.

Interviewer : Okay, so row two. So you would get $100 for heads, and $30 for tails.

Participant: Yeah.

Interviewer : Can you explain a little bit why, why row two?

Participant: So row two, $30. Worst case scenario, $30 is, it's still $30. That's still manageable. I'm willing to risk a little bit than if I did with row 1, where it's still I'm still getting 50 regardless.

Interviewer : Sure.

Participant: Um, I'm willing to lose a little bit more but still come out with $30 if I didn't get the 100. But row three, although 200 sounds nice I'm not willing to just completely come out with only $10, because nowadays $10 doesn't, it doesn't get you anything. And then basically same thing with row four.

Interviewer : Great. Especially if you only have the 50/50 chance of getting either one.

Participant: Yeah.

Interviewer : Okay. So then row two.

Participant: Yeah.

Interviewer : Okay. So then the last thing that I have for you to do, um, is a small little questionnaire. And here's this pen back.

Participant: Thanks.

Interviewer : Um, so all of what's going onto the questionnaire is confidential, and anonymous. Um, it's not going to part of the interview. It's going to be put with other stuff. It's just kind of like general data collection like about you, stuff that we might not have covered in the interview.

Participant: Um, it says other, please describe, and then it says Hispanic or Latino in the next one. Do I just ...

Interviewer : Uh, how would you typically kind of answer?

Participant: I would say Hispanic. But then for like, but what about the two, do I just leave it as other?

Interviewer : You can write other, do other then write Hispanic in the next column, or next question I guess. Um, if there's any questions on there that you don't want to answer, you don't feel comfortable, again, please don't do that. Don't answer them. Um, there's a couple on ... just the way the paging got messed up. So like 23 down here, the question's here and then the response is here. There's one or two that are like that. So if you see just boxes with no question that's probably what happened.

Participant: Is there a specific reason then now a lot of people tend, do you know, or why it tends to be separated now, like Latinos from like this question? I've noticed this a lot. Like, Hispanic just got completely cut out and made like it's own separate topic. I've notice that a lot.

Interviewer : I've noticed, um, because you can identify as white non Hispanic, or white Hispanic. And so I think that's what the, those two questions are trying to get at. I'm not sure why that's been happening, but I have noticed it as well.

Participant: For this one, I would put high school diploma plus some college or university, right? Because I haven't got my degree yet.

Interviewer : Um, what is the highest level of education you have completed ... um, yeah.

Participant: So I'll do that. Um, living situation is that with school or at home, home.

Interviewer : Um, I would say for at school right now.

Participant: Because that's, that's where my dorm is.

Interviewer : I don't know.

Participant: Okay.

Interviewer : All right. Great. Okay, so that is just about everything. Um, I have this sheet I want to give to you. It's a list of PrEP providers here in [Upstate NY City 2], so places you can go to get it if you wanted it. And then uh, the bottom half here are different counseling services, um, one of the, the risks of doing kind of in depth interviews like this is talking about some things can raise, um, issues or feelings that you've been trying to push away for a while, so these are here if you do feel like you need someone to talk to. You do have access to them. If you need help scheduling an appointment or anything please let us know, and we'll do our best to, to get you seen by someone. Um, I, I don't know that you will need these, but it's important to have.

Participant: Yeah.

Interviewer : Great. All right. Um, so then again, on the back of the consent form, um, you have the, the contact information for the subject advocates. Uh, so if you want to talk to someone who's not involved with the project you can reach out to them. Uh, you have my phone number, so if you have any questions going forward, or want to know more about this please don't hesitate to contact me. Um, so before we get you paid (laughing) is there any other questions or anything that you want to bring up, or ... like if you, if you get home and in like half an hour you just have this burning feeling like, "I should've said this," like anything like that?

Participant: Um, no. I think I'm pretty good in terms of like everything I said.

Interviewer : Sure. Okay. Great. Well, thank you so much for sitting here with me for two hours and talking.

Participant: No problem. It honestly didn't even feel like two hours, so.

Interviewer : That's one of the nice things about doing these interviews, is the time goes by quickly. Um, so okay, I'm just going to go ahead and shut this off.

Interview IDM 108

S1 01:08:36.814 All right. It seems like you know quite a bit about it, about transmission, about prevention. Can you tell me a little bit about how you learned about that?

S2 01:08:47.654 The Internet.

S1 01:08:48.450 The Internet? So was it something that was ever talked about in high school in a sexual education class?

S2 01:08:56.359 Sexual education class. Let me remember it, what I learned [laughter] in sexual education class. I remember [enough?] anatomy. I guess they mentioned condoms, but they mostly focused on abstinence. Focused a lot on pregnancy, which doesn't help me at all. Nothing at all about gay sex, obviously. They talk about diseases, but it was mostly a list like, "Oh, yeah. These are diseases that you can get," and this. I don't remember. It's the sort of thing that I know I saw it, but in the way it was presented, it was the most uninteresting in the world. So I don't think I learned a lot from that in high school.

S1 01:09:50.250 Have you ever talked about HIV or AIDS with friends, then?

S2 01:09:55.812 Yeah. But the friend I told you about with his boyfriend, there was a time that he talked with me about it, like getting tested. That, for example, he after a year, I think, of the relationship, they decided to get tested, and then they stopped using condoms. But it was like, "Oh, yeah. We test ourselves for everything that we could. We are clean, so we're starting to stop using condoms [inaudible] sex to not have to explain to [laughter] who's wearing condoms." But besides that, with my straight friends, I don't really talk about it.

S1 01:10:53.735 Did it ever come up in conversation with any of the people that you dated or hooked up with?

S2 01:11:00.069 Back in DR, not much. With my boyfriends, yes, it has come up in the sense of previous sexual experiences, getting tested, and stuff like that. But with hookups, it doesn't [come up?]. I use a condom all the time, so yeah. When I started getting active sexually, there was a guy in our circle of friends from [inaudible]-- I don't know if I should use names.

S1 01:11:44.023 You could make up a name for him if you want.

S2 01:11:46.934 Oh, okay. Let's say, Manuel. That's the friend, where I told you, with the boyfriend. So we have a circle of friends. That was when I met him. And there was a guy in that circle of friends, he lived in another town. And he liked to do stuff bareback. He was on top. But I didn't want to take that risk. I knew that he and one guy from that circle of friends also had-- I knew them separately, and then they hit it off at one time. And I started avoiding them [laughter] because I guess I didn't-- I told myself that if I tried to have sex with them, that I wasn't really sure if I going to-- you know when you say, "Okay, I'm going to use a condom," but I was scared that in the heat of the moment, I would forget about it. So I decided to just avoid the possibility of the matter of having sex with one of them. But there are a few people in the community, I say, in Santo Domingo, who are kind of careless about it. So I always been very cautious about that in the sense of always using a condom. I only talk about the possibility of going bareback when I already in a relationship, inside a relationship. There's also the issue about-- I heard about PrEP before in the Internet, but for the life of me, I don't know where the fuck I am going to get that back in DR. I tried to look for it. I couldn't find anything. I mean, I don't know [laughter]. So I guess I just had to make do with always using condoms.

S1 01:13:58.524 Right. So then you said you have been tested for--?

S2 01:14:05.377 Yeah.

S1 01:14:05.604 Right. Did HIV or AIDS ever come up in those conversations with the doctors, or about PrEP, or condom usage, anything like that?

S2 01:14:13.942 Back in DR, last time I got tested before coming here, I had to make-- I had to take a few vaccines to be able to move here. So I took the chance and took the HIV test. I didn't talk a lot about it. It was like, "Yeah." It was, to me, it don't-- nobody talked to me about PrEP back there in DR. I guess they assume that you're straight.

S1 01:14:44.572 Possibly.

S2 01:14:45.973 Also, there is a conversation that I had with a few straight friends. I don't remember when it was. I guess it was this year or last year, in Chad, and we were talking about sex. This one guy would say it that, "Hey, but you don't have to worry about it. You don't have to use a condom. You ain't going to leave someone pregnant." And I was like, "Dude, what are you talking about [laughter]? Have you heard about diseases?" So I think that the mentality of a few people back in DR is that you only need condoms to avoid pregnancy.

S1 01:15:32.732 Right. So there's not necessarily the worry about the diseases that can come from sex.

S2 01:15:37.963 Some people don't care.

S1 01:15:40.128 So do you know anyone who's living with HIV or AIDS?

S2 01:15:47.832 One of the guys that I have been talking here from [inaudible] [shop?]. There is a guy that he says he's positive in the profile. I've been talking with him, but I'm not sure what to respond. I mean, I haven't-- I know that he puts it in his profile. I have a talk normally with him, but I don't know how to-- well, I kind of worried about, if it comes to sex-- but when I say I'm [bored?], I say that I started looking at it, about all these chances of infection and stuff like that, or what do you do when you do have a serodiscordant. That's the word?

S1 01:16:36.460 Can you say that again? I'm sorry.

S2 01:16:37.861 When you have a person that is positive and a person that is negative, how do you have sex without infecting the--?

S1 01:16:46.833 Oh, sure.

S2 01:16:48.010 So when I met him, I started talking with him. I saw that he was positive. I didn't talk with him about it. I haven't reached that point, but on my own, I started doing research because I guess my first instinct is to look on the Internet, not to ask people to them stuffs.

S1 01:17:08.284 Do you think his HIV status would affect your decision to have sex with him, or to hook with him, or--?

S2 01:17:20.370 Probably.

S1 01:17:21.165 Can you explain that a little bit? So how would it affect it, or why, or--?

S2 01:17:26.889 I guess that I wouldn't have sex with him until I know which practices are safe and which aren't: what can I do, what should I do, what shouldn't I do. If, from that research, I decide that it is fairly safe, then okay. Probably, knowing myself, I would be still nervous about it. But I guess it's just getting informed. I don't want to-- this may seem a way just for that, but I like to get informed.

S1 01:18:00.108 Of course, yeah. Can you tell me a little bit how HIV is talked about in DR or it comes up at all anywhere? Or how people who have HIV are treated?

S2 01:18:16.438 I remember that I went to a few talks organized by the LGBT community back there. There was a guy. I was talking about it. He said that he was HIV positive. I don't remember the context of the talk, but I know that in the community, there are people who talk about it. The community itself is very aware about the-- just for example, I remember reading a comic that someone from the community did about HIV, but it was mostly focused on wearing protection. And that's pretty much what I remember about the talk. Well, I know there are services for people infected to get the medicine. I know that it's something that actually is managed by an organization called Profamilia. It's kind of like the-- the [version?] here would be-- what's the name of-- what's the name of this organization that the Republicans want to--?

S1 01:19:36.908 Planned Parenthood?

S2 01:19:38.186 Yeah, Planned Parenthood. Let's say that Profamilia is kind of like a Dominican version of Planned Parenthood. And they have quite a few programs to fight teen pregnancy because that's a big problem back in DR. I mean, some people want to sweep it under the rug before most of the teen pregnancies occur in the poor demographic, in the relation, but it's a big problem. But also, I guess that I tried to find information about Profamilia in their page, but I didn't see a lot about PrEP. I guess, I tried to look on the Internet, and then I didn't find something specific. I think, [it said?] I had to go by myself and ask. That's what I didn't want to do [laughter].

S1 01:20:39.908 So did you end up going to Profamilia?

S2 01:20:42.445 No.

S1 01:20:43.278 No?

S2 01:20:43.609 No. At least not for-- I was curious about PrEP availability, but I didn't got around to ask.

S1 01:20:54.784 Right. Okay. So I have some more questions about PrEP that we'll get to in a little bit. But going back to HIV kind of more explicitly, can you tell me what you know about the transmission of the disease?

S2 01:21:11.065 I know that it's transmitted sexually, and that the virus-- I said correctly?

S1 01:21:16.591 Hmm.

S2 01:21:16.591 Yeah. And it's very hard for the virus to survive on the surface, so it is mostly direct contact with the blood or something similar. The virus, it has to be [inaudible] in specific situations because it's not as easily-- it can't be transmitted that easily. I know it can be transmitted by blood infusions like sharing needles and pregnancy. I don't know how, but I guess there are ways to avoid that now. But I don't really-- I haven't researched a lot about it. It doesn't--

S1 01:22:06.010 Right. It doesn't necessarily affect you.

S2 01:22:08.551 I know that it's progressed in that regard, but I haven't researched about AIDS. I also know that there is PrEP to avoid [development?], to reduce the chances of getting HIV. I know that there is another medicine, in case-- it's like the after pill but for HIV, where you have to take it for a month after the contact. Something like that. I have no idea if the is available in DR or it's covered by insurance. I didn't [encouraging?] to ask, mostly because back in DR I was in my mother's insurance, Migration. So I did it through her [laughter].

S1 01:23:08.821 Sure. So other than PrEP or kind of the morning after pill, what are some of the other prevention options for HIV? How can you keep it from spreading and keep from being infected with it?

S2 01:23:23.787 Besides using a condom?

S1 01:23:25.635 Sure. I mean, so condoms are one, and you did talk about that. But do you know if there's any other methods? Okay. So you're shaking your head, no. That's fine.

S2 01:23:34.631 No.

S1 01:23:34.957 So say if you did become infected or if you acquired the virus, what kind of treatment options are available? Or do you know of any?

S2 01:23:45.220 I know there's a medicine called Truvada. It's Truvada? I don't remember it very well.

S1 01:23:51.801 Truvada is something else.

S2 01:23:55.482 Oh, okay. I got them mixed around then. For [inaudible], I haven't researched a lot about it, so I don't really-- I guess I didn't want to research a lot about it. But in the general sense, I know that, I guess if you had the money to pay for it, you can pretty much live a normal life with it. Some people, if they take the medicine, they can have HIV in undetectable levels and, yeah, in a sense, that's okay. It probably will be something uncomfortable to talk about with partners and stuff, but it's manageable. It's not like in the '80s.

S1 01:24:46.312 That's true [laughter].

S2 01:24:46.884 Also, I remember I read, last year, a book called And The Band Marched On. It was a book about the spread of AIDS and [HIV?] in the US in the '80s. And that book hit me really hard.

S1 01:25:09.551 Can you explain a little bit about why it affected you so much?

S2 01:25:14.613 I guess that the focus in the book was-- there were narrating, like, "This is a chain of events since the first infection until--" there was an actor that got infected with AIDS. I don't remember the name. It was very famous. And then after that, when he started getting recognized, he brought it in the mainstream media. But I guess it was the combination of the personal event that I met with this of people dying from the community. And also the indifference from politicians or from the blood donation sites. So they wanted to stop accepting gay people. And it was the combination of everything, like, "Fuck [laughter]."

S1 01:26:19.463 So you talked a little bit about this. So the majority of information that you know about HIV and AIDS, how have you found that out?

S2 01:26:36.950 Asking questions on Google.

S1 01:26:38.082 Just on Google?

S2 01:26:39.022 Yeah.

S1 01:26:40.079 Are there specific sites that you've looked at, or is it just kind of a general--?

S2 01:26:44.193 I don't remember. Well, when I was in high school, in the very simple way, they taught me, "Okay, HIV is infected to sexual transmission. You can avoid it by using a condom, by abstinence, of course." I think that was pretty much it. Also, "You can get infected by sharing needles, by blood transfusions, and by pregnancy." And that was pretty much everything they told me about it. But it was enough to start asking more questions later, a few years later.

S1 01:27:21.696 Right. So other than the book that you read, have you seen or heard anything about HIV or AIDS in other forms of media? Like in TV shows, in movies, other books?

S2 01:27:41.468 TV shows. Right now I can't remember a TV show that talked about it. But also, there was a time when it was-- there was a time when I watched quite a few gay movies, so probably-- I might not remember it now, but I probably have learned something about it. But it was still in high school and start of college. So it was my way to know about the community, but right now, I can't tell you a specific movie, a specific something.

S1 01:28:27.390 That's fine. So can you tell me about personal practices about using condoms? I know you mentioned you try to always use condoms, but are there times where you haven't used them, or situations or circumstances where you might decide not to?

S2 01:28:48.191 Situations where I haven't used it, in my second relationship. There was a time after we started dating that we got tested, and then we were negative, and then we stop using condoms. That was, more or less, after we were long-distance. In retrospective, it might not be that smart to do, but I trusted him. So I guess that's a risk that I took. But in the end, I didn't got infected, so I guess it went well. But I don't know if I could do something like that now. Okay, you're going bareback with someone who is long distance, and maybe for the few times where I could have met to be together. But in that relationship, I did that. There have been other cases where I didn't have a condom, or I didn't want to, but I just didn't do anal sex. I just did oral, or used less [inaudible] or something like that.

S1 01:29:55.955 Right. So outside of the relationship, you've always used a condom?

S2 01:30:01.054 Yeah.

S1 01:30:02.751 So are there circumstances where you might not use it? So, moving forward in the future, are there factors that would kind of make you think, "Oh, maybe I don't need to use a condom right now, or--?"

S2 01:30:16.274 It would have to be-- knowing myself, I would be very paranoid about it. So it would have to be someone that I know that is negative, and that I know that would be like-- it's just that, if you suggest me to not use a condom, I automatically assume that you're doing the same with any number of people. And the thing that, when I stopped using a condom with my second boyfriend, I noticed that it feels better [laughter]. So there's a temptation there, but the paranoid side of me, usually it wins, so.

S1 01:31:06.762 Sure. So have you ever been worried that you were infected with HIV or you had contracted it somehow?

S2 01:31:15.790 Yeah.

S1 01:31:20.154 Can you tell me a little bit more about that?

S2 01:31:23.672 Before coming here, I was already with my third boyfriend. But since the breakup with my second one and my third, I haven't got tested. I use condoms with him, with my third one, but I haven't gone to test me. When I took all the tests before coming here, I told him-- I didn't tell him that I did bareback with my second boyfriend, but I told him that, "What if I infected?" I know, me being paranoid. And my friend felt it. "What if I didn't know about it? What if I infected you?" I started [laughter] going the train of paranoid talk. In the end, it was okay. But, I guess, he probably thought I was really paranoid. But since I have the [laughter]-- since I have the history with my second boyfriend, I was worried about that.

S1 01:32:28.861 Sure. So did you get tested then, or--?

S2 01:32:32.023 Yeah.

S1 01:32:32.484 So when was the last time you got tested?

S2 01:32:34.734 I came here almost 19, and all the testing, the vaccine, and all the medical stuff, it was in the 2 weeks before coming here.

S1 01:32:48.067 Oh, so fairly recently.

S2 01:32:50.280 Yeah.

S1 01:32:50.700 Okay. And did any of the tests come back positive for anything?

S2 01:32:56.327 No. They even had to do a tuberculosis test. Yeah. Just because I live in Dominican Republic, they require me to do that.

S1 01:33:05.612 [Interesting?]. So other than the HIV, have you been worried about having other sexually transmitted infections?

S2 01:33:14.458 There was a time a few years ago that I was with a guy. We used condoms, but we also did oral. Days later, I had burn when I pee, and I was worried about that. And I took a urine test, but it didn't come with anything. So, I guess, I don't know what happened there, but I was worried. I didn't know what to tell my mom, but I just said, "Hey, I want to--" I just feigned ignorance like, "Hey, it hurts when I pee. I want to [inaudible]. You know where I can take a urine test?" I was pretending that--

S1 01:34:16.050 And was she--?

S2 01:34:17.102 --it might be something else [laughter].

S1 01:34:20.655 So how did she react to that?

S2 01:34:25.856 I was very scared [laughter], yeah.

S1 01:34:29.618 But so did you actually tell her that then, or--?

S2 01:34:33.309 I told her that I wanted to take a urine test, but I didn't imply any sort of relationship with sexual activity. I just say that this is what happening.

S1 01:34:46.532 So when you told her about that, how did she react?

S2 01:34:51.435 She either knew but didn't say anything about it, or she didn't knew. But she told me about a clinic that used to do tests nearby from where I used to work. And then I took the test, and it was [read?] okay. I don't really know what happened with that, but it only last me like one or two days.

S1 01:35:14.224 Did you ever talk to that guy about it, or--?

S2 01:35:18.802 The guy would probably be in another country at the time [laughter], but I didn't tell him anything about that. I guess since the test that I didn't have anything, I just ignore it.

S1 01:35:35.341 Okay. So in the future, how worried are you about contracting HIV?

S2 01:35:47.981 I guess that I don't know if I'm going to be more worried here because back in DR, what I used to do, it was having a serious relationship, focusing more on serious relationships. But since I decided that I wasn't going to that here, I probably could be having more sex than usual. So I guess I'm going to be worried about that.

S1 01:36:19.588 Okay. So if you had to kind of assign a number to it, so say on a scale from 0 to 100, where 0 is absolutely lowest chance, will never happen, and 100% is absolute certainty - it's the highest chance - where would you say your risk of contracting HIV falls on that? Does that make sense?

S2 01:36:44.250 Yeah. Just, well, given my breadth of-- if I always use condoms. But I'm not a [prick?], and I might be doing more relationships down the line, so there is some risk, but not really that much. So between 30 and 40. Let's say 35.

S1 01:37:13.435 35%?

S2 01:37:14.291 Yeah. Something that I should be aware about. But there's something I can do better by, but not really that.

S1 01:37:26.333 All right. That makes sense, so thank you. So moving into the next set of questions, we're actually going to start talking about PrEP. So it's kind of like with the questions about HIV, just kind of trying to see what you know about the drug, about pre-exposure prophylaxis or PrEP.

S2 01:37:46.586 I know that there are people that have secondary effects like nausea, something like that, but it starts receding after a few weeks of taking the medicine. I also know that it's not effective until after the first week of taking it every day. I heard something about taking it every day at the same time, at the same hour. I don't know if I can do it.

S1 01:38:18.306 Well, we can get into a little bit more depth. But just let's start off with, can you tell me who PrEP is for? Who would be taking PrEP, basically?

S2 01:38:33.572 People who are HIV negative who are in high risk. Of course, they'd consider themselves in high risk or are dating someone who is HIV positive.

S1 01:38:49.144 Okay. Is it specific to a certain gender, or age grouping, or anything like that?

S2 01:38:57.748 I don't think it should-- I think no. I didn't read anything about it being, yeah, for a specific gender or something like that.

S1 01:39:09.997 Yeah. So how have you found out about PrEP? I know you talked a little bit earlier about looking online, but can you tell me a little bit more about that?

S2 01:39:21.518 I can't remember the first time I read about it, but I guess it has to be in a forum or something, or maybe in the news. There should be one place where I read about-- I can't pinpoint a specific moment where it has to be maybe a new-- I'm pretty sure that it would be something local from the DR. Probably something from international news in English, or I was reading about something on Facebook, or--

S1 01:39:59.018 Sure. So is PrEP something that you've ever talked to your mom about?

S2 01:40:04.594 No [laughter].

S1 01:40:07.158 Why not? Is there a reason why you wouldn't want to talk to her about it?

S2 01:40:15.340 I don't talk to her about this sexual stuff.

S1 01:40:17.710 That's--

S2 01:40:18.287 There's probably a line of people that I will talk about sex, and she's probably down the line, like--

S1 01:40:26.628 She's way over there.

S2 01:40:27.523 Yeah.

S1 01:40:28.842 Have teachers ever mentioned PrEP, like in a sex ed class or anything like that?

S2 01:40:36.126 No. Did you know that in my college, in my undergrad, I had to take two required classes about religious studies--

S1 01:40:50.250 Religious studies?

S2 01:40:50.630 --even though my major was engineering? It's required by the university because it's a Catholic university, so you have to take two electives for religious stuff. One of them was Christian life, family, and marriage. In family and marriage, I remember there was a test that I-- I had the teacher subtract me points because I broke the definition of marriage. I put two persons, and he wanted me to put man and woman. So yeah, I was really quiet in that class [laughter]. But no, I'm pretty sure that the last time I took sex ed classes was in high school, but at the time, I had no idea was PrEP was. I'm pretty sure of that. I only knew about the points I told you about HIV. It didn't go beyond that.

S1 01:41:49.950 So when you've gone to get tested or gone to different clinics, have doctors ever brought up PrEP?

S2 01:41:57.325 Not that I can remember, no.

S1 01:41:59.304 Have you seen anything in their office or waiting rooms? Like different brochures for it or anything?

S2 01:42:06.917 I seen a lot of brochures but mostly focused on pregnancy prevention. That's the big focus back there in DR.

S1 01:42:15.998 Sure. Have you told--?

S2 01:42:18.008 I've told--

S1 01:42:18.639 Oh, go ahead. Sorry.

S2 01:42:19.332 In the Profamilia website, I think they have a [amazing?] catalog, but I haven't checked. They probably have, maybe, medicine, but I don't know if it would be for people who are already infected. But now I think maybe I should have researched more, but I kind of--

S1 01:42:48.494 Is PrEP something that you've talked about with friends or partners?

S2 01:42:58.692 Partners, only mention, but they got around the idea of searching about it. Sorry.

S1 01:43:14.283 Oh, you're fine.

S2 01:43:15.196 I have a circle of friends, gay friends, queer friends, male and female. Within ourselves - we are four or five persons - we talk a lot about topics of society, activists, and stuff like that. We, within ourselves, I have mentioned the PrEP stuff. In that case, if I wanted to talk about it, it would be with them because the group, I kind of trust more with that sort of information.

S1 01:44:04.522 So you said you have talked about it with partners in the past?

S2 01:44:10.474 Mentioned.

S1 01:44:11.190 You mentioned?

S2 01:44:11.907 Yeah. Something [inaudible] mentioned, but really consider for taking [inaudible] relationship.

S1 01:44:18.578 Do you know anyone who's currently taking PrEP?

S2 01:44:22.986 Back in DR?

S1 01:44:23.906 Or here in the US or back in DR.

S2 01:44:26.077 Back in DR, no. Here in the US, quite a few people, they put in their profile in SCRUFF. They put they're on PrEP. I mean, I don't know if I should trust that information, but--

S1 01:44:41.229 Have you talked to any of those people about PrEP?

S2 01:44:43.790 There was a guy that, actually, he even told me about it. Like I could get-- in RIT, I could ask around in the health services. I could get the resources to [inaudible] [stay on?] PrEP. I was thinking about doing that, but, well, I also saw the size [laughter]. But there has been at least one people here that have told me about it, but back in DR, no. It's like I went from a being in a place where at least something-- I know some people in DR would know about it, but it's mostly something that happens in your country.

S1 01:45:33.011 Sure. So have you thought about taking PrEP?

S2 01:45:38.234 Yeah. I will have to-- I went to see if my insurance covers it or if I had to pay for it because I have insurance for [inaudible] risk of infections [and stuff?]. I don't know if it covers it. I guess, [I prefer?] [inaudible] classes than having told myself to research to see what steps would I have to take and stuff like that. But I'm thinking about [inaudible] it.

S1 01:46:09.135 So other than insurance, what are some of the factors that would affect your decision to take PrEP?

S2 01:46:17.327 I'm not sure, now, if I have to take it every day or every certain time. I'm kind of bad with that and that sort of stuff for medicine in general. I know that taking PrEP and not sticking with the schedule can be worse because the effectivity of it decreases. But like I say, I'm pretty bad with that, so I don't know if I could use. That would be a factor, I guess.

S1 01:46:59.613 Sure. So I'd like to ask you about some things other people have told us are important about PrEP. So you mentioned how frequently or how regularly you have to take it, the insurance. So just asking about what they've said is important and what you think about that stuff. So there's no right or wrong way to answer these, and it's fine if you don't really have anything to say. If I say, "Well, what do you know about this?" And you can say, "Nothing." If you don't know, that's fine. So what do you know about the cost of PrEP?

S2 01:47:35.687 I'm getting conflicting information with that because I've seen people say that it can be covered by insurance. But for someone in my case, they recommend-- when they gave me the insurance information, only specified emergencies and stuff. Doesn't know if it covers much. I don't know. I think it's expensive. I'm not really sure about it.

S1 01:48:09.908 Would you feel comfortable-- if your insurance covered it, the international student insurance, would you feel comfortable using that?

S2 01:48:18.400 I would give it a try.

S1 01:48:19.585 As opposed to, say, using your mom's insurance?

S2 01:48:24.695 When I came here, I decided to use the international insurance because in order to be able to use my mom's one, I would have to upgrade it to cover international expenses. That was expensive. I already had to pay for the plane ticket and other stuff, so I decided to-- I need the money, so I just use the insurance that the scholarship issued to us, so.

S1 01:48:56.981 Sure. Okay. Have you heard about people having trouble getting insurance to cover it?

S2 01:49:05.023 Not really. I guess it's just-- not that I haven't heard about people having trouble in the-- I haven't heard much about that topic in general. I'm really still kind of confused. The whole issue with insurance, and medicine, and prices, I'm still kind of in the air with it. I haven't finished researching.

S1 01:49:34.111 So what, if anything, have you heard about where you can go to get PrEP?

S2 01:49:41.016 I guess, that the student health center in the university. I don't know if I can just go to a regular hospital and ask for it, but probably I would prefer to go to the center in the university. And I think that's it. I know about the pill offer. I know that fast as possible is to-- fast as possible. That thing.

S1 01:50:13.445 Yeah. Like the morning after [crosstalk]. Yeah.

S2 01:50:14.667 The morning after, yeah. I know that you can go to emergency room and you can ask for it. Last Friday there was a activity in the university. It was like a Bingo with some drag queens, and they were talking about sexual terms. And they mentioned that you can go to any emergency room, and you can ask for it. It was super silly and [educational?] [laughter].

S1 01:50:42.751 [inaudible]. So you talked about this a little bit already, but what have you heard about taking PrEP itself? How frequently? What type of medicine is it? Is it an implant, an injection? Is it a pill? That kind of thing. How frequently do you have to take it? Those kind of questions.

S2 01:51:13.262 Now that I think about it, I get the morning after one and PrEP kind of mix up. Because I know that the morning after one, I have to take it every day for 30 days. But right now, I can't remember if it's the same frequents with the PrEP or if something a little bit spread out. So I going to say that I think this, they're pills, but I'm not really sure. I guess I focus more in the effectivity than how you take it. So I kind of-- I don't like needles, so I guess I know that-- I don't like needles, so that would be a factor because taking a needle every day would be, oh, wow.

S1 01:52:01.393 So say if it was an implant under your arm here - they do something similar things with birth control - would that be more preferable to an injection or to taking a pill?

S2 01:52:15.725 Do you mean you have an implant and you-- how often do you have to--?

S1 01:52:21.339 So say, maybe every few months, maybe every three or four months, that would have to be changed out.

S2 01:52:28.011 That would be nice, actually. I just have to put that reminder in my calendar, and--

S1 01:52:32.706 Yeah. All right. So you mentioned the effectiveness is something that is important to you, Rick. So where have you heard about the effectiveness of PrEP?

S2 01:52:44.241 I remember percentages. It was something about-- well, it will reduce the chance of infection by 90-something percent, but since that percentage, that's about it. But I heard it's very effective. But it's not - what? - something like 100%, but also condoms aren't either at 100%, so it's more like it's stacking the [inaudible] in your favor.

S1 01:53:19.820 How effective would PrEP have to be in order for you to take it? So what percentage would it have to be effective by for you to consider wanting to take it? Does that question make sense?

S2 01:53:36.371 I guess, yeah. In my case, there was something more about effectiveness but also more about accessibility. If I think it is less accessible, or if it's, say, covered by insurance, or at least not expensive, I could say, "Okay, even if it's only a 50% or 60%, if it's cheap or if I can afford it, I can do it." Anything that increases my odds. But be more expensive, be more be less accessible, it will have to be really good to compensate.

S1 01:54:12.781 Sure. No, I understand. So you mentioned that you have heard a little bit about side effects of PrEP. So can you tell me about that?

S2 01:54:24.301 I read that people will be-- for example, there may be symptoms of mild nausea or [foreign]. I don't know what to say in English.

S1 01:54:36.129 I'm not sure.

S2 01:54:36.529 Yeah, nauseous, headaches. But I think that only like mild effects, and they go away after a few weeks. And if they don't go away, then you have to talk to your doctor to see what you can do. But yeah, pretty much that's all.

S1 01:54:58.052 Are there any side effects that you would be particularly worried about, or--?

S2 01:55:03.442 I don't remember any.

S1 01:55:05.318 So say--

S2 01:55:07.047 Maybe something I would worry about, [I know?]. I guess, as long as they go away in one or two weeks, I don't really mind.

S1 01:55:22.944 Okay. Do you think people look-- do you think people look favorably or badly on people who take PrEP? Is there an image that's associated with people who take PrEP.

S2 01:55:39.922 Not really. I mean, at least here in the US, I seen that the gay community is very focused on-- they talk a lot about it. So I don't see a trouble with it, but probably in DR. I guess that one of the reasons why it hasn't taken off is mostly because it's marketed to gay people, so pretty much that kills the opportunity to see it in DR. But, I guess, I think almost it's be useful. But I mean, if you're straight and you take PrEP, it only protects you from HIV, but it doesn't protect you from pregnancy which is also a big problem back in DR. So that I pretty sure that if it is the problem in the [inaudible] that do that, there's going to be someone who is going to say that the medicine is promoting promiscuity and depravity for all the [inaudible].

S1 01:57:00.953 Oh, yeah. So do you think that's how most people think, that it promotes promiscuity?

S2 01:57:10.975 I think that most people don't even know about that, probably, at least back in DR.

S1 01:57:14.752 Right. So have you heard anything about PrEP on campus here at RIT or about how people kind of think about PrEP in the US?

S2 01:57:24.410 I heard about PrEP in the [the big one?] [inaudible], and mostly from the gay people that I met. But outside the community, I haven't heard a lot. I guess I haven't had the chance yet.

S1 01:57:39.437 Sure. You haven't been here that long yet either, so. If you were taking PrEP - let's say your insurance covered it, and it was easy to take, and you were on it - would you tell sexual partners?

S2 01:57:53.093 Yeah.

S1 01:57:53.847 Okay. Why? So why would it be important to share that information?

S2 01:58:02.019 Well, I think that I would still use a condom, even though I used PrEP, until there is a time I'm so comfortable with PrEP that I will mind using condoms. But the way I see myself now, I will use both, so I just don't see why not share that.

S1 01:58:24.546 Sure. Okay. If you were hooking up with someone and they were on PrEP, would you expect them to tell you?

S2 01:58:34.599 Not really. Right now my [inaudible] just use a condom no matter what. So I guess it would be nice to know, but it won't be required.

S1 01:58:48.722 But then you still feel like you would share it without necessarily being asked?

S2 01:58:55.357 Yeah.

S1 01:58:56.399 Okay. So say you were talking to someone and you were going to hook up with them. If they told you that they were on PrEP, would that affect your decision to hook up with them or to have a relationship with them?

S2 01:59:12.641 No.

S1 01:59:13.252 No. Okay. Would it make a difference if-- say, you were in a relationship with someone, and they came to you and said, "Oh, I just started taking PrEP." Would that have an affect on the relationship?

S2 01:59:26.299 It depends if it [inaudible] the relationship. But yeah, I guess not. I guess that I understand why people would be worried about that because they would be thinking, "Oh, yeah. You're taking PrEP, so you're worried about someone else besides me." But still, even if he comes, I don't usually feel jealous. But if it comes to that, you say, "Did you want to open the relationship? Or do you want to talk about it? Or do you want to start going bareback?" I guess if you ask questions or whatever, it make him be suspicious.

S1 02:00:08.526 Okay. Okay. So we're getting close to the end, I promise [laughter]. We've been talking for a while. So I actually have this sheet here that I'm going to give you, and you're free to take it with you when the interview is done. So this is just a question of different facts and information about PrEP itself. I--

S2 02:00:33.714 Sorry, I--

S1 02:00:35.065 Oh, that's fine.

S2 02:00:36.477 Oh, just--

S1 02:00:39.533 Do you need to go, or--?

S2 02:00:41.381 No, no. Don't worry.

S1 02:00:44.838 Okay. I promise we're almost done. There's that, there's one other little sheet, and then another little exercise, and then it's done. But so this is just some information about PrEP. So I'd just like you to just take a minute or two to read through it, and then we can kind of talk about what you've learned.

S2 02:01:03.191 Okay.

[silence]

S2 02:01:15.841 Oh, Truvada. The pill.

S1 02:01:17.247 So when I said earlier Truvada is something else, it's the same thing as PrEP [laughter].

[silence]

S2 02:02:43.836 Well, I didn't know about the program in New York for people who weren't covered by insurance. I guess I could ask about it in the health center back at RIT. I also know about-- well, I also know that PrEP only works with HIV, so will still need the condom to not get gonorrhea or all that stuff, so that's probably a factor that I'd probably still use condoms with PrEP. And the kidney issues, I didn't know about it, but I guess it's a trade-off. I don't really [like it?].

S1 02:03:27.832 I mean, is that kind of alarming to you or kind of something that you'd be thinking about if you were to take PrEP?

S2 02:03:34.141 If it go away if the medication is stopped, it probably is fine. Also, I don't have [berries?]. I don't have issues with my kidneys, so I guess it's fine.

S1 02:03:46.313 Right. So was there anything else on the sheet there that was kind of surprising or you thought it was cool to learn about?

S2 02:03:55.153 The PrEP is every day, every three months, further testing, I guess. I guess that's fine, yeah. And [inaudible] yourself. Everything else, you know what your status is, so you don't have to worry like it was in earlier times.

S1 02:04:27.549 And does any of the information there kind of change how you think about PrEP or change deciding to use it?

S2 02:04:37.530 Change, yeah, but in a better way. I'm more comfortable with using it, but my concerns were mostly about the accessibility. So I am pretty much convinced on the other stuff.

S1 02:04:52.627 Sure. Do you think if people knew more about these facts or more of this kind of information about PrEP, do you think they might be more likely to take it or decide to?

S2 02:05:03.235 Yeah. Well, it depends. I'm going to think that if my friends back in DR think the same way as me, they will be more likely to take PrEP. The big concern would be accessibility because there is no way that we can afford it. I mean, I guess, back in DR. But otherwise, I don't think it would be a problem.

S1 02:05:35.110 Yeah. So your friends back in DR or some of the places here in the US, do you think people actually know some of this information?

S2 02:05:46.077 I don't know about straight people [laughter], but I think gay people do know about it, at least here in New York. I only [blame?] in New York state or Washington, and there was a kid in Miami, so that shouldn't be a good reference. So I don't know anything about red states or [swinging?] states. I have a friend that lives in Utah. And, well, he thinks this stuff's bad. He's telling me really interesting stuff. I don't think it's-- it mustn't feel like the same country, actually.

S1 02:06:28.423 Okay. Well, thank you for reading through that, for sharing your experiences your body's been-- you're really open about things that some people like to keep private, so I really appreciate it. And before we're done, I have two small little exercises that I'm going to have you do. And then there's a short questionnaire survey kind of thing that I need you to fill out. So the first of these exercises is, on these index cards here - there should be seven - are some of the factors that people have said are important. So when I asked you, "What do you know about the cost, about the effectiveness?" that kind of thing, those are on the cards here. So what I want you to do is to read through them, see if there is anything you think is missing, like if you were to add another card to this pile about important factors. I have blank ones here that we can write on. And then I want you to put them in order of most important to least important across the table.

[silence]

S2 02:07:44.739 [inaudible].

S1 02:07:46.196 Oh, if you can't read my handwriting, please just tell me.

S2 02:07:48.448 It's fine.

[silence]

S2 02:08:02.812 Okay.

S1 02:08:11.589 So is there anything that you would add to these cards, or--?

S2 02:08:13.759 I think that, no, they are good.

[silence]

S2 02:08:49.474 I guess that, in that order.

S1 02:08:52.598 Okay. So the most important to you is ease of access?

S2 02:08:57.165 Yeah, yeah. I got to kind of decide between the two, but yeah, ease of access.

S1 02:09:03.386 Okay. I mean, you could put them maybe in the same position if they're of equal importance. Okay. So tied for first place, then, is ease of access and cost?

S2 02:09:15.299 Yeah.

S1 02:09:15.949 Okay. Then, how often you have to take it. Then effectiveness. Then how you can take it, stigma, and then the least important is the side effects.

S2 02:09:27.475 Yeah. That's mostly because the side effects are really mild and they disappear away mostly in the first two weeks. So I feel like you forget about it, so.

S1 02:09:36.867 Right. So later, I won't have the cards set up in the same way, so by saying it out loud, I can revisit this. So is there a reason why you've kind of put ease of access and cost tied?

S2 02:09:52.046 Mostly because of the fact of where I come from. That the main barrier for me-- I mean, I knew about it, but it was mostly because I read about it on the Internet. And the main barrier for not getting that back in DR, it was accessibility and cost. I had no idea how to get it. I had no idea, if I took, how much I had to pay for it. So that's pretty much-- if something is expensive, if medicine is expensive, you won't get people to buy it back in DR, even if it's good. Have to eat something [laughter].

S1 02:10:37.638 So okay, thank you. And so then the next most important was the how often you have to take it, and is that because you mentioned--?

S2 02:10:45.654 That I might be-- I'm not always really strict with the medicines that I have to take. For example, when I was teenager, the medicine for the acne, I was really irregular with it [laughter]. But I guess that's aesthetic, so probably it's different. But it's something self-discipline. I know it might be a problem for me, but I also know I only have to shut up and do it, so it's something personal.

S1 02:11:26.759 Sure. Okay. All right. Thank you for taking the time to sort those out. So if you want to put them back together, I can just set them aside. All right. So then the second exercise that I have, so I want you to take a look at this. And so this is kind of a hypothetical thing. We're not actually going to actually do this, unfortunately. But so imagine that I was going to flip a coin, and depending on which row you pick, you would get the amount of money for how the coin lands. So for example, if you pick row one, if the coin lands on heads or tails, you get the $50 either way. For row two, if it's on heads, you get 100, but if it's on tails, you only get 30. Does that make sense?

S2 02:12:14.655 Yeah.

S1 02:12:15.415 So for you, in this hypothetical situation, which row would you pick?

S2 02:12:24.181 I'm pretty sure that I wouldn't pick the last two rows. Too risky. So it would be between the first one and the second one. It would be if I feel lucky that day, or-- probably the second one.

S1 02:12:54.039 So row two?

S2 02:12:55.041 Yeah.

S1 02:12:55.616 Okay. Why that one as opposed to row one?

S2 02:12:59.065 Row one is pretty much-- well, I guess I'm telling here how risk averse I am. Row one is still a whim. Row two, even 30, I still get some-- either way, I can win in. Row three, I already spent 10 bucks coming here, so I'm pretty much [inaudible]. So I guess this possibility of earning money I wasn't expecting, but even if the worst case scenario, it's still pretty good. It's still enough winnings. So some risk, but not so much that-- even in the worst situation, you would still win something. You're still better than it's-- you're still winning something, so.

S1 02:14:00.510 Right. Okay. All right, thank you. So then the last thing that I have for you is this short kind of survey that I just need you to fill out. If there's any questions in there you just don't want to answer, don't feel comfortable answering, just leave it blank. That's fine. So you have the pen there. This isn't connected to the oral interview that we're doing now. This is just more so we have--

S2 02:14:30.118 Demographics? Like the--

S1 02:14:30.782 Demographic kind of data about that. Because the second stage of this project is much more of a quantitative focus, so we need some of this information to help design that part. So it's not attached to your name. It's not attached to anything you've said here. So if you're not comfortable answering anything, just leave it blank. Sorry, before you go through, the way it prints out is kind of strange. So like number 13 here, this question, and it goes number of male partners in your life, and then the responses are the top of this page. And I think it happens one or two other times. So if you don't see a box or a line, it's probably on the next page.

[silence]

S2 02:15:33.528 In the Caribbean, well, it's like a mix. I'm going to put Latino too. We have black heritage, but it's not fully black like you see here, so.

[silence]

S2 02:16:38.001 I am in a scholarship, so I don't have to pay rent. It's covered for me. But my earning to a job, it's just I had to calculate a year [inaudible].

S1 02:16:58.307 I mean, even a rough estimate is okay.

[silence]

S2 02:17:53.364 In my case, I'm covered through my-- I don't know, it's the school's [inaudible].

S1 02:18:02.806 So I would do other at the bottom. And then just maybe write school insurance, or--

S2 02:18:10.908 RIT International Student Insurance.

[silence]

S2 02:19:05.992 Pretty much. I came here last month, right?

S1 02:19:08.986 Yeah, if you said August 19th.

S2 02:19:09.838 So I guess pretty much two; my ex and the guy from last week [laughter]. I don't know if I remember his [laughter].

[silence]

S2 02:20:56.141 Not drugs, but alcohol.

S1 02:20:58.572 Sorry, what was this about? In the last six months, how often were you--?

S2 02:21:01.257 I was drunk once.

S1 02:21:03.288 So I think maybe then just the--

S2 02:21:09.601 I guess once or twice, but not really. I don't do drugs.

[silence]

S2 02:22:09.163 I have a herpes breakout but in the mouth, but not really genital. I have this since I was a kid in the mouth, but I don't think that counts as yes.

S1 02:22:23.434 I don't think so, unless it's a sexually transmitted thing, but.

S2 02:22:29.987 No, I think it was some kids when I was a kid, I guess.

S1 02:22:33.103 Yeah. No, so then I don't think I would mark anything there.

S2 02:22:44.568 That's pretty much it.

S1 02:22:45.636 Okay. Thank you for filling that out. Okay, so then the last thing to do is to give you money, to compensate you. So before we do that, were there any kind of final thoughts or questions that you had?

S2 02:23:05.012 No. Well, if you finish the research, where can I read it [laughter]?

S1 02:23:10.410 Yeah. Sure, yeah. I'm not sure how long the project is going to be.

S2 02:23:17.675 [inaudible].

S1 02:23:18.135 We're still in the process of interviewing people. And then, like I mentioned, there's going to be that second portion that's much more quantitative, and it's going to be a larger scope, I believe. But you do have our contact information.

S2 02:23:32.999 I think.

S1 02:23:33.763 It should be on there. You do have my email and the phone number. But I believe that once we have actual kind of results or things that we're working towards, there's no real reason why that couldn't be shared. I'm just not sure about a timeline--

S2 02:23:55.976 Okay. But [inaudible].

S1 02:23:56.705 --because it took us a long time to get approved to do the project at all [laughter]. We were supposed to start over a year ago.

S2 02:24:06.449 Oh, wow.

S1 02:24:06.913 It just kind of kept getting pushed back. So, hopefully, it'll be wrapped up in the near future. So just one thing that I do want to mention on the consent form, just as a reminder, at the back are the subject advocates. It's just important to highlight that if you want to talk to someone who's not part of the project, they're the people that you would give a call to or send an email, just as a reminder. And I have this sheet for you, just something to take with you. It's a list of places in [Upstate NY City 2] that will provide PrEP. So this top half is different places that you can go to to talk to someone about getting it if you're interested. The bottom half are counseling or therapy kind of services. One of the risks of an interview like this is you could bring up something that you have really negative feelings about or is kind of emotionally difficult to talk about. So, if that happened, then there are resources available to you to talk to someone else about that. If you want to schedule a counseling session or a therapy thing but you're not comfortable setting that up, you can reach out to us, and we will help you do that. But these are just kind of resources for you, in case you are interested in pursuing any of them. All right. So if you don't have any other questions or comments, we can get you paid, and then we'll get you out of here.

S2 02:25:34.544 Okay, sure.

S1 02:25:35.129 All right. And then, you said there was a financial cost for coming here?

S2 02:25:43.422 Oh, yeah. The Uber.

S1 02:25:44.480 You took an Uber? Okay. From RIT?

S2 02:25:47.594 Yeah. From RIT to here.

S1 02:25:48.889 Okay. All right. So this is kind of just for budgetary things. It's just acknowledging that you have received money or compensation for your time. So I just need you to-- on this first line here, you're going to print your name, sign, and then put the date. And then that's the amount that--

[silence]

S2 02:26:34.046 9--?

S1 02:26:35.612 The 22nd, yeah.

S2 02:26:36.282 22nd. Yeah [laughter]. I always remember that you write the date a different way, so I had to--

S1 02:26:44.894 Oh [laughter].

S2 02:26:45.565 --stop myself and write the 22 first.

S1 02:26:48.190 Right. Okay, so here is 40. The compensation for the interview itself. And then 5, 6, 7, 8, 9, 10. That's all the travel reimbursement we're allowed to give out, but hopefully that does cover a bit of it.

S2 02:27:05.042 Yeah. That covers the Uber, yeah. Thank you. Thank you very much.

S1 02:27:08.337 Yeah, thank you for taking the time to talk to me. I really, really appreciate it. Okay, I'm going to get this turned off.

Interview IDM 109

Speaker 1: Yeah. Um, and what about ways to prevent HIV-

Speaker 2: Well.

Speaker 1: What do you know about that?

Speaker 2: Well I know that, uh, PrEP exists-

Speaker 1: Mm-hmm (affirmative).

Speaker 2: Um, I know that condoms do, they work to an extent, um-

Speaker 1: Just to an extent?

Speaker 2: Well I mean, you know, they, from, from my understanding condoms are not, uh, 100% effective in any scenario. Um, and so you've just gotta be careful. Um, obviously abstinence-

Speaker 1: Okay, (laughs) yes, that will work 100% of the time.

Speaker 2: That, that is the 100%-

Speaker 1: At least for sexually transmitted infection, yeah. Um, and so what are treatment options, what do you know about HIV related treatment options?

Speaker 2: Um, well, I know that there, well I know that like, if you think you have, if you think you've come in contact with it you can, like, run to the, um, to like a treatment center and they'll give you, um, the PEP, the post exposure prophylactic. Um, and that, and I think it's like 72 hours within contact, um, aside from that I mean, I don't really know what treatment options exist, uh, I don't really know what the treatment is for when you really have it, so.

Speaker 1: Okay, I, I mean this isn't going to be a test-

Speaker 2: Yeah, yeah, no.

Speaker 1: I'm ju- I'm just trying to get a sense of what-

Speaker 2: Mm-hmm (affirmative).

Speaker 1: You know, where your sort of, where you're at with HIV and AIDS.

Speaker 2: Yeah.

Speaker 1: And you said that most of the information that you have comes from online, do you have any other, do you identify any other sources of information, like I may not know what they talk about in health class in high school, I don't know if they teach about HIV and AIDS in high school these days.

Speaker 2: Um, well that's, that was, you know, 6 or 7 years ago-

Speaker 1: Mm-hmm (affirmative).

Speaker 2: But, um, I don't think they taught us that much about HIV in health class, I really don't remember what they taught us at all.

Speaker 1: Okay. So it's mostly internet you were saying.

Speaker 2: Yeah.

Speaker 1: Talking to doctors?

Speaker 2: Yeah I've talked to, um, I've talked to, um, I didn't talk to my, my primary care physician, but I, I have talked to the doctors that we have here at school.

Speaker 1: Mm-hmm (affirmative).

Speaker 2: Um, about HIV and other STDs so.

Speaker 1: Okay. Um, and so we've talked a little bit about this already, but, go maybe a little bit deeper and sort of think about this, um, can you tell me about your, your personal practices um, with respect to using condoms, um, so, do you use them regularly, do you use them with some partners but not others, are there certain circumstances when you don't use them, um, anything you want to say about condom use in your own practices.

Speaker 2: Well, um, I, I use condoms with anal, always, um, I, I think I've used a condom once for oral but, um, and sometimes I've swallowed in the past, but, uh, more recently I have decided I don't want to do that anymore so I, I don't. Um, and since I, as I, well as I mentioned before, I didn't think that HIV could be contacted via skin to skin so I haven't used, um, condoms for just like, o- uh hand jobs or whatever.

Speaker 1: And is the, your decision not to swallow anymore, is that HIV related or is there some other factor.

Speaker 2: Um, well, it's, it's pretty gross, um, but also that you know, um, it's really it's just not, not enjoyable. But yeah also there is you know, that, that threat of if this person, you know, is HIV positive or if they have some STD, you know, don't uh, ingest that.

Speaker 1: Okay. And do you think there are circumstances where you might choose not to use a condom with someone, or is that too abstract to think about.

Speaker 2: Well, I, well I think if at some point in my life I would like to be in a committed relationship.

Speaker 1: Mm-hmm (affirmative).

Speaker 2: And at that point I would say you know, but I, when that happens, I have no idea when that will happen, so.

Speaker 1: Okay. And any other circumstances or, or is that pretty much.

Speaker 2: Um, I, I really can't think of one. Um, yeah if someone said, "Let's have sex," but they didn't have a condom and I didn't have one it's like, "Well we're out of luck," but, can't really help you on it so.

Speaker 1: Okay, um, and have you ever worried about being infected with HIV is that, um-

Speaker 2: Yeah well I mean-

Speaker 1: That one time that you told me about.

Speaker 2: Yeah, yeah that one time, that one, and then-

Speaker 1: Were you really worried that you might have got it?

Speaker 2: I wasn't, I wasn't um, well he, he had said that it was his first time, um, but I wasn't so sure about that.

Speaker 1: Okay.

Speaker 2: Um, so I, I was worried but I wasn't, I wasn't completely paranoid, but I was like well, I don't know anything about this guy so.

Speaker 1: Do you talk about HIV with your partners? Is that something that, that comes up when you're hooking up with people or-

Speaker 2: Um no, not really.

Speaker 1: Kinda hard to imagine, that, that's a hard thing to do right?

Speaker 2: Yeah, say, yeah. Yeah I don't, um, uh- uh- I mean I really should, I guess, but I, I think I would if I, I haven't had anal sex in quite a long time. Um, and, if, if it was someone who I'm having anal sex with I would be inclined to discuss it, but since I normally don't do that-

Speaker 1: Mm-hmm (affirmative), Um, and have any of your partners ever disclosed their HIV status to you?

Speaker 2: Um, well I've had people say they were negative, but I've never met anyone who was positive.

Speaker 1: Okay. Um, and you told me about, um, the HPV infection, have you had any other sexual transmitted infections?

Speaker 2: No that was the only one.

Speaker 1: Okay, um, and then, sort of how worried are you about getting HIV in the future like on a, how worried are about it?

Speaker 2: Um, not that worried because now that, now that I know that I don't have it, um, I know that I can, uh, take measures to prevent it so.

Speaker 1: Um, if you had to put it on like quantified on a 0 no risk at all to 100 very very worried, how, what would you say your chances are?

Speaker 2: Probably like 1 or 2 out of 100, yeah.

Speaker 1: Okay. Well we're at the PrEP questions finally, unless there's anything else you want to tell me-

Speaker 2: Mm-hmm (affirmative), no that's all right.

Speaker 1: About your background, experiences?

Speaker 2: Um, no, nothing else.

Speaker 1: You good?

Speaker 2: Yeah.

Speaker 1: [inaudible 00:41:16]?

Speaker 2: Sure.

Speaker 1: All right, um, so these next set of questions are about, um, pre exposure.

Speaker 2: Mm-hmm (affirmative).

Speaker 1: Pre exposure prophylaxis or PrEP which is a daily pill-

Speaker 2: Mm-hmm (affirmative).

Speaker 1: Um that can be taken to prevent HIV so, um, I'm going to refer as PrEP as PrEP and use that-

Speaker 2: Yeah-

Speaker 1: Word so it's easier to say. Um, so let's just start with some basic knowledge, um, kinds of questions. Can you tell me what, if anything, you've heard about PrEP, what do you know about it?

Speaker 2: Um, well, I know that you take it once a day, I know that, um, from what I've heard, I haven't really done my own research into it-

Speaker 1: Mm-hmm (affirmative).

Speaker 2: But from what I've heard, um, it's, it's usually pretty expensive, uh, not all insurance covers it, um, I, I heard there, there might be, uh, like long term side effects that people don't really know about yet, um, uh, what else have I heard. Um, I think that's about it.

Speaker 1: Okay, and how have you heard about these things? Like, what are your sources of information?

Speaker 2: The internet.

Speaker 1: Yeah.

Speaker 2: Um, doctors.

Speaker 1: So you've talked with your doctor about PrEP?

Speaker 2: I, I have, I talked to my doctor here, uh, like here at school about it, yeah.

Speaker 1: Okay. Is that the doctor you've gone to for the HPV treatment and followups?

Speaker 2: Yeah, uh well, well I ori- I went to her, um, to get examined but she's not a dysplasia person so she sent me to Strong Memorial so.

Speaker 1: Okay. Okay. Um, and can you tell me a little bit about how that conversation's gone, have you brought it up? Has your doctor brought it up? Um, did it feel like a, uh, useful conversation? Anything?

Speaker 2: Um, she brought it up and, um, I mean it's, it's useful to know about, um, I'm not necessarily sure I want to take it but it's good to know that it exists.

Speaker 1: Sure. Um, and what about your parents or friends or teachers or anything like that, have you discussed it with any of them?

Speaker 2: No, um, yeah no. Not like, uh, definitely not my teachers, um, I've never really talked about my, uh, I haven't really talked to my parents about personal things a lot, um, mostly because I, um, like, as, as a kid they would, they would say, uh, or I would tell them something like, I would show interest in something and they would kinda like brush it off.

Speaker 1: Mm-hmm (affirmative).

Speaker 2: And so, it's kinda been like, they, they never really showed interest in things I was interested in so I just don't tell them about things.

Speaker 1: Mm-hmm (affirmative). Um, do you know anyone whose taken PrEP, do you personally know anybody?

Speaker 2: Uh, no I don't.

Speaker 1: Um, um and, you said you haven't really considered taking PrEP yourself-

Speaker 2: No, not really.

Speaker 1: Um, um, do you have any, so what factors have effected that decision for you?

Speaker 2: Um, well I, I have no idea if my insurance would take it, um, taking a pill every day really isn't that big of a deal, um, so that doesn't really factor in, um, I don't know, really just like, I didn't really think I need it 'cause I, I really don't have sex that much.

Speaker 1: Mm-hmm (affirmative).

Speaker 2: And the sex I have isn't, as far as I know, like, high risk for HIV, so.

Speaker 1: Okay. So we've been talking to a lot of people about the factors that effect their decisions around PrEP-

Speaker 2: Yeah.

Speaker 1: So I wanna sort of ask you-

Speaker 2: Okay.

Speaker 1: Some very specific kind of questions-

Speaker 2: Yeah.

Speaker 1: And if you have more to say about your [inaudible 00:45:18] then-

Speaker 2: Yup.

Speaker 1: You know, that's fine too. Um, there's no right or wrong answer, you know, as with any of this, um, so what have you heard specifically about the cost of PrEP. You mentioned that you, something like that but what, what more could you say.

Speaker 2: I want to say that I've heard it was, uh, it, I, the numbers that come to mind are either, like $50 per pill, or I think it was $1300 a month, are the numbers that come to mind.

Speaker 1: Okay, and you got those numbers from-

Speaker 2: Uh I think from online.

Speaker 1: Okay.

Speaker 2: So- some people talking about it.

Speaker 1: So, so are you in like chat rooms or are you on-

Speaker 2: On, on, on Reddit.

Speaker 1: Reddit, okay.

Speaker 2: [crosstalk 00:46:07] website there's lots of, lots of separates for gay people talking like-

Speaker 1: Mm-hmm (affirmative).

Speaker 2: The, uh, et cetera.

Speaker 1: Talk about different topics and stuff.

Speaker 2: Yeah.

Speaker 1: Um, so in any of those contacts have you heard of any, about people having trouble paying for PrEP or what have you heard about how people manage those costs?

Speaker 2: Um, I haven't really heard anything, um, mostly just that it's expensive and I, I think some people have said like, "Oh I can't afford this," or like, "I'd like to be on PrEP but, you know, too expensive," or something like that.

Speaker 1: Again, kind of in the Reddit context?

Speaker 2: Yeah.

Speaker 1: And do you know if insurance covers it?

Speaker 2: Um, I, I think that insurance does cover it, um, but I'm, as far as I know every insurance plan is different so, um, you know, if my, if my insurance specifically covers it, I have no clue.

Speaker 1: Okay. And in the same context that you were talking before about people making comments about cost, has anyone talked about insurance in relation to PrEP that you're aware of or have you picked up anything about that?

Speaker 2: Um, not really, no, um, other than someone saying, "Yeah I use insurance for PrEP." That's really all I've seen, at least on Reddit.

Speaker 1: What about you personally, like if you found out that your insurance covered PrEP and you decided you wanted to go on it would you have any concerns about using your insurance to cover it?

Speaker 2: Uh, no, not about using my insurance. Um, I would, I definitely, would do more research into PrEP, um, like I, I believe there are some short term side effects as well, but like, I don't really know what they are, so, do some research.

Speaker 1: And then are you on your own insurance or you someone else's insurance?

Speaker 2: I'm still on my parents uh, insurance plan, so.

Speaker 1: And would that matter to you like if, if you went on PrEP and you were on their insurance is that a worry at all?

Speaker 2: Not, not really no, um, 'cause I mean, as, as far as I know they would be like, "Oh, your staying safe, good."

Speaker 1: Oh you think they would be supportive?

Speaker 2: I, I think they would, yeah.

Speaker 1: Mm-hmm (affirmative).

Speaker 2: Um, yeah I think so.

Speaker 1: Okay, um, so what have you heard, if anything, and again if you've heard nothing you can just say that-

Speaker 2: Mm-hmm (affirmative).

Speaker 1: But um, about access to PrEP, um, you know, you know where would you go if you wanted to go on it, um, do you think it's easy to get on PrEP, is it hard to get on PrEP, like, what have you heard about access?

Speaker 2: Well I know there's like, um, in [Upstate NY City 2] there's uh, there's, there's some people on Grindr that are like, they're like, uh, spokespeople for this, um, I, I don't know, it, it's like a, it's some like clinic or something, it's called [Health Center Upstate City 2], um, but so I, I think you can get it there, um, I, I haven't really talked to those people, but, um, you know they, you, I imagine you can get it there, um, any, I don't know if I could get a doctor to prescribe it, um, yeah, um I think there was a second part to that question that I didn't answer?

Speaker 1: Um, just you know, do you think it's easy or hard to get on PrEP?

Speaker 2: Oh sure, um, I, I think it's like relatively easy, um, I haven't heard like people saying, I, well I haven't heard stories either way, um, but I, I would imagine that you know if you say, "I want to be safe," they, they would be like, "Okay."

Speaker 1: Um, so what have you, if anything, have you heard about actually like taking PrEP, um, do you take it daily, weekly, monthly, liquid, pill, implant, like what are the modes, do you have to go to a doctors office, what's the sort of process for taking it?

Speaker 2: Um, well I know it's a pill, I know you take it once a day, um-

Speaker 1: Are there any other ways to take it? Besides a pill?

Speaker 2: I don't, I have no idea.

Speaker 1: And do you have to go to the doctors office, um, to get it or is there any requirements for followup or anything like that?

Speaker 2: I don't, I have no idea.

Speaker 1: Okay. Um, do you feel like you would personally be able to take PrEP if you decided you wanted to, do you think it would pose a problem for you personally?

Speaker 2: Um-

Speaker 1: To manage it, I mean like-

Speaker 2: Oh like daily-

Speaker 1: Just to manage, yeah-

Speaker 2: Taking the pill, no I can do that.

Speaker 1: Okay. Um, what do you think about if it were an injection rather than a daily pill, would that change your decision making around it in any way?

Speaker 2: Like how often of an injection? Like once a month?

Speaker 1: Wh-what matters to you?

Speaker 2: Well sure, um, I mean, if it was probably like, like once every, or like, like bi- bi annually I would, I would probably be okay with that, um, but, I don't know, going to the doctors every month to get a shot, I wouldn't like that.

Speaker 1: Okay.

Speaker 2: Um, yeah it would have to be like, you know, not very, very infrequent.

Speaker 1: Um, and what about if it was like an implant that was like under the skin somehow.

Speaker 2: Implant? Um, well-

Speaker 1: Like a time release implant kind of-

Speaker 2: No. That's, that's interesting, um, I- I'm, I'm not really on board with that.

Speaker 1: Mmm.

Speaker 2: Um, I don't know, it's, it, it's like-

Speaker 1: Can you say why?

Speaker 2: It, it just-

Speaker 1: [crosstalk 00:52:07]

Speaker 2: I'm a real, I'm a real like Sci-fi guy and, uh, like-

Speaker 1: (laughs) I was wondering if that's where you were going.

Speaker 2: Yeah that's where I'm goin', um, and so that- like an implant of like, it's not really that, that crazy, but like, like 'cause when I think of implants you know I think of like, uh, like there's, there's a show called [inaudible 00:52:30] and in one of the episodes they put, uh, they get an implant where they can, from the point that the implant's installed they have access to all of their memories.

Speaker 1: Mm-hmm (affirmative).

Speaker 2: Uh, like forever. Um, and it's, it like, you know shows all the like crazy stuff that happens. But so like, when, when I talk to people about implants that's like, terrifies me 'cause they, they have the possibility to go wrong and so like if I got implanted with like a, a time releasing piece of PrEP like what, what happens if it malfunctions and it, you know, and it releases too much, it releases too little, you know, and, and you really don't have any control over it, so if it malfunctions you're just kinda fresh out of luck, so, no, no, I'm really not on board with that. (laughs)

Speaker 1: Okay, um, all right, so let's think a little bit about, um, di- does the whole PrEP sound a little like Sci-fi-ish in a way or does it feel real?

Speaker 2: Uh, seems real enough I mean it's just like, you know, ev- we take drugs for everything so, I mean it's just another drug, at least as far as I know.

Speaker 1: Yep. Um, let's think about, um, effectiveness. What have you heard about the effectiveness of PrEP, how effective do you think it is for preventing HIV.

Speaker 2: Um, I've heard that it is effective, um, to what extent, I'm not really sure. Like, I don't, I don't really know numbers or anything about that but from what I've heard, I mean, I don't, I don't know. 'Cause like I, I've heard people like say like, "Oh yeah it's great at preventing, preventing HIV," but, uh, I guess, I guess the thing that, um, confuses me I guess is it's like, well if it gets prevented then how do you know that it was prevented, I guess? Is, is my question, but-

Speaker 1: You mean how do you know [crosstalk 00:54:42]-

Speaker 2: Like, like so would, wouldn't like the HIV still be in your system or something that, so like, in knowing that the PrEP is effective, you, you would have to know that they have HIV in their system-

Speaker 1: Like that you've been exposed to it.

Speaker 2: You've been exposed to it, but like, if you, like for example, you, you woke up with someone and you don't know if they had HIV like, and, you're on PrEP, you don't know if, if it was effective or not if you don't get HIV unless, I don't know, if it's still in your system and you don't.

Speaker 1: So are you asking a question about how they know that it-

Speaker 2: How they get-

Speaker 1: [crosstalk 00:55:20].

Speaker 2: Yeah.

Speaker 1: So they do sort of, they do it research with discordant couples where they have one person whose-

Speaker 2: Mm-hmm (affirmative). Positive.

Speaker 1: Positive and one whose negative.

Speaker 2: Mm-hmm (affirmative).

Speaker 1: Right? Um, that's when we're making sure-

Speaker 2: [crosstalk 00:55:32].

Speaker 1: That you could look at effectiveness of PrEP-

Speaker 2: Mm-hmm (affirmative). Okay.

Speaker 1: Um, so do you think PrEP's more or less effective at preventing HIV than condom use?

Speaker 2: Um, I, I would imagine it's more effective, um, solely because it's, uh, it's like a drug designed to, like, kill HIV, or I don't know what it does to HIV but it does something to it that prevents it-

Speaker 1: Mm-hmm (affirmative).

Speaker 2: So, I mean that's, that's more than just a physical barrier, um, which I would think would do more.

Speaker 1: Okay. Um, so it's, um, I'll ask you one more, so, um, how effective, a couple more questions about effectiveness, how effective do you think it would have to be in order for you to consider using it, do you have like a threshold in mind?

Speaker 2: Um, well, if, if I was, well if, if I was told like, if you use, uh PrEP and condoms all the time and there's, there's like uh, I don't know, like a super like, super tiny chance that you can get HIV, like, I, I could, I couldn't tell you a number, but like, um, for, for an example I guess, I think I've seen like, um, like 1 in it's like 1 in 1000, or something like that, when, when you use a condom, uh, or, uh, or when, when you don't use a condom, I don't know. Um, I don't really know the exact numbers.

Speaker 1: The numbers.

Speaker 2: But um, I guess if, if they said like, you, if you use PrEP and condoms to, uh, at the same time and you're diligent about it, you, like, you will not contact HIV then I would be like, okay, sure.

Speaker 1: So it would have to be close to 100%.

Speaker 2: Yeah.

Speaker 1: What about PrEP without condoms, what if they, what would, like what if you were someone who really didn't like condoms, didn't want to have to use condoms anymore-

Speaker 2: Mm-hmm (affirmative).

Speaker 1: How effective would have to be for you to consider using it.

Speaker 2: Um, if I, well, well so if I, if I was a person who didn't, didn't use condoms, um, I would definitely have like a less, uh, I would, I think, well, I, I would say that I would definitely need it to say like, well, cl- like, close to 100, even more so.

Speaker 1: If it cut your risk in half, would you use it?

Speaker 2: Um, maybe? I'm not, I'm not entirely sure, I would have to think about it.

Speaker 1: All right. Um, so let's talk about side effects.

Speaker 2: Mm-hmm (affirmative).

Speaker 1: What, what have you heard, read, about side effects?

Speaker 2: Um in terms of short term side effects I think I've heard like, nausea, um, maybe like headaches or something, um, the thing that, uh, worried me that I saw is I, I hea- I saw some study that was done or something that said like there might be like shocking long term uh risks with taking PrEP like, some, some organ damage or something.

Speaker 1: Mmm.

Speaker 2: Uh which was like, oh that's, uh, that's not great so that was something that I think would need to be looked into more.

Speaker 1: Um, which of the side effects are most concerning to you would you say?

Speaker 2: Um well, I think that, well obviously like organ damage is definitely a big concern for me, um, but like even, even if I'm taking it daily and it makes me nauseous um I really wouldn't want that, um, I definitely wouldn't want something that, you know, I'm taking it to not, obviously not get HIV, um, but like, getting, being nauseous all the time, having headaches all the time, its not something I'd want as well, so.

Speaker 1: Um, so, lets talk, um, about how you think friends or potential partners look at people who are on PrEP. Do they think that, do you think people look at people on PrEP positively, um, favorably, or negatively, what is your sense of how they're, how PrEP is viewed.

Speaker 2: I mean from what I've seen it's pretty positive, um, 'cause there's really no, I don't, I don't see any reason to be negative about it. You know, um, they're just trying to be more safe. Um, yeah I don't, I don't think I've seen anyone be like, "Oh you're on PrEP, uh, you're a terrible person," or something like that, but, yeah I've only see- I've really only seen positive things.

Speaker 1: Would you tell someone- sexual partners that you were on PrEP, if you were?

Speaker 2: Um, I mean I don't see why not, like, it's good to know I guess.

Speaker 1: Mm-hmm (affirmative). Um, and would you expect your partners to tell you if they were um-

Speaker 2: Um, yeah probably.

Speaker 1: So you would have an expectation.

Speaker 2: I- I mean, well, I guess I wouldn't expect them to tell me but I, I would probably ask. Um, like, like if I, if I, if I was on PrEP I'd be like, "Hey, I'm on PrEP are you on PrEP?"

Speaker 1: Mm-hmm (affirmative).

Speaker 2: Um, but like I g- I guess, um, I guess that might be a good thing to ask anyways.

Speaker 1: Um, so if a partner disclosed that he was on PrEP to you-

Speaker 2: Mm-hmm (affirmative).

Speaker 1: Would it, um, how would you feel about that?

Speaker 2: Um, I wouldn't have a problem with it, really, like it's not, um, it wouldn't like, it re- it really wouldn't make a difference to me, um.

Speaker 1: Would it matter if it was a new partner or a casual partner or, um, a regular sexual partner, would any of those, that context change anything for you?

Speaker 2: Well I guess if it was like uh, like a new, new person that I've met, um, and they say, "Oh, I'm on PrEP," um, I don't know, I guess one of the things that I would think about is like, "Oh, like if he's on PrEP, does he have sex a lot?" Like, uh, like, like an obscene amount, or, something like that because, I guess that's anoth- another reason you'd want to take PrEP is, you know, if you're, like, if you have a lot of sex an, and you wanna reduce your risk, that's what- one way to do it, so.

Speaker 1: And, would that, would that matter to you if your partner was having a lot of sex?

Speaker 2: No, not really, um, I mean the, the one thing is if they're having, you know, as long as like, they, they don't hook up with like sleazy people, you know, but obviously I don't know that, so that's, that's, that's one of the, uh, hard things about hooking up is it's like, you don't really know what other people have been with.

Speaker 1: Mm-hmm (affirmative). Um, and would you worry if you told other people that you were on PrEP that they might change their perception of you in that regard?

Speaker 2: Um.

Speaker 1: Or make assumptions about you?

Speaker 2: Yeah, I, I guess, I guess that, um, I guess that, I, since I make those assumptions I guess I would imagine other people do as well. Um, so I mean yeah, it's, and like I guess they, they're free to make those assumptions but it's, I guess it's something you'd have to deal with.

Speaker 1: Um, all right. Um, so what we have here, anything else you wanna say about PrEP just from your own sort of, experience or knowledge or-

Speaker 2: I don't think so, no.

Speaker 1: Okay. So, what I have here is um, sort of a fact sheet-

Speaker 2: Okay.

Speaker 1: So take a minute, you can keep this, but take a minute and read through that and then I just have a couple questions once you sort of, digest that, a couple questions.

Speaker 2: Mm-hmm (affirmative).

Well um, now that I've read this, uh, it's pretty interesting, um, just a lot of the information. Like I didn't, I didn't saw like the, the kidney problem. I didn't know that they went away after you stopped taking it so that's interesting.

Speaker 1: Mm-hmm (affirmative). So do you have any other thoughts about, now that you see these kind of facts about, do you have any other um questions or comments or does this change your thinking about PrEP in any way?

Speaker 2: It definitely seems like a better option, um, like now so like, uh, same with like the, the short term side effects, like the fact that they go away, um, after a week of taking it is, it's fine, um, and it really just, it seems like, it seems like a better option.

Speaker 1: Than you thought it was?

Speaker 2: Than I thought it was, yeah, sure.

Speaker 1: Any other thoughts, reactions, um.

Speaker 2: Um, not really, um.

Speaker 1: To what extent you think people in the gay, bi, trans community know these facts about PrEP, do you think this is common knowledge, do you think this is not so common knowledge, any sense of that?

Speaker 2: I think that they, mmm, I don't think they know, like, all of this. Um, I would, I would imagine that they would know like it stops HIV, um, I, like, I wouldn't imagine they know the, the, um, the risk, the risk reduction percentage that I was given here, um.

Speaker 1: Does that surprise you?

Speaker 2: Yeah, that does surprise me. That's very, that's really good. Um, I, I don't imagine I- if- if- like for example, you know, reading about these side effects, um, like online when people like talk negatively about things they're not gonna say, "Oh this is terrible, but if you stop taking it goes away," they're just gonna say it's terrible. So, I mean like, like I didn't know that those things went away, so.

Speaker 1: Mm-hmm (affirmative). Does that change your thinking about PrEP for yourself in anyway, or is it, I'm not putting you on the spot-

Speaker 2: Yeah.

Speaker 1: I'm just, uh, um, just, just curious.

Speaker 2: Definitely, um, like I still don't think I w- I really want to take PrEP right now, but I think if in the future I was considering it, I definitely feel, would feel better about taking it.

Speaker 1: Okay. Um, so I really appreciate your taking all this time, we've been talking for a while so-

Speaker 2: Mm-hmm (affirmative).

Speaker 1: Taking this time to talk to me so I've got a few other, um, things, a couple short exercises in this self administered questionnaire but we're getting towards the end here.

Speaker 2: Mm-hmm (affirmative).

Speaker 1: Um, these next things that I'm going to ask you are a little bit different than what you've been doing.

Speaker 2: Okay.

Speaker 1: But um, hopefully they'll, they'll just take a few minutes and they'll make sense. So, let me start by giving you this sort of cards. On each card I've written um, something that um, people tell us matters to them in making decisions about whether they're gonna use PrEP.

Speaker 2: Okay.

Speaker 1: So this is a ranking exercise, we're asking you to sort them out-

Speaker 2: Okay.

Speaker 1: Just putting that in front of you and then put them in the pile with the most important factors for you on top and then kind of the ones ranked below that.

Speaker 2: Mm-hmm (affirmative).

Speaker 1: If something that you think matters isn't there, we've got some blank cards that you can write your own thing on right?

Speaker 2: Mm-hmm (affirmative).

Speaker 1: So just stack them up and once you do we'll read through them together so that we see what the order is and whether you have any comments or thoughts about um, how you would rank this in decision making.

Speaker 2: Mmm.

Speaker 1: Kind of from most to least important.

Speaker 2: Yeah. (pause)

I think that's uh, that's how how I'd rank it.

Speaker 1: All right let's look, let's look, if you want to add anything?

Speaker 2: I don't think so I think these are all pretty um, good, uh, these are like the concerns I would have about PrEP so-

Speaker 1: Okay.

Speaker 2: I don't think I have any more.

Speaker 1: Was this hard to do or easy to do?

Speaker 2: Um, for some of them it was kind of hard to decide, um, like, uh, like I, I think definitely um, effectiveness is the most important thing for me. Um, but then like, when it comes to like uh, how, how often I have to take it, uh, how easy it is on a stigma, I think those are like, three things I was unsure of where to rank them the most. Um, side effects are important, um, you know-

Speaker 1: So just to be clear, you ranked it effectiveness, side effects, how often you have to take it, ease of access, stigma, cost, and how you take it, whether it's a pill, an injection, et cetera et cetera.

Speaker 2: Yeah.

Speaker 1: So that's the least important for you, effectiveness and side effects are the two most important.

Speaker 2: Yes.

Speaker 1: So, cost is pretty low, what, can you tell, talk with me a little bit about why cost is low in your, um-

Speaker 2: Well, um, to me it's like, it- it's still, I don't know, it, it's not like, I think, I think they're all, aside from how you take it, I think these are all still very important, um, in my decision. Like there, there's a big, a big difference in between cost and how you take it in terms of like, importantness ranking, if you know what I mean.

Speaker 1: Even with the one un- under the skin there?

Speaker 2: Okay well, I mean like-

Speaker 1: (laughs)

Speaker 2: I, no I wouldn't do that but um.

Speaker 1: (laughs) I'm just teasin' you.

Speaker 2: Yeah, yeah. But like for example, like effectiveness is- like I'd rank it at like 100 and then you go like, 95, 90, 80, 75, 70 and then like, like 20.

Speaker 1: So from cost to how you take it is like a huge jump.

Speaker 2: Yeah, I- 'cause like that's, to me, I don't really care. Um, but like the cost, the cost is definitely important, um, 'cause you know, I, I don't, did it say anything, it said it's covered by insurance. But so like, if it is like expensive, you know, that, that is important, 'cause if I can't afford it then I can't really take it. Um, but, you know.

Speaker 1: So for you, this is the order that you would put these in.

Speaker 2: Yep.

Speaker 1: Any other comments about the ranking or the ease or difficulty of doing this or any other particular dimension that's come to mind?

Speaker 2: Uh no not really. Um, I mean, obviously, the, well I'm- not obviously, but the thing, I mean as I- as I just explained like the, the difference in between these are so like different that using like index cards to rank them, while it does rank them it doesn't show the difference between.

Speaker 1: So in some ways, effectiveness and side effects are kind of top for you-

Speaker 2: Mm-hmm (affirmative).

Speaker 1: And then these other, how often you take it, ease of access, stigma, and cost are kind of in the middle-

Speaker 2: Yeah [crosstalk 01:13:48].

Speaker 1: Other ones kind of at the bottom.

Speaker 2: Yeah.

Speaker 1: If I understand what you're saying.

Speaker 2: Yeah.

Speaker 1: I'm not trying to put words in your mouth I'm just trying to understand.

Speaker 2: Yeah that's basically it. Um, yeah 'cause the, these are extremely important to me and then, these are very important, and then not important really at all for the how you take it.

Speaker 1: Okay.

Speaker 2: So, yeah.

Speaker 1: All right, all right I'll take the cards back and use them another day. Um, so the second exercise is, I give you this.

Speaker 2: Okay.

Speaker 1: So take a look at this table, right, um.

Speaker 2: Mm-hmm (affirmative).

Speaker 1: You can just and then I'll ask you to circle the row that [inaudible 01:14:28] you so, imagine that I'm going to flip a coin, heads or tails, um and you will get what is indicated in the heads column if it comes up heads and you will get what is in the tails column if it comes up tails.

Speaker 2: Mm-hmm (affirmative).

Speaker 1: Which row of the table would you pick? You can only pick one. So just think about it and then circle the row and-

Speaker 2: Mmm.

Speaker 1: Tell me a little bit about why you made the choice that you made.

Speaker 2: Mmm. I would circle row two here. Um my, so, I do, um, like, so I, I play a card game called Magic: The Gathering, um, and, in tournaments you, there's, if you, if you get to the top and you're gonna win prizes a lot of the time you discuss like a prize split with your opponent. And so th- to me I was thinking about this like a prize split.

Speaker 1: Mm-hmm (affirmative).

Speaker 2: In the way that it's like, so if I, if I, if I do row 1 where it's 50/50, it's pretty good, I can make 50 either way. If I do row 2, uh, I either get 100, that's really good, or 30 which is, you know, it's pretty okay. And then 200, it's a lot of money, but I could also get $10 which is-

Speaker 1: Mm-hmm (affirmative).

Speaker 2: That would, I wouldn't feel great about getting $10, and same thing for row 4 where it's, you just get nothing, which I definitely wouldn't want, so, um, with, with row 2 I know I'm definitely going to get something and I wouldn't be disappointed with either result.

Speaker 1: Cool, excellent. Um so I'm gonna take that back from you.

Speaker 2: Mm-hmm (affirmative).

Speaker 1: So the last thing that um, I have for you to do is our self administered questionnaire so um, you can see your name's not on it, it's just the ID number, I'm gonna ask you to go ahead and fill that out and just put it into this envelope when you're done.

Speaker 2: Okay. (pause)

Speaker 1: Okay. Thank you. Just slide it into that envelope. Um, so just thinking back over the conversation that we've had, the interview, um, is there anything that you want to add or any questions you had, I mean thinking back to your childhood all the way through HIV and AIDS, PrEP, your sort of coming out experience, is there anything you feel like, you didn't cover that you want to or that you wanted to say at the end of the interview or final sort of statement, anything?

Speaker 2: Um, not really, I thought, I think this was very interesting in terms of, uh, learning more about PrEP and, uh, I'm glad I was, I'm part of this study, so.

Speaker 1: Well I'm really grateful for your taking your time to do this and being so willing to talk about some really-

Speaker 2: Mm-hmm (affirmative).

Speaker 1: Personal things and, um, trusting us to be, um, good stewards of the information you've given us, um, we're talking to lots of people and we're gonna analyze the data and, and, and try to make a contribution to the scientific literature on-

Speaker 2: Mm-hmm (affirmative).

Speaker 1: On, um, PrEP, um, and, of course we'll respect your confidentiality in doing that, um, that's very important to us, um, we're still enrolling people in the study so if you know of other people-

Speaker 2: Mm-hmm (affirmative).

Speaker 1: Who might be interested please feel free to give them our contact information, um, uh, they can email-

Speaker 2: Mm-hmm (affirmative).

Speaker 1: How did you hear about the study?

Speaker 2: I found, uh, I found flyer on campus.

Speaker 1: Yeah, good, okay. So yeah, if um, you know, if you know of other people point out the flyer to them and encourage-

Speaker 2: Mm-hmm (affirmative).

Speaker 1: Them to contact us, we'd be happy to, either me or one of the other interviewers on the team would be happy to come out and-

Speaker 2: Mm-hmm (affirmative).

Speaker 1: Talk with folks, um, so, you've got the consent form there, this is the fact sheet for you, we just give this to everyone, um, here's some resources in [Upstate NY City 2]-

Speaker 2: Mm-hmm (affirmative).

Speaker 1: In terms of PrEP providers, counseling services, just some community services just, information sheet in case you ever need it.

Speaker 2: Mm-hmm (affirmative).

Speaker 1: Um, for your participation there's $40.

Speaker 2: Mm-hmm (affirmative).

Speaker 1: And we just ask that, um, you acknowledge receipt so write in the $40, the date, and then print and sign your name.

Speaker 2: 28th. 28th? 20- yeah 28th.

Speaker 1: It is the 28th.

Speaker 2: It is the 28th. Tomorrow a new game comes out that's very exciting.

Speaker 1: Oh, there you go. Do you need this paperclip for those papers or are you good?

Speaker 2: No I'll be good, I'll throw 'em in my back pack.

Speaker 1: Okay. Um any other questions before we close up?

Speaker 2: Nope.

Speaker 1: Excellent. Again thanks, um, thanks so much for participating in this study.

Speaker 2: Mm-hmm (affirmative).

Speaker 1: It's really, really great.

Speaker 2: Yup.

Speaker 1: I'll go ahead and turn off this tape recorder now.

IDM 110

S1 50:19

So what are some ways that you can prevent the spread of HIV?

S2 50:24

Well, there is obviously abstinence. You could just avoid it entirely. There is always talking with your partner, "Hey, I want to be honest before we start. Have you been tested? Are you clean? Are you anything like that?" And if they say, "No, I'm not clean," or if you don't ask, it's always safer to err on the side of use a condom so that the fluids don't intermingle with each other, openings won't be introduced with the said fluids. But besides that, I have not used nor do I really know of any other way.

S1 51:01

Okay. How about treatment options for HIV?

S2 51:05

So I know that there are places which will do free screenings, free testings for HIV. I also know that obviously a doctor, physician can do that for you as well. In terms of treatment options, I believe that there is medications out there that treat, but I don't know if there's any that have a cure yet. Besides that, not too familiar with the idea of what medications do you what and for where, for who. But I do know that there is medications that will treat HIV/AIDS and other diseases, sexually transmitted or not.

S1 51:41

So you mentioned briefly that you've seen some stuff in your news Flipboard. But are there other forms of media where you've seen conversations about HIV or AIDS, like so movies, TV shows?

S2 51:56

Oh, there's movies. TV shows, maybe one or two that just kind of touched on it real quick. But I never tend to trust those sources just because a lot of them can be dramatized, dramaticized for the viewer's pleasure, for things like that, and as well as for good ratings. So I try not to trust things like that. If it's part of a TV show or movie, I just say, "Oh, this is probably a fictional situation," or whatever. I do know, obviously, it's a real thing that does happen to real people. However, the information that's being brought up in this movie or TV show is probably not the most reliable to be listening to. So I just try not to listen to it.

S1 52:37

Okay. Have you talked to your current partner, pseudo-partner?

S2 52:43

You can just say partner for the [experiences?] that we're having here.

S1 52:45

Okay, sorry.

S2 52:46

No, you're fine. It's just to make it easier on you. Have we talked? Yes. So after our first time together, I had messaged him and said, "Hey, I was just thinking about this. Have you been tested for HIV/AIDS? Are you clean?" And he had said, "Yes, I get tested before every semester, and I had not had any [real?] sexual relations since then. So I know that, yes, I am clean." And I had messaged back, "Okay. That's great, however, even continuing onward, I think I do want to continue using a condom just because different things could occur. You could have relations with other people that I may not know about. And because it's up to you to tell me about it or not, if you want to keep it a secret, I would just prefer using a condom, because I don't want to become infected, or infect you if I have a situation like that where I find a sexual partner besides you and then don't tell you about it. So I do want to have that honesty between us. So using condoms is probably the best way to protect ourselves and trust one another that we would never be diagnosed from each other."

S1 53:54

So how did he react to that conversation?

S2 53:57

He and I are pretty much-- we think alike a lot, so he definitely agreed. He said, "I think that's a good idea. I'm very fine with using one. I have no problems, no reservations about it. So, yeah, no problem."

S1 54:11

Okay. I wasn't sure if maybe there would be some tension from the idea that you'd have other sexual partners possibly.

S2 54:18

Because the relationship is where it is right now, we both know that it is an option that is on the table because we had specifically said to each other, "Hey, this isn't a relationship right now. It could be in the future, but as of right now because of how we are taking things because of the old relationship I was in-- not I, with him, it would just be better to have a relationship like this, and if it continues, then we will monogamous. We will just have he and I. But for right now, it could err on that side. So we're both on the same page with that.

S1 54:58

Sure. Did you ever talk with either female partner about being worried about HIV or STDs?

S2 55:06

So, no. I know that both female partners I had had were never-- it was their first time, with me, so I knew that they were clean because they were obviously very nervous about it. They were asking a lot of questions and things. And because I was younger and I really hadn't thought about it much, it was just kind of, oh, the teacher says so, like whatever, who cares. It was never anything that I had really thought about, no.

S1 55:37

Okay. So I think you've talked about this a little bit already. But so every time you've had sex, have you--

S2 55:45

Have I used--

S1 55:45

--[has there been?] a condom?

S2 55:46

Yes. Every time.

S1 55:47

So every time?

S2 55:47

Mm-hmm.

S1 55:48

Is there ever a difference between, say, anal sex and oral sex?

S2 55:53

So, that's not a weird question. It's--

S1 55:58

I don't know if I phrased it--

S2 55:59

Yeah. I'm trying to think of the best way to answer that. In terms of difference, I mean, I'll always have one on for either anal, oral, vaginal. I'll just have one on just for simplicity sake. Having said that, there's definitely an order of operations in terms of what goes where first. But besides that, I mean, I put one on at the beginning and it stays on until the end, so.

S1 56:25

So even for oral sex--

S2 56:26

Yes--

S1 56:26

--you use condoms?

S2 56:27

Yep, even for oral sex. We have tried a flavored condom once or twice, but there is always a condom, yeah.

S1 56:33

Okay. Do you think there might ever be a circumstance or situation in which you wouldn't use a condom?

S2 56:41

Yes, I believe that if a relationship with this gentleman or otherwise gets to the point where I fully trust him, he fully trusts me, we have both been tested semi-recently, we both know that we are both clean, and it is monogamous, there is no intermingling with other people, there is definitely a possibility where nonprotected sex can occur. If it's with a girl-- so if I'm having a relationship with a female, I will always use a condom just for the pregnancy sake, even if they say they're on the pill, even if they say this or that, they're infertile, whatever, just erring on the safety side, unless I want to have a child. And that will come in time. So there's a few different ideas of whether or not I will, but basically, if it's with a guy, if I trust him and we've both been tested, yes, there's a possibility. If it's with a girl, if I trust her, we've both been tested, I want a baby, then yes, maybe.

S1 57:43

Okay. Have you ever been worried that you might have contracted HIV?

S2 57:48

Yeah, so the first time I had met this gentleman, and he and I had sexual relations, because I knew that he wasn't a virgin, there was probably about-- it was that morning, and then I had messaged him later that evening before work. So it was a good span of maybe five hours, maybe even six, that I was kind of nervous that I might have contracted it. But once he explained everything to me that evening, I was fine. So no. I mean, that five hour span, but, yeah.

S1 58:24

So for that five hours though, what was kind of going through your mind?

S2 58:27

Just a lot of various, just kind of stressed out, going wow, if that happens-- wait, did he have-- I never asked him if he was clean, and I know this wasn't his first time, so obviously I used a condom, so I should be okay, but again, something different might have happened. Something could have occurred. Oh, man. It was really just kind of questioning myself that, wow, this happened, but maybe something could have happened anyway, so I better check. I better check. So then I had asked him that evening.

S1 59:05

Okay. So other than HIV, have you ever been worried that you might have contracted a different sexually transmitted infection like gonorrhea, chlamydia, or--?

S2 59:14

Again, all those were kind of going through my mind during that five hour span. Just sexual STDs in general were on the mind that day.

S1 59:27

So in the future, how worried are you about contracting HIV?

S2 59:31

So considering that when I make a promise to myself, I'm definitely one to stick with it, I know that I have promised myself to ask and be honest with people, use a condom, things like that. I'm not incredibly worried that I might get contracted with the disease or I might contract someone else with a disease. However, it is always a possibility. There is always some chance that the condom breaks or that we are-- I trusted him or her and they were untruthful about their answers to me. So there is always that kind of hint of worry in the back of my mind, but taking the steps that I have and using them, applying them, I believe I should be okay. But you never know.

S1 60:19

So if you had to put that on, say, a numerical scale going from 0 to 100, where 0 is literally never going to happen, absolutely impossible to contract HIV, and 100 is absolute certainty, like inevitable, it will happen, where would you put your risk of contracting HIV?

S2 60:38

My risk or my feelings about being at risk?

S1 60:44

Would they be different numbers?

S2 60:46

They would be. So I believe that my actual risk of being contracted is somewhere maybe around a 10 or a 15 because I'm using a condom, because I'm open about it, because I'm being sure that they're being tested and I'm being tested, I feel like it is more somewhere on the lower scale. However, my worry about it, my thinking that, "Oh wow, that could still happen," is probably somewhere around the 25 to 30 on that scale just because it's better to err on the side of caution, right. And that's kind of the mindset I have.

S1 61:18

But they're still somewhat relatively minor?

S2 61:20

They're relatively minor. Yeah, it's definitely close, but there is a number difference. Absolutely.

S1 61:25

Okay. Good. All right. Great. So this next set of questions that we're going to get into, and it's the last set, are about HIV pre-exposure prophylaxis.

S2 61:34

Would you happen to have the time on you by chance? I do have an exam later.

S1 61:38

It's 2:38 right now.

S2 61:39

Oh, we have so much time. We're good.

S1 61:40

Yeah. Okay. What time do you need to--?

S2 61:42

It's 3:30 is the exam. So I have this room booked until 3:00, and we can stay here until 3:00.

S1 61:47

All right. Well, there's just this one last section and then we'll do a couple minor exercises.

S2 61:53

Yeah. Absolutely.

S1 61:54

So, like I was saying, this is about PrEP basically, pre-exposure prophylaxis. And the questions that are going to come after this, it's really just to see what you know about PrEP. So I'm not going to be telling you if you're right or wrong about it. It's just kind of trying to get a sense of what you know, all right? So to start of, can you tell me what you know, if anything, about PrEP?

S2 62:18

So I first learned about PrEP when I had seen the advertisement for this study. I had never heard of it. I had never really [taken?] any thought about it. And honestly, because of how these past few weeks have been going, trying to get tests and classes and things, although I've been thinking about it, I've never done any research on the medicine itself. So I can't say-- I know literally nothing about it. I don't know if it's a pill or a shot, or what its effects are, what its side effects might be. I have no idea, no.

S1 62:57

Okay. So do you know anything about who PrEP is for?

S2 63:01

I believe, based on the survey that we have that you've administered, I have a feeling that it's for male, or male to female transgendered men, who have sexual relations with other men. However, that is just the scope of this survey. So it could be very well for anyone who has this concern above the age of 18 or whatever, or it could be limited to just male or male to female transgender clients. So I don't know, no.

S1 63:38

Okay. So you haven't done any research on PrEP itself?

S2 63:44

I have not. I have considered it. Not just considered it, but I have had plenty of time to say, "Maybe I should look this up." This sounds like an interesting idea. It sounds like something that could very well be important for people in my situation to have or at least take as a precaution. But I never actually sat down and researched it.

S1 64:07

Is it something that's ever come up, like in your sex ed class in high school?

S2 64:12

No. Our sex ed class did a good job of kind of scaring us away from sex, ironically. It was never more-- it was never any, "This is how you need to go about it safely." It was more of a, "You shouldn't do it until you're this and that," and then, "It's not a bad thing, but it's a taboo, and it's weird, and it's this." So the idea that what drugs are used to treat what was never [inaudible]. It was more just, "HIV is bad. Don't have sex unprotected." So.

S1 64:47

So any conversations about it with your doctor or anything?

S2 64:51

No. However, I will say, I haven't seen my doctor in far too long. So maybe when I go to get tested, I will bring it up, and that's actually probably what I will do.

S1 65:00

Your partner right now, has there ever been a conversation about PrEP?

S2 65:06

No, never been any conversation about it really. Never had any second thoughts or questions about it, no.

S1 65:13

Do you know if he knows anything about PrEP or has considered taking it?

S2 65:16

I know that he has considered taking medication for an HIV. Again, I know he is clean, but more as the precaution thing and all that. So we've talked about it once or twice before that maybe it wouldn't be a bad idea, but it's never accumulated into a serious conversation. It's more of like a, "Oh, have you ever thought about doing this?" "Oh yeah, maybe," and then we kind of moved on from it.

S1 65:43

How about with either of your friend groups, especially maybe the one that's more homosexual?

S2 65:49

Homosexual-leaning. I think there might be one person in the homosexual friend group who might already be taking it but I've never questioned it. Again, I respect his space. I respect his decision to tell me about it or not. So I never really inquired, but I think there's one person who actually does take it in our friend group.

S1 66:13

So what makes you think there's someone taking it?

S2 66:16

Just, again, we all have-- there's a group chat for the group and there's probably a good 20-- no, maybe 10 people in the chat, so it's just a constant flood of messages back and forth at different times. And as I scroll through to catch up with everything, I will see the PrEP name come up here and there, and I'll go in and I'll read the message that it's about, and it's usually, he says, "Oh, I need to take it. I need to go back and get more," or whatever, so. But besides that, he doesn't ever go into any more detail about it and I don't question it.

S1 66:57

Sure. So other than this one friend who might be on it, do you know anyone who's taking PrEP?

S2 67:03

Besides him, no.

S1 67:04

Okay. So since you don't know much about PrEP, have you considered taking it?

S2 67:12

I would have to do more research to answer that question fully. However, if what it is, is a precautionary HIV medicine that you can take without having the illness, then I might be interested in taking it, again, just as the precautionary side of things. However, again, I would need to at least sit down, spend a good afternoon, maybe two hours or so, reading on different sources that are published and at least trying not to be too one-sided about the issue. I would want to get as many sides of the issue as I could before I'd do anything about it.

S1 67:57

Sure. So if you were thinking about taking it, what might some of the factors be that would be important for your decision-making?

S2 68:05

So if I were to take the drug as it is right now, so still being closeted, still all the information that I've talked to you about, it would have to be relatively discreet. If it's a shot, I would have to be able to schedule a time where I could leave my home, get the shot, and then return without being noticed by my mom or whoever. I would have to-- if it's a pill, it's a lot more manageable because I'd be able to hide it somewhere and take it every morning before my family wakes up. If it's another form, again, it would have to be relatively discreet. Obviously, cost is a factor. I am a college student. I pay for a good chunk of my tuition here. And so it would need to be affordable in that terms. It would have to be-- the side effects would have to coincide with things I don't have. So my family has a history of heart disease, my mom's side, so I would want to make sure that the drug doesn't have some kind of heart disease related issue. I would have to make sure that certain factors with blood sugar or weight gain or weight loss would have to be thought about because I am on a special diet just for weight loss right now. And it's working really well, but I would want to make sure that, okay, I'm taking this drug. It says weight loss is a thing. Let me talk to my dietitian. Let me make sure that I have my diet set up so that it compensates for that. Or weight gain's an issue, etc., so let me do the same thing. But yeah, the main points would be side effects, availability, and discreteness.

S1 69:58

Okay. [Good?]. So I want to ask you about some things other people have told us are important for them when they think about taking PrEP. So you've mentioned availability, discreteness, cost, right? So these are some of the other factors that people have kind of come up with or have suggested matter to them. So it's going to be asking, "What do you know about these factors?" So about PrEP itself. So it's perfectly fine if you just want to say, "I don't know anything," or just, "No, I don't know." Just answer as fully as you can if you do know something about it, but otherwise, just you can say no or that you don't know.

S2 70:35

No problem. No worries.

S1 70:37

All right. So what do you know, if anything, about the cost of PrEP?

S2 70:41

Again, I don't know about the cost. I know that there are prescription drugs, certain ones, that are blown through the roof in cost, especially if there is money to be made there. This whole thing with the insulin shots, where it increased, what, like 100, 200, 300, 500 percent or something like that. So I know it can be incredibly expensive, or it could be quite cost manageable. So I don't know.

S1 71:11

Do you know anything about if insurance plans will cover PrEP?

S2 71:16

No, I don't know much about insurance and what they will and will not cover.

S1 71:21

Are you on your mother's health insurance or--?

S2 71:25

So I have a health insurance that's actually my dad's plan. He still has us-- I believe he still has us as dependents there. So I am on my dad's health insurance plan. I can't remember what it's called. I think it's [Amica?], but yeah.

S1 71:41

So being on your dad's plan, if it did cover the cost of PrEP, would you feel comfortable using that?

S2 71:48

That does bring up a good point in terms of discreteness. If the insurance company has this document that says I'm taking PrEP, and somehow that information is brought to my dad's knowledge, or brought to the knowledge of other people, or affects the cost of-- well, actually, if the information that I'm taking this drug gets to my dad, I would want to say no. I'm not too worried about the cost going up. If I was to take this drug and the insurance company kept it under wraps that I was taking this drug but they did bump the cost, I wouldn't have too much of a problem because my dad is a little bit better off. He does have a little bit more money, and I know he's in a more financially stable state so he'd be able to afford it. However, just the idea that me taking this drug could get back to him could definitely have an impact there.

S1 72:45

Sure. So what have you heard, if anything, about where you can go if you want to get PrEP?

S2 72:52

I would assume obviously your doctor, physician. Maybe certain, what do they call it? The places that do sexual-related consultations, things like that, pregnancy tests, all that. I have a feeling that those would be places that I would go, at least feel comfortable going, if I were to take the drug.

S1 73:12

Sure. Do you think it would be easy to get on PrEP or to get PrEP if you wanted it?

S2 73:17

By the way the-- from the things I've heard and from the people who I have a general idea, I think that as is with any drug, after you ensure that the side effects and everything follows what you need, it should be a relatively simple process.

S1 73:35

True. So what have you heard about taking PrEP? Do you know anything about the frequency, the form, how often you see a doctor while you're on it?

S2 73:46

I actually have not heard much about that at all. In fact, I don't think I've heard anything about how it is administered, or what precautions need to be taken after taking it, whether you need to be in the office for a half hour to make sure to monitor your progress, or this or that or whatever. I'm not sure, no.

S1 74:06

So say it were a pill, an injection, or an implant that you could have under your arm, is there one that would be preferable to you?

S2 74:16

The injection would be not as preferable, not because I have a problem with needles or anything, but just because availability becomes a little bit harder. I would probably prefer the pill but the implant sounds like an interesting take on it. Sounds like something that I would need to think through. It's a good idea where you put it in once, and then you monitor of course with a doctor, but once it's in there, it's in. However, you're also putting something in your body. So that would be kind of [an?] iffy thing. So if I was to rate them best to worst, it would be pill, maybe implant, injection maybe last, yeah.

S1 74:55

So say if it's a pill, if it were a daily pill, weekly, or monthly, is there one that would be better, or--?

S2 75:03

I would be open to any of the three. Frequency really isn't a problem. I would like to have as little of it on my person as possible. So maybe a monthly or annual thing would be better. However, if scientists were like, "Okay, you need to take this daily to get the full effects," then you need to take it daily to get the full effects. That's no problem.

S1 75:25

So what have you heard about the effectiveness of PrEP?

S2 75:29

I've heard from a friend who has messaged on the group chat that it's effective, that he hasn't had any problems with it. Through his sexual encounters, he doesn't have any sort of real issues or real big problems, so I feel like it is at least relatively effective. But again, I also know that he's not having sexual relations with any person that he sees that may or may not have AIDS, so he's usually pretty reserved about it as well. So he could have just not come across anyone who's had AIDS yet. So who knows? I don't know.

S1 76:07

So how effective would PrEP have to be for you to consider taking it?

S2 76:12

Probably towards the 80% effective scale. I would want it to work most of the time. Obviously, the 99.9% effective, as much as that's the goal, I know it can be difficult, especially for medication, to reach that. And each person reacts to it differently as well. So it wouldn't be so much as how effective is it in numbers, it would have to be how effective is it for me personally? Is it degrading my health? Is it actually helping me fight off HIV/AIDS or other sexually transmitted diseases? Or is it just kind of sitting there being stagnant? Like what's happening with me?

S1 76:52

Sure. So on a similar note, what have you heard about the side effects of PrEP?

S2 76:56

Again, I've heard that they're mild. I've heard that it's really-- at least for the one friend who takes it, he hasn't had any problems where it has degraded his health or anything like that he feels nauseous or anything. It's usually just, "It's time for me to get my next dose," or whatever. And he's always said it's not a problem, so.

S1 77:21

Are there any side effects that you might be potentially worried about? I think you mentioned this a little bit already about blood sugar, weight, kind of thing?

S2 77:30

So yeah, the heart health, the blood sugar, the weight, all that is important. But those are things that I could potentially get past if I were to take other medications in order to help balance those, or if I were to just continue on my path, exercising, dieting, [things like that?] in order to help myself stay in a good standing. Those are all relatively overlookable. Really, the heart health is the biggest one because I do have a history of heart disease in my family, and I would like not to have that happen to me [laughter]. But also the fact that if it degraded my white blood cell counts, or if it kind of helped attack my immune system, I might be a little bit more limited on taking it as well just because I've seen, through my mom, that she has-- she kind of has problems with her immune system right now. So she does steroids and things like that, and it's kind of taken its toll on her. But yeah, so immune system and heart health would be the two big ones that would kind of be a no-go for me.

S1 78:40

Okay. So then, do you think friends or potential partners would look favorably or badly on taking PrEP?

S2 78:49

So with the partners I've had, and how we talked each other, and how open we are, I think if one of us were to say to the other, "Hey, I'm taking a medication which helps defeat or it helps attack or whatever sexually transmitted diseases, it might be at first, "Oh, does that mean you have a sexually transmitted disease?" in which case I would be open and honest. However, if the answer was, "No, I'm just taking preventatively," I think it could be definitely almost a positive thing where they can see it as, "Wow, he's taking steps towards his physical health and towards his sexual health. It's something I should look for in people. So I think it would be a positive experience hearing that, "As a preventive measure, I'm taking this drug."

S1 79:40

Sure. So if you were taking PrEP, would you want to tell your partners about it then?

S2 79:45

Yes, yeah, no question.

S1 79:47

So why would you want to tell them?

S2 79:49

Just, again, being open and honest is very important in a relationship, and especially something that could affect them sexually or otherwise. Plus, I couldn't find a reason why you wouldn't want to say something, especially if you were already diagnosed and began taking PrEP because you were diagnosed, it would be very important for you to tell them. But if you were taking it preventatively, there's no reason not to say, "I'm taking [PrEP?]."

S1 80:15

So then you would expect your partners to tell you as well?

S2 80:18

I don't have the same expectations from my partners because I know each person reacts to different things differently. Would I like them to tell me? Yes, I would like to know. However, if they don't, I would respect their privacy and say, "Okay. It would've been nice to hear it from you other than someone else, but I'm okay with you taking it. I understand the idea behind it." And, of course, I would ask them, "Does that mean you are currently with STDs?" And depending on their answer, it might change my outlook on things. But, no. I would hope that they would tell me, but I wouldn't think of them differently if they didn't.

S1 80:55

[Okay?]. So then would it make a difference then to say a partner told you that they're on PrEP? Would it make a difference if it was a casual partner or someone in a long-term relationship?

S2 81:06

If it was a-- I feel like it would increase my standings with them. If I already knew that they were clean-- which, as I said before, those are really the only kind of people I would want to have sexual relations with. If I knew that they were clean, and they told me, "Hey, I'm taking PrEP, or a medication which helps defeat STD/AIDS," I would think of them, "Hey, this person obviously has an idea that it is a thing, it can happen, and they are taking steps to prevent it." It would put them in higher standing with me. I would feel a little bit more comfortable being around them, a little bit more open to having sexual relations with them because I know that not only are they clean, they are taking steps to make sure they stay clean. Yeah, that's how I feel about that.

S1 81:50

Okay. Good. All right. All right. So those are some of the factors that people have mentioned that are important [inaudible] PrEP. So I actually do have a small, one-sided fact sheet about PrEP itself. You're free to take this with you when the interview's over.

S2 82:09

I absolutely will.

S1 82:10

It's just information about the medicine itself: what it does, the frequency, the method of taking it, those kind of things. So I'd like you to just take a minute or two to read through that now.

S2 82:21

Yeah. Absolutely.

S1 82:22

Okay. If you have questions about what something means, just let me know, and I'll do my best to answer, but I'm not an expert on it.

S2 82:29

So that's fine. So let me ask you a question real quick. So are you just an advocate for PrEP? Do you actually work with people who are helping develop PrEP into a better drug? What's your involvement with this? Why are you making this survey?

S1 82:46

So me, personally, or the collective group?

S2 82:50

The collective is fine.

S1 82:51

So really, we're trying to see right now why are people choosing to use it or why are they not choosing to use it. What would potentially make it more appealing? What kind of information is out there about PrEP itself? So we're partnered with SUNY Upstate and several people who are very involved with trying to get people on PrEP. But so all of this, the qualitative interviews, what we're doing now, is going to lead into a more survey-based kind of thing to really try and figure out how to make PrEP more appealing for more people.

S2 83:26

Gotcha. Gotcha. [inaudible].

[silence]

S2 83:53

So I actually have seen this name, Truvada, once or twice on TV or whatever it was that was advertising it. So, yes. I have, under this name-- not under the PrEP name, but under the Truvada name, I have seen it more often, but I still don't really know much about it except for now, obviously as I'm reading this. [inaudible].

[silence]

S2 84:42

So the implant, would it be like a small like you put it into a syringe and have it implanted, or would you need to have a small incision made and have it [inaudible] in?

S1 84:56

That's more the option that they're looking at. It'll be something similar to how implants for birth control work [under arm?]. That's something that's still very much in development, so I really don't have a lot of information on. I can ask for more about it if you wanted it.

S2 85:12

No. I mean, considering the fact that it's still kind of in the shaky end of things-- not shaky, but it's still under development, I'm not incredibly worried about it. However, like I said, as it says here, you take it as a daily pill, which is, like I said earlier, what I would prefer in the [inaudible], or the Truvada rather. So the daily pill alternate is definitely what I would prefer anyway, and seeing that it has a 90% rate of effectiveness, there's no real crazy side effects besides the bone density thing which is-- again, my mom suffers from that, but that's because she's been on steroids for years. The fact that it's covered by insurance plans are great and also the fact that if you don't have insurance, there's programs that you can get it besides that. So, from what I'm reading here, this is a very viable drug for a lot of individuals who would like the extra prevention from HIV.

S1 86:25

So is there anything on there that kind of surprised you or maybe just you weren't expecting to see or--?

S2 86:33

Not so much that surprised me. Again, because I went into this not knowing much about PrEP and the PrEP program. However, again, seeing the percentage rate that it's effective is really good. I'm very happy with the 90%. The fact that insurance companies cover this is really good. So just a lot of good things going on.

S1 87:00

Do you think any of the information on that sheet changes how you think about PrEP now?

S2 87:05

Well, yeah actually, all of it does because, as I said, I went into this not knowing anything. So seeing this sheet and seeing all the positives definitely increased my thoughts on it. However, if I were to take this, I would still want to find other sources which don't come from someone who are advocating for PrEP. It would come from sources who may or may not have other experiences, other things like that, just so I had more of a not so one-sided idea of [inaudible]. So I'd want to have a full, broad knowledge of it before I actually started taking it.

S1 87:38

Oh, right. So to what extent do you think other people in the LGBT community know about that information there?

S2 87:47

I have a feeling that this information is well known for people who are very much into the LGBT community. Unlike myself, who is bisexual who doesn't have sexual relations a lot, who isn't into any certain group or fetish or whatever that has more sexual activity. People like me, who kind of, I'll say, skirt around the edges of the community, might not know about it as much. But I would hope that this information is more apparent with those who have relations like that more often.

S1 88:25

Sure. Do you think increased awareness of these facts or this kind of information would affect people's choice to take PrEP?

S2 88:33

Yes, it would. But again, these facts do come from a source which is advocating for it. I mean, you are advocating for it, right? Yeah. And it's great. I'm really happy that you are, and if these facts are actually correct, then yes, fantastic. However, if people were to take this, I would hope that they would see all sides of the story, figure everything out, then decide, based on their own health and their own experiences, how would they take it, how they would handle it.

S1 89:03

Sure. Okay. Great. So again, thank you for kind of going through all of this with me. I know we've been talking for a while, but we're almost done. So there's two short exercises left. One is a brief ranking exercise, there's another, and then the self-administered questionnaire that I'd mentioned earlier. So on these cards here - there's seven of them - are the factors that other people have said are important for thinking about taking PrEP. So it's the things I asked you, like what do you know about this? What have you heard about? So if you can't read my handwriting, let me know. But so look through these cards and tell me if there's anything that you would add, or that you might think is missing from here because I have other note cards that are blank and we can write on.

S2 89:54

When you say stigma, do you just mean how other people would see you taking this?

S1 89:58

Yep.

S2 90:03

[inaudible]. I think these cover pretty much all the bases that I would have personally with them. I don't have any other issues that would come up. Even if I was thinking from someone else's point of view, I have a feeling that these cover all the bases, and they are broad enough where, if you think of a situation or a scenario, one of these covers it. So yeah, no. Maybe discreetness might be one you might want to add. Again, taking a pill is relatively discreet, which kind of comes into the ease of access as well. But the fact that you're able to take it, see your doctor, have these things come to you relatively discreetly if you're closeted could have a large impact on some people.

S1 90:54

Definitely. Great. So discreetness there. All right. So now with the eight cards now, I want you to put them in order of most to least important for you.

S2 91:04

Left to right? Most important's on the left, least important--

S1 91:06

You can do it that way. You can do it just stacking them on top of each other. It's whatever the space really allows for.

S2 91:11

All right. So I'll do it left to right, least effective on the left, most effective on the right-- or not effective but most important. So least important would be probably stigma. How often do you have to take it really [nothing to do?] either. Side effects are pretty important. How you can take it is pretty important. Cost. Those are the three big ones. Ease of access. That's pretty important [with this economy?]. Discreetness, effectiveness, or discreetness [inaudible]. So it'd probably be ease of access, effectiveness, and [let's scoot this a little more over?]. Whatever. Probably discreetness after that. And then we'll go back down here, back to [inaudible]. It'd be side effects, cost, and how it's taken. So stigma would be the least important; the how often you have to take it would be second; ease of access, third; effectiveness, fourth; discreetness, fifth; side effects, sixth; cost, seventh; and then how you take it would be the most important.

S1 92:21

Okay. Can you tell me a little bit about about why how you can take it is the most important?

S2 92:27

This kind of falls into the discreetness as well, but it's also the fact that the more ways you can take a drug, in my opinion, the better. It gives people who have fear of needles, the inability to take a pill for whatever reason, if they are already under a plan where they need injections every week or whatever, this could fall into that plan. If they are taking certain drugs which shouldn't be mixed, each one of these options has a different usefulness. So if you can take it more ways, more people would be open to taking it. And that is closely matched with cost, where, obviously, if you're someone like me who's a college student or who has bills to pay or works maybe a single, full-time job and has a car, an apartment, this, that, whatever, the cost is also really important as well.

S1 93:20

All right. Why is stigma the least important?

S2 93:23

As I said before, there really shouldn't be a stigma for people who take this drug. It's important. It's something that if you're taking it, in my opinion, it shows off that you are thinking highly about yourself, about your body. It shows that you are at least thinking that this is a possibility. I'm taking steps to prevent it. It's almost like the stigma should be the fact that if you're taking it, you should be thought of in higher regards of [crosstalk]. Plus, especially in the community as it is right now, a lot of people are thinking it's my thoughts, it's my [train?] of ideas. Why should I let other people decide on that?

S1 94:05

Great. Okay. Great. So if we want to put these away. Thank you. All right. So then this other exercise that I have. It's a small table here so unfortunately, we can't actually do this. We don't have the funds. And so hypothetically or just imagine that I'm going to flip a coin and you're going to pick one of four rows. And so, depending on which row you pick, if the coin lands on heads, you get that value, and if the coin lands on tails, then you get that value. But you can only pick one of the four rows.

S2 94:47

Gotcha.

S1 94:47

Does that make sense?

S2 94:48

Yep, totally understand. So you would flip the coin. Do I choose before or after you flip?

S1 94:54

So you're going to pick before.

S2 94:55

Before. [Oh, I see?].

S1 94:56

And then the coin will be flipped.

S2 94:57

Why would I choose after [laughter]? Why would you ask that? It's dumb. Okay, so if were to pick before, as much as row one would be the best alternative, you're guaranteed to get something, a 50/50 chance, I personally like to take a little bit of risk, and I like to-- especially when it comes to gambling [or tight?] situations. So me personally, I would like that either row two or three, more on the end of row two, especially since there's no buy-in value or anything like that. I'm not giving up any money to get this. I would probably go with actually row three.

S1 95:35

Row three?

S2 95:36

Yeah, definitely row three because either way I'm getting more money than I started with. However, if on the 50% chance it does land on heads, I get a large sum of money, a nice quantity of money. If it lands on tails, again, 10 bucks will pay for lunch or something, and that's all based on the flip of a coin, so that's fine. So I would probably choose row three.

S1 95:59

Okay. Great. All right. Great. So then the last thing that I have for you to do, this is a short questionnaire. This is really kind of just demographic information so we can help make the second part of our study.

S2 96:11

Okay. Should I write my name on this at all or just leave it?

S1 96:12

No, no, because this is not connected to anything that you've said throughout the interview. It's not connected to you at all. If there's any questions you don't want to answer just skip through them. There's a couple where the formatting is just weird so the question will start at the bottom of a page--

S2 96:29

Oh, gotcha. It ends on the--

S1 96:30

--and it ends on the next page.

[silence]

S1 97:55

I'm sorry. [She had?] this reserved.

S2 97:57

Maybe. She might.

S3 97:59

I'm sorry. Just so you know, I have reserved this room for an interview in 15 minutes.

S2 98:04

Yep. Well, I'm almost done with this and we'll be right out.

S1 98:06

We'll be like five more minutes.

S3 98:07

Okay. Thank you. Sure.

S1 98:09

Thank you.

[silence]

S2 98:28

Nope. [inaudible] in the last six months. [inaudible] will make you feel drunk. Maybe my [inaudible]. No and no. [inaudible] risk of infection. [inaudible].

S1 98:46

All right.

S2 98:48

There you go.

S1 98:48

All set?

S2 98:49

I'm all set.

S1 98:49

Great. So just a couple little things to kind of wrap up the interview. I have another sheet for you to take. So this is a list of PrEP providers here in [Upstate NY City 2], different services or health centers, that are available if you wanted to find out more on them. But it seems like you might be more inclined to look online to see other sides as well.

S2 99:12

Yeah, I'm open to all sides. If I do decide, "Hey, I want to take this," then I'll learn more about how I can get involved, I will definitely contact one of these people first.

S1 99:21

Okay. Great. And then at the bottom here, there are counseling services available. So one of the risks of doing these kinds of interviews is potentially bringing up traumatic memories or negative experiences. So if you need these services, they are here for you. For any of these, if you want help scheduling an appointment or getting in, you can reach out to us and we'll do everything to help you as well.

S2 99:45

Okay. Yeah, no problem.

S1 99:46

Whether it's for counseling or for seeing someone about PrEP or being tested.

S2 99:49

Okay. Yeah, no problem.

S1 99:51

All right. And then as a brief reminder, you do have my contact information. You have the subject advocates at the back of your consent form.

S2 100:01

Which I have right here.

S1 100:02

Yeah, so Nuala and Catherine. So if you want to talk to someone who's not involved with the project, those are going to be who you reach out to. So and the last thing is just to get you paid. So this is for budgetary reasons so we can keep track of where our money's going. Did you need travel compensation?

S2 100:21

No, I was on campus already.

S1 100:23

All right. So it's just the 40. So if you want to put the date here, print, and then sign your name.

S2 100:29

It was the 6th, I believe. Is that right?

S1 100:31

Yes.

S2 100:31

Okay. So--

S1 100:32

So that's just the acknowledgment that you have been compensated for your time.

[silence]

S2 100:46

There's that. Thank you.

S1 100:49

All right. So did you have any other questions, concerns, or comments before--?

S2 100:55

No, not at all. Obviously, I wish you the best of luck with your research. And everyone listening to this tape right now, I hope all you guys, you get the information you need and you're able to promote obviously this medicine, which seems to be a very good thing to promote, if all the facts are correct here, which I assume they are. So, yeah, I hope you guys do what you need to do and get things done and those who ask me, this was a very nice experience. So, yeah. Thanks much.

S1 101:27

All right. So I'm going to go ahead and turn this off then.

Interview IDM 111

S1 01:00:21.298 Okay. So then what are some ways that you could prevent the spread of HIV?

S2 01:00:25.674 Definitely condoms. I would say [oral?] dams. Not brushing your teeth and creating cuts in your mouth before performing oral sex. Yeah.

S1 01:00:44.410 So say you did become infected with HIV or you acquired the virus. What are some of the treatment options that are available if you know of any?

S2 01:00:54.677 If you feel like you've been infected within I don't know, say a couple hours, I think you can take a pill. PrEP I'm aware. Yeah. And they put you on a regimen of pills for a month.

S1 01:01:09.903 Let's say maybe you didn't realize that or you didn't take it in time. So say you actually have HIV--

S2 01:01:16.469 You got it [crosstalk].

S1 01:01:18.118 What do you know of any treatment options that are available?

S2 01:01:20.858 There's an alternate I think of PrEP that you can take.

S1 01:01:30.602 Okay. So have you seen anything about HIV or AIDS kind of online? Have you looked into it online at all?

S2 01:01:44.310 I may have asked Google a couple of questions before.

S1 01:01:48.810 Okay. I'm just trying to get a sense of how have you found out about HIV/AIDS because I know you mentioned a human sexuality class, a little bit of information with doctors when you were getting tested, but I'm trying to see if there's other places that information might have come from.

S2 01:02:04.497 No.

S1 01:02:07.504 Have you seen anything in the media? So books, news, movies, magazines, anything like that about HIV or AIDS or people who are living with it?

S2 01:02:19.814 I love the movie Rent, so.

S1 01:02:22.464 Sure [laughter]. That's one of the key parts of the movie.

S2 01:02:26.801 Yeah. So I definitely think maybe that counts as media. I do know that I think-- I just learned recently, [inaudible] HIV Prevention Month or HIV Awareness Month, [which is purple?], I think. So I recently just learned about that, kind of through social media.

S1 01:02:48.515 Are there any more contemporary films about-- or TV shows? Because Rent, I want to say is early 2000s. It's also set in the '80s or early '90s.

S2 01:03:04.819 Yeah. No. [I guess not?].

S1 01:03:06.658 All right. So for you personally, how regularly do you use condoms when you have penetrative sex?

S2 01:03:21.058 [It really?] depends.

S1 01:03:25.574 So what does it depend on?

S2 01:03:28.735 Depends on how well I think I know the person. But I'm going to say out of all the times that I've had sex, I'd say I might have used condoms in a handful of times.

S1 01:03:41.050 A handful of times?

S2 01:03:41.919 Yeah.

S1 01:03:42.815 So the majority were not with condoms, then?

S2 01:03:45.775 [This is true?].

S1 01:03:48.585 So other than maybe just knowing the person well, what other kinds of circumstances have led to not using condoms?

S2 01:03:55.071 Spur of the moment. Location. Yeah.

S1 01:04:06.270 So when you say location, what's special about a certain location that would not warrant the use of condoms?

S2 01:04:14.781 Whether or not they're on hand. Yeah.

S1 01:04:20.560 Do you use condoms for-- it's an awkward question because you've said you've only used them a handful of times. But have you used them for types of sex other than penetrative sex?

S2 01:04:31.063 No.

S1 01:04:31.999 Okay. Is there a reason why not?

S2 01:04:36.032 No [laughter]. [I actually never have?] used them. This would be referring to oral dams, finger condoms--

S1 01:04:43.666 Or using condoms for oral sex, as well.

S2 01:04:46.140 Yeah. I'm going to say no.

S1 01:04:47.095 Okay. Have you ever wanted to use a condom but have a partner say no?

S2 01:04:53.306 No.

S1 01:04:55.826 So in those situations where you did use a condom, can you tell me a little bit about why you used a condom, if for the majority of times you haven't?

S2 01:05:08.543 If it was with a completely random person, then I felt the need to use a condom.

S1 01:05:20.313 Was that the only?

S2 01:05:22.491 I'd say that's like-- yeah, that's probably the only time I ever use a condom.

S1 01:05:27.702 Okay. Have you ever been worried that you might be infected with HIV?

S2 01:05:32.369 Yes.

S1 01:05:34.016 Can you tell me a little bit more about like what happened or what made you feel worried about it?

S2 01:05:40.298 Because it was a complete stranger. In the end, I definitely did not catch chlamydia but I was worried that I did catch chlamydia and so I feel like the person [might be?]-- and when I went to the doctor, she kind of scared me a little bit into thinking that I could've been infected. Yeah, I was kind of--

S1 01:06:07.769 So how did she scare you into thinking that?

S2 01:06:11.331 I don't know. She asked a series of questions. Yeah. I don't know, her whole tone. [inaudible] she was kind of like, "Okay. [inaudible] this could be something else, so."

S1 01:06:29.051 Got you. So what made you worry that you might have chlaymdia?

S2 01:06:34.120 Because I had like side effects-- or not side effects, I had symptoms.

S1 01:06:37.891 Like what?

S2 01:06:38.985 Like burning pain when I pee. Pus in your urine. [inaudible].

S1 01:06:49.690 [inaudible] don't know being chlamydia or--

S2 01:06:53.310 Okay. It ended up being like I tested negative but I still had the symptoms, so it was definitely something that could be chlamydia adjacent.

S1 01:07:03.488 Were you prescribe anything or--?

S2 01:07:05.447 Yes. She gave me some medicine. Well, back to your case. I ended up taking PEP for a month.

S1 01:07:15.967 Okay. So you have taken PEP [though?]?

S2 01:07:17.961 Yeah.

S1 01:07:19.177 Can you tell me what your experience was like taking that or--?

S2 01:07:21.954 This is going to be really bad. I didn't take it the whole prescribed time. It's like she gave me pills for chlamydia and she told me that I'm supposed to take this for a month but I didn't end up taking it for a month but--

S1 01:07:47.790 Was there a reason why you didn't take it the full month or--?

S2 01:07:51.793 I think, in the end, she made it seem like it was just like a safety precaution. It's like I'm not supposed to take it for the whole time and just to be safe. But I think, in the end, I felt like I didn't think that I had [to?].

S1 01:08:10.057 Sure. So how long ago was that?

S2 01:08:14.800 Two years.

S1 01:08:17.242 Yeah. Did you talk to that guy about this?

S2 01:08:22.661 I did. Well, I mentioned it. I was like, "Hey. BTWs, you might want to get tested because-- yeah."

S1 01:08:32.579 Did he have a response?

S2 01:08:34.413 He just said, "Thanks."

S1 01:08:38.480 Did you two continue to talk [after that?]?

S2 01:08:40.056 Yeah.

S1 01:08:43.039 So thinking in the future, how worried are you about contracting HIV?

S2 01:08:53.717 Well, I have now recently decided that I wanted to try to get on PrEP, so. And I've also limited my number of sexual partners [a lot?], so.

S1 01:09:10.474 [inaudible] but if you had to base it on kind of a numerical scale. So from 0 to 100 - 0 being the lowest chance, never going to happen; 100 being absolute certainty - what would you say your risk of getting HIV in the future is?

S2 01:09:28.892 Wait. That was 100% certain that I would get it?

S1 01:09:30.814 Yes. So 0 is never going to happen. 100 is definitely will happen.

S2 01:09:38.016 I'd say there's like a 2% chance.

S1 01:09:41.280 A two?

S2 01:09:41.920 Yeah.

S1 01:09:42.819 So why that number?

S2 01:09:46.679 Well, because I have not contracted it yet, one. I'm not as sexually active now, I'd say. I mean, sure, there's a [still?] chance, but I'd like to think that there's a [better?] chance mostly.

S1 01:10:17.629 All right. Do you think that number would potentially-- it's actually fairly low already. But decrease--

S2 01:10:23.939 [crosstalk] [laughter].

S1 01:10:25.189 --if you were to, say, use condoms for every sexual encounter, or--?

S2 01:10:29.554 Certainly.

S1 01:10:31.823 Great. So then moving to kind of like one of the last areas of the questions for the interview, is about PrEP. It's kind of trying to see how much you know about it. Right. So to start, you said you did have a friend, a previous sexual partner as well, who's on PrEP.

S2 01:10:55.094 Yeah.

S1 01:10:56.986 So what have you heard from him about it?

S2 01:11:00.459 He's a bad example [laughter], but--

S1 01:11:04.579 That's perfectly fine.

S2 01:11:06.731 I mean, he takes it once a day. He says you don't have to wear-- or you don't have to worry about wearing condoms every single time. Yeah. And that's what the gist of what he says.

S1 01:11:21.628 Okay. Do you know anything else about PrEP other than what he has told you or--?

S2 01:11:29.883 Well, besides what the doctors have told me every time I've gotten tested. I knew that it doesn't protect against STDs. There's like if you don't take it every day then you still become susceptible to catching it and stuff.

S1 01:12:02.884 So you said doctors have talked to you about it--

S2 01:12:04.484 Yeah.

S1 01:12:05.231 --when you have gone tested before. So what kind of things have they shared with you about PrEP?

S2 01:12:12.046 That it's a very good protection against catching HIV, especially for someone that's in a high-risk group which I have been told that I'm in a high-risk group. Yeah. Okay, some of them have mentioned about access to getting PrEP. So [inaudible] if your insurance doesn't cover it then someone will do like sponsor or pay for it which is also something my friend has told me.

S1 01:12:48.500 Do you know if his insurance covered the medicine or--?

S2 01:12:51.496 I think his insurance does.

S1 01:12:54.194 So have you found out about PrEP from other people or have you looked into it online at all?

S2 01:13:01.609 I've Googled it before. I can't tell you anything about everything [inaudible] but I've definitely looked it up to find out what it is. I think I actually looked at it when it first came out. I didn't know anything about it. I think I might have heard about it from TV or something and I looked it up.

S1 01:13:21.397 Has it come up in a conversation at all with friends or--?

S2 01:13:27.345 Come up as in I've talked with my friends that I've considered it? Yep.

S1 01:13:30.995 What kind of reactions have they had?

S2 01:13:34.135 My friends don't know anything about it, so, yeah.

S1 01:13:40.635 Have you tried to tell them about it or--?

S2 01:13:43.433 I just went to my girlfriend's-- but it's like birth control for gay men. That's my simplest way to explain it.

S1 01:13:52.496 So you said maybe you've seen it on TV?

S2 01:13:56.297 Oh, yeah.

S1 01:13:57.213 So in what kind of context, like a TV show, or a commercial, or--

S2 01:13:59.716 [inaudible].

S1 01:14:01.810 --was there a particular show?

S2 01:14:03.702 Looking on HBO.

S1 01:14:06.142 Can you say that again?

S2 01:14:07.314 I'm sorry, looking on HBO.

S1 01:14:09.369 Looking on HBO.

S2 01:14:10.356 Yeah.

S1 01:14:11.037 Can you talk about--

S2 01:14:11.148 [inaudible] about it.

S1 01:14:13.092 So how does PrEP factor into that show or come up in there?

S2 01:14:16.884 I don't think anybody on the show is like, "Oh, man". But I think they may have mentioned before. Some of the [inaudible] characters might have been [HIV?]. And also one of the characters I think has HIV so he was like-- I think he mentions it to his partner. I'm pretty sure. I'm spoiling this.

S1 01:14:36.638 That's fine [laughter]. I don't have HBO.

S2 01:14:39.923 Oh, really?

S1 01:14:40.270 So that makes it a little bit harder to watch. So what has stopped you from finding out more about PrEP or deciding to take it before now?

S2 01:14:57.739 Procrastination. Yeah.

S1 01:15:04.935 Well, that's fair.

S2 01:15:06.037 I haven't felt the need because I-- yeah, I haven't felt the need that I needed it.

S1 01:15:15.430 Especially if you're limiting the number of sexual partners.

S2 01:15:20.117 Yeah.

S1 01:15:22.067 All right, okay.

S2 01:15:22.977 We'll go with that [laughter].

S1 01:15:24.518 Well, is there something else you would go with?

S2 01:15:30.005 No, I think that-- yeah, limiting sexual partners, yeah.

S1 01:15:33.378 Okay. I guess I'm trying to see too, what are some of the other reasons you haven't felt the need to use PrEP before?

S2 01:15:46.692 Yeah, definitely procrastination. Because I feel like I've talked like I would use it. "Oh yeah, I'll definitely get on it." And then I never did. And time passed. And I also was not sexually active, so didn't feel the need to get it.

S1 01:16:10.635 Sure. And then, your friend and sexual partner at that time, is he still - excuse me - is he still taking PrEP now, or--?

S2 01:16:21.875 As far as I'm aware.

S1 01:16:23.059 Okay. Have you talked about PrEP with any other sexual partners or--?

S2 01:16:30.470 Not really, no.

S1 01:16:31.995 Have you seen it come up kind of on different Grindr profiles or anything?

S2 01:16:35.827 Definite.

S1 01:16:36.775 Does that affect your decision to either talk to people or--?

S2 01:16:40.511 No, not necessarily.

S1 01:16:41.478 Okay. So I'd like to ask you about some things that other people have told us are important when they think about taking PrEP. So there's no right or wrong answer to these questions either. And it's totally fine if you don't actually have anything to say in response. So what do you know about the cost of PrEP, if anything?

S2 01:17:08.569 I don't know.

S1 01:17:15.732 You said though you think your friend's insurance covers it?

S2 01:17:19.737 Yeah.

S1 01:17:20.627 You mentioned something about programs that might cover it if your insurance doesn't. Do you know anything more about them, or--?

S2 01:17:30.892 No.

S1 01:17:31.742 Okay. It's totally fine. Would you feel comfortable using your own insurance to pay for it?

S2 01:17:40.330 I would.

S1 01:17:41.487 Are you on school's insurance or--?

S2 01:17:43.352 I have no insurance.

S1 01:17:44.397 Okay, okay. So where would you go if you wanted to access PrEP?

S2 01:17:53.660 To my care physician.

S1 01:17:56.981 Do you know if there's other places that you might go, or--?

S2 01:18:00.882 So, okay, I recently talked to this lady who's-- I feel like she targeted me, it was really weird. Not a bad weird but I was at a bar and she came up to me and she started having a conversation. Her friends were [inaudible] with my friends and she came up to me and asked. And eventually it led to a conversation about PrEP. And she's a PrEP specialist who works for Upstate-- yeah, who works for Upstate. And she basically said, "If your insurance doesn't cover it, they'll figure out something to pay for it or something." I don't know if they have ways of trying to get people on it, or--

S1 01:18:48.968 So it was just a woman who came up to you at a bar and started--

S2 01:18:52.295 It sounds like it, but it happened so suddenly that it-- it didn't start that way but it ended up that way. So, yeah.

S1 01:19:01.158 And how recently was this?

S2 01:19:03.794 That was a week ago, maybe two weeks ago. A week ago.

S1 01:19:10.583 Do you--

S2 01:19:11.546 It just happened to coincide with me finding the study and I was like, "Oh [laughter]." Kind of gave me [inaudible].

S1 01:19:17.900 Do you happen to remember some of the other things she might have mentioned about PrEP?

S2 01:19:21.112 No. She didn't really give details of-- because I think I kind of said, "Oh, I kind of know. I know what it is."

S1 01:19:32.835 Did she say her name by any chance?

S2 01:19:34.192 Her name?

S1 01:19:34.813 Yeah.

S2 01:19:35.542 You want her name? I thought it might have been [crosstalk].

S1 01:19:37.389 [inaudible].

S2 01:19:38.906 Do you know her?

S1 01:19:40.512 I'm wondering if it might be someone I know.

S2 01:19:42.447 If you say her name, I'll know.

S1 01:19:43.864 No, it's fine. It's just curious that someone who works from Upstate is talking in a bar about PrEP [laughter]. So anyway, going back to where you go to access PrEP, you mentioned your primary care physician.

S2 01:20:01.720 Yep.

S1 01:20:02.907 Do you know if there's anywhere on campus or around campus that you could go to?

S2 01:20:06.667 No. I mean, I'm pretty sure the LGBTQ Center might give you information about it. I've never been there, but, sure.

S1 01:20:18.730 So what if anything have you heard about what you have to do to take PrEP ? Meaning, do you know how often you have to take it? Do you know what kind of form it is? Is it a liquid, a pill, an implant, injection kind of thing? How often do you go to the doctor's when you're on it?

S2 01:20:40.205 So I know that it's a pill. I know that you take it daily. I know that you have to get tested, or generally you're supposed to get tested every three months just in general, I think. But, so yeah. That time period.

S1 01:21:00.205 Do you feel like you'd be able to take PrEP on a daily regimen?

S2 01:21:05.468 I've always questioned that, and I mean, yeah. I'll say yes.

S1 01:21:12.318 I guess I know some people tend to have difficulty swallowing pills or kind of an aversion to it.

S2 01:21:18.748 No, [inaudible].

S1 01:21:21.197 Would you feel more comfortable or more likely to take PrEP if say it were an injection rather than a daily pill?

S2 01:21:27.916 No.

S1 01:21:28.751 Okay. What about if it were say an implant under your skin?

S2 01:21:33.004 No.

S1 01:21:33.764 No? So the pill would kind of be the best option then?

S2 01:21:39.053 From what I know about those three things, yes [laughter].

S1 01:21:43.202 What if anything have you heard about the effectiveness of PrEP?

S2 01:21:49.850 I have not heard of any, I don't know. I haven't heard of anything.

S1 01:21:58.720 About it just generally being effective or--

S2 01:22:01.140 Well, I heard it's pretty effective, but I haven't heard cases where it has not been effective.

S1 01:22:08.562 Sure. How effective would PrEP have to be for you to consider taking it?

S2 01:22:16.995 Well, I know nothing is 100% effective [laughter] as [any?] condoms, but 99.9.

S1 01:22:24.871 So you would want PrEP to be 99% effective for you to take it?

S2 01:22:28.515 I would like for it to be 100% effective, yeah.

S1 01:22:31.189 Would you be willing to take it, say if it only cut your risk of contracting HIV in half? Meaning say that it cut that risk down by 50%, would you still consider taking this daily pill?

S2 01:22:47.231 Potentially. I'd consider it.

S1 01:22:50.942 Have you heard anything about the side effects from being on PrEP?

S2 01:22:55.708 I have not.

S1 01:22:56.865 Okay. And then, do you think friends or potential partners might look favorably or badly on people who take PrEP?

S2 01:23:08.703 Yes.

S1 01:23:09.647 Okay. Can you tell me a little bit more about that?

S2 01:23:12.185 I told somebody that I was considering it, and the-- it was a friendly reaction, but it was still a pretty shitty reaction. He called me a slut, which I thought was interesting, so. Can I say this? I'm going to say there are people, I think, who have a negative reaction of people that take-- which I've also heard of that [inaudible] comment. I've heard of people saying that people that take PrEP are very slutty, and yeah.

S1 01:23:43.307 So are there other kind of negative associations, or is it just really kind of the sluttiness?

S2 01:23:49.327 Yeah, I think that's-- as far as I know.

S1 01:23:51.758 Okay. If you were taking PrEP, would you tell your sexual partners about it?

S2 01:23:59.659 I think so. Yeah.

S1 01:24:01.582 And if they were taking PrEP, would you expect them to tell you about it?

S2 01:24:07.129 Yes. I guess [I was just?] [inaudible] too, but I don't know. Yeah [laughter].

S1 01:24:11.090 So can you kind of explore a little bit, why would you expect them to tell you?

S2 01:24:16.725 I think it would be good to know. I think it could also potentially give insight into maybe-- or open up the conversation about what their sexual activity's like, or if they've had sex with people that have been HIV positive. Things of that nature.

S1 01:24:39.381 So how would you feel if a partner disclosed that they were taking PrEP? Either before you've hooked with them, or maybe after once or twice, and they come out and just tell you, "Oh, by the way, I am taking PrEP." How would you feel about that?

S2 01:25:01.384 I think if it happened after, I'd be like-- I mean, I guess that'd be a good thing to know. I guess it's good [they were?] [inaudible] before.

S1 01:25:13.717 Do you think there's ever a situation where you might react negatively from being told that, or it might not be a good thing that you've heard this?

S2 01:25:26.899 I'd say no.

S1 01:25:28.384 Okay. [That's all right?]. So I have here, somewhere in this pile, a fact sheet about PrEP, just containing some information about those different things that I just asked you about, like cost, where you can get it, ease of access kind of thing. So you're free to take that with you when the interview is over. But I'd like you to just kind of read through it briefly. If you have questions about what something means, let me know. I'll do my best to answer.

S2 01:26:03.487 I do have questions.

S1 01:26:04.456 Sure.

S2 01:26:05.027 So you asked about the effectiveness of PrEP. What do you think about the effectiveness [laughter]? Have you heard of situations where it's not effective?

S1 01:26:16.858 I'm happy to talk to you about that when we get towards the end of the interview.

S2 01:26:21.565 Okay.

S1 01:26:22.656 But for the time we have right now I'd love to focus on what you know about PrEP.

S2 01:26:26.724 Okay. That's okay.

S1 01:26:27.933 I do think as you read through the fact sheet you might find an answer to your question as well. You can feel free to ask me any questions towards the end.

S2 01:26:38.632 Wait, so this is related?

S1 01:26:39.738 No, read through that now.

S2 01:26:40.892 Oh.

S1 01:26:41.556 Yeah. So I'd like to take a minute or two to just kind of read through that.

[silence]

S2 01:28:43.099 Okay.

S1 01:28:44.022 Great. So just the one side. So having read through that, was there anything that surprised you or that you didn't know before?

S2 01:28:54.537 I didn't know about the kidney problem. I definitely didn't know about the newer forms of PrEP that are being studied or researched. I didn't know but I guess I did kind of know, but the people that use condoms. And I guess if the condom, if they've been exposed, then they take PEP rather than taking PrEP every day. It's kind of like, "Oh, I'll take it if I need to take it," which I thought that was kind of weird, but okay. 90% or more was also kind of a-- I'd never heard that number before, so that was a first time [for that?]. And I also didn't know about the [inaudible].

S1 01:29:49.633 Right. So the effectiveness of, or cases where it hasn't been effective. Is that an answer to the question, or--?

S2 01:29:55.584 Yeah.

S1 01:29:56.303 Okay. So you pointed out, in particular, the kidney problems. Is that something that is alarming to you or kind of really stood out?

S2 01:30:05.273 I mean, 1 in 3,000 is not a huge number, so yeah.

S1 01:30:12.373 Okay. I was just trying to see--

S2 01:30:13.495 Interesting, surprisingly.

S1 01:30:14.730 Great. All right. So I know you mentioned that you haven't necessarily been to the LGBT Center on campus here, but do you think the people that work in that space or go to that space would know about some of this information?

S2 01:30:31.991 Potentially.

S1 01:30:32.839 Okay. What about the LGBT community in general? Do you feel or do you think people know about this kind of stuff or about [crosstalk]?

S2 01:30:43.773 No. Not necessarily [crosstalk], no. Not to this extent.

S1 01:30:46.524 Okay. Do you think if people were more aware of these facts or the numbers-- do you think that kind of awareness would affect people's choices to obtain PrEP?

S2 01:30:59.444 Maybe, maybe not. I don't know. [inaudible]

S1 01:31:06.565 So why would it maybe not affect their choice to use PrEP?

S2 01:31:12.644 Well, I still think maybe people still think that condoms are the best way to go, and if the condom breaks or if you decide not to use a condom, maybe they feel that the person they're having sex with does not have HIV or they're not worried that the person has HIV. Or maybe they don't think about it, or they could be lied to. So [inaudible].

S1 01:31:39.619 All right. So there's a couple of other small things that we need to do. And I know we've been talking for awhile, but I promise we're close to being done.

S2 01:31:50.216 Do I keep this?

S1 01:31:50.989 Yeah. You can hang onto that. That's yours. So there's two exercises, and they're a little different than just kind of talking like we've been doing. So I have written on these index cards the factors that I talked about when people said, "Oh, this is important, for me, when I think about PrEP." So I want you to take a look at those. If you can't read the writing, let me know and-- anyway. So looking through those - there should be about, I think, seven in the stack - I want you to tell me if you think there's anything missing. If you do, I have more index cards that we can write on.

S2 01:32:33.357 Okay.

[silence]

S2 01:32:51.596 Honestly, no. Nothing that I would add.

S1 01:32:53.781 Okay. So if you could take the cards and kind of rank them in order of most important to least important.

S2 01:33:02.330 Most important to--

S1 01:33:02.782 For you. Yeah. So you could go across the table this way, or you could go vertically here.

S2 01:33:08.807 Okay.

[silence]

S2 01:33:38.682 Yeah. I think that's [done?].

S1 01:33:39.947 So in order of most important to least important.

S2 01:33:42.953 Most important to least important, yeah.

S1 01:33:44.393 So we have effectiveness at the top, followed by ease of access, then cost, then side effects, then stigma, then how often you have to take it, and finally how you can take it. So is there a reason why the how you can take it is kind of at the bottom or is--?

S2 01:34:03.293 Because I've never thought about this before, yeah.

S1 01:34:05.947 Okay. And then how about why effectiveness is the most important?

S2 01:34:14.665 Well, I feel like that's the whole purpose of it. [Or at least?] I think, yeah.

S1 01:34:19.281 All right. So is there a reason why stigma is kind of down towards the bottom, as well, or--?

S2 01:34:25.589 It's more of a personal thing. I don't really care [laughter] about stigma. So it's not like-- I mean, I understand the stigma, but maybe it doesn't really affect me as much. Just [personal?], yeah.

S1 01:34:41.420 All right. And so you don't think there's anything missing from this, or something that would be important for thinking about using or not using PrEP?

S2 01:34:55.879 I'd say no.

S1 01:34:55.914 Okay. [That's fine?]. I just wanted to make sure. All right. Okay. So you can slide them back together. [I've got another?] small exercise here. I'll trade you. So on this sheet of paper there's four rows. And so I want you to imagine that I'm going to flip a coin. And depending on how the coin lands you would get what's in the heads column or the tails column, but you can only pick one row.

S2 01:35:32.502 Say that one more time.

S1 01:35:33.322 Right. So you're going to pick one of those four rows.

S2 01:35:36.445 Okay. Sure.

S1 01:35:36.983 One, two, three, four, based on how a coin would land. So you pick row one. If the coin lands on heads, you get $50. If it lands on tails, you get $50. Row two, if the coin's on heads then you get 100, but if it's on tails you'll get $30. So for you, if we were actually going to do this [laughter] - unfortunately, we don't have the funds to do this - which row from this table would you pick?

S2 01:36:12.966 And I can only pick one row?

S1 01:36:14.118 Yep, only one.

S2 01:36:20.989 I guess I'd go row one.

S1 01:36:31.813 Row one? Okay.

S2 01:36:32.224 Yeah.

S1 01:36:32.828 So you'd go for the one that 50/50?

S2 01:36:35.591 Yeah.

S1 01:36:36.443 Can you explain your decision [about that?]?

S2 01:36:38.323 I would love to go for one of the higher ones, but it felt like the opposite of the higher ones is not as high. So I guess, yeah, a medium kind of, just to walk away with something.

S1 01:36:51.349 Okay. But row two is--?

S2 01:36:54.411 Row two would be my second choice, yeah. Because $30 [inaudible].

S1 01:37:00.406 Okay. Great. All right. It's a really short little exercise. And the last thing that I'd like for you to do is just a small, self-administered questionnaire that I'd like you to fill out. So, a pen. Your name, no identifying information is going to be attached to this. It's just more for the demographic part of the research. So if there's anything on there that you're not comfortable answering or you don't want to answer, just feel free to skip over it. It should maybe take about five minutes to fill out. Sorry, before you get started. So like here, number 23, the question is here and then the responses are here. It's just the way the page got formatted. I think there might be one or two other questions like that. So if you see a question with no boxes, or spaces, or anything, it's probably on the next page.

S2 01:37:58.814 Okay.

S1 01:38:00.604 If there's anything you have any questions about, feel free to ask.

[silence]

S2 01:41:26.587 Okay. Yeah.

S1 01:41:28.339 All right. All set. Okay. We're just about there. So another sheet for you. This is a list of PrEP providers in [Upstate NY City 1]. It's, unfortunately, a smaller list. And then, so down here, and then onto the backside, are counseling services because one of the risks of participating in these kind of in-depth interviews is talking about negative experiences or bringing up emotions. So if you want to talk to someone about this, these are services available. If you want help making an appointment somewhere, please feel free to reach out to us and we will do everything to help you with that. But that's yours to take with you. So then the last thing is to just get you paid for participating. Today is October 2nd.

S2 01:42:32.256 Is this-- sir?

S1 01:42:33.421 Sure.

S2 01:42:34.092 Do you know these places on this list?

S1 01:42:35.640 I don't know them personally, no. I'm sorry.

S2 01:42:39.116 That's okay.

S1 01:42:40.617 Is there one that you were actually interested in, or--?

S2 01:42:43.776 [inaudible].

S1 01:42:45.522 Asking about, or--? Okay.

S2 01:42:47.268 [inaudible].

S1 01:42:49.641 I'm sorry. I haven't used the services before so I can't speak for them personally. So this is another kind of formality to sign that you acknowledge that you participated in our research study and you've been compensated for your time. So it's $40 for the interview. And did you have any kind of parking or travel expenses?

S2 01:43:11.802 No.

S1 01:43:12.074 Okay. So if you want to just print your name here and then sign. This is really just kind of for budgetary concerns. So again, I just want to say thank you for talking with me, for sharing some of your history, your experiences. I know it's not always easy to sit down with a stranger and get into some of this. So before you head out, did you have any other questions, concerns, anything you wanted to talk about?

S2 01:43:52.222 [Not personally?]. I don't know if you can talk about it, but what exactly are you guys going with the research in the study?

S1 01:44:00.179 So where are we moving forward with this?

S2 01:44:01.868 Yeah. Where are you trying to go with it?

S1 01:44:03.635 So the next step of the study is making a quantitative research project. So it's something that we could market-- or not market, have a lot more people do. So right now, we're doing these kind of in-depth interviews, just trying to understand some of the circumstances about using PrEP, what's important for it, what knowledge exists. And then once we've had those interviews transcribed, we'll take that information and kind of code it in a way that lets us see these recurring patterns or themes; that way we can tailor more of the survey kind of stuff to that. But so the next step is we're creating a discrete choice experiment, which basically you'll be given a few options. Option A might be PrEP is a daily pill but it costs $100 and you have to see a doctor every three months. Option B is PrEP as an implant under your arm, but it costs $300, but you see a doctor once a year for it. So what would you choose there? So it gets a little bit more into the clinical side of it. At this stage, we really want to try and get more of the lived experience around it, rather than just kind of getting numbers about it. I'm not entirely sure where it heads after we've done those discrete choice experiments, mostly because it took us over a year just to be able to do the project at all. And so this took longer than we expected. This is taking a little bit longer than we expected. So everything's just kind of been put on the backburner for now, so I'm not totally sure. That's something that, on the consent form that you have--Elizabeth, James, Andrew, or Amy would be able to answer much more easily and readily if you wanted to ask more about it.

S2 01:46:03.213 Okay.

S1 01:46:03.557 Anything else?

S2 01:46:07.343 That's it.

S1 01:46:07.973 Okay. Great.

S2 01:46:09.451 [inaudible]. Sorry, last--

S1 01:46:10.580 Sure.

S2 01:46:12.176 You're only doing men? [crosstalk].

S1 01:46:13.365 Yes. So the trans women, but part of our study criteria is that your sex at birth was male.

S2 01:46:22.504 Okay. Yeah. Okay. Yeah.

S1 01:46:26.110 Great. Thank you.

S2 01:46:26.836 No problem.

Interview IDT 004

S1 00:44:26.636 Okay. So you know about-- just for the record can you state about transmission, how it's transmitted, prevention, treatment.

S2 00:44:35.378 So, more so HIV, exposures happen through things like protein based-- not saliva, not urine. So things like blood, breast milk, yeah. You can be detectable, undetectable. There's window periods. There's how much you-- there's window periods and then there's being undetectable and not detectable. The treatment is the tricky part because it was over 115 forms of medicine, but-- yeah. That's really what I-- you know what I mean? Put a condom on, use PrEP, yeah. You don't know what your partner has. So that's what I got [laughter].

S1 00:45:22.516 Yeah. That's a lot more than a lot of people know, for sure. So how have you found out-- or when did you find out about HIV and how do you [crosstalk]?

S2 00:45:33.196 I found out about HIV probably a week before my cousin passed from it, and--

S1 00:45:41.529 When was this?

S2 00:45:42.519 This was probably 2007, '06, or something like that. And I didn't know she was my cousin. We had school or whatever, [it was?] sex education, but it wasn't that serious. And then a week later my dad was crying. I'm like, "Why's he crying?" I've never seen my dad cry. He's like, "Cousin just passed," and next thing you know, we're at a funeral [of hers?]. And I'm just thinking nothing of it, but everybody has this tone, this weird underlying, "How'd she die? How'd she die?" or whatever. And nobody was saying HIV or AIDS. Nobody was saying AIDS, and it was more so, "Well, she was fast," and all that other stuff. And she had kids, and they were making sure that the kids didn't have it and all that other stuff. And this is just a bunch of women in my family talking. And I'm just young, and I'm like, "Why is there such an undertone?" But it wasn't until later that I found out that she died from AIDS. And she recently had her son, and her son was perfectly fine, born without it. And yeah, that's when I understood like, "Okay. AIDS can kill you. HIV is real. There's ways that other diseases can be passed to a child and whatever, so." Yeah.

S1 00:47:06.011 And what about the education that you-- or your knowledge about HIV?

S2 00:47:09.913 At the time there was no knowledge [laughter] there. It was just me being in fourth grade accepting it. There wasn't a big emphasis on it, but there was definitely a lot of, "Wear a condom. Wear a condom. Wear a condom." And it was for the older kids. And kids, us, would be playing with them and not thinking that there's anything of it. But yeah, it seemed like the three letters on the buses driving by and dot org behind it or anything like that. Those are the things that you start to see and associate, so. I remember when I first seen a PrEP bus thing. What do you call those? Billboard on the bus. And I was, "What the hell is that?" And it's like, "Protect yourself from HIV," and I was like, "Oh. Okay. Well, I guess HIV's cured," and that's what I thought at the time. It was just like, "Okay. Well, it's cured. It's not that serious. We're getting more advanced, and it's going onto the bus," but here I am, working full against HIV [laughter].

S1 00:48:08.257 How long have you been in this type of work?

S2 00:48:09.511 I just started this job last year around this time, been here about a year. But I've always done sex education. I've worked at Adult World and tried to have groups there. [inaudible] over the counter and stuff like that. So, yeah.

S1 00:48:24.378 Interesting.

S2 00:48:24.761 Yeah.

S1 00:48:25.635 Wow, pretty cool. Can you tell me about your personal practices with respect to using condoms? I'm interested in hearing about such things as how regular you use condoms, whether you use condoms for some types of sex but not others, are there certain circumstances where you don't use condoms?

S2 00:48:46.508 I've always used condoms except for oral. Even when I was the bottom, condoms were used. And I'd make sure. I'd be reaching back there and stuff. There was one guy - I still see him sometimes in [Upstate NY City 1] - and when I see him I want to stop the car, put it in park and catch him [laughter]. He was [inaudible] in my last two years in college, and he kind of tried to stealth me, basically. That's when you take off the condom in the middle of sex. But he didn't try to take it off. He tried to break it. So he would try to break it with baby oil, Vaseline. He would use all these other things. And I knew for a fact this was what he was trying to do, so him doing that and I felt the snap from the elastic [laughter], and I was like, "No. That's it." [inaudible] flew right off and I was done for it. And yeah, I've always tried to use condoms. Even when it felt like, "I can't catch [inaudible] from this white guy," or, "I'm not going to catch anything right now," I still used a condom. And I would make sure. I would almost be turned off at the fact that the other person wants to be bareback and stuff like that. Not because it's like, "You want to be bareback," but it's like, "You're putting too much of emphasis on something that's going to last 10 minutes. And I'm going to wear this condom so you can kick me out now or you can do this. It's up to you." But it's just annoying having to tell someone, "Well, I like it bareback. I want to do bareback." "Okay, that's fine. But I'm just here to [inaudible]. Not in you." So, there's just, yeah. But I've always used a condom, I'd say. Except for oral.

S1 00:50:35.679 Except for oral?

S2 00:50:35.894 Yeah.

S1 00:50:36.844 Okay, cool. And you and your partner also practice using condoms?

S2 00:50:43.597 No. Before we got together we both got tested for HIV, and STDs, and things like that. And then we didn't use a condom. We haven't used a condom. Yeah, we never used a condom.

S1 00:50:57.072 Okay. All right, I was about to say, "Not to get in your business," but I guess I am in your business [laughter]. I was just thinking back to what you said earlier in the conversation about your partner testing for HPV, and you having to take care of that before you start being sexually active. How long did that take before--?

S2 00:51:16.621 Before we actually did have sex?

S1 00:51:18.180 Yeah, [inaudible].

S2 00:51:19.024 So we would kind of have sex when we first got together. And then that's when he noticed it. And then it took us about, I would say literally till about the last day that we were together to actually have some type of intimacy. Because it was a lot of-- we both had someone's confidence, and then making sure that I don't have it, and if I do have it I have to check for it. Getting my medical records, seeing if I had gotten vaccinated and all that other stuff. Yeah, the first time that we had sex in the past six months was yesterday. So that is a lot of-- there was just a lot of rebuilding to get to that point. And even at that point, it was like you're still not ready for it, you know what I mean?

S1 00:52:14.073 Mm-hmm.

S2 00:52:14.579 So, yeah. But it was just a lot of-- I would say about, maybe about eight months. Eight months for us to even consider being resexual, or just having that initial fear of spreading something back and forth. Where, if you have it, you don't have it. If I have it, I don't have it. Who's resistant to it, who's not? Yeah, so it was just--

S1 00:52:41.731 And when you were tested for HIV before you started a sexual relationship with him, were you tested specifically going into this relationship, knowing that you, at one point, would want to have [crosstalk]--?

S2 00:52:51.949 Yeah, that we definitely wanted to have unprotected sex, yeah. Probably around October, he ended up getting really sick, like a fever. And he wasn't talking, he wasn't saying anything. And I was like, "What's going on?" And he was having his inner turmoil and he was thinking because right before we got together he had sex with someone else. And I told him not to do that, this is the [first thing you get?] HPV from. So thank God it wasn't HIV, but it was a lot of, "I think I have HIV. I'm scared. I don't want to say anything to anybody." And then I asked the question. I was like, "Are you scared you might have HIV?" And he's bawling out, crying. So I took him to the hospital, and then we went to the hospital and we just got the tests. My test came back and they were like, "You're fine, anything like that." And his test, I guess because he was sick, they were like, "Your test is still running." They were like, "We're still trying to figure out." So that was just like--

S1 00:53:40.736 Oh my gosh, [inaudible].

S2 00:53:42.345 Yeah, why didn't you even just wait until they come back? But yeah, it came back negative for him as well. And then we were like, "Oh good." And it was just HPV that we had to take care of later on, which we found out probably two months later. Yeah.

S1 00:54:01.396 So we already covered that. I was going to ask you about other sexually transmitted infections. But we haven't talked about other than HPV. Have you ever had one or worried that you had one?

S2 00:54:15.256 I'm only worried now that I might have one only because I just engaged with people that I don't know. But I've never had an STD, and I don't feel like-- having that initial, "Oh my God, I have something." Let [inaudible] one me. And call somebody [inaudible] [laughter], like, "God damn you, what did I do?" But that blows over. Yeah, no initial STDs or anything.

S1 00:54:44.830 Sweet. And do you worry at all about contracting HIV or anything else?

S2 00:54:50.994 Yeah, still. Even through oral. So even though I know that the chances are extremely slim, I'm still looking at the person's lips, and their face, and all that other stuff. This line of work definitely freeze-- it fries you mentally about sex. It makes you not even want to touch people on the bus. But I wouldn't think so, yeah.

S1 00:55:17.000 Okay. On a scale of 1 to 100, if 0 was the lowest chance and 100 was the highest chance, what would you say is your risk of getting HIV in the future?

S2 00:55:32.277 Now that I know that, it's 50/50 [laughter]. I would say for me probably like-- I'd say probably like 45. 45%, or on a scale of 45, which is-- I'm on Grindr and these apps to hook up. I'm not here to date or anything like that, and if I do do that it's because I'm single, or I'm not with my partner anything like that. So me getting the partner, gauging them, questioning them, usually gets me really nowhere, because a person is so intimidated, or they're like, "You're asking too many questions, da, da, da, da, da." And it's like, "You just don't [give a fuck about yourself?]." And I'm not going to put myself in that situation where I'm going to jeopardize myself, especially from people who are anonymous, or, "Come on in," and things like that. Like, "Let's just do it bareback." It's like, "Yeah, the idea's hot. It's fun and all that, but I'm not going to risk myself." It's tempting, especially when you're in America. You can do whatever you want, get to wherever you want for free. But then let me just not do that because I don't want to risk that. So I would say 45, and that's a high 45.

S1 00:56:56.324 I was going to say that's kind of high up. I mean, I'm just thinking-- yeah. You're thinking almost a 50/50 chance every time you go into a situation, so you do think about it quite a bit.

S2 00:57:11.265 Yeah. Definitely. Yeah. Definitely. Even if it's oral. Even if it's a older white male or if it's a younger white male, I'm still thinking about it, just because I know that they get around. If you've been on Grindr, I've seen your face before. I know you get around. Every gay man's going to get around, especially in a small town, so yeah. That freaks me out just a little bit.

S1 00:57:38.808 That's the second time you mentioned race. I was just curious, your idea about HIV, is it different when you think about race and/or age?

S2 00:57:51.015 Definitely. [inaudible] [laughter].

S1 00:57:57.555 Sorry.

S2 00:57:57.753 So I know the rates of minorities and the rates of white males. But it's like, for the type of person that I am, I know that I might slip up and say, "Well, let's just whatever." But it's like, I want to be cautious through all spectrums. And usually, a lot of times when I'm on Grindr and things like that, it's a minority who's prostituting themselves. And it's usually an individual who's not in the right state of mind, or they're not doing what they need to do, or they're visiting in the city, or they're hosting at their hotel, looking for cash. And a lot of the gay community in [Upstate NY City 1] already knows each other. Not to say that minorities are the ones who have HIV or anything like that, but I feel like, obviously, it's higher in these demographics. But when it comes to white men, I feel like there's more of an opportunity to catch other things, like herpes, gonorrhea, chlamydia, syphilis, hep C, just because there are things like injection drug use that happen more here than they do here. I mean, of course injection drug use happens here, but in this area, it happens in this demographic way more. And I feel like a lot of older men, from 35 and up, don't think about hep C, or they don't think about contracting something from someone younger. But a lot of times when we're testing, it's usually someone younger who's popping up with these things, and they're either exposing themselves to other people who are in their demographic or in their age, or they're exposing it to other older men. And these  other older men are usually looking for BBC, big black cock. And it's like, you take that, and you go here, and then you got individuals who want to prostitute themselves or just get their rocks off like, "Get it. That's fine." But they're engaged with them so it's just-- the way my mind works is just like, "Who got it [laughter]? Who has it? Who doesn't have it?" But I just feel there is prejudice in my head, and that comes from being where I'm from and being told by specific people that this demographic has it and this one may have it a lot less. So, yeah.

S1 01:00:35.707 Yeah. Well, thank you for being honest. I mean, I think we all have prejudices and biases, and they're hard to talk about sometimes. And so I appreciate you being honest about it because it's real.

S2 01:00:47.116 Yeah [laughter].

S1 01:00:48.808 It is real. All right. So let's see. Okay. So the next set of questions is about what you know about HIV, PrEP, which is a daily pill to be taken to prevent HIV. And I already suspect that you know quite a bit. And so the questions that follow, I'm going to use the term PrEP to refer to pre-exposure prophylaxis because it's so much easier to say, for sure. Well, you tell me what you want to, but don't feel like you have to [crosstalk].

S2 01:01:20.796 It's PrEP. Small, blue pill you take every day, protects you from HIV, Truvada. They were used in the '90s and now they're using it now. It works, 99% effective. Yeah. [crosstalk].

S1 01:01:36.422 And who's it for?

S2 01:01:37.385 Who's it for?

S1 01:01:38.035 Mm-hmm.

S2 01:01:38.791 Anybody who is having-- [anyone's] high risk, or injection drug use, or prostitution, or work in a facility where you might be exposed to it. So there's PrEP, and then there's a nPEP, and then there's [oPEP?] [inaudible].

S1 01:01:54.058 Do you discuss PrEP with friend, parents, family?

S2 01:01:58.248 I usually talk to people who are-- I usually bring it up when people are talking about not wearing a condom, or not seeing that condoms are comfortable, or--

S1 01:02:10.431 So in terms of-- so you talk it about it like you're educating?

S2 01:02:13.947 Yeah. So I always have friends that are all talking about sex, always talking about sex. And they're like, "Oh. Well, I don't want to use a condom, da, da, da, even with a girl." I'm just like, "Okay. Well, there's PrEP. There's things that you can do to protect yourself. I don't want to hear it. Because if you show up with HIV, that's on you." Yeah. Usually, I will just-- a quick spiel. I'm like, "Yeah, a little blue pill. You take it every day. What's wrong with that? You taking vitamins. You put on tampons. You put on your drawers. It is what it is. Just take it every day." It usually does work. People are like, "Whoa! Wow! Really? That's amazing!" I'm like, "Oh, well [laughter]. Use it. Use it."

S1 01:02:52.635 Right. Do you mostly hear about PrEP in the media or do you hear it all [inaudible]?  Or some of the sites even that you--

S2 01:03:05.026 Definitely on the sites. I see a lot of-- well, I used to see a lot of it. I haven't been on it that long since, say, now. But I see it more so targeting gay men. So when people do bring it up they're thinking it's only for gay men as well or they're thinking it's only for people who are addicted to drug use or have hep C and things like that. But it's not mainstream enough. It's not comfortable across the spectrum for everyone. Straight white women to asexual Middle Eastern people. It feels like it's specifically for gay men and then on top of that, gay black men. And, yes, that's who we need right now, is to be on it, but it doesn't feel like it's a accessible thing to everyone. So, yeah.

S1 01:04:04.883 Interesting. Are there guys that you have met in the past, or whenever, from some of these sites-- do any of them bring up PrEP, or talk about it, or say that they are or not?

S2 01:04:18.470 No. If they don't have it on their profile, they aren't really interested in protecting themselves or they're not really thinking about contracting HIV. There's just a lot of barebacking going on [laughter] and not everybody's so enthused about--

S1 01:04:37.680 Do you want some water? Sorry.

S2 01:04:38.654 No, I have water. Thank you. Just that and--

S1 01:04:44.788 Do people ask you?

S2 01:04:46.425 Ask me about PrEP? Like, am I on PrEP?

S1 01:04:48.843 Yeah. I mean, well, the men that you meet, do they ask you, "Are you on PrEP?"

S2 01:04:52.403 No.

S1 01:04:53.150 No?

S2 01:04:53.213 No.

S1 01:04:53.842 Wow.

S2 01:04:55.174 I'm usually the first one to-- so [inaudible] I don't take off the condom. I don't do anything like that. I don't play games and you got to send a face pic. Yeah, I don't play that. Two fingers on your chin. You heard about that?

S1 01:05:10.935 No.

S2 01:05:11.961 So [laughter] me and my friend were like, "You know what? I'm not being catfished no more. I'm not going be popping up at some random person's house. Send me a picture with two fingers on your chin. So a lot of the times you have [inaudible]--

S1 01:05:25.357 Oh, okay.

S2 01:05:25.813 I'll show you a picture. [inaudible] I don't play that. I don't play that and just for my safety and for knowing anything or if anything was to happen. Say I was to get exposed to anything, I know your face. I'll see you.

S1 01:05:39.318 So you're saying you ask them to take the picture with two fingers on--

S2 01:05:41.660 Yep. Two fingers so I know it's them, yeah. And it sounds crazy and stuff like that but--

S1 01:05:45.654 I like it actually.

S2 01:05:46.509 --it works. It really does work and, yeah, usually they're more concerned about a face pic, or how big your dick is, or what do you like to do instead of are you HIV positive. I don't think I've really come across anybody who asked me what your status was first. It's always me asking me asking them, and they're like, "I'm clean." I'm like, "Okay. Well, what does clean mean to you?" And then that just makes everybody flaccid [laughter]. So it's just like, "Whatever. You'll do what you want [laughter]." It's hard.

S1 01:06:27.360 I'm liking this [laughter]. Okay. So do you know anybody who's actually taking PrEP?

S2 01:06:33.756 Yeah, I know a few people.

S1 01:06:34.967 All right. And close to you, I mean? Not just in work.

S2 01:06:38.818 No, I wouldn't say so.

S1 01:06:40.662 No?

S2 01:06:41.139 No.

S1 01:06:41.071 None of your friends?

S2 01:06:41.555 Hmm-mm.

S1 01:06:43.246 Okay. So have you ever considered taking PrEP? And what factors affected your decision-making?

S2 01:06:55.828 I did consider taking PrEP. I was in a relationship, so that was one reason why I didn't feel like going outside of my relationship. Even when he was telling me to go out, I was like, "I don't really want to do that." I thought about taking it in the last few weeks, just so I can protect myself if I was to go out and have sex with anyone else. And I'm going to DC so I'm really thinking about it. But I'm like, "I could just wear a condom because I don't feel like putting my dick in any hole [laughter]." But I've thought about it. But what's stopped from it was just the fear of putting something in your body, something that you don't really know what it does. There's so much going on in the world right now that I don't even want to trust my mother. It's just so crazy right now. Being black and that history, and things like that, it really messes with you. Being a young black man as well, it really makes you think like, "Okay. Well, who am I supposed to believe? Am I going to believe a conspiracy theory or facts?" It sounds stupid, but it's a lot of hearsay and you can, of course, do your own research and stuff. But because of the way Google works, because of the way Wikipedia works, or anything like that, it's just nothing feels like it's supposed to be.

S1 01:08:29.193 I see. So are you thinking that with the medication is not-- they're not purposely not being completely forthcoming with what could happen to your body by taking this medication?

S2 01:08:44.148 Yeah. It's just like, "Take this pill and protect yourself from HIV." But, even though it's been there since the '90s, where are these facts? Let us see these facts. Let us know what is-- what are these outcomes of these pills? Is there going to be [Resprinol?], am I going to grow a titty? Let me know what's going on. I can't be having my kidneys shut down while I'm in the middle of nowhere. I need to know what these pills will do. And they're like, "Okay. Well, it works. And you've got to keep taking it. And it'll build up an immunity in your system." I want know if this thing is making me super prone to [inaudible]. I don't know what it's doing. There's just not a lot of support to protect people's fears. And I'm hearing specifically from the black community, because of African American history, like, "I don't trust this government. I don't what that pill's about. That pill came out of nowhere." And, yeah, I don't-- I personally, I don't feel comfortable taking a pill as well, especially if I'm not sick. I have a hard time even telling clients, "It's not a vaccine. It's not a vaccine. It's not a vaccine. It's protecting you. It's coating your cells, it's protecting your cells. It sounds great." I can explain it and it sounds like, "Yeah. Everybody should be on this. It should be pumped in the waters if anything." But I just don't feel comfortable-- black people don't feel comfortable taking another pill being given to you by a white doctor, on top of that. It just-- yeah.

S1 01:10:26.129 I get you. Honestly, we've had a problem about [inaudible], a lot of people feel that way, and I think you have a really unique perspective on it because of what you do, and who [inaudible] you're able to talk to.

S2 01:10:36.833 Yeah. I get a lot of people, black [inaudible] like, "I don't want it." Like, "Okay, you got it. You got it. You got it."

S1 01:10:43.593 Yeah, yeah. It's real too.

S2 01:10:46.080 And it's accessible enough-- I don't know [laughter]. It's just accessible, yeah.

S1 01:10:53.761 Right. Well, thanks for that. Okay, so I'd like to ask you about some things that others have told us are important to them when they have considered using PrEP. There is no right or wrong answer to any of these questions, and it's fine if you don't really know-- don't really have anything to say in response to one of them. Just please answer each question as fully as you can. So, what if anything, do you know about the cost of PrEP?

S2 01:11:21.577 The cost of PrEP? I know that recently they allowed it to be free for [only the?] state residents, I think. I know there's loopholes that these insurance companies are doing to basically get people on to PrEP. There's cost adjustments. There's a way for everyone to get PrEP, whether they have insurance or not have insurance, or if the insurance will pay for it, or if they're DL, or anything like that. There's ways for you to get it discreetly and to pay for it abundantly. There's ways, so.

S1 01:11:58.298 Okay. Have you heard about people having trouble paying for it?

S2 01:12:04.014 Usually--

S1 01:12:05.233 What was that?

S2 01:12:05.929 Usually, they're like, "My insurance doesn't cover it. My insurance doesn't cover it." And then I'm like, "Okay, well go to Upstate and see what they could do about it." Then they'll get it. I don't know what they're doing at Upstate [laughter], if they're [inaudible] or whatever. But it usually doesn't-- it's usually, "I can't pick it up from the pharmacy." You'll get the prescription but you can't pick it up. And what else have they said? I've had someone say, "I don't even know what this is." I was like, "But you went to the whole doctor and spoke to them for a whole hour [laughter]." Usually, it's a lot of, they can't pick it up from the pharmacy. They can get to it, but they can't pick it up from the pharmacy. Or if they do, they'll end up getting a bill for just the hospital service for them to do the intake to start PrEP. And then they get the Truvada.

S1 01:12:56.719 Wow.

S2 01:12:57.296 Yeah, so that's usually it. But usually it's a case of just sending in the letter and then they'll fix it or whatever. But I don't hear too much about that.

S1 01:13:12.316 Would it worry you to use your health insurance to pay for PrEP?

S2 01:13:15.924 No.

S1 01:13:16.529 If you decided to do it?

S2 01:13:17.546 Mm-mm. I think I'd raise hell if they were like, "[inaudible]." Like, "Excuse me?"

S1 01:13:23.193 Yeah, yeah. Do you think a lot of your clients worry about that? Or people that you talk to?

S2 01:13:30.051 Worry about not getting it?

S1 01:13:31.517 Yeah. Well, worry about, not necessarily not getting it, but worry about disclosing, I guess, to your insurance company or their employer maybe finding--?

S2 01:13:38.661 Yeah, yeah. That happens way, way too often. It's almost scary because, because of that I know that they're engaging in a risky behavior. So you engage in a risky behavior, you can do that, but it's more detrimental for you to actually get it written on a piece of paper that these are your habits. So we're in a college town. A lot of teens are coming out. There's a lot of white teens who are from really religious backgrounds and they're like, "Oh well, I can't let my parents know what I'm doing." Or they just don't know how to go about it, or they want to get on it but they can't. There's a lot of-- because of this, I know that you're doing that. Not to say, or assume, but this is what's happening, it's happening.

S1 01:14:23.876 Wow. Okay. I'm trying to adjust some of these questions because I know you have a lot more familiarity with PrEP and stuff so. We've got to make sure we cover everything. So you pretty much know where you can go to get access to PrEP, I would assume. Do you think it's easy to get on PrEP?

S2 01:14:52.403 To get on PrEP?

S1 01:14:53.094 Yeah, for people--

S2 01:14:53.767 I think it's easy. I mean, the only problem would be setting up an appointment at a primary care or set up an appointment upstate. There's no specific walk-in clinics just for PrEP. I mean there is downtown, but you still got to go by appointment. But if there was just like a building dedicated to PrEP and HIV or just specifically for HIV. Like we are here and stuff, but we do so many other things that we don't have enough-- we don't doctors to just prescribe something, so. If there was a [beacon?] for it, I feel like it would be more accessible.

S1 01:15:30.419 Okay. I think a lot of these are what we've been talking about, and all your suggestions, and think that your insights have been very helpful so far. So you've been great, so thank you again. Let's see. What, if anything, have you heard about what you have to do to take PrEP? Do you have to take it daily? Weekly? Monthly? Liquid? Pill? Implant? Shot? Doctor's office? Do you have to go to the doctor [inaudible]? We talked about most of that stuff, but if you have anything you want to add to.

S2 01:16:08.326 I mean, if it could be an implant that'd probably would be a little bit scarier. But if it would be more effective, it'd be the one-time thing. Every three months you get your little implant. It's easier, and it's less worrisome when you have to take something every day because if you're thinking like, "Oh my God. I didn't take it." Or a lot of people-- a lot of our clients are like, "I didn't take. I didn't take it." It's like, "You have one job [laughter]. Just take the pill." But it happens, and I get it.

S1 01:16:44.474 Yeah. Would you say you feel as though you would be able to take PrEP if you decided yourself without any problems?

S2 01:16:53.557 I have a anxiety about it. But I feel like I can take it. HIV isn't the STD that I'm scared of. I'm scared of herpes. [inaudible] back because that's visible. But yeah, I think I can take--

S1 01:17:16.370 Where's your anxiety lay exactly? I think I know what you mean, or what you're-- what would your anxiety be from taking it every day?

S2 01:17:28.985 Just I don't know what this pill is doing, and I don't know if the pill is working. I might come across that one individual who is Truvada resistant. Anything can happen. People take their medicine wrong, and then you could just be in that situation is just-- I'm crazy, so I've been in situations where I'm like, "Is this really happening right now? Seriously?" So I feel like things like that can happen again.

S1 01:17:59.038 Okay. I got you. Would you think about taking PrEP if it were an injection rather than a pill? Or an implant even?

S2 01:18:10.997 Oh, that's tough. I'd rather the pill because I can stop it at any time, and then continue at any time. After that, I'd say the implant would be better than an injection because once you inject something, it's all over you, and you can't really just take it out. Yeah.

S1 01:18:34.518 Okay. So what do you know about the effectiveness of PrEP? I think you may have mentioned it, but if you don't mind.

S2 01:18:44.057 It's like 99%. Only like one case where individual contracted HIV from it. I think it was in Texas or something. It was a long time ago, though. I think.

S1 01:18:57.432 Okay. And how effective would something have to be for you to be willing to take it?

S2 01:19:05.275 I just want to know the side effects. And don't bullshit me on headaches, vision blurry, stomachache. Get over that. I get that every day. I need to know what is going on. What are the long-term effects? Like I don't want to be sitting on my couch and then, "I think I've been taking Truvada for the past five months. Da, da, da. Cool. How do I deal with that?" But if it was extremely effective, and if I knew that it was more beneficial than what it is, I feel like I would be all down for it.

S1 01:19:40.660 Okay. So if you knew for sure PrEP would cut your risk of HIV like by in half?

S2 01:19:48.830 Yeah. Yeah. I'd still use a condom, but I feel like the likelihood of me actually committing to using a condom would be a lot less just because I know that, "All right. Well, you're not going to get HIV." And there aren't any symptoms that you see from an individual, so yeah.

S1 01:20:10.072 Great. So what are the most common side effects you can talk about? Possible side effects? What would be [inaudible]?

S2 01:20:20.006 Headache, nausea, heartburn [laughter].

S1 01:20:23.327 What would be the most concerning to you?

S2 01:20:24.385 The most concerning? I'd say the headaches and abdominal pain. That, to me, would be-- that's you. That's like what makes you or makes you stop. I feel like people have too much going on in their mind already to be with a headache all day. Like am I going to sacrifice a headache for some ass? To me that just-- priorities [laughter]. Yeah, yeah.

S1 01:20:56.553 Cool. Do you think your friends and potential partners look favorably or badly on people who take PrEP?

S2 01:21:06.845 I don't think they really have a-- I don't think they have a bad idea of it. I think it's like when it first came out it was, "Oh, look at that. It's just a slut pill." That's what we were saying. It's just a pill for you to be all--

S1 01:21:24.482 Right?

S2 01:21:25.798 Now, I feel like it's becoming more of a relevant thing like, "All right. It works." People were, "It's consistent, and it's still out there. And there haven't been any recalls," or stuff like that. But I think they would definitely have no bad feelings about it or feelings [either way?] about someone else using it, so. You can't get mad at someone for using a condom or using a pill, so.

S1 01:21:50.433 True. But if you were taking PrEP, would you tell potential--?

S2 01:21:53.626 I think I would tell people, yeah. Yeah. Definitely.

S1 01:21:57.318 Why is that?

S2 01:21:58.739 Just because I can now be the spokesperson for it [laughter]. I can [say?], "Well, this is frontline. This is what it's doing. And of course, we're all different. I mean, it may work better for me than it does for you, but you'll never know until you try." But yeah, I just don't want to be that guinea pig [laughter].

S1 01:22:15.531 Right. So anybody that you've met, like any potential partners, if they were taking PrEP, would you want them to tell you or would you expect them to tell you?

S2 01:22:24.640 That wouldn't matter to me. It's the actual status. Knowing whether or not you have HIV or an STD is what I want to know, not if you're taking PrEP. If you're taking PrEP, that's fine. I'm still going to use a condom. Just know that. But yeah, it doesn't matter to me if they're taking PrEP or not.

S1 01:22:44.198 Okay. Okay. Well, the next set of questions are for people who are less than 18. So now, I'm going to give you a PrEP fact sheet. You probably know all about this. I can see it right on the wall [laughter]. Perfect. So there you go. That's for you. Feel free to take it with you at the end of this interview. Take a few minutes to read it. If you have any questions about what something means, please let know and I'll do my best to answer them. But you probably can answer them better than I can [laughter].

S2 01:23:24.475 I try.

S1 01:23:26.151 But yeah, take a second.

S2 01:23:33.915 See, there's this part right here. There [is a program stating the?] drug company that makes PrEP [inaudible] the cost associated with it, basically helps you pay for it. That's the hard part. People don't understand the jargon for health insurance and no cost and margins and all the-- it's comical. You know what I mean? They're not looking, they're not studying this stuff because it's not their job. There are individuals at risk because they don't have a job. There's [a costing loophole?] but there's no clarity, I feel, for insurance, or for how to obtain it through your insurance, or that it can be obtained discreetly and it's just only word of mouth. And then the individuals who are connecting most people to it can only say so much and then when they feel comfortable with that person, now they brought in another person and they're just expecting to get this pill and just be in and out and not really build that relationship with that doctor, so.

S1 01:24:36.184 How will you fix that though? I mean, do you think it's in terms of marketing and getting that out there?

S2 01:24:40.664 I feel like it's marketing. I feel like it's actually explaining to people, and simplifying the words, and simplifying how things are going to work if this is the route that you take. It's a lot of big words. I've worked for a health insurance too and we were trained for two months and still, I was like, "[inaudible] knowledge." I'm like, "I don't know what the heck this means. [inaudible] Google and stuff." And it's just because people don't know. People don't know these things and we're expected to know these things and the reality of it is nobody reads their health insurance. Nobody's going read the whole thing, it's like five pages long.

S1 01:25:23.503 Yeah. [inaudible] like you said.

S2 01:25:25.269 Right. It's tiring. It's unfair. But, yeah, I think it's just marketing, being clear at what insurances cover it, what insurances don't cover it. What will probably be an even better list, how much it costs, what the costs would be of this for that, if not that for this. Yeah. I just feel like it would be easier for people to get associated with it.

S1 01:25:50.341 Yeah. I mean, personally, I'd like to see the medical community actually be more educated because I have several friends that I've asked them about it and they said, "Yeah. My doctors never brought that up to me." [inaudible], "Who brought it up to your doctor?" And he's like, "Well, apparently he didn't really want to talk about it." And he felt like he didn't want to talk about it because he didn't really know about it for some reason.

S2 01:26:12.060 Right. I feel like the doctors are usually just consumed by the service that they're providing and not really the insurance and all the other stuff and explaining things to clients is not-- because it's not their job. I mean, it's not but I feel like there's not a lot, like you said, not a lot of competency for PrEP or engaging with the community about making sure that they actually know it. So, yeah.

S1 01:26:44.100 I agree. Okay. You doing okay?

S2 01:26:48.411 Yeah. I'm good. It's up to you. You let me know.

S1 01:26:50.049 No, no. [crosstalk].

S2 01:26:50.653 I can [chat?] all day. I just [inaudible] [laughter].

S1 01:26:55.434 Okay. Just seeing where we are here. So having heard the information about PrEP, do you have new questions or comments that come to mind? I just talked about that [inaudible]? Does this information change your mind at all about thinking about PrEP?

S2 01:27:20.406 Not really. I mean, these are things that if you really wanted you're going to find out anyway, or you're going to do that research. Those feelings are, they're there [laughter]. They ain't going to go nowhere.

S1 01:27:34.642 Yeah. What extent do you think others in the Gay-Bisexual-Trans community know these facts about PrEP?

S2 01:27:43.256 I'm sorry?

S1 01:27:43.126 In general.

S2 01:27:44.221 What do they know?

S1 01:27:45.008 Yeah.

S2 01:27:45.266 I think they just know that it's a pill that can stop an STD. They don't know what STD. They don't know how effective it is. They do know the side effects. But they don't really know-- I don't think they-- I don't think it's clicked at who's it intended for. I don't think that gay people know like, "This pill is for you." Like, "This is for you." I think they're just accepting it as just something that everyone can access. Because this is something that the times have given and that the times have led us to this. And so we're just-- I don't know, I think people just pick and choose what they want to know and hear, so. There's not that much of an investment into knowing what it's about.

S1 01:28:43.022 So we're kind of wrapping up, actually, believe it or not, in terms of the interview phase, so thank you. I really do appreciate all your honesty, I mean for coming and all you have to share and stuff. And I think you're unique perspective and what you do and stuff is really valuable I think to-- or will be valuable to our study. So you have set the the bar pretty high.

S2 01:29:02.531 Cool [laughter]. I try, I try.

S1 01:29:05.878 So I actually have a [inaudible] I'd like you to do. So it's a little bit different than what we've been doing so far. And then finally I have that self-administered questionnaire to give you. And that should take you about five minutes or so. Okay?

S2 01:29:19.631 Okay.

S1 01:29:20.850 So let me find my index cards. So here we go. So I'm going to give you these cards, okay. Now, I've written on these index cards on the factors that people have told us that are important to making a-- important to them in making decisions about PrEP. So take a look at them, and then what I would like you to do is put them into order from the absolute most important aspect of PrEP to the least important. So most important on top, least important on the bottom.

S2 01:29:58.925 For me or for--?

S1 01:30:00.631 What you think [inaudible]. And then I'm going to give you some blank cards in case you think of something that is also important, and you can write them down here. So here's some blank ones if you think of something that is not there that I didn't write down that you think is important that needs to be included. And then, if you do that, then also put that into the ranking.

S2 01:30:28.865 PrEP can be taken as an injection? Is that--?

S1 01:30:31.602 No.

S2 01:30:32.026 --something. No?

S1 01:30:34.599 So these are not just necessarily you though. Let me read this [laughter]. Okay. I've written on these index cards the factors that people have told us are important to them in making decisions about using or not using PrEP. Please take a look at these and let me know if you think any are missing. We can write other things on these blank index cards and include them. Now, please place-- no, that's written wrong. Please put these in order for me in the absolute most important aspect of PrEP to the least important, with the most important on top and the least important on the bottom. So not necessarily just what you think but people in general. What do you think-- what do you people think are the most important? And when it says, "How you take it," so is that important? Pill opposed to injection opposed to implant. Is that something that people think about? And how important do you think it is compared to those others?

[silence]

S1 01:33:14.311 Okay. So, you think how you take it is the most important? Pill, injection, etc. Effectiveness. Side effects. How often you have to take it. Stigma. Cost. And ease of access. Correct?

S2 01:33:33.145 Mm-hmm.

S1 01:33:35.413 If you could put a button to that.

S2 01:33:36.959 Do you think the [inaudible]. I'm like, "Did I? No? Me? No [laughter]."

S1 01:33:41.366 Awesome. Thank you. Okay. So I'm going to give you this sheet. And so this is our second exercise. So this exercise goes like this. So imagine that I'm going to give-- that I'm going to a flip a coin, and you'll get what is indicated in the heads column if it comes up heads, and you'll get what is indicated in the tails column if it comes up tails. Which row would you pick from the table that you are looking at? You can only pick one row.

S2 01:34:10.608 You guys are sneaky.

S1 01:34:12.450 Yeah.

S2 01:34:13.888 You can only pick one row?

S1 01:34:16.823 Yes. So you can only pick one of these rows. And so if I flip the coin and it lands on heads, you get that. If it lands on tails, you get that. Does that make sense?

S2 01:34:27.722 Yeah. I'd say row two.

S1 01:34:35.510 Row two?

S2 01:34:36.196 Yeah.

S1 01:34:36.479 Perfect. So you picked row two. So if it landed on heads you get a 100, and if it landed on tails you get 30. Perfect. Awesome.

S2 01:34:52.311 What's that about? What's that supposed to be?

S1 01:34:54.253 It's a risk assessment, basically.

S2 01:34:57.567 Okay. I see, okay. [inaudible].

S1 01:35:00.789 Cool. I'm glad it [inaudible]. Honestly, when I first read it-- the first time I saw this, I was--

S2 01:35:10.723 What?

S1 01:35:10.922 --like, "Huh?" And I think we changed the wording around too, for the instructions, and once it happened, it made more sense to me.

S2 01:35:17.997 Oh, cool.

S1 01:35:18.193 But I think the first time I read it I wasn't getting it.

S2 01:35:20.707 Just risk [quiz?]. And I was like, "Wait. What? Okay. All right."

S1 01:35:23.305 Yeah. Yeah. But then I could see it, I was like, "Oh yeah, I get it." So now the last one we're going to do is ask you to do this self-administered questionnaire. And just take your time with that. Do you need privacy? You want want a pencil?

S2 01:35:38.731 Nah. I have a pencil.

S1 01:35:40.687 Okay. And then when you're done, you can fold up that questionnaire, and you can put it into this envelope.

S2 01:35:46.589 Okay.

S1 01:35:47.435 And then we're [done on our side?].

S2 01:35:49.401 Really?

S1 01:35:50.128 Yeah.

S2 01:35:51.389 What time is it?

S1 01:35:52.180 I know.

S2 01:35:52.337 6:30.  Let me get this so you can all go home.

S1 01:35:54.796 No. Me? You've been working all day. I'm fine. You take your time.

[silence]

S2 01:37:23.049 I don't count the last sexual partners though. Women? I don't really count them as sexual partners.

S1 01:37:31.030 That's fine.

S2 01:37:32.358 Okay. I feel like I'm lying [laughter].

S1 01:37:34.978 No, you're not lying.

S2 01:37:36.901 I looked at one, and I touched it [laughter].

S1 01:37:44.529 You don't have to count if you don't want to [laughter].

[silence]

S2 01:38:01.955 Don't you hate that you actually have to write things out like this? Estimate if you're not sure, but oral or anal sex, we mean your penis in a partner's mouth or anus. Ridiculous. Ridiculous. It's cool, I get it.

S1 01:38:16.537 Some people [laughter]--

[silence]

S2 01:38:47.058 In the last month, you mean this month? Or July?

S1 01:38:52.614 What's the question, I'm sorry?

S2 01:38:54.005 Number of male partners in the last month.

S1 01:38:57.153 Today's the 14th? Yeah, I [inaudible].

S2 01:39:02.263 I'm [inaudible].

S1 01:39:02.963 Yeah, August.

S2 01:39:04.673 I can't believe it's August.

S1 01:39:05.234 Yeah [laughter]. Maybe mid-July to where we are now.

[silence]

S2 01:40:15.578 Okay.

[silence]

S2 01:41:04.751 This is forward. [In the last six months?], how often do you use condoms when having any type of anal sex? I only used a condom once in the past six months, but-- okay. Less than half the time. I just feel like that's going to be like, "Oh [laughter]." I feel like it's going to skew things. There's some-- not misleading. I just [inaudible] for relationships.

S1 01:41:36.448 Oh, okay. I'm glad you mentioned that.

S2 01:41:37.993 Yeah. That's it.

S1 01:41:43.040 What's the exact question?

S2 01:41:44.963 In the last six months, how often do you use condoms when having any type of anal sex? So I've only had one partner outside of my boyfriend, and we use a condom, but--

S1 01:41:55.106 I see what you're saying. Well, I mean, that's what it was, so. But yeah, you can make note of that, or-- but they'll catch that in the transcription, too.

S2 01:42:08.500 Cool.

S1 01:42:09.189 It's a good point though.

[silence]

S2 01:42:55.300 Yeah [laughter].

[silence]

S2 01:43:19.676 Marijuana's not a drug now [laughter]? That's what you're saying? Okay.

S1 01:43:25.057 I didn't put that together [laughter].

S2 01:43:26.836 All right, all right. I'll just make my own little box.

S1 01:43:29.070 There you go [laughter].

S2 01:43:31.278 Yeah. I probably spelled it wrong too but it's cool. That's my [inaudible].

S1 01:43:37.012 [inaudible].

S2 01:43:39.348 How often do you consume [inaudible]?

[silence]

S2 01:44:32.345 Okay. I don't think [inaudible]-- what [inaudible]?

S1 01:44:36.000 It kind of feels weird to me [inaudible]. We sat here for like an hour, two hours talking about all those types of things and you have to fill that out and put it [inaudible].

S2 01:44:44.230 Yeah. Can you [watch?] it? Can you read it? Are you able to look at it? No? [inaudible] [chocolate?].

S1 01:44:50.096 No, thank you. Can you seal them for me though? I'm not supposed to.

S2 01:44:53.419 Oh. I got to do all--?

S1 01:44:54.632 Yeah. You got to do all the work, right? [inaudible]. You're like, "Oh, [inaudible]. I'm not working for you [laughter]."

S2 01:45:00.869 I haven't licked a envelope since I was like 18 [laughter]. I don't know. Send me a email. I can't do it. I'm so [inaudible].

S1 01:45:11.040 That's okay. Don't worry.

S2 01:45:11.994 Don't worry [laughter].

S1 01:45:12.885 [inaudible]. It's fine. They're going to open it soon anyway.

S2 01:45:16.554 [inaudible].

S1 01:45:18.358 [inaudible] here. Cool. [inaudible]. Okay. So here comes the fun part.

S2 01:45:32.898 What?

S1 01:45:34.186 Money, money, money.

S2 01:45:35.219 Yes. [Forgot all about that?].

S1 01:45:37.019 They forgot about it [inaudible] $20 [laughter].

S2 01:45:43.106 [inaudible] put it in your wallet. You strong. You a strong man.

S1 01:45:49.019 No comments. It's recording [laughter]. Right. Okay. So I just wrote out here the date. Did I spell your last name right?

S2 01:46:04.413 Mm-hmm.

S1 01:46:04.966 Okay. And that I gave you the $40 and the time I [inaudible] this interview, the time that I gave it to you. And on the back here if you could yourself, fill out the rest.

[silence]

S1 01:46:28.089 Awesome. Great. And so the last thing I want to do is, I want to give you this. It's a resource sheet in case you need to access any of these services or have questions, or if you need to guide someone. Again, you probably know a lot about that. Did you have any questions for me at all?

S2 01:46:50.360 No. Do you guys do referrals, like if I can tell people [about that?] and stuff?

S1 01:46:56.370 Absolutely. Please do. Please do. Please do. Yeah. Please do. And even if you don't-- if you're unsure whether or not they are eligible for the study, just send them our way anyway, okay? And let us decide that. We'll do the screening like I did with you over the phone and we'll find out. So, yeah. So please, anyone you [inaudible].

S2 01:47:16.666 Okay. [inaudible].

S1 01:47:19.024 [crosstalk].

S2 01:47:20.283 [crosstalk] had fun. Very enjoyable.

S1 01:47:22.512 Well, thank you. I appreciate it.

S2 01:47:23.979 [inaudible] this recording. Your boss [inaudible].

S1 01:47:27.996 [inaudible].

S2 01:47:31.556 You know.

S1 01:47:31.589 No, I appreciate it. And I really do appreciate your openness and candor and you made this very easy for me actually, so. You were really easy to talk to so it was great. I really appreciate it.

S2 01:47:41.548 Thank you.

S1 01:47:41.589 Thank you. And one last reminder too, if you have any questions, you've got the consent form. You can contact anybody on there or you can feel free to contact me. Okay?

S2 01:47:51.588 Cool. Thank you very much.

S1 01:47:53.686 Now, I'm just going to turn this off [laughter].

IDT 005

Interviewer:	Okay. Good. So can you tell me a little bit about that, like how it is contracted and transmission-

Respondent:	It's-

Interviewer:	... prevention, treatment.

Respondent:	It's prevent with, like, a condom, lubricated or non-lubricated, um, and it's transmitted through sexual, um, intercourse, orally or physical. Um, it's ... you get HIV before it turns into AIDS, um, and it can kill you (laughs).

Interviewer:	For sure, for sure. How'd you find out about this information?

Respondent:	Just through, just through the regular knowledge, um, growing up, in school and shit and like the drug resistant programs and shit and, like, ADAPEP and shit. You know what that is? ADAPEP?

Interviewer:	No.

Respondent:	That's like a sex class.

Interviewer:	Oh, really? Is it i- in school?

Respondent:	Yeah.

Interviewer:	Okay, never heard of it.

Respondent:	Well, it's really long ago.

Interviewer:	Oh, okay.

Respondent:	But it's from [inaudible 00:35:59]

Interviewer:	What grade were you in when you-

Respondent:	Um, I probably was like in third grade.

Interviewer:	Wow, that early?

Respondent:	Yeah.

Interviewer:	Okay.

Respondent:	And still might.

Interviewer:	Nice. Nice. You did, you went to public schools here in [Upstate NY City 1]?

Respondent:	I went to Salem Hyde on Durston.

Interviewer:	I, I'm not from [Upstate NY City 1] originally, so I don't know some of the, um, areas or the schools. Is that a public school or private?

Respondent:	It's a public school.

Interviewer:	Okay.

Respondent:	Um-

Interviewer:	Is it in the southside?

Respondent:	No, it's in [Lancourt 00:36:21].

Interviewer:	Oh.

Respondent:	Um, I went to [inaudible 00:36:24] High School. I went to Lincoln Middle School.

Interviewer:	Um, do you ever hear about HIV and AIDS on, in the media, at all?

Respondent:	Mm-mmm. I haven't really heard about it a lot other than that, actually.

Interviewer:	Okay.

Respondent:	Other than [inaudible 00:36:42]

Interviewer:	Yeah, yeah, great. I know you already told me that you use condoms most of the time, and sometimes you don't, but those are the people that, um, you know. Um, how about ... do you ever get into a situation with someone where they don't want you to use a condom, and you don't know them? Like, how to you navigate that?

Respondent:	Well, that would be not the question. We're not fucking. I'm not gonna get an STD 'cause, like, just to have sex, and I can go have sex with somebody else.

Interviewer:	Right.

Respondent:	We don't have a fucking [inaudible 00:37:21].

Interviewer:	So that you set that down all the way?

Respondent:	I sure do.

Interviewer:	Okay, cool. You ever thought you might be infected, yourself?

Respondent:	Negative.

Interviewer:	You never thought so?

Respondent:	No. I get tested in Room 80.

Interviewer:	Oh, d-

Speaker 3:	At the Civic Center.

Interviewer:	Okay. What do you, where do you go?

Respondent:	Room 80. It's like B80.

Interviewer:	I don't know. I never heard of that, either.

Respondent:	In the Civic Center?

Interviewer:	Nope. Oh, yeah, okay, yes, yes, I did. I have heard of it. I was like you telling me all this information I don't even know (laughs).

(Laughter),

Okay, yeah, I have heard of that place, yeah. Um, what about other STDs. Have you ever had- [crosstalk 00:37:58]

Respondent:	[crosstalk 00:37:57] for my entire life.

Interviewer:	Have you ever been worried about one?

Respondent:	No.

Interviewer:	Okay. Cool. So, on a scale from one to a hundred, zero was, um, the lowest chance and a hundred was the highest chance, what would you say your risk of HIV would be- [crosstalk 00:38:13]

Respondent:	Zero.

Interviewer:	Okay, and you're [inaudible 00:38:14] about that. Okay. Got you. Okay, so now I'm gonna ask you more about PrEP, okay? Um, and your knowledge of PrEP, and again, there's no ... Don't worry. I'm not really testing you. This is for research pr- purposes, so just answer the best to your knowledge and, um, I'm just curious to know what you know and what you don't know. Okay? So, first of all, can you tell me anything about PrEP?

Respondent:	I know that's a prevention pill that prevents STDs.

Interviewer:	And you've never taken it before, right? And, um, how do you found out about it?

Respondent:	[inaudible 00:38:53].

Interviewer:	Oh, okay. Um, so do you discuss it with anybody else? Have you discussed it with anybody-

Respondent:	Well, he's not how I found out about it 'cause I been finding out about on, like, the bus stop.

Interviewer:	Oh, okay.

Respondent:	(Laughs) I found out about it early.

Interviewer:	Oh, cool. Did you know it was from the, um-

Respondent:	It said a PrEP prevention pill. Actually, I seen it on a commercial a while.

Interviewer:	Okay.

Respondent:	Mm-hmm (affirmative)

Interviewer:	And so you knew what it was immediately.

Respondent:	Mm-hmm (affirmative)

Interviewer:	Okay. Just from the commercial. I mean, you saw the advertisement on the bus, as well?

Respondent:	Mm-hmm (affirmative)

Interviewer:	Okay. Nice. And then you j- did you bring it up with [Corbin 00:39:24] first, or, you know, did you talk to Corbin about PrEP because you saw those ads, or did he-

Respondent:	No, well, yeah, yeah.

Interviewer:	Did you have questions about it with him from the advertisement?

Respondent:	No. I'm definitely interested in it, though. I could, I could do my own research when I leave here and see how it's gonna affect me and my body and shit.

Interviewer:	Okay.

Respondent:	Because I really don't really to take prescriptions.

Interviewer:	I see.

Respondent:	You know what I mean? But if this is a prescription that's gonna prevent fucking STDs, why the fuck not? I'm sorry (laughs). I'm sorry (laughs).

Interviewer:	Speak freely. Like I said, I've heard everything, so you ain't shocking me. Trust me. Um, okay, so you're ... so it sounds like you might be concerned about how, what effects it has on your body. Are you talking about side-effects, or ler- long-term effects?

Respondent:	Is there long-term effects?

Interviewer:	I'm not sure. Um, I, I don't, I can't answer-

Respondent:	Is it sex? I need to know all the side-effects (laughs).

Interviewer:	Yeah. Well, I can answer some questions about it, but I'm not a, an expert and so I would say to you but talk to Corbin because he's more of an expert in that than I am and he probably can answer a lot more of those questions for me. From what I understand, though, there are n- just what we know, there's no long-term side-effects. But what possibilities there are-

Respondent:	What short-term program ... what short-term, um, side-effects?

Interviewer:	Um, some people report, um, headache, nausea when you first take the medication just until they get used to it. Yeah, and usually those are brief and they go away, but that's all I know. That's all I can really say for sure. Again, I would talk to Corbin to get the correct information, 'cause there may be others that I don't know. He could tell you more about the length of time and stuff like that, okay?

Respondent:	Mm-hmm (affirmative).

Interviewer:	Um, but don't stop asking me questions. I'll answer the best of my knowledge, okay? Um, do you know anybody who's taken PrEP?

Respondent:	No.

Interviewer:	Um, all right. So you have considered PrEP but you don't know, um, you don't know if that's gonna happen because of the side-effects and stuff, so ... Well, do you know anything about the cost of PrEP?

Respondent:	Um, there would be no cost, right?

Interviewer:	Um, well, it depends. I mean, there's different ways. Again, if you're interested in thing- uh, obtaining PrEP, again, there's different ways to get it, through health insurance, through programs, through ba- sponsored programs and so on and so forth, and again, I'm not the expert on that part. I know y- I know it, it's accessible to people. Um, again, Corbin probably would be the person that you would talk to. He can tell you how, you know, would work for you, given your circumstances. Um, have you heard of anybody ... Have you heard anything at all about the cost of PrEP and how, besides what I just said, how it's paid for?

Respondent:	No.

Interviewer:	Um, so currently, do you have health insurance?

Respondent:	Yes, I have Fidelity.

Interviewer:	And so if you were to go on, um, PrEP, would you ever be, um, concerned about reporting it to your health insurance?

Respondent:	No, why would I do that?

Interviewer:	Why would you? 'Cause they may be paying for it.

Respondent:	Then why would I be concerned about?

Interviewer:	Oh, I don't know. Some people might be concerned that people, other people, know that they're taking PrEP because they're having sex with men.

Respondent:	Well, is that just for you just take it, just for men?

Interviewer:	No, no.

Respondent:	Or does it prevent an STD against a woman, either?

Interviewer:	Well, it prevents HIV, not STD. It helps prevent ... there's other STDs that it doesn't prevent.

Respondent:	So that's the only thing it prevents, is HIV?

Interviewer:	Mm-hmm (affirmative). Right. That's what it's for. So a lot of times, s- I'm just saying that somebody could make the assumption. It may not be true 'cause you can get ... You're absolutely right. You can get HIV from heterosexual sex, as well, but sometimes people make the assumption that, um, that's more for men who are having sex men. They can. I'm just wondering if that is a concern for you.

Respondent:	I'm sorry. What did you just say? (Laughs)

(Laughter).

Just the last part.

Interviewer:	Nothing. Oh, I was just saying, I was just wondering if it was a concern for you i- if you had to report it to your insurance company that you wanted- [crosstalk 00:43:48]

Respondent:	Uh, no, it's not a concern at all.

Interviewer:	Okay. That's all I wanted to know. I- yeah. Um, so, where would you go if you decided to get PrEP? Do you know where you would go to get it?

Respondent:	Well, I'd probably go to the doctor's first (laughs).

Interviewer:	Okay. Do you have a regular doctor?

Respondent:	I have one but I don't see him (laughs).

Interviewer:	Okay. Okay. Where else? Do you know any other places that you could go?

Respondent:	Um, [inaudible 00:44:21]. No, I don't.

Interviewer:	Okay. Um, so, do you know how PrEP is administered? Like, if you decide to take PrEP, do you know what that involves?

Respondent:	No.

Interviewer:	That's fine.

Respondent:	What does it involve?

Interviewer:	It involves taking a pill once a day.

Respondent:	Oh, yeah. Of course I knew that (laughs).

Interviewer:	Okay (laughs). Do you know ... have you heard if there was a liquid or implant or a shot, an injection like that? Have you heard anything like that?

Respondent:	Mm-mmm.

Interviewer:	Just the pill form?

Respondent:	Are there other forms?

Interviewer:	No. I was just wondering if you heard anything. I thought that might be the case. But no. Um, do you think you would be able to take it? If you decided to get on PrEP, do you think you'd be able to, um, take it once a day and keep-

Respondent:	If I'm throwing up, no. I don't vomit and I don't like that word. I don't even say that.

Interviewer:	Okay. Um, would you take it if it were an injection?

Respondent:	Mm-mmm.

Interviewer:	You don't like needles.

Respondent:	Mm-mmm.

Interviewer:	Okay. (Laughs) So you probably wouldn't want to take it if it was like an implant either, like under your skin, like, you know, they put, sometimes they put things under your skin.

Respondent:	No, 'cause they're not [inaudible 00:45:34]

Interviewer:	'Cause what?

Respondent:	[inaudible 00:45:39]

Interviewer:	Do what? I'm sorry.

Respondent:	No, I wouldn't do that either.

Interviewer:	You wouldn't do that, either. Okay. All right. Have you heard anything about the effects, how effective PrEP is? Um, would you, would you take PrEP if it cuts your HIV risk in half? Would that be enough incentive for you to take it?

Respondent:	No.

Interviewer:	It'd need to be more than that?

Respondent:	Hell, yeah.

Interviewer:	Okay, how much-

Respondent:	I'm looking for prevent it 100%.

Interviewer:	Okay.

Respondent:	So what good's a pill gonna do that a p- condom on my dick every time I fuck is not?

Interviewer:	So w- right. So-

Respondent:	I don't like those side-effects. I don't like, I don't like that.

Interviewer:	They're not for everyo- I mean, everyone doesn't experience it. Everybody's different, so I don't know what ... I really don't how likely the side-effects are. Okay, now we have that conversation with Corbin, he probably could tell you a lot more than I could about how likely they are. I do know that some people don't experience some side-effects at all. Some people do, and the type of side-effects ... sometimes it could be just a headache. Um, so, of the side-effects, which ones would be the most concerning.

Respondent:	The vo- the vomiting.

Interviewer:	All right. You already said it. You don't even like to say that word. Um, do you think people l- would look, um ... Do you think people would look at people who would take [inaudible 00:47:07] in a negative light, a positive light, not all, doesn't matter?

Respondent:	The only thing is like benefit that mattered at all. No, think they all look at them in a positive light, you know? They're p- preventing HIV.

Interviewer:	Right. So if you were on PrEP, would you tell people that you were doing that?

Respondent:	No. It's none of their business.

Interviewer:	Okay, but if somebody found out-[crosstalk 00:47:27]

Respondent:	Other than my sexual partner. [crosstalk 00:47:31] Other than my sexual partner?

Interviewer:	Right. Then you think that people would look at you positively if you did decide to ... I'm talking about your sexual partners ... if you told them that?

Respondent:	Mm-hmm (affirmative).

Interviewer:	Okay.

Respondent:	Of course. Of course. Of course they will.

Interviewer:	Um, so, how many more? Let's skip a part, as long as you're over 18. Um, so, I'm going to ... just so you have this ... I'm gonna give you some information about PrEP.

Respondent:	Yeah, I saw it on the wall.

Interviewer:	What's that?

Respondent:	That's a "Yes, sir."

Interviewer:	Okay, so here. That's for you to keep. This is information about PrEP again. You can read this over and it'll give you some of the things that we just talked about, and some of the things might answer some of your questions, but again, I strongly encourage you, if you are considering it, to talk to Corbin 00:23:58] or someone else, your doctor, whomever. Okay? Um, so, you wanna take a minute and just read this over? I want us to make sure you understand-

Respondent:	PrEP is a way of preventing HIV while using anti-HIV medication.

Interviewer:	You can read it to yourself. You don't have to read it out loud.

Respondent:	(Laughs). You don't want me to read it out loud?

Interviewer:	You can if you want to. That's up to you.

Respondent:	The medication works for preventing HIV from getting into your body when you are exposed. PrEP has been shown to be useful for pr- preventing HIV for people with a range of risk for HIV, including sex and injection drugs. Right now, PrEP is only available as a daily pill called TRUVADA ... Did I say that right?

Interviewer:	Yep, TRUVADA.

Respondent:	... which contains two different ... I'm gonna read this [inaudible 00:49:02].

Interviewer:	Yeah, I don't know even how to pronounce those myself. (Laughs). That's two different drugs.

Respondent:	After they say health care provider, I read [inaudible 00:49:36].

Interviewer:	Yeah, I think that's the case.

Respondent:	All right. I have [inaudible 00:50:51].

Interviewer:	No questions? All right, and let's move on. We're almost done, so, uh, thanks again for your patience and everything. Um, but keep that, okay? That's yours. Um, so what do you, um, thinks, think other ... Well, first, let me ask you this question. Are ... do you feel you are connected to, uh, the LBGTQ community, at all? Do you-

Respondent:	No.

Interviewer:	No. You don't have friends or people who are-

Respondent:	I really hate, like, really feminine people. Um, I don't wanna be around them 'cause there's just too much, like, you're gonna be the [inaudible 00:51:28], like, you know, like a little girl.

Interviewer:	Uh-huh.

Respondent:	I really cannot stand ... I don't know, so I really don't have, like, gay friends or no shit like that. Um, I have one gay friend.

Interviewer:	Um, male or female?

Respondent:	Male.

Interviewer:	Oh.

Respondent:	Oh, I do have ... I have my friend, Paula, too. She's a dyke.

Interviewer:	Oh, yeah?

Respondent:	Yeah. She was just on fucking, um, fucking ber- she was on camera on Facebook fighting on her job.

Interviewer:	Fighting at her job?

Respondent:	Yeah, whenever she got arrested.

Interviewer:	Wow. Okay.

Respondent:	But other than that, no, I don't [inaudible 00:52:13].

Interviewer:	Okay. So, do you think in general people know about PrEP, or have-

Respondent:	Yeah, PrEP has definitely made its way around so-

Interviewer:	Yeah, and then you think people have a positive, uh, outlook or view about-

Respondent:	Well, I would definitely think that it should be, like, definitely for females as well. It shouldn't just be for, like, men.

Interviewer:	Yeah.

Respondent:	Like, I don't understand that.

Interviewer:	Yeah. Yeah, I think it, it is. It is. This particular study where we're looking at is how it affects men who have sex with men, but there are definitely ... PrEP can be, um, administered to females, to women, as well.

Respondent:	Mm-hmm (affirmative).

Interviewer:	Yeah. Um, all right, so, I've just got two more things to do and then we can wrap up and then I can kind of pay you and everything, okay?

Respondent:	Good.

Interviewer:	So, these are just some exercises. Um, one is a exercise I'm gonna ask you to do, and I'll go over it, and then the last one is gonna be to fill out the questionnaire, okay? So I'm gonna hand this ... well, actually I have two more exercises. Sorry. I'm gonna hand this to you. Um, so, imagine I had a, a coin, heads and tails on both sides, and when I flip the coin, and whatever it land on, you would receive that dollar amount. So again, if I flip the coin and it landed on heads, you receive this dollar.

Respondent:	This dollar amount?

Interviewer:	If I flip the coin on tails, you would receive ... Oh, I'm sorry. It should go this way. Sorry. If it would land on heads, you would receive this. If it land on, um, tails, you would receive this, in this [inaudible 00:53:59]. Does that make sense?

Respondent:	I guess actually I've [inaudible 00:53:59]?

Interviewer:	No.

(Laughter).

This is just a-

Respondent:	A exercise?

Interviewer:	Yeah, it's just a hypothetical question, so imagine that we did have a coin and you would receive that and I'm asking you, okay, so which row would you pick, one, two, three or four, and I would flip the coin, so if I have to say it ... you said you wanted to pick row one, if it landed on heads, you'd get 50 bucks. If it landed on tails, you'd get 50 bucks, so it's, like, 50-50. If you wanted to ... If I flip the coin and you chose row four, it would land- landing on heads, you would get the $500 but if it landed on tails, you would get zero dollars.

Respondent:	Do you wanna know which one I would choose?

Interviewer:	Which one would you choose?

Respondent:	Well, I, I don't know. I would choose two of them. This is really hard, 'cause I would definitely see ... and [inaudible 00:54:43], and so-

Interviewer:	Because you're what?

Respondent:	I'm really strategic, so-

Interviewer:	Okay.

Respondent:	... Through a one and three, I can win either way. Row four, I'm gonna take a chance on one and zero dollars.

Interviewer:	Right.

Respondent:	So I've had to choose. I'm going to pick ... I would pick row one.

Interviewer:	Row one. Okay.

Respondent:	Or row four.

(Laughter).

Interviewer:	That's so different. Well, which one, if you had to choose just one?

Respondent:	Just one?

Interviewer:	Yeah. Which one would you choose?

Respondent:	I'll choose row one.

Interviewer:	Row one? Okay. Great.

Respondent:	I'll just ... I'll choose row four, but I'm pretty lucky.

Interviewer:	Okay.

(Laughter).

So we gonna go row four?

Respondent:	Yeah.

Interviewer:	All right (laughs). Okay, cool. So just for the record, we chose row four. Okay. So this is, um, just a ranking exercise. What I'm gonna do ... I have these index cards and I have things on the index card that I'll go over them, and what I wanna know is ... I want you to take a look and I want you to rank them and put them in importance, and, um, and this is about your decision about taking PrEP, okay? Um, we can, um, so I want you to put the important on the top, the least important on the bottom, and this is, again, about, um, your decisions and your fact as we're going to, um, taking PrEP, all right? So, it's, um, how you could take it, whether it's pill, injection, etc., how effective it is, effectiveness, what the side-effects are, how often you have to take it, stigma, cost and ease of access, okay? All those make sense?

Respondent:	Mm-hmm (affirmative).

Interviewer:	Okay, so again, in your considering to take PrEP, what would be the most important, um, factors on top and the least important on the bottom.

Respondent:	Define stigma.

Interviewer:	Um, if someone, um ... stigma is somebody who, uh, places negative, um, thoughts or feelings about a certain subject. And if you could think of-

Respondent:	I'm sorry. What did you say again? What was it?

Interviewer:	So, stigma is something like, say, if somebody had a negative thought or feeling about something and, um, and they somehow, uh, communicated that to you, you or to others. Like a lot of people feel stigma about that they were HIV and, you know, positive ... the stigma be attached to them about that. And if you, you, think of anything else you could write-

Respondent:	Is there any ... can these be right or wrong?

Interviewer:	No, there's no right or wrong. This is what's important to you.

Respondent:	Well, what do you ... What do you think?

Interviewer:	No, this is what you think, what's important to you, and the fact is if you were considering, um, taking PrEP, these factors w- would be what's the most important to the least important.

Respondent:	Great, 'cause it really is.

Interviewer:	(Laughs). Kind of.

Respondent:	Do you want me to read these out loud?

Interviewer:	Um, I'll read it out loud once you finish.

Respondent:	I'm done.

Interviewer:	Okay. So you're saying, for you, when you're making a decision about using or not using PrEP, these are the most ... the most important one is on top.

Respondent:	Oh, I started from the bottom.

Interviewer:	Okay, so I'll put it [inaudible 00:58:19]. I probably confused you. Okay. So, this is the most important factor is the one on top, just so we're clear. Okay. So you're saying ease of access ... Oh, I'm sorry, before we get ... Did you have ... do you have any other thoughts, any other consideration that go into your decision about taking PrEP that were not on these cards?

Respondent:	Yeah.

Interviewer:	So, you can write them here. All right, so you're saying ease of access is the most important, cost, how you take it, um, effectiveness, side-effects, how often you have to take it and stigma. So stigma's the least important thing for you. Somehow I knew you were gonna say that.

(Laughter)

Just by ... from our conversation. All right. So that's it in terms of the exercises, and the last thing I need to do is to fill out this questionnaire. All right, so I'm gonna give you the questionnaire, fill it out and then, um, and when you're done, you can fold it and you can put it in this envelope, okay?

Respondent:	(Laughter).

Interviewer:	Let me know if you have any questions.

Respondent:	Yes. Can I get something, I got-

Interviewer:	What's that?

Respondent:	[inaudible 01:00:01] What do you define Cherokee Indian as? Native American?

Interviewer:	Yes.

Respondent:	But we don't have a clan.

Interviewer:	That's fine.

Respondent:	See, [inaudible 01:01:10] ourselves. This is a problem in the current right now, so ... and I have a meeting with my, um ... I'll, I pay taxes. I got a meeting on Friday for my money. I didn't even ... I couldn't get my check Monday.

Interviewer:	Okay.

Respondent:	Fucking, um ... [inaudible 01:01:28]. What [inaudible 01:01:28] other question?

Interviewer:	I don't know what question you're looking at, sorry.

Respondent:	Question eight. What is your yearly household income? Your household includes ...

Interviewer:	Okay, it doesn't have to be, I mean, just mark something that you think it's closest to, so it doesn't have to be exact. Stuck on something?

Respondent:	No, I'm thinking.

Interviewer:	[inaudible 01:02:56] get it stamped by [inaudible 01:03:03].

Respondent:	You know, I'd say one. I'm not sure, but I-

Interviewer:	I don't even know what question you're looking at, so-

Respondent:	It's something [crosstalk 01:03:12]. [inaudible 01:03:46]

Interviewer:	Okay.

Respondent:	(Laughs). [inaudible 01:05:50] drug. I, I'm gonna put marijuana on it, okay?

Interviewer:	Okay.

Respondent:	Right here (laughs).

Interviewer:	That's fine.

Respondent:	'Cause that's the drug that I do.

Interviewer:	That's cool.

Respondent:	This is definitely not ever going to be, um, like, this is always confidential.

Interviewer:	Always confidential.

Respondent:	And like, not even like the probation people have this access.

Interviewer:	No.

Respondent:	But I'm not on probation, but [inaudible 01:06:39] is.

Interviewer:	Yeah, no. So, um, just to go back to that, so the information you are gonna provide to us is always kept confidential. It's gonna be ... that kind of a form's gonna be locked away. Um, and, uh, yeah. So it's always confidential.

Respondent:	You're not [inaudible 01:07:04] several days a week, once a week, more than once a week or less than once a month. Also went to ... (laughs). Never, ever, ever. All right, so, you know, can I just fold it and put it in an envelope?

Interviewer:	That way I can't see (laughs).

Respondent:	Oh, yeah, not much [inaudible 01:08:11]. You want me to seal it?

Interviewer:	Yeah.

Respondent:	[inaudible 01:08:29] like, roaches be around to crawl [inaudible 01:08:30].

Interviewer:	Vegan.

Respondent:	I always feel like roaches be crawling on them.

Interviewer:	(Laughs) I can get some tape if you want to, then seal it that way, it's fine.

Respondent:	Yeah?

Interviewer:	Yes, I need to get some other envelopes. [inaudible 01:08:48] I can tell you, other words, it has been in box, so ...

Respondent:	Um.

Interviewer:	It's been in the box, so no roaches been it. I can tell you that.

Respondent:	I mean, like, in the, in the-

Interviewer:	Oh, in the warehouse and stuff?

Respondent:	Yeah.

Interviewer:	Oh, got you. Yeah, I can't, can't, can't say anything about that. Thanks. Appreciate it. So, I just wanted to point out that you did answer. I, I, It's on your confiden- the confidentiality agreement that you just signed. Um, just so you know that we can't be, um, we can't even be forced to give out that information, even if get a subpoena or the judge or something-

Respondent:	The judge [inaudible 01:09:20]

Interviewer:	Yeah, they, they got your cash. Thank you. All right, I wanted you to see that, all right? So if you decide you wanna disclose information to someone, that's fine. All right, so you have any questions for me?

Respondent:	No, sir.

Interviewer:	All right. I'm gonna turn this off, so we're going-

IDT 006

S1 00:30:32.540 And what about prevention?

S2 00:30:34.665 Well, other than condoms, I know about PrEP and PEP. I don't know.

S1 00:30:41.797 And what about treatment options? Have you heard anything about treatments?

S2 00:30:44.887 I just know that [inaudible] is incredibly expensive, and then in high school, my teacher once said something [inaudible] she was like, "Yeah, honestly, if you get it you can still live a very long time," but just in my head it's a [inaudible].

S1 00:30:58.484 Your head. Right. So where do you get most of your information about HIV and AIDS? Your [crosstalk]--

S2 00:31:05.261 Honestly, it's just from [sort of the university?] classes. I took two sexual diversity classes last year to take care of a what you guys call [inaudible] core. So I took two of them, and I learned about [inaudible] through that class, more so than from high school sex ed.

S1 00:31:22.142 What about through other people like your peers or anything [crosstalk]--

S2 00:31:31.549 No.

S1 00:31:32.261 Can you think of anything? The media? Social media or media in general?

S2 00:31:34.988 I don't think so.

S1 00:31:37.005 Do you see a lot of ads or--?

S2 00:31:38.398 No. I never see any ads.

S1 00:31:40.503 Interesting. Right. Can you tell me about your personal practices with respect to using condoms? I'm interested to hear about such things as how regularly you use them.

S2 00:31:52.012 Almost always.

S1 00:31:55.618 Do you use them for all types of sex?

S2 00:31:58.345 Not oral.

S1 00:32:01.350 Are there circumstances where you don't use them?

S2 00:32:06.470 Sometimes but there's nothing really [specifically?] special. There's no pattern when I don't use condoms.

S1 00:32:14.225 Can you give me a little bit of insight of what that circumstance might be?

S2 00:32:19.138 I don't know.

1 00:32:21.528 Okay. That's fine. Have you ever thought you might be infected with HIV or AIDS?

S2 00:32:28.495 No, no.

S1 00:32:30.592 No. What about other sexually transmitted infections? Have you ever had one or worried that you had one?

S2 00:32:41.119 No.

S1 00:32:45.105 And how worried are you about getting HIV in the future?

S2 00:32:49.199 [crosstalk]--

S1 00:32:49.597 I mean, on a scale from 1 to 100, 0 being the lowest, 100 being the highest.

S2 00:32:53.285 I think like a 10. Honestly, I'm very consistent on using condoms. I have a master stockpile in the bedroom.

S1 00:32:58.648 Great. Great. You said about 10? From 0 to 100?

S2 00:33:02.844 Yeah, 10. Yep.

S1 00:33:03.754 Perfect. Great. So the next set of questions is about what you know about PrEP, which is a daily pill that you can take to prevent HIV. The questions that follow, I'm going to use the term PrEP, obviously, to refer to pre-exposure prophylaxis because it's easier to say. For starters, can you tell me what, if anything, you've heard about PrEP?

S2 00:33:30.682 Somebody explained to me last week - I can promise you that - but I completely forgot. Yep, like you said, you take it every day. It's really good if you don't have it to prevent, and it's good for people whose partner has the disease to keep them from getting it.

S1 00:33:45.799 Did you hear about it before somebody explained it to you last week?

S2 00:33:48.930 I mean, I know what it is, but I've had [inaudible] like I said, I don't remember.

S1 00:33:56.857 Don't remember. Was this a friend or--?

S2 00:34:02.086 No, it's a professor.

S1 00:34:03.110 Oh, okay [laughter]. That's good. I'm glad they're talking about it, at least. Have you ever discussed it with your friends, parents, or teachers other than this one particular professor? Did you hear about it in the media at all? [inaudible] remember any kind of postings or an advertisement about PrEP?

S2 00:34:22.344 No.

S1 00:34:23.231 No? Do you know anybody taking it?

S2 00:34:26.651 No.

S1 00:34:29.639 All right. Do you ever have discussions or have you had discussions with any of your sexual partners about PrEP?

S2 00:34:39.570 Oh, about PrEP, no. Oh, wait. Yeah, no, I did because I was having [inaudible] [sex?] when he was on PrEP.

S1 00:34:45.602 Oh, okay. How did it come up?

S2 00:34:48.597 I asked him why he wasn't using a condom.

S1 00:34:51.512 Okay, got you. And at that point, did you know enough about PrEP to understand what he was saying?

S2 00:34:57.432 Yeah. No, he only said [inaudible]. And he never came inside of me or anything, so.

S1 00:35:06.086 So have you ever considered taking PrEP yourself?

S2 00:35:10.154 Yeah. Because somebody explained. At the doctor's maybe, after I was getting some tests on, that I should really consider it. But after that it was just sort of been in the background. I should, at some point, considered going on it.

S1 00:35:22.341 So you're still weighing the pros and cons of it?

S2 00:35:25.355 Sort of. It's honestly a lot more like insurance-related and I don't want my parents to know that I'm getting PrEP because I don't want them asking me questions about it. Yeah. It's good. The reason why I [inaudible] mention it to my parents [inaudible] medication, medical [inaudible] just like they would see it and then they would ask why I'm taking it.

S1 00:35:47.729 Right. If that was removed then do you think you would be at that high-risk [inaudible]--

S2 00:35:53.214 Maybe. But I also need to be able to afford it, so.

S1 00:35:58.302 That's useful information. So now I'd like to ask you about some of the things that others have told us are important to them when they have considered using PrEP. There is no right or wrong answer to these questions. And if you just find you don't really have anything to say in response to one or more of them, please just answer each question as fully as you can. So what do you know about the cost of PrEP?

S2 00:36:31.214 Nothing [laughter].

S1 00:36:33.334 That's fine. But you just mentioned that affordability--

S2 00:36:38.786 Yeah. At some point I will be out of my parents' health insurance. At some point, I'll start paying for grad school and stuff. At some point, I have to start paying for my own things, so.

S1 00:36:48.540 Got it. But you have no idea of like how much the medication costs?

S2 00:36:52.369 No. Not [currently?].

S1 00:36:53.997 Okay. So have you heard anything about people having trouble to pay for [inaudible]?

S2 00:37:01.644 No. I just remember that Martin Shkreli raised the pill from $13 to $750-- I mean, to some insane astronomical price.

S1 00:37:10.017 Who was that?

S2 00:37:10.375 Martin Shkreli.

S1 00:37:12.258 Oh, that guy.

S2 00:37:13.466 That guy. The Pharma Bro.

S1 00:37:14.625 Got it. Do you know if insurance covers the cost of PrEP?

S2 00:37:21.130 My insurance? I don't think it does. I don't know.

S1 00:37:23.471 Okay. Have you heard anything about other people using insurance, the challenges of using insurance for PrEP?

S2 00:37:31.786 No. Not to my knowledge.

S1 00:37:33.416 Okay. And you said you're on your parent's insurance, right?

S2 00:37:43.836 Mm-hmm.

S1 00:37:46.633 So they are the one who receives the explanation of benefits, I would imagine. Yeah. Do you know what is included in the explanation of benefits in your [own?] insurance?

S2 00:37:59.284 No. Not at all.

S1 00:38:01.392 Never really looked at it?

S2 00:38:03.001 I just know what to give people if I get hit by a car [laughter].

S1 00:38:06.592 That's important. Okay. So what, if anything, have you heard about where you could go if you wanted to access PrEP? Do you think it's easy to get or a challenge?

S2 00:38:17.684 I was told that I can go to the health service offices and they'll make a referral. [inaudible] in Toronto, but I don't live in Toronto anymore. So I guess my answer is sort of in between yes and no.

S1 00:38:31.539 Okay. Do you think that would be difficult?

S2 00:38:37.397 I don't know.

S1 00:38:41.626 So can you tell me what you know about how people take PrEP?

S2 00:38:53.113 Orally. It's a pill.

S1 00:38:56.848 That's it. Do you know if it's once, twice, three times a day, or once a week?

S2 00:39:00.206 It's just once a day.

S1 00:39:03.000 Okay. Do you know if there is an alternative to the pill?

S2 00:39:07.583 There might be, but I'm not sure.

S1 00:39:09.287 What do you think [inaudible]?

S2 00:39:10.488 I think it's just a different type of pill. It's not PrEP. It's some other type of medication.

S1 00:39:14.204 Oh, okay. What about the way you take it? Could it be maybe injection or [crosstalk]?

S2 00:39:19.283 If it's injection, I'm not interested, so [laughter].

S1 00:39:23.177 Implant [inaudible]?

S2 00:39:23.891 Oh.

S1 00:39:24.266 Have you heard of any of those things?

S2 00:39:25.206 No.

S1 00:39:25.730 Okay. Do you know how often you have to go to the doctor's office for follow-up visits if you're on PrEP or anything like that? If you ever decide, do you think it would be a challenge for you to take it on a regular basis?

S2 00:39:47.223 Might be. I've been falling out of habit because my mental health is sort of suffering here. But actually, I just stopped taking that because it was messing with my body.

S1 00:39:58.917 And what was the name of that?

S2 00:39:59.872 Oh, I was taking penicillin last year after a surgery. I just said it was giving me digestive problems. It's due to that. And when I'm about to-- my body gets slightly better but I was [inaudible], I'm just not doing this anymore because this is not fun at all.

S1 00:40:16.009 Right. But you were doing [inaudible] so good. That wasn't the problem, being consistent and--

S2 00:40:22.551 Not really.

S1 00:40:23.279 --[crosstalk] the fact that it was having a negative effect on your body?

S2 00:40:25.036 Yeah.

S1 00:40:26.108 Got it. So you seem pretty organized to me so that, I mean, I think if you decided you wanted to take care of that you seem like a person that would be pretty consistent.

S2 00:40:33.638 Probably.

S1 00:40:34.862 In thinking about, if you were to take it, would it make a difference if it was pill form, injection--

S2 00:40:45.482 Pill. I'm not shoving a needle into my veins.

S1 00:40:49.613 What about an implant? [crosstalk].

S2 00:40:51.124 I don't even know what that is. [crosstalk].

S1 00:40:51.926 [crosstalk] underneath your skin and you wouldn't have to take care of it. It would just be there forever.

S2 00:40:56.678 No. I would rather just-- yeah [laughter].

S1 00:41:01.546 Okay. Got you. I think that's pretty clear. What about, have you heard anything about the effectiveness of PrEP?

S2 00:41:11.646 No.

S1 00:41:12.365 No. How effective do you think it is?

S2 00:41:17.552 I'd say it's pretty effective.

S1 00:41:20.662 If you had to guess a percentage of effectiveness.

S2 00:41:25.386 90 something percent, I think. 90 to 99 percent.

S1 00:41:29.525 Cool. So how effective would something have to be for you to-- or how effective would PrEP have to be for you to consider taking it?

S2 00:41:41.820 I think 90% is good.

S1 00:41:43.573 90%?

S2 00:41:44.196 Yeah, at least.

S1 00:41:55.373 Do you think you would take it if it cut your risk in half or is that not good enough?

S2 00:41:59.404 No, not really, because it costs something.

S1 00:42:03.406 Okay. Have you heard anything about side effects?

S2 00:42:08.120 No, I have not.

S1 00:42:14.680 Which side effects would be more concerning to you if it did present? Maybe that's not [an appropriate?] question to ask. Would side effects be concerning-- something that you would be concerned--

S2 00:42:30.629 Depends on what the side effect is.

S1 00:42:36.681 Do you think the people that you meet as possible sex partners--

S2 00:42:47.118 I'm sorry?

S1 00:42:47.869 People that you meet as possible partners. Sexual experiences and partners. Do you think they would think they favorably or unfavorably about a person using PrEP?

S2 00:42:57.027 Probably it would be favorably. It helps us [get in?] something, so yeah.

S1 00:43:05.237 Because I don't really know. Just kind of asking. Is there anything on these apps, like Grindr or anything like that, like ads [or something?]?

S2 00:43:14.014 What? To get on PrEP?

S1 00:43:16.181 Or just in general. Is anything popping up about PrEP? Anybody have discussions about it?

S2 00:43:21.532 I mean, I received a message to text you from Grindr, but [outside?] that, no. Not really.

S1 00:43:26.943 Okay.  So you have this one person that you talked about, who did disclose that they were using PrEP.

S2 00:43:41.571 Mm-hmm.

S1 00:43:42.117 Has that been your only experience with--?

S2 00:43:44.347 What? Someone who's disclosing that they're using PrEP?

S1 00:43:45.605 Mm-hmm.

S2 00:43:46.721 [Most of these?e] told me that they're on PrEP.

S1 00:43:48.051 Oh, okay. All right. How do you feel about that? I mean--

S2 00:43:50.508 No. I don't care. [crosstalk]--

S1 00:43:51.814 Favorably? Unfavorably?

S2 00:43:52.087 Yeah. [No?], yeah. Favorable. Favorable.

S1 00:44:00.521 Would you prefer someone to be on PrEP, if you-- yes?

S2 00:44:04.263 Yes.

S1 00:44:06.537 Okay. Well, we're moving through these pretty quickly, so that's good. So I'm going to give you just a fact sheet about PrEP, just so you can hold on to that. Give you some more information, if you like. Oh, actually, sorry. Do you mind taking a few minutes, just to read that over? And let me know if you have any questions.

S1 00:44:36.922 Mm-hmm.

[silence]

S2 00:46:33.986 Okay.

S1 00:46:33.869 Do you have any questions?

S2 00:46:34.794 No.

S1 00:46:35.718 After reading that, does it change your mind at all, anything about PrEP?

S2 00:46:40.568 Not really.

S1 00:46:41.534 Nothing comes up for you with that? To what extent do you think others in the community, the LGBTQ community, think about PrEP? What do you--

S2 00:46:52.538 They see it positively.

S1 00:46:57.261 And what about what do you think they know about? Do you think that it's a general knowledge?

S2 00:47:01.225 I think they would just know that it exists. I don't think they know how [inaudible] work, but I think they know it exists.

S1 00:47:09.113 And in your opinion, what do you think stops people from getting on it, mostly?

S2 00:47:13.066 Money. I guess people just thinking that they can just use condoms.

S1 00:47:19.090 Just easier to do that. All right, well thank you so much again for sharing. We really breezed through this pretty quickly. So I have a couple more things to give you before we wrap up, okay?

S2 00:47:31.694 Okay.

S1 00:47:33.153 So it's a little bit different than what we've done so far. So these are some cards that I wrote. It's a ranking exercise, so I'm going to give you the index cards, and what I'd like you to do is rank them in the-- well, these are factors of what people told us are important about taking PrEP. And so I'd like you to rank them in the order that you think are the most important on the top and the least important on the bottom. So I'm just going to leave them on [inaudible]. This is important to the decision-making about taking PrEP. So stigma, how often you have to take it - meaning once a day, once a week, [inaudible] - side effects, if there are any, effectiveness we talked about, cost, ease of access, how you take it, again, orally, injection, that kind of thing. So take a moment, and here's some blank cards here if you think of something that I haven't listed that might be significant factors that go to decision-making.

S2 00:48:48.251 So is it just like least--?

S1 00:48:50.455 Least on the bottom. Least important factor.

S2 00:48:52.767 Yeah, okay. So, I'll put these three at the bottom. I'll put these two [inaudible]. Actually, [I'll just?] put this one in the middle.

S1 00:49:02.653 Sure.

S2 00:49:02.920 I put--

S1 00:49:04.033 [inaudible].

S2 00:49:06.517 It's more--

S1 00:49:08.553 On top of these?

S2 00:49:09.307 Yes. On top of these.

S1 00:49:10.333 So it's more [important than these?].

S2 00:49:10.745 Yeah. I'm going to do-- or no. So ease of access, and stigma, and then cost.

S1 00:49:19.658 So this is the most important--

S2 00:49:20.746 I feel like that's the most important.

S1 00:49:21.818 Okay. And least important is on the bottom.

S2 00:49:23.141 Yeah.

S1 00:49:23.888 I'm just going to read them off. So cost is the most important, then stigma, ease of access, side effects, effectiveness, how often you take it, and how you take it.

S2 00:49:36.717 Yeah.

S1 00:49:37.404 Okay. Great. Is there anything else that you think that might go into the decision-making [inaudible]?

S2 00:49:42.552 No, because I think what I'm thinking [inaudible] right now is covered by stigma, so.

S1 00:49:49.543 Covered by what?

S2 00:49:50.134 Stigma.

S1 00:49:50.307 Stigma. Oh, okay. Okay. Great. Thank you. So the next thing I have is another exercise. It's fairly quick. So imagine I'm going to flip a coin, and you'll get what is indicated in the heads column if it comes up heads, and you'll get what is indicated in the tails column if it comes up tails. Which row would you pick on the table that you're looking at? You can only pick from one row. So for instance, if I flipped a coin,  you picked row one, and it landed on heads, and you said heads, it would be-- you would get $50. If it landed on tails, you would get $50. And if you picked row four, it landed on heads, you would get $500. It landed on tails, you would get zero. Does that make sense? Okay. So which row would you pick--?

S2 00:50:50.025 Two. What does this have to do with PrEP [laughter]?

S1 00:50:55.833 Well, the study, which a lot of times when we have these qualitative studies, we ask questions about what we're specifically looking for, but we ask things that are related in some kind of way because [crosstalk]--

S2 00:51:08.965 Right. So how is that related?

S1 00:51:10.270 This assesses your risks. So we try to do this, and then we cumulative answers from everyone we interview, trying to see if there's a pattern here. So sometimes we can collect data and we don't really have a specific goal in mind when we're collecting this data. But sometimes things emerge and it can lead us to something. It could spur another study, so we try to cover all the bases we can. All right. So thank you so much for doing that. And so the last thing we're going to do is to give you the self-administered questionnaire. [inaudible]. Oh, here it is. So take your time. Unfortunately, I don't have an envelope, because you're supposed to finish this and put it envelope and seal it. But if you fold it up, I promise to you, as soon as I get out of here, I will put it in an envelope and seal it.

[silence]

S1 00:53:00.522 [inaudible].

S2 00:53:03.277 Six months. Today's the 4th. So that would be--

S1 00:53:15.831 [inaudible] [83?]. Okay.

[silence]

S1 00:54:03.645 [inaudible].

[silence]

S1 00:54:43.655 [Okay?]. Okay. Thank you. Do you have any questions for me?

S2 00:54:58.816 [Not really?].

S1 00:54:59.445 [I'm going to?] also give you this. It's a resource guide. [inaudible]. And--

S2 00:55:06.573 Hurray.

S1 00:55:06.474 --[inaudible] [laughter]. If you could just fill out this receipt for me.

S2 00:55:14.193 Like I received the incentive?

S1 00:55:15.538 Yes.

S2 00:55:16.193 All right.

S1 00:55:18.631 And today is-- [inaudible]. Print and sign there.

[silence]

S1 00:55:38.367 Awesome. Thank you so much.

S2 00:55:39.903 Mm-hmm.

S1 00:55:46.213 I hope things get better.

S2 00:55:47.462 [inaudible] [up?]. They won't.

S1 00:55:50.938 Yes. They will.

S2 00:55:51.719 [They never do?].

IDT 010

S2 51:35

Awesome. I know you said you've seen stuff on apps. They mention if HIV and [crosstalk]--

S1 51:46

Yeah, yeah.

S2 51:47

Do you see much stuff on the media? Do you take note of that? I'm always curious because [I don't know?]--

S1 51:52

I don't. I don't see it a lot.

S2 51:53

Yeah. I don't see--

S1 51:53

The one thing that I can think of was in this show, How to Get Away With Murder. There was one character that was HIV-positive, and his boyfriend had to go through a whole process to [inaudible], get his [inaudible]. Maybe he was on this. I have no idea. But he was taking medication before they could get together again. He wanted to be safe. The other partner wanted to be safe. Something else, though. You don't see it much, though. It's not a lot of representation.

S2 52:29

Yeah. I've found the same thing, and it's kind of surprising to me. And then having done this study and doing interviews for a while, it seems to be the same. But I remember years ago, you would see billboards and commercials and lots of stuff. I guess maybe [inaudible] a little bit. I bet that makes some--that might have something to do with it. But I'm still surprised, though, about that. So can you tell me something about your personal practices with respect to using condoms?

S1 53:02

Oh, sure. Always use a condom, personally. I want to make sure I'm being safe. I want to make sure that the other person is being safe. But yeah. That's a policy of mine.

S2 53:16

Okay. That's good. Good. Have you ever thought you might be infected with HIV?

S1 53:23

I haven't been tested, and I want to be tested, just so I do know. But I don't believe so.

S2 53:32

Okay. What about other sexually transmitted infections?

S1 53:37

I don't think I've ever experienced one, no. But again, I haven't been tested. But I mean, it's definitely possible.

S2 53:45

Sure. Have you ever had a scare, worried that you might have HIV or any other sexually transmitted infection?

S1 53:50

Oh, definitely. Definitely. There was one time, and I just remember, it stung when I peed, and it was a little inflamed. But I kind of self-diagnosed myself. I was kind of like, "I don't think it's anything." I went online, looked it up. I don't even know what it was, but it went away. So I was like, "Cool."

S2 54:09

How long ago was that?

S1 54:11

Maybe a year ago.

S2 54:19

Great. Again, I'm not judging you. Just for your own information, so there seems to be a spike in sexually transmitted infections now. And some of them don't present symptoms. So you could have that kind of a pain, and it could away, and you may think, "Well, so?" If that ever happens again, don't hesitate, though.

S1 54:38

Right.

S2 54:39

Go to the clinic. [inaudible] services here. I don't know what they're like. It's good to be safe. Other than that [inaudible] there's a spike. I think I was reading something recently about gonorrhea and a lot of people have been testing. But that's one of the reasons why it's spreading is because people [inaudible] the symptoms, so. Take my dad hat off [laughter]. So on a scale of 1 to 100, with 0 being with the lowest chance of getting HIV and 100 being the highest chance, where on the scale do you think you might fit about getting HIV in the future?

S1 55:14

Probably the lower end. But I could put a number on it. Just 25 maybe? It's definitely possible as with anything. But--

S2 55:26

[crosstalk]?

S1 55:26

Yeah. I'm trying to be as safe as possible. So I hope I can go and be on the lower side of the spectrum.

S2 55:34

Sure. Absolutely. I hope so too. Okay. So the next set of questions is about what you know about HIV pre-exposure prophylaxis or PrEP, which is a [daily?] pill taken to prevent HIV. The questions that follow, I'm going to use the term PrEP to refer to it because it's just easier to say, for sure [laughter]. So can you tell me what, if anything, you've heard about PrEP?

S1 56:02

I've heard that it suppresses the disease or the virus. Or it can. I heard that it doesn't work for everyone. I don't know if that's necessarily true. But like you said earlier that the goal is to the count or whatever it's-- I don't know the correct terminology. But just make sure that it's undetectable and sort of lies dormant.

S2 56:29

And who would PrEP be for?

S1 56:33

People that are diagnosed with HIV and may have the virus, I guess.

S2 56:44

And how have you found out about PrEP?

S1 56:49

I think it came up in a conversation once with my friends. We were just talking about it and somebody had said something about, "Oh, hey. Did you hear about this? This is going on." I was like, "Oh, wow. That's interesting." If we're moving in the right direction. So I was super excited about that and--

S2 57:11

Awesome. Anybody else that you've discussed PrEP with, teachers, family members? Doctors?

S1 57:24

Yeah. Did I? I think I did because we have a pretty open communication, my choir director and actually from the time I was 6 to the 12th grade. She moved up with us. Went to our high school. But yeah, she has been super supportive since day one. And we've got a really good friendship, and we were always talking about gay stuff and education and all that.

S2 57:57

That's great.

S1 57:58

So it's definitely possible it popped up in conversation at least once. I don't remember a specific time, but I'm completely positive that it did.

S2 58:07

Okay. Great. Do you know anyone, personally, taking PrEP?

S1 58:13

No, I don't.

S2 58:14

Okay. And none of the people that you've hooked up with, have had encounters with, have ever mentioned PrEP to you or--?

S1 58:25

Not that I know of. No.

S2 58:26

Okay. So can you tell me if you've ever considered taking PrEP, and what factors would have affected your decision-making?

S1 58:35

Sure. Yes, I have definitely considered taking the medication. I get wary with medications. I don't want to be hooked on something my entire life if don't have to be. Unless it's life-threatening. I'll be like, "Absolutely. If this going to--" but yeah. That's kind of my philosophy on medications. But I definitely have to do more research into it, though, if I was considering it.

S2 59:01

When you say, "Hooked on something," are you maybe not conscious of how it affects your body?

S1 59:07

Right. Yeah. Maybe not so much like addictive. I guess I couldn't-- I don't know, actually, if you could addicted on this, if it has that kind of--

S2 59:17

No.

S1 59:18

But more of, if I do start taking it, then I will have to continue taking it. Yeah. And side effects like with any medication. But I have to do more research and look into it more before I make a final decision.

S2 59:35

Great. So you are considering it?

S1 59:38

Definitely, definitely.

S2 59:39

Okay. Let's see. Do you know anything about-- so if you are considering it, do you know anything about how to obtain it? Do you know anything about the costs?

S1 59:52

No.

S2 59:52

Do you know if your insurance covers the cost of those medications?

S1 59:56

I'm not sure if my insurance does. I haven't really looked into that. I do remember-- was it two, three years ago? That some guy making the medication or something. But I don't know if it was necessarily PrEP, but it was a treatment for the disease and expect the cost. But it was a whole, big-- yeah. Yeah, I remember that. But I don't remember-- I don't know exactly how much it would cost or if my insurance covers it or anything like that.

S2 60:22

Okay. And so then, I would say that you've never heard of anyone, at all, getting it, obtaining it--

S1 60:30

Not personally, but you definitely hear stories and all the--

S2 60:35

In terms of?

S1 60:36

So I guess less privileged people that don't necessarily have the money or they can't get access to it when they need to. Just being more available, yeah.

S2 60:51

Would it worry you to use your health insurance to pay for PrEP, if it did?

S1 61:02

No.

S2 61:02

You wouldn't worry about maybe your mom seeing it on the bill or something?

S1 61:08

I mean, I would definitely talk to her before I'd do--

S2 61:11

Before you'd do it?

S1 61:11

Yeah. Just be like, "Hey."

S2 61:14

Cool. And you wouldn't worry about people that you didn't know making judgments about you?

S1 61:19

I don't really care.

S2 61:19

Okay [laughter].

S1 61:21

Judge me all you want [laughter].

S2 61:22

Cool.

S1 61:22

I'm still the same person [laughter].

S2 61:23

Cool. Are you under your mom's health insurance still?

S1 61:26

I believe so. I think so, yeah.

S2 61:31

Do you know what [inaudible] benefits are?

S1 61:36

No.

S2 61:37

Okay. So each insurance might have a different kind of-- explains what it covers and what it doesn't cover. So that's what it be, so. If you are on your mom's insurance you can-- and you can probably look it up online and see what it does and what it doesn't cover. What, if anything, have you heard about where you can go if you want to access PrEP? Do you think it's easy to get? [inaudible].

S1 62:03

I have no idea. I haven't a clue.

S2 62:09

Do you know what you have to do to take PrEP? In other words, is this something that you would do daily, weekly? Do you know if it's a pill, injection, some kind of implant under the skin? Do you know--?

S1 62:25

I do know that it is daily. I knew that. I assume it's a pill. I haven't heard of anybody injecting themselves or anything like that. So yeah, those are the two things that I--

S2 62:38

And if you came to the decision, the conclusion, that you did need to do that, do you think that could do that daily, take a pill daily?

S1 62:47

I hope so.

S2 62:48

[inaudible] that.

S1 62:49

I hope so. I'd try. I sometimes take allergy medication and sometimes when it gets bad, some days I just forget.

S2 63:00

Yeah. That's real. I mean, it certainly [inaudible]. It really depends on how you [inaudible]. There's certain people who are really good with being regimented and sticking to [the stuff?] and then other people like me who's like, "Holy [inaudible] [laughter]." And what would you think about taking PrEP if it were an injection rather than the daily pill? No?

S1 63:25

I really don't like needles.

S2 63:26

No [laughter]. It seems to be--

S1 63:29

I don't like needles.

S2 63:30

A lot of people say that.

S1 63:32

I mean, I could definitely do it if I had to, but--

S2 63:36

Right, if it was your life depends on it or something?

S1 63:37

Yeah.

S2 63:37

I get what you're saying. What would you think about taking PrEP if it were an implant under the skin [laughter]?

S1 63:45

Ow [laughter].

S2 63:47

That says it all.

S1 63:49

Is this a daily thing or just once?

S2 63:50

No. There are some medications, they put an implant underneath the skin and it's like a time-released thing and it releases the medication to your bloodstream.

S1 64:00

Would it have to be refilled or--? This is all hypothetical, of course.

S2 64:04

Yeah. Absolutely.

S1 64:06

It depends really. If you totally put it in next month to get-- reopen it and take it out? Maybe not.

S2 64:15

I'm getting lightheaded thinking about it [laughter]. Cool. What, if anything, have you heard about the effectiveness of PrEP? How effective do you think it is for preventing HIV?

S1 64:29

Well, like I said, I have heard that it doesn't work for everyone the same way, but I've heard that it does suppress the virus pretty well when it does work.

S2 64:42

And how effective would PrEP have to be for you consider taking it? In other words, what if it reduced your chances of getting HIV by half, or three-quarters, or 99%?

S1 64:56

Right. Well, I guess it would I would have to weigh the cost value. If I feel this is going to be very strenuous on my life and it was cost wise and it didn't have that great of sort of effective rate, then I guess I would kind of brush it aside. But if it was cheap and accessible-- and even if it didn't have that great of an effective rate, it would still be worth it to get it. Prolong your chances of getting the disease. But if it was higher, even better.

S2 65:32

Right. I get you. So if it worked like-- okay, if it reduced your changes by 20%, you still would consider taking it?

S1 65:41

If I could afford it, yes.

S2 65:42

If you could afford it [inaudible].

S1 65:42

Yes.

S2 65:45

Okay. What about side effects? Have you heard anything about side effects with it?

S1 65:53

No.

S2 65:55

Just thinking about medication in general, about side effects. What type of side effects would bother you the most or worry you the most about taking any kind of medication?

S1 66:04

Probably [crosstalk] because I guess I could say that I already kind of experience those, so just to kind of like amplify that, especially depression too. Again, diagnosed depression. So anything to amplify those kinds of negative effects I probably would want to shy away from. Which is why I have been. So adamant about not taking certain medications like, for example, for the depression, I've been asked, "Would you like to get on medication to try and stabilize the balance or whatever?" I was like, "No." I've experienced people who have gotten onto medication. It's a long journey, finding the correct dosage, all that. So it's like, "I don't know. Maybe I should stick to what I do know and shy away from that."

S2 67:03

I see. I see. Interesting. Okay. Cool. Do you think friends or potential partners will look favorably or badly on people who take PrEP?

S1 67:17

For me, personally, and my friends, good.

S2 67:23

Favorably?

S1 67:23

Yes. It's always for us, for me, as long as you're taking the correct steps in your life to better yourself, good for you. I don't have any judgments where it's like, "Oh. You're what?" or, "You're taking that? Why?" It's more of it should be celebrated. It should be, "Good for you."

S2 67:47

Yeah. So you make your friends feel the same way.

S1 67:50

I definitely do. Yes.

S2 67:51

Very cool. How would you feel if a partner disclosed that he was taking PrEP? Would it make a difference if that person was new, casual, regular sex partner?

S1 68:00

No. Not really. As long as I'm still safe as I was, if not safer now.

S2 68:07

If somebody was taking PrEP, would it make a difference if they-- would you want them to tell you? Or is that something that they don't think they need to disclose to you?

S1 68:14

If you had the virus, definitely. I would want to know, for sure, where my safety lies. But if you're just on it to be extra careful or whatever, I don't necessarily need to know that. If you want to tell me, great. Good. But I don't think it really affects anything.

S2 68:34

If you decided, yourself, that you were going to take PrEP, do you think that you would disclose that to people?

S1 68:41

Again, if I did have the virus, I would probably be like more open. I'd be like, "Hey. Look. This is my circumstances. Just want to make sure that you're okay with it. I am on PrEP. It's undetectable. If it is, just see where it goes from there." But if I was just taking it to be safe, I probably wouldn't, I guess.

S2 69:05

Okay. And if you were just taking it as a preventative measure and your decision not to tell someone, is that just because it's-- I mean, what's behind that decision not to?

S1 69:20

I guess it's kind of sort of like-- I just feel like it wouldn't affect anything, sort of. It's kind of different than using other preventative measures, like condoms or something, for example, because both parties have to be-- an understanding of what's going on when you use a condom. Whereas, if I'm just-- I guess if it was like a relationship, sure. Absolutely. I'd be like, "Hey. Yeah. I'm taking PrEP. I'm on the medication." But if it was just somebody that I met for the first time and we wound up hooking up, or something like that, I don't know if I would just because I feel like it wouldn't change anything that's going on or affect the other person in any way.

S2 70:11

Right. That makes sense to me. Is there anything else that you want to share about your feelings about PrEP, or anything? Burning questions or [inaudible]?

S1 70:30

No. Not really.

S2 70:31

Think we've covered it all?

S1 70:32

Yeah.

S2 70:33

Okay. You doing okay with this so far?

S1 70:34

Oh, yeah. Definitely.

S2 70:35

Okay. Good. All right. So I'm going to give you this. This is a PrEP fact sheet. And I would say take a moment. Read it over. There's some things that might clear up on some of the things that you've said-- and, again, I'm not testing you [inaudible]. But I certainly want you to be clear about [inaudible]. So take a moment. Just read this over and then we can talk about that a little bit.

[silence]

S2 71:21

If you run across something that doesn't make sense, you can ask me too.

S1 71:23

Sure.

[silence]

S1 72:29

Sure.

S2 72:30

Okay. So having read that, does anything-- maybe comments, any [inaudible]--

S1 72:37

It clears up some stuff.

S2 72:38

Okay. Like what?

S1 72:39

With the side effects. For example, about 1 in 10 people on Truvada-- is that how you pronounce it?

S2 72:45

Mm-hmm. Mm-hmm.

S1 72:47

Get headaches, nausea, or both. Excuse me. They tend to go away after a week of taking the medication for the first time. About 1 in 3,000 people could have a kidney problem resulting from Truvada. These problems almost always go away if the medication is stopped. Okay. And then there's something about bone-- where was that? Down [inaudible]. Where was it? Oh, okay. There's a very minimal decrease in bone density on Truvada that recovers after stopping PrEP. What does that mean?

S2 73:25

What's that?

S1 73:25

There's a very minimal decrease in bone density.

S2 73:29

So do you know what bone density is?

S1 73:31

Sort of.

S2 73:32

So bones are porous, and so decrease in bone density just means that there's a-- you know what osteoporosis is?

S1 73:48

Yeah.

S2 73:49

So that's a decrease in bone density. It's a disease, right? So then your bones become more brittle, [crosstalk]. But it's a very minimal--

S1 73:56

Sure.

S2 73:56

And just so you know, with any of these side effects-- well, you read that some of the most of the common ones are the nausea, headaches, and stuff like that. It's about your body adjusting, and that will go away in about a week, like it says. But the other thing that you read, about the kidney-- some people having kidney issues. I can't say 100%, because I don't know, but I think when you're on PrEP, that's something that you are tested for to make sure that your kidneys are healthy, and so on, so forth. And I don't know if they do any tests about bone density. There's a bone density test that you can have, but I don't recall anyone having to take that [inaudible] test [on the?] PrEP. Is that all?

S1 74:37

Everything else seemed pretty straightforward.

S2 74:39

Okay. Does this information at all change your mind at all anything about what we talked about PrEP?

S1 74:47

I mean it seems successful if most New York State insurance claims cover it. But I'd still have to do more research into it, for myself.

S2 75:00

And the side effects that you did [inaudible], does that change your mind at all about whether or not you would take PrEP, in your decision right now?

S1 75:14

Well, the headaches and nausea, that's kind of-- I already experience that. I have chronic migraines. So that wouldn't really make a difference, unless if they got super bad. But if it's only going to go away in a week, it would be worth it. Kidney problems, sure. That could be scary. Bone density. I don't know if it would-- it might. It might, but I don't think so. I'd definitely be cautious, but--

S2 75:47

Yeah, yeah. And just like any kind of medication. I think you would want to make sure that your body is not experiencing negative side effects and just stay true to [inaudible] the doctor. To what extent do you think others in the gay, bisexual, trans community know about the facts about PrEP, the effects?

S1 76:03

I don't know personally. I just hope that most people know the basics, what it is, what it does, how to get it. But, yeah.

S2 76:19

And do you think increased awareness of these facts would [prevent people's stresses?] about PrEP?

S1 76:23

Definitely, definitely.

S2 76:25

And why would that be?

S1 76:26

Just the more exposure to things you have, the more you start to learn about it, you start to realize the benefits and the drawbacks, and just everything. And people would definitely be able to make more of an informed decision about things, other than just hearing about, "Oh, PrEP? Okay. Well, I know what that is, but I don't really know what that is." So increased sort of knowledge about that would definitely be beneficial.

S2 76:54

Great. I wanted to just point something out to you while I'm thinking about it. So during our conversation you've mentioned a couple times about the use of PrEP for people who already have HIV. That wouldn't be the case.

S1 77:08

Really?

S2 77:08

So PrEP is only for people who are HIV negative. So it's a preventative measure, so.

S1 77:15

Oh, okay.

S2 77:16

Now, a person who is HIV or who has the virus, they can take the same medication, but it wouldn't be considered PrEP. Just so you're clear with that. Okay.

S1 77:27

Would that be PEP? Or what's that? Where did I see that?

S2 77:34

No.

S1 77:36

[crosstalk] to take something called PEP which is a short course of HIV medication to prevent getting-- oh, okay, never mind.

S2 77:44

Right. So they're both preventative measures, so it's the same medication as someone who would use, but PrEP and PEP are for preventative measures, too. [inaudible] so you weren't incorrect when you were saying this medication could suppress the virus if you have the virus or if someone had the virus. You weren't incorrect in that sense, but they wouldn't be calling it. They would call it something else, which I [inaudible] right now. So just [inaudible]. Yeah, I think we're done with that part, so thank you, again, for sharing your thoughts. I really appreciated this and it's been a fantastic experience for me. It's been really easy to talk, so it's been great. We're almost done. I just have two short exercises to do. I'm going to hit pause for a second. I need to go, just grab some water. One second. Okay. Sorry about that. Thanks for being patient. So I have two-- again, just really two short exercises and then the questionnaire and then we'll be done. Thank you, again. Do you have any questions for me before we move forward? Any thoughts, even, on what we've discussed? Anything involving what we've discussed already?

S1 79:09

No.

S2 79:10

Okay, good. I always have trouble just bringing this [inaudible] but I will try my best because it's very easy. This is a ranking exercise and so it goes like this. So imagine I'm going to flip a coin and you'll get what is indicated the heads column if it comes up heads. And you'll get what's in the tails column if it comes up tails. And I want to know which row you would pick. In other words, if you flipped a coin and you chose row one, you would get $50 for heads, $50 if you landed on tails. Or if you chose row four, you would get $500 if it came up heads, and you would get $0 for tails. So which row would you choose?

S1 79:50

Probably either row three or row four. Because you're not losing anything. It's all potential. Yeah, I'd say row four.

S2 80:05

Row four?

S1 80:06

Yeah.

S2 80:06

Okay. I like it [laughter]. Great.

S1 80:13

Go big or go home. [laughter]

S2 80:15

Go big or go home, right. I like that. Okay. Great. The last exercise, if I have is-- one second. So I have this-- I'm sorry, that was a risk. Sorry, that was a risk preference exercise, now we're going to do the ranking exercise. Sorry. I apologize [inaudible].

S1 80:45

Oh, that's okay.

S2 80:46

So I'm using index cards. I have factors that people have told us that are important to them in making decisions about using PrEP. And so I want you to take a look at these carefully. And then I want you to rank them from place-- put them in order for me, would be absolute most important aspect of PrEP to least important, with the most important on the top and the least important on the bottom.

S1 81:12

Sure.

S2 81:13

I have some plain cards if you think of anything else that might be an important factor. So we have cost, stigma, ease of access, side effects, effectiveness, how often you have to take it - once a day, once a week. So on and so forth - and how you can take it, by pill, injection, or that skin thing that we talked about that was horrible [laughter]. So take a look at all these.

S1 81:42

I'll do it this way, just from most effective to-- or whatever.

S2 81:49

So most effective is on the top and [crosstalk]--

S1 81:51

Oh, on the top, you want me to layer them, or--?

S2 81:53

Yes.

S1 81:53

Okay. So, cost.

[silence]

S1 82:25

All right. So cost, ease of access, [inaudible]. All right.

S2 82:43

Good.

S1 82:44

Seems about right.

S2 82:45

Okay. Do you have any other thoughts about what might go on to other factors that you might want to list here? You don't have to--

S1 83:02

Yeah, I'm just--

S2 83:04

Take your time.

S1 83:05

Maybe not.

S2 83:07

Okay. So let's see--

S1 83:10

Is this just about PrEP and taking it personally or--?

S2 83:18

Mm-mmm.

S1 83:17

Okay, never mind.

S2 83:18

So factors that someone would weigh in terms of whether or not they would-- the decision whether or not to go on PrEP. And these are the factors. So would that change this order at all?

S1 83:29

No, no, no. Because I was just thinking maybe education or sort of a--

S2 83:36

[inaudible]?

S1 83:38

Yeah, sort of like-- what's the word? It's representation. Sort of.

S2 83:45

I see. I got you.

S1 83:47

But I guess it kind of falls [crosstalk] stigma.

S2 83:50

Yeah, I guess you could make an argument for that. So let's see what you've got here. So effectiveness is the most important. Definitely. So effectiveness, cost, ease of access, how you take it, how often you have to take it, side effects, and stigma.

S1 84:09

Yeah.

S2 84:10

Great. Perfect. Okay, so that's it for that. So the last thing I'll have you do is our self-administered questionnaire. So take your time. Read that. Do you need a pen?

S1 84:31

Yes, please. Sorry.

S2 84:36

Here you go. I'll give you this one. I'll use this one.

S1 84:39

Thank you.

S2 84:40

And then when you're done, you can fold this up and then place it in this envelope.

S1 84:45

Sure.

S2 84:47

Thank you.

[silence]

S1 85:30

And also, if something doesn't make sense, just let me know.

S2 85:32

Okay.

[silence]

S1 89:11

Cool. Yep.

S2 89:14

Okay. So you just want to hold that up and [crosstalk]?

S1 89:16

[Is this right?]? How should I do this?

S2 89:22

I know. I hate doing those things [laughter]. [inaudible] three.

[silence]

S2 89:54

[inaudible]. Thank you very much. And the last order of business [laughter]. For you. So sorry for the singles.

S1 90:02

[inaudible] sure.

S2 90:04

35. One, two, three, four, five.

S1 90:07

Awesome.

S2 90:07

40 for you. If you wouldn't mind just [signing?] the receipt to me.

S1 90:10

Sure.

S2 90:12

And sign here.

[silence]

S1 90:31

There you go.

S2 90:31

Great. And last, last order of business [laughter] is this is our resource sheet for services here in the [Upstate NY City 2] area.

S1 90:40

Oh, awesome.

S2 90:41

Okay. So you can hold onto that. If you have any questions about what you do to research PrEP, or if you have any questions about-- any more questions or how to obtain it, or how to get on it, or whatever the case may be. There you are. Or anything else. Any last thoughts? Any questions or anything for me?

S1 90:59

No. We covered most of everything.

S2 91:02

Great. Cool. Well, I appreciate your time.

S1 91:04

Thank you.

S2 91:05

Great. Thank you. I hope it was a good experience for you too.

S1 91:09

Yes, it was.

S2 91:10

Cool.
