# Supplementary material for: Perceived access to PrEP as a critical step in engagement: A qualitative analysis and discrete choice experiment among young men who have sex with men
Source: PLoS One. 2022 Jan 26;17(1):e0258530. doi: 10.1371/journal.pone.0258530 (PMC8791519; doi:10.1371/journal.pone.0258530)
Supplement: Attachment — Submitted filename: Perceived access to PrEP as a critical step in engagement_JCS review 2_4_21.pdf [file pone.0258530.s005.pdf]

Reviewer comments on Perceived access to PrEP as a Critical Step in Engagement

The authors have conducted an elegant mixed-methods investigation that deepens our understanding of PrEP uptake among young MSM. The use of the discrete choice experiment methodology is novel, and the manuscript should inspire other investigators to employ this technique more widely in HIV prevention research. The compelling findings the authors present have important implications for the development of PrEP programs serving young MSM. The manuscript is very well-written and there are only a few places that needed strengthening. Mainly, the authors should provide additional details about the qualitative analysis methods used in the first component of the study, and about the recruitment methods for both components of the study.

Lines 119-139 describing the in-depth interviews should be strengthened. The authors did not describe how participants were recruited for the in-depth interviews, nor did they include full demographic information on the group of interview participants. The authors should add these demographic data as a table or in the main text. The authors should also consider describing relevant demographic characteristics of the interviewers (i.e. were they also young MSM or otherwise peers or near peers of the interview participants?) and findings from any reflexive activities that were conducted during the interview data collection cycle. If the authors conducted a quantitative assessment of inter-rater reliability between interviewers, they should include that data in this section as well. Additionally, the description of the qualitative analytic techniques the authors used warrants a more detailed description. The authors can do this by providing a short definition of both grounded theory and of inductive methods, as well as describing how these methods were used throughout the interview cycle.

Line 157-158 The authors should provide information about the types of social media applications that were used to recruit participants. Were these traditional social media sites like Instagram and Facebook or were they hookup/dating apps like Grindr and Scruff (or a combination of the two). Delineating this can help the reader better understand the sampling strategy.

Line 532 very minor typo- should read “which was also not addressed.”
