# Supplementary material for: Perceived access to PrEP as a critical step in engagement: A qualitative analysis and discrete choice experiment among young men who have sex with men
Source: PLoS One. 2022 Jan 26;17(1):e0258530. doi: 10.1371/journal.pone.0258530 (PMC8791519; doi:10.1371/journal.pone.0258530)
Supplement: Attachment — Submitted filename: Response to Reviewers_19May21.docx [file pone.0258530.s006.docx]

Elizabeth Asiago-Reddy, MD, MS

Associate Professor and Chief, Div. of Infectious Disease

Medical Director, Inclusive Health Services

SUNY Upstate Medical University

Syracuse, NY 13210

May 19, 2021

Dear Reviewers and Editorial Team,

We thank you for your favorable and challenging comments to our manuscript. We have addressed the requests and suggestions and we believe that the manuscript has emerged all the stronger. We have responded to each of the points queried below, in red text, in order to assure that all elements were addressed.

We look forward to hearing back from you.

Sincerely,

Elizabeth Asiago-Reddy

Elizabeth Asiago-Reddy and the DiCE4PrEP team

Journal Requirements:

When submitting your revision, we need you to address these additional requirements.

1. Please ensure that your manuscript meets PLOS ONE's style requirements, including those for file naming. The PLOS ONE style templates can be found at

https://journals.plos.org/plosone/s/file?id=wjVg/PLOSOne_formatting_sample_main_body.pdf and

https://journals.plos.org/plosone/s/file?id=ba62/PLOSOne_formatting_sample_title_authors_affiliations.pdf

These elements have been changed. However, there were 437 changes to the original document as a result, so I did not include these in the tracked changes as they would have made it difficult to follow the changes made. Other changes as recommended by reviewers have been left in track changes.

2. Please include additional information regarding the survey or questionnaire used in the study and ensure that you have provided sufficient details that others could replicate the analyses. For instance, if you developed a questionnaire as part of this study and it is not under a copyright more restrictive than CC-BY, please include a copy, in both the original language and English, as Supporting Information."

The questionnaire submitted to the Institutional Review Board has been placed into the supplementary material. Please keep in mind that, for our Discrete Choice Experiment, elements were randomly varied (except for excluded elements) as described in the protocol. Therefore the precise Discrete Choice Experiment (DCE) for each participant was unique. All possible elements are listed in the protocol in Table 1 and an example DCE is shown in Figure 1.

3. Please clarify the date range for which you collected data. The current manuscript states "From July to January, 2018" which seems like an error."

This was an error and has been corrected to reflect July, 2018-January, 2019.

4. Please list the name and version of any software package used for statistical analysis, alongside any relevant references. For more information on PLOS ONE's expectations for statistical reporting, please see https://journals.plos.org/plosone/s/submission-guidelines.#loc-statistical-reporting.

This has been updated.

5. We note that you have indicated that data from this study are available upon request. PLOS only allows data to be available upon request if there are legal or ethical restrictions on sharing data publicly. For information on unacceptable data access restrictions, please see <http://journals.plos.org/plosone/s/data-availability#loc-unacceptable-data-access-restrictions>.

We have discussed this with our IRB and we are able to provide data as requested as long as it is de-identified. Relevant datasets to be uploaded with the package.

In your revised cover letter, please address the following prompts:

a) If there are ethical or legal restrictions on sharing a de-identified data set, please explain them in detail (e.g., data contain potentially identifying or sensitive patient information) and who has imposed them (e.g., an ethics committee). Please also provide contact information for a data access committee, ethics committee, or other institutional body to which data requests may be sent.

b) If there are no restrictions, please upload the minimal anonymized data set necessary to replicate your study findings as either Supporting Information files or to a stable, public repository and provide us with the relevant URLs, DOIs, or accession numbers. Please see http://www.bmj.com/content/340/bmj.c181.long for guidelines on how to de-identify and prepare clinical data for publication. For a list of acceptable repositories, please see http://journals.plos.org/plosone/s/data-availability#loc-recommended-repositories.

We will update your Data Availability statement on your behalf to reflect the information you provide.

6. Please include a caption for figures 3 and 4.

This has been done.

7. Please include captions for your Supporting Information files at the end of your manuscript, and update any in-text citations to match accordingly. Please see our Supporting Information guidelines for more information: http://journals.plos.org/plosone/s/supporting-information.

This has been done.

Additional Editor Comments (if provided):

The authors present an innovative study design. Major revisions are required for further clarification for the audience.

Abstract

-Please include the number of in-depth interviews and the number of online surveys conducted within the methods section. In the results section of the abstract, please remove the online survey sample size.

Numbers of surveys conducted are standardly a part of the results, not the methods. One might make enrolment targets in the methods, but actual numbers of individuals enrolled is a result. We are hesitant to make this change.

-In line 55, a more accurate term for insurance enrollment could be “enroll in” new insurance.

This has been changed.

Reviewer #1 presents with important points regarding the Intro and Methods sections.

I will address this in comments to Reviewer #1.

-Insurance is not a modifiable category as Reviewer #1 presents. Please revise these sections.

Please see the response to Reviewer # 1.

-DCE sampling has limitations and the authors should incorporate this in the manuscript

This was partially addressed in the discussion but an additional line has been added to acknowledge this limitation, starting on line 171.

-Reviewer #1 brings up a good point regarding the subgroup analyses in the results section.

-Please comment on the high behavioral risk categorization that the authors discussed and as reviewer #1 brings up as a limitation.

Please see the response to Reviewer #1.

-As suggested by Reviewer #2, there needs to be more detail on the methods and the definition of grounded theory.

This has been updated in lines 129-149.

-In line 246, please provide a reference.

This has been updated and is now on line 271

Results

-Table 1 requires a demographic breakdown of participants.

Table 1 is not related to the participants; the main table in the results describing online survey participants does have a demographic breakdown of participants.

Information about the demographics of the in-depth interview participants has been added to the text and a table to the supplementary index, Appendix 3.

-Clarification of the definition of PrEP access is needed.

I am not sure which section this refers to, but following along in the results, I have changed the wording to read “participants’ ability to access PrEP.”

-Clarification on ranking is required.

Additional clarification has been added.

-In line 297 regarding ACA, please provide a reference.

A reference has been added.

Discussion

-Reviewer #1 indicates that the results are overstated; please revise the discussion section in response to Reviewer #1's comments. Please comment on the study limitations regarding not having a national representative sample for the in-depth qualitative interviews and their influence on the online survey.

Please see response to Reviewer #1.

-In line 521, the authors should highlight study limitations instead of disadvantages.

This has been updated.

Figures

-The Figure text is not legible.

This has been updated.

[Note: HTML markup is below. Please do not edit.]

Reviewers' comments:

Reviewer's Responses to Questions

Comments to the Author

1. Is the manuscript technically sound, and do the data support the conclusions?

The manuscript must describe a technically sound piece of scientific research with data that supports the conclusions. Experiments must have been conducted rigorously, with appropriate controls, replication, and sample sizes. The conclusions must be drawn appropriately based on the data presented.

Reviewer #1: Yes

Reviewer #2: Yes

________________________________________

2. Has the statistical analysis been performed appropriately and rigorously?

Reviewer #1: Yes

Reviewer #2: Yes

________________________________________

3. Have the authors made all data underlying the findings in their manuscript fully available?

The PLOS Data policy requires authors to make all data underlying the findings described in their manuscript fully available without restriction, with rare exception (please refer to the Data Availability Statement in the manuscript PDF file). The data should be provided as part of the manuscript or its supporting information, or deposited to a public repository. For example, in addition to summary statistics, the data points behind means, medians and variance measures should be available. If there are restrictions on publicly sharing data—e.g. participant privacy or use of data from a third party—those must be specified.

Reviewer #1: No

Reviewer #2: Yes

________________________________________

4. Is the manuscript presented in an intelligible fashion and written in standard English?

PLOS ONE does not copyedit accepted manuscripts, so the language in submitted articles must be clear, correct, and unambiguous. Any typographical or grammatical errors should be corrected at revision, so please note any specific errors here.

Reviewer #1: Yes

Reviewer #2: Yes

________________________________________

5. Review Comments to the Author

Please use the space provided to explain your answers to the questions above. You may also include additional comments for the author, including concerns about dual publication, research ethics, or publication ethics. (Please upload your review as an attachment if it exceeds 20,000 characters)

Reviewer #1: The authors have conducted an elegant mixed-methods investigation that deepens our understanding of PrEP uptake among young MSM. The use of the discrete choice experiment methodology is novel, and the manuscript should inspire other investigators to employ this technique more widely in HIV prevention research. The compelling findings the authors present have important implications for the development of PrEP programs serving young MSM. The manuscript is very well-written and there are only a few places that needed strengthening. Mainly, the authors should provide additional details about the qualitative analysis methods used in the first component of the study, and about the recruitment methods for both components of the study.

This has been updated, in lines 112-131. A Table listing key details of the in-depth interview participants has been added to the supplementary material.

Lines 119-139 describing the in-depth interviews should be strengthened. The authors did not describe how participants were recruited for the in-depth interviews, nor did they include full demographic information on the group of interview participants. The authors should add these demographic data as a table or in the main text. The authors should also consider describing relevant demographic characteristics of the interviewers (i.e. were they also young MSM or otherwise peers or near peers of the interview participants?) and findings from any reflexive activities that were conducted during the interview data collection cycle. If the authors conducted a quantitative assessment of inter-rater reliability between interviewers, they should include that data in this section as well. Additionally, the description of the qualitative analytic techniques the authors used warrants a more detailed description. The authors can do this by providing a short definition of both grounded theory and of inductive methods, as well as describing how these methods were used throughout the interview cycle.

This has been updated, starting on line 133.

Line 157-158 The authors should provide information about the types of social media applications that were used to recruit participants. Were these traditional social media sites like Instagram and Facebook or were they hookup/dating apps like Grindr and Scruff (or a combination of the two). Delineating this can help the reader better understand the sampling strategy.

This has been updated to reflect platforms used and reasons for the choices.

Line 532 very minor typo- should read “which was also not addressed.”

This has been updated.

Reviewer #2: This purpose of this paper was to assess preferences for PrEP access in the United States among YMSM through discrete choice experiments informed by in-depth interview findings. Overall, this was a very interesting and novel approach to assess how to improve PrEP uptake among a population with higher HIV incidence; however, there are design and methodologic concerns that present major barriers to the generalizability of the findings. I have the following suggested edits and comments for the authors:

Introduction

Overall, the introduction is well written. However, a stronger argument needs to be made for the structural barrier attributes included in this analysis, as insurance status is not easily “modifiable”. This can be acknowledge/justified more strongly in the introduction (as an introduction to the concepts) or in the methods. While insurance status can change, it would more likely need adjustments in policy versus being modifiable on an individual-level. To inform future research, this work identified a clear determinant for PrEP uptake, but interventions targeting insurance coverage may be limited (especially in states that have not expanded Medicaid).

I wholeheartedly agree with the comment that insurance is not always easily modifiable, certainly by any given individual. I have updated the language in the introduction to reflect this. There are other elements presented in this DCE that also pertain to policy and not just to individual choice [whether or not online PrEP care is available (keep in mind, this was conceived and conducted pre-pandemic), whether or not certain time frames are required prior to services, what types of drugs manufacturers ultimately decide to market]. Certainly, DCEs can be used to determine preferences for currently available or future goods or services. Ideally when it comes to future policies or services, or future marketable goods, DCEs help to prioritize what consumer would like in the future that they may not currently have available now. Other PrEP DCEs cited in our bibliography also looked at both immediately available and possible future types of goods and services.

This was also acknowledged to participants. The following comments were shown to participants prior to beginning the Access DCE.

- Some people may find it easy to get access to PrEP. They may already have medical insurance covering PrEP and they have a clinic close by that offers PrEP. Others might have more difficulties accessing PrEP.
- In this next section, we’d like you to think about aspects of PrEP access such as insurance coverage options, how long it would take you to get on PrEP, and where you would get your care. We’ll be showing you pairs of options for accessing PrEP. Choose which set of options you like best. If you don’t like either, select the “No PrEP” option.

This is now included along with all of the rest of the questionnaire in the supplementary materials. This acknowledges to participants that we are trying to get at not just what is immediately possible to them now, but what might be possible to them in the future. Additionally, this manuscript offers the opportunity to consider our data as insurance policy is being created.

Methods

Major concerns are related to the in-depth interviews being conducted with participants from Upstate New York and the DCE surveys being distributed nationally. If attributes selected were partially informed by the qualitative findings (esp. structural barriers), this leads to bias in the attributes selected for DCE in the survey because of the IDI participants contextual barriers related to the region in which they live as well as factors related to their identity/race/ethnicity (which is not provided). If additional attributes were selected based on literature review, then this should be clearly outlined in the methods section as to which attributes were informed by the researchers’ findings versus by the literature review. Also, the authors should describe how they decided on which PrEP characteristics to have participants rank for in-depth interviews?

It is acknowledged that this was not ideal however perhaps less major. Several of our in-depth interview participants were from other parts of the country originally. The available literature at the time was reviewed extensively to determine whether alternate or additional attributes should be added. There was little information to address the specific concerns raised by our participants regarding perceived access to PrEP. It seemed logical that if these were significant concerns in our area, they could be even more significant in other parts of the country, since New York State had been putting forth numerous efforts to make PrEP more accessible. By creating strict inclusion criteria for both surveys and having the national DCE survey participants match the in-depth interview participants in terms of age, lack of past PrEP use, and risks for PrEP, it is reasonable that the items in the DCE would reflect areas of concern for a broader geographical sample. I have updated a sentence about this limitation in our paper.

Additionally, the authors do not describe if they had a target sample size based on the number of levels within attributes as well as choice sets created. Also, I would have considered a geographic subgroup analysis, based on looking at structural barriers. Insurance coverage and acceptance of costs may differ based on the region in which YMSM live.

Our target sample size was at least 150 participants in order to extract meaningful differences in preferences. The strict inclusion criteria and the filtering of surveys deemed untrustworthy resulted in a a small proportion of overall responses being eligible for participation and/or being included in the analysis. This likely limited our ability to observe differences between subgroups. It is important to note, however, that the participants overall had many similarities, in that they were all young U.S. men who have sex with men who are PrEP eligible but reported never taking PrEP. Additionally the curves as seen in Figures 4-5 are quite discreet indicating a high level of accuracy of the data. We did consider a geographic analysis but ultimately did not pursue it.

Page 5, line 105 – “relative importance of PrEP attributes”. I would recommend delineating attributes related to PrEP itself and service delivery attributes to help with clarity.

This has been further clarified.

Page 13, line 241 – Why is the “high behavioral risk” group > 10 anal sex partners in the last 6 months while never using condoms? I understand wanting to show high risk, but based on prevalence and CDC guidelines, I am unsure if such a high number is necessary to illustrate risk.

Objectively, all our participants were high risk, since they were all eligible for PrEP. Indeed, among those eligible for our study, sociodemographic risk may be more likely to predict HIV acquisition in the U.S. than more detailed specifics on behaviors. The goal was, within the group of participants we recruited, to identify whether those who were reporting the greatest amount of risky behavior were any different in their preferences for PrEP. In the end, they were not objectively different so it turned out not to be meaningful in our sample, either due to truly not being meaningful or to a type II error given the somewhat low sample size. We could remove this from the paper altogether but we left it in to acknowledge that it was considered.

Results

Major concerns in the results section for the in-depth interviews, was not describing demographics for participants in text or in a table. Also, it was very hard to read Figure 1-3. I would recommend the authors make the text more legible and look at different color scales.

The in-depth interview results have been updated; this is mentioned above as well.

I am not 100% certain what the challenges are with Figures 1-3 since they appear very clear on my end. I will review this further with the PLOSOne editorial team in case there is something happening as the files are transmitted or compiled.

Page 15, Line 307 – “This example reveals how stigma and privacy concerns, potentially related to being…” This seems like a sub-theme related, but distinct from privacy concerns with parents. The concern that participant will be judged for being on PrEP by “people” may support the need for other interventions addressing stigma among late adolescents who are not able to realistically obtain privacy in billing for PrEP when on their parents insurance plan.

We agree with this observation though it was outside the scope of this particular research study. It was actually quite interesting that our in-depth interview participants rated stigma as the least of their concerns during the ranking exercise, but it did emerge as a theme in the in-depth interviews. This may be due to stigma being difficult to categorize into a simple attribute, or due to lack of clear understanding on the part of the participants as to what was meant by stigma.

Page 16, Line 326 – The label “Prioritization of PrEP Elements” should potentially be changed to attributes for clarity sake.

This has been changed.

Page 17 – Overall with the ranking process, I had a few questions? Again, how were attributes included for ranking selected, was this based on literature review and if so it should be clearly stated? How was access defined by the authors for participants, because it could have several meanings? This is made even more confusing the sentence, “Assuming high effectiveness, access issues – cost (insurance coverage, privacy) and ease of access – were highly important, since from the standpoint of participants, some the specifics of use did not really matter if you could not get it.” This sentence makes it appear that cost was included in access.

It has been updated in the manuscript that the attributes were chosen after discussion with local PrEP providers, community educators, care coordinators, and stakeholders at the hospital and health department sites in the community where the research was conducted.

Also, the sentence quoted has been updated for clarity.

Discussion

Overall, the major findings outlined in the discussion are stated too strongly in that YMSM may not be pursuing PrEP due to costs and insurance coverage. I think the authors need to better present the context when drawing major conclusions from the results. This paper largely focused on structural barriers to PrEP access, which are unique and need to be addressed. However, stigma and other individual- and community-level barriers are also important in improving PrEP access. Additionally, this survey was done with a sample not entirely reflective of current HIV epidemiology in that it did not have a large enough representation of BMSM and persons living in the South, with bias in it being conducted over an app which likely skewed results in preference for on-line PrEP services. In saying this, the results still add to the literature in the type of analysis done and the impact it may have on future intervention development. This is one piece of a complex puzzle when trying to improve PrEP uptake among YMSM.

Page 23, Line 451 – “Results from in-depth interviews that informed the design of the DCE provided key explanatory evidence that concerns about affordability supersede concerns about mode of administration, frequency of administration, side effects, and stigma associated with taking PrEP.” This is an example of drawing conclusions not necessarily supported by the results. First, the IDIs were done to inform DCE and, therefore, cannot be explanatory but are exploratory. Also, I am not sure how based on IDI findings the authors have come to the conclusion that affordability supersedes the factors listed above. That is not the goal of qualitative research and these factors were not included in the DCE analyses.

These are valid comments and attempts have been made to address them by re-wording the discussion.

I would recommend rewriting the discussion section with these overarching comments in mind.

In all, this paper was well written and provides a unique analysis to better understand how to improve PrEP access among YMSM. I believe that with the major revisions suggested above, it will be a great addition to the literature.

________________________________________

6. PLOS authors have the option to publish the peer review history of their article (what does this mean?). If published, this will include your full peer review and any attached files.

If you choose “no”, your identity will remain anonymous but your review may still be made public.

Do you want your identity to be public for this peer review? For information about this choice, including consent withdrawal, please see our Privacy Policy.

Reviewer #1: Yes: Justin C. Smith

Reviewer #2: No

[NOTE: If reviewer comments were submitted as an attachment file, they will be attached to this email and accessible via the submission site. Please log into your account, locate the manuscript record, and check for the action link "View Attachments". If this link does not appear, there are no attachment files.]

While revising your submission, please upload your figure files to the Preflight Analysis and Conversion Engine (PACE) digital diagnostic tool, https://pacev2.apexcovantage.com/. PACE helps ensure that figures meet PLOS requirements. To use PACE, you must first register as a user. Registration is free. Then, login and navigate to the UPLOAD tab, where you will find detailed instructions on how to use the tool. If you encounter any issues or have any questions when using PACE, please email PLOS at figures@plos.org. Please note that Supporting Information files do not need this step.
